# Base-promoted deacylation of 2-acetyl-2,5-dihydrothiophenes and their oxygen-mediated hydroxylation

**DOI:** 10.3762/bjoc.22.13

**Published:** 2026-01-28

**Authors:** Vladimir G Ilkin, Margarita Likhacheva, Igor V Trushkov, Tetyana V Beryozkina, Vera S Berseneva, Vladimir T Abaev, Wim Dehaen, Vasiliy A Bakulev

**Affiliations:** 1 TOS Department, Ural Federal University, 19 Mira str., Yekaterinburg 620002, Russia; 2 N.D. Zelinsky Institute of Organic Chemistry, Russian Academy of Sciences, 47 Leninsky ave., Moscow 119991, Russia; 3 North-Ossetian State University, 46 Vatutina st., Vladikavkaz 362025, Russia; 4 North Caucasus Federal University, 1 Pushkin st., Stavropol 355009, Russia; 5 Sustainable Chemistry for Metals and Molecules, Department of Chemistry, KU Leuven, Celestijnenlaan 200F, Leuven B-3001, Belgium

**Keywords:** 2-acetyl-2,5-dihydrothiophenes, deacylation, 2-hydroxy-2,5-dihydrothiophenes, transformations

## Abstract

Solvent-dependent transformations of polysubstituted 2-acetyl-2,5-dihydrothiophenes to the corresponding 2-hydroxy- or deacetylated derivatives are described. The treatment of a methanolic solution of the dihydrothiophene substrates with sodium methoxide afforded the deacylated products. Conversely, the treatment with sodium ethoxide in an oxygen saturated ethanolic solution produced 2-hydroxy substituted 2,5-dihydrothiophenes.

## Introduction

Oxidative transformations are an important area of modern organic synthesis [1], producing a broad range of valuable synthetic products for the industry. A variety of catalytic reactions were developed for the oxidative conversions of unsaturated compounds [2–5], alcohols [6–7], alkanes [8–10] and more complex molecules [11]. Rearrangements of the oxidized compounds are equally important transformations [12].

**Scheme 1:**
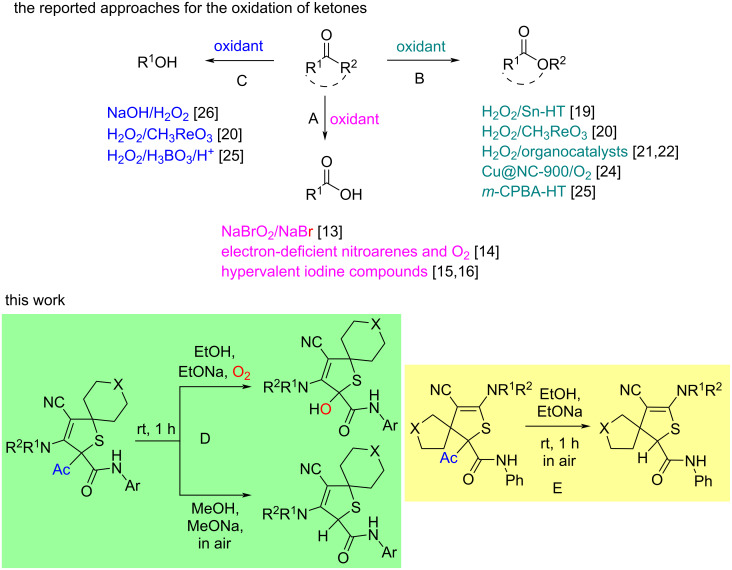
Previous reports (A‒C) and our work (D, E).

Oxidation of compounds containing a carbonyl group into carboxylic acid derivatives can be divided into two large groups: direct oxidation and oxidative rearrangements. Direct oxidation of ketones includes C‒C-bond cleavage, and carboxylic acids are predominantly formed. This can be achieved by the treatment of acyclic ketones with hypohalites [13], in the nitroarene-catalyzed oxidation with oxygen under basic conditions [14] or by the use of hypervalent iodine compounds (Scheme 1A) [15–16].

Oxidative rearrangements of carbonyl compounds are based on Dakin [17] and Baeyer–Villiger reactions [18] and their modifications.

Cyclic and acyclic ketones were oxidized to afford lactones and esters, accordingly, involving catalytic reactions with hydrogen peroxide [19–22], oxygen [23–24] or with *m*-CPBA (Scheme 1B) [25–26] or non-catalytic transformations [27].

The few known oxidative transformations of *o*- or *p*-hydroxy-substituted aromatic ketones, that in most cases lead to phenols, involve the use of hydrogen peroxide as an oxygen source (Scheme 1C) [20,25–26]. Bernini et al. have developed a catalytic system, containing hydrogen peroxide/methyltrioxorhenium and an ionic liquid, to oxidize acetophenones to afford phenols [20]. Junjappat et al. found that hydrogen peroxide activated by boric acid can act as oxidant for the direct conversion of aromatic ketones to phenols [25]. Hocking has described the oxidation of *o*-hydroxyacetophenone and some benzophenones with an aqueous alkaline hydrogen peroxide solution [26]. The key steps of oxidation of ketones into phenols include: a) nucleophilic addition of the hydroperoxide anion to the carbonyl carbon; b) [1,2]-aryl migration in the formed tetrahedral intermediate to afford formate ester; c) hydrolysis of the latter to form phenols [28].

The development of methods for the construction of heterocycles and their modification is an important area of organic synthesis [29]. Although the Dakin oxidation has become a convenient tool for the preparation of phenols from aromatic ketones, several specific approaches have been developed for the synthesis of hydroxylated heterocycles [30–34].

The deacylation of ketones is also another important direction of their transformation [35–38]. Dihydrothiophenes can be considered as analogs of organic sulfides. Accordingly, in oxidative reactions they are also easily oxidized to the corresponding sulfoxides [39–40]. Despite the fact that the synthetic applications of dihydrothiophenes are being actively studied [39–46], their oxidative functionalization that does not disrupt the heterocycle or oxidize sulfur has not been previously reported.

Dihydrothiophenes exhibit a broad spectrum of biological activity [47–49]. In this regard, the development of new routes for their modifications with the use of inexpensive and easily available reagents is an important task.

Recently, we have developed a copper(I)/rhodium(II)-catalyzed method toward two types of regioisomeric 2,5-dihydrothiophenes **1** and **4**, containing an acetyl group [50]. In this work, to evaluate the synthetic utility of these compounds (the scope is presented at page S3 of Supporting Information File 1) we have studied their transformations in ethanolic or methanolic solutions in the presence of sodium ethoxide or methoxide, accordingly. As a result, catalyst-free oxidation under mild conditions of 2-acetyl-2,5-dihydrothiophenes into 2-hydroxy-substituted products (Scheme 1D) or the deacetylated products (Scheme 1E) have been developed.

## Results and Discussion

Dihydrothiophene **1a** was selected as a model substrate for our optimization study (Table 1).

**Table 1:** Optimization of the transformation of dihydrothiophene **1a**.^a^

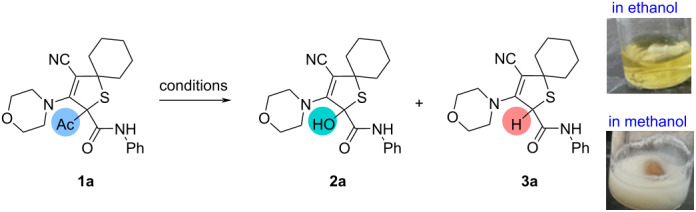					

Entry	[M] or base (equiv)	Solvent (mL)	[O]	Acid (mL)	Yields of **2a**/**3a**, %

1	Na (1)	EtOH (2)	O_2_	‒	22/36^b^
2	Na (2)	EtOH (2)	O_2_	‒	21/27^b^
3	Na (2)	EtOH (2)	O_2_	HCl (0.25)	30/35^b^
4	Na (5)	EtOH (2)	O_2_	HCl (0.25)	41/0
**5**	**Na (5)**	**EtOH (2)**	**O** **_2_**	**HCl (0.25)**	**51/0** **^c^**
6	Na (5)	EtOH (2)	O_2_	‒	28/trace
7	Na (5)	EtOH (2)	O_2_	HCl (0.25)	44/0^c,d^
8	Na (5)	MeOH (2)	O_2_	HCl (0.25)	trace/71^c^
**9**	**Na (5)**	**MeOH (2)**	**O** **_2_**	**HCl (0.25)**	**0/78**
10	Na (5)	iPrOH (2)	O_2_	HCl (0.25)	35/0^c^
11	Na (5)	*n*-BuOH (2)	O_2_	HCl (0.25)	46/0^c^
12	Na (5)	TFE	O_2_	HCl (0.25)	0/75
13	NaOH (5)	EtOH (2)	38% H_2_O_2_ (0.5)	‒	0/62^c^

^a^Conditions: dihydrothiophene **1a** (0.12 mmol), dry solvent, [M] or base, rt, 1 h. Water (2 mL) or/and acid were added after evaporation of solvent. Isolated yields after centrifugation in Et_2_O (2 × 1 mL). ^b^Products were isolated as a mixture. ^c^Oxygen was bubbled (1 min) after Na dissolving. ^d^Ice bath. TFE – trifluoroethanol.

Initially, this compound was treated in ethanolic solution (2 mL) at room temperature in air for 1 h in the presence of sodium ethoxide prepared from 1 equiv of sodium. After the reaction had completed, the reaction solution was concentrated under reduced pressure and the residue was quenched with water and extracted with dichloromethane (DCM). Centrifugation of the concentrated extract in Et_2_O afforded a mixture of products **2a** and **3a** in 22 and 36% yields, accordingly (Table 1, entry 1). When the loading of sodium was increased to 2.0 equiv, the yield of deacylated product **3a** was slightly decreased to 27% (Table 1, entry 2). 2-Hydroxy-substituted dihydrothiophene **2a** was formed additionally in comparable yield (21%, Table 1, entry 2). When acid (HCl) was added after quenching the residue with water, the yields of products **2a** and **3a** were increased (30 and 35%, Table 1, entry 3).

The selective formation of 2-hydroxy-2,5-dihydrothiophene **2a** in 41% yield was achieved when using 5 equiv of sodium and 0.25 mL of HCl (Table 1, entry 4). In the oxygen saturated solution, the product **2a** was obtained with increased yield (51%, Table 1, entry 5). When no acid was added, product **2a** was isolated in decreased yield (28%, Table 1, entry 6). Conducting the reaction in the oxygen saturated ethanolic solution at 0 °C afforded product **2a** in a slightly decreased yield (44%, Table 1, entry 7). To our surprise, when ethanol was replaced with methanol, the deacylated product **3a** was isolated as the major product in 71% yield (Table 1, entry 8). In this case, dihydrothiophene **2a** formed only in a trace amount. In contact with air this reaction proceeded more selectively, and the pure product **3a** was isolated in 78% yield (Table 1, entry 9). Hydroxy-substituted product **2a** also formed in solution of iPrOH or *n*-BuOH, and this product was isolated in 35 or 46% yields, respectively (Table 1, entries 10 and 11). In a solution of TFE the deacylated product **2a** was formed in 75% yield (Table 1, entry 12). Carrying out the reaction under Hockings conditions [26] resulted in the selective formation of the deacylated product **3a** (Table 1, entry 13).

Thus, the optimized conditions for the synthesis of 2-hydroxy-substituted 2,5-dihydrothiophene **2a** were found to be the use of 5.0 equiv of sodium in an oxygen saturated ethanolic solution at rt for 1 h. The deacylated product **3a** was synthesized in high yield when the reaction was performed with 5.0 equiv of sodium in methanolic solution at rt for 1 h in contact with air.

**Scheme 2:**
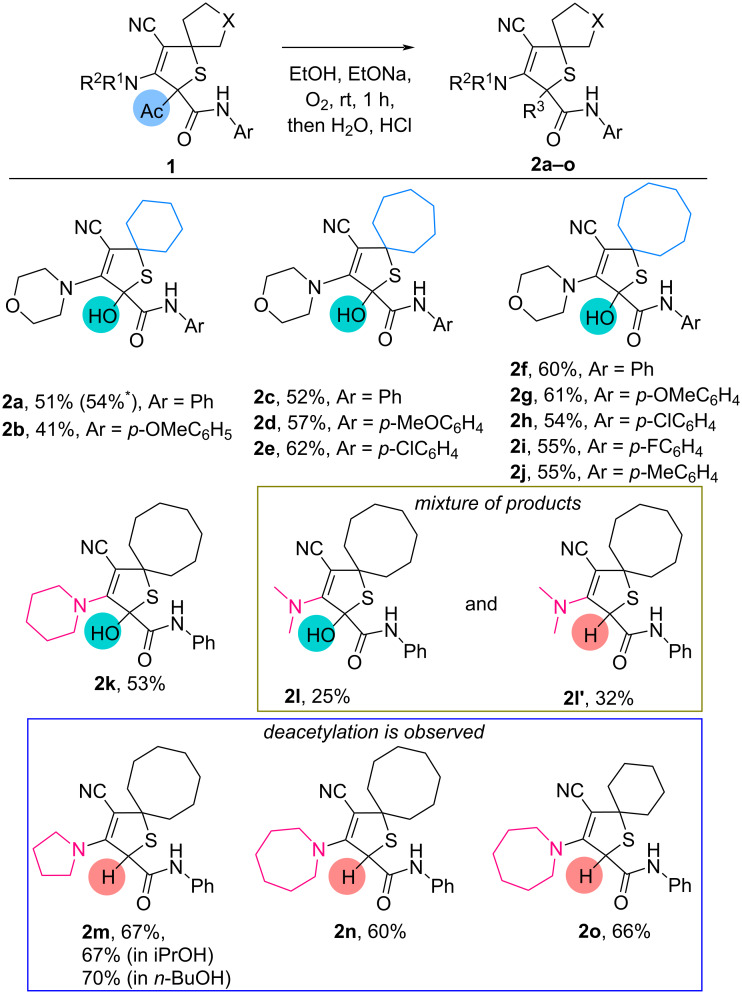
Oxidation of 2-acetyldihydrothiophenes **1**. Conditions: dihydrothiophenes **1** (0.12–0.21 mmol, 1.0 equiv), sodium (0.60–1.05 mmol, 5 equiv), and dry EtOH (2.0–3.0 mL). ^*^Scaled-up synthesis: dihydrothiophene **1a** (1.17 mmol, 1.0 equiv), sodium (5.87 mmol, 5 equiv), in dry EtOH (5 mL).

With these optimal conditions in hand, we have investigated the oxidation of 2-acetyl-2,5-dihydrothiophenes **1**, containing various substituents (Scheme 2).

Cyclohexano-, cycloheptano- and cyclooctano-spiroannulated 2-acetyl-3-morpholino-*N*-phenyl-2,5-dihydrothiophene-2-carboxamides **1a,c,f** were oxidized into 2-hydroxy derivatives **2a,c,f** in 51–60% yields. Various *N*-aryl-substituted 2,5-dihydrothiophene-2-carboxamides **1b,d,e,g–j** selectively transformed into oxidized products **2b,d,e,g**–**j** in 41–62% yields. Variation of the amine moiety in the cyclooctano-spiroannulated 2-acetyl-*N*-phenyl-2,5-dihydrothiophene-2-carboxamides showed that the oxidized product formed from morpholine- (**1f**) and piperidine-substituted (**1k**) 2,5-dihydrothiophenes in 60 (**2f**) and 53% (**2k**) yield. Oxidation of the dimethylamino-substituted 2,5-dihydrothiophene **1l** afforded a mixture of products in 25% (OH-substituted, **2l**) and 32% (H-substituted, **2l′**) yield. Pyrrolidine- and azepane-substituted cyclooctano-spiroannulated 2,5-dihydrothiophenes **1m** and **1n** were found to be transformed into deacetylated products in 67% (**2m**) and 60% (**2n**) yields. When experiments were performed in iPrOH or *n*-BuOH, we observed the formation of the deacylated product (**2n**) in 67 and 70% yield, accordingly.

**Scheme 3:**
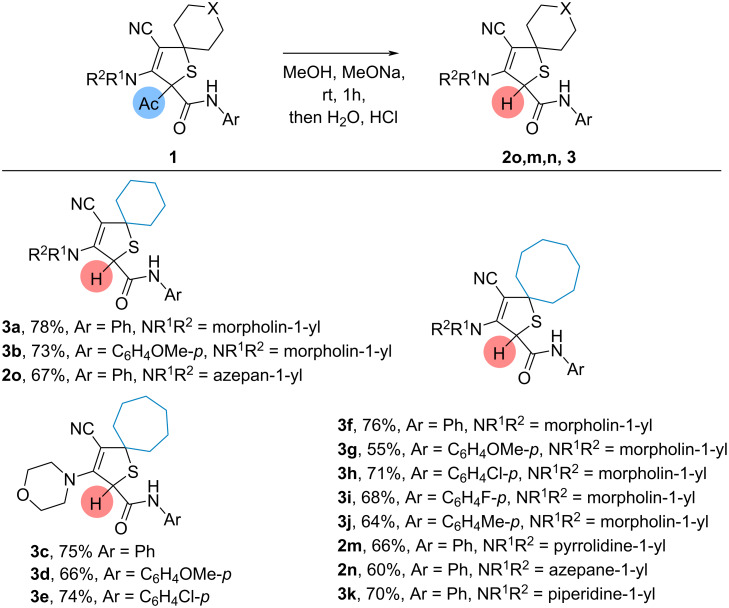
Deacylation of 2-acetyldihydrothiophenes **1**. Conditions: dihydrothiophenes **1** (0.11–0.18 mmol, 1.0 equiv), sodium (0.55–0.88 mmol, 5 equiv), dry MeOH (2.0–3.0 mL).

Next, the deacylation of 2-acetyl-2,5-dihydrothiophenes in methanolic solution was investigated (Scheme 3). Cyclohexano-, cycloheptano- and cyclooctano-spiroannulated 2-acetyl-3-morpholino-*N*-phenyl-2,5-dihydrothiophene-2-carboxamides in these conditions gave deacylated products in 75–78% yield. Different *N*-aryl substituted 2,5-dihydrothiophene-2-carboxamides selectively formed the deacylated products in 55–74% yield. Variation of the amine moiety in the cyclooctano- and cyclohexano-spiroannulated 2-acetyl-*N*-phenyl-2,5-dihydrothiophene-2-carboxamides resulted in all cases in the selective formation of the deacylated products in 60–70% yield.

In continuation of the research, isomeric dihydrothiophenes (Scheme 1, E) were also treated in ethanolic solution with sodium ethoxide. Initially, cyclohexano-spiroannulated dihydrothiophene **4a** was treated with ethanolic solution (3 mL) in the presence of sodium ethoxide obtained from 2 equiv of sodium at rt in air for 1 h. After the reaction was completed, the deacylated product was isolated in 69% yield (Table 2, entry 1). When the loading of sodium was increased up to 5.0 equiv, the yield of deacylated product was slightly increased to 72% (Table 2, entry 2). In a more concentrated ethanol solution (1 mL) the product was obtained in 70% yield (Table 2, entry 3). When the residue was quenched with concentrated HCl (1 mL), the product was isolated in reduced yield (58%, Table 2, entry 4). Adding 0.25 mL of acid (HCl) after quenching the residue with water resulted in an increase in the product yield (74%, Table 2, entry 5). Increasing the amount of sodium up to 10 equiv resulted in a decrease of the yield of the product **5a** (61%, Table 2, entry 6).

**Table 2:** Optimization of the transformation of dihydrothiophene **4a**.^a^

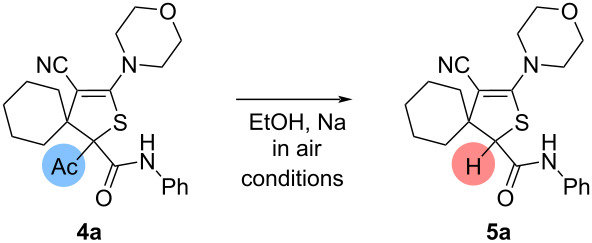					

Entry	Na (equiv)	Solvent (mL)	Water (mL)	HCl (mL)	Yield, (%)^b^

1	Na (2)	EtOH (3)	2	‒	69
2	Na (5)	EtOH (3)	2	‒	72
3	Na (5)	EtOH (1)	2	‒	70
4	Na (5)	EtOH (3)	‒	1	58
5	Na (5)	EtOH (3)	2	0.25	74
6	Na (10)	EtOH (3)	2	0.25	61

^a^Conditions: dihydrothiophene **4a** (0.19 mmol), rt, 1 h. Water or/and HCl were added after evaporation of the solvent. ^b^Isolated yields after centrifugation in Et_2_O/hexane (1:2).

Thus, the optimized conditions for the synthesis of dihydrothiophene **5a** were found to be the use of 5.0 equiv of sodium in ethanolic solution (3 mL) at rt for 1 h in air (Table 2, entry 5).

**Scheme 4:**
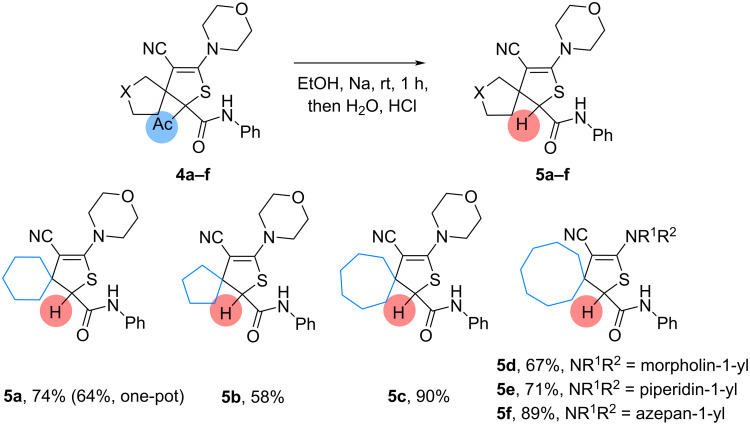
Synthesis of dihydrothiophenes **5**. Conditions: dihydrothiophenes **4** (0.13–0.22 mmol, 1.0 equiv), sodium (0.66–1.11 mmol, 5 equiv), dry EtOH (2.5–3.0 mL).

With optimal conditions in hand, we have investigated the deacylation of acetyldihydrothiophenes **4a**–**f**, containing various substituents (Scheme 4).

Thus, cyclohexano-, cyclopentano-, cycloheptano- and cyclooctano-spiroannulated dihydrothiophenes **4a**–**f** were transformed into products **5a**–**f** in 58–90% yield. Piperidine- and azepane-substituted cyclooctano-spiroannulated dihydrothiophenes **4e,f** also transformed into deacylated products **5e,f** in 71% and 89% yield, respectively.

**Scheme 5:**
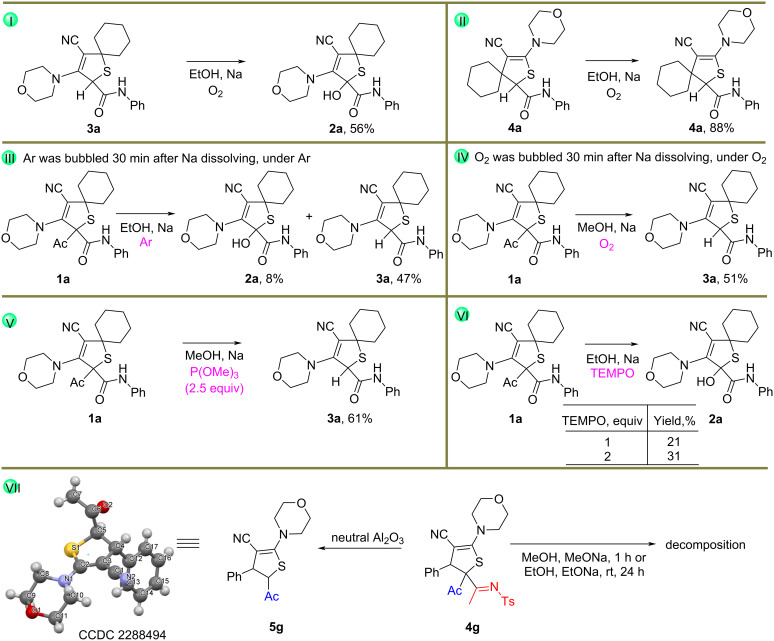
Control experiments.

Several control experiments were performed to find the effect of oxygen, argon, additives and TEMPO on the outcome of the oxidation and deacylation reactions (Scheme 5).

When the deacylated dihydrothiophene **3a**, obtained in methanolic solution, was treated with sodium ethoxide in an oxygen-saturated ethanolic solution, 2-hydroxysubstituted product **2a** was isolated in 56% yield (Scheme 5, I). On the other hand, isomeric deacylated dihydrothiophene **4a** in these conditions did not transform into the oxidized product, and deacylated product **4a** was recovered in 88% yield (Scheme 5, II).

Next, the transformation of **1a** in an argon saturated ethanolic solution results in the formation of 2-hydroxy-substituted product **2a** in low yield (8%), although the yield of the deacylated product **3a** increased up to 47% (Scheme 5, III). This suggests the participation of oxygen in the formation of 2-hydroxy-substituted product **2a**. When the methanolic solution was saturated with oxygen, the 2-hydroxy-substituted product **2a** was not isolated. Only the formation of the deacylated product **3a** in 51% yield was observed (Scheme 5, IV).

The influence of a reductant (trimethyl phosphite) on the reaction in methanol was evaluated (Scheme 5, V). This did not have a significant effect on the reaction outcome. Thus, there was no formation of oxidized intermediates during transformation.

The effect of TEMPO (up to 2.0 equiv) was evaluated on the oxidation reaction, and TEMPO was found to not inhibit the formation of 2-hydroxy-substituted product **2a** (Scheme 5, VI). Therefore, the reaction is most likely not proceeding via a free radical mechanism.

To clarify the influence of the amide group on the developed transformations, dihydrothiophene **4g** bearing a sulfonylimine group instead of an amide was treated with sodium methoxide in methanol or with sodium ethoxide in ethanol (Scheme 5, VII). However, under these conditions only decomposition of **4g** was observed, and neither deacetylated nor hydroxylated products were isolated. Interestingly, chromatography of **4g** on neutral alumina resulted in elimination of the sulfonylimine group to give compounds **5g**. Therefore, the amide group plays an important role in these transformations.

**Figure 1:**
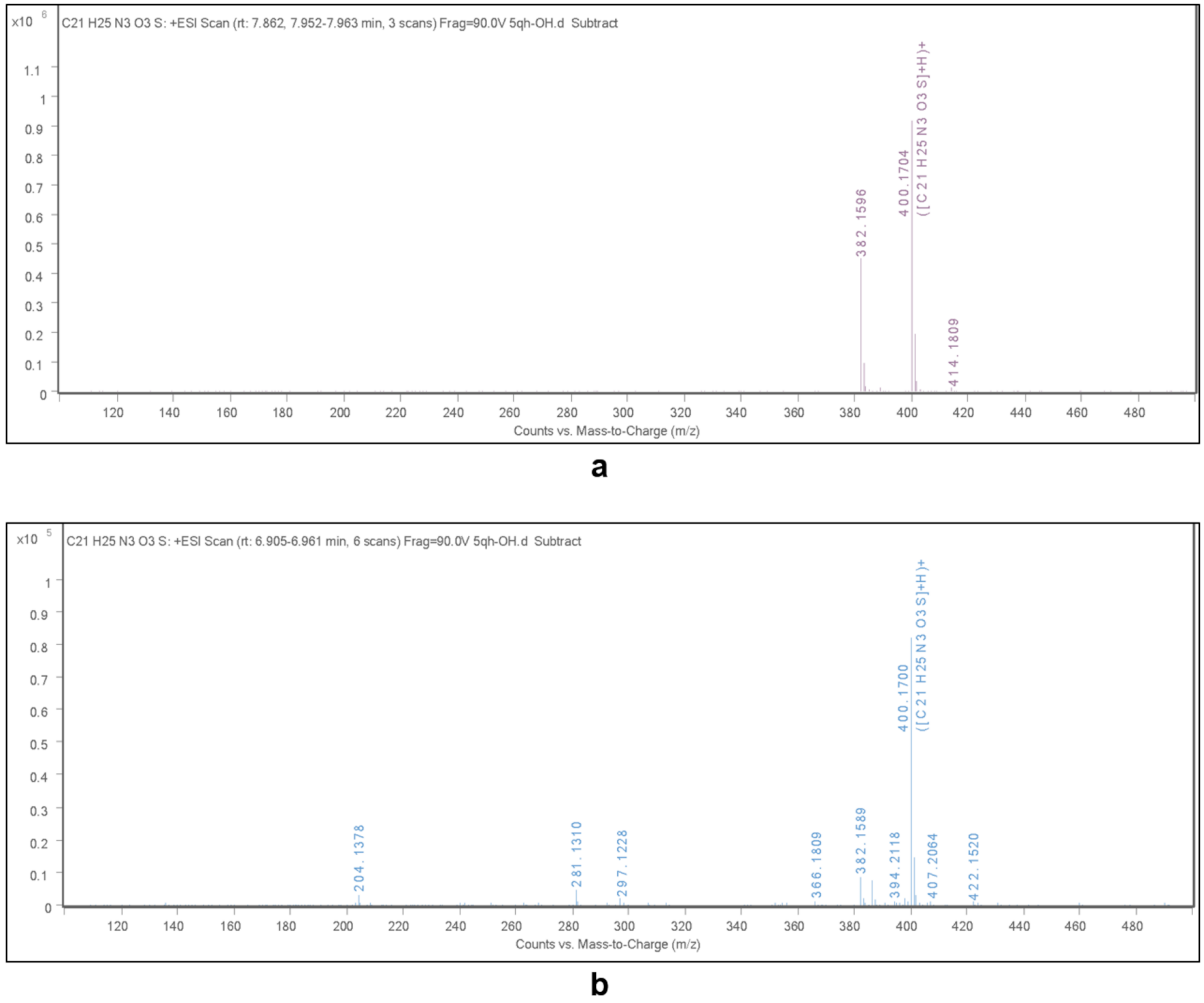
HRMS analysis of the crude product.

**Scheme 6:**
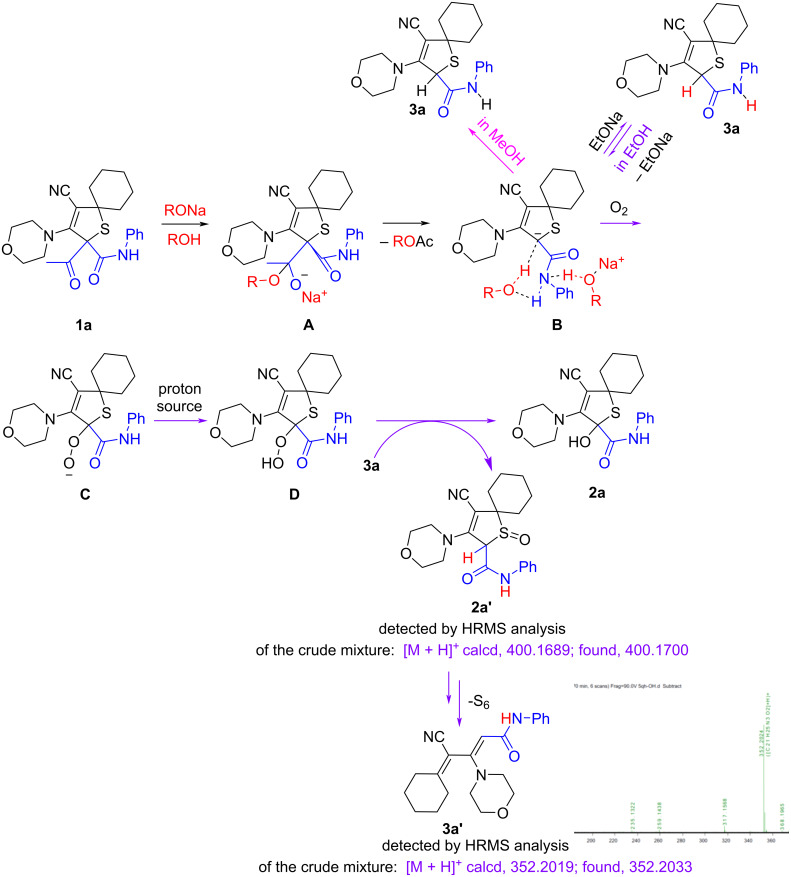
Proposed mechanism.

In addition, analysis of the reaction mixture obtained in ethanolic solution was performed after evaporation of the ethanol. HRMS analysis showed the presence of two peaks with *m*/*z* values 400.1704 (retention time 7.952‒7.963) and 400.1700 (retention time 6.905‒6.961). One of these peaks can be assigned to the product **2a**, while the other peak showed that the deacylated product **3a** is formed during the transformation with subsequent oxidation by sulfur in the oxidation/reduction step to form 2,5-dihydrothiophene 1-oxide **2a′** (Figure 1, a and b and Scheme 6).

The analysis also suggests the formation of product **2a** before water and acid were added. This also indicates that the reaction proceeded through an oxidation/reduction step.

**Figure 2:**
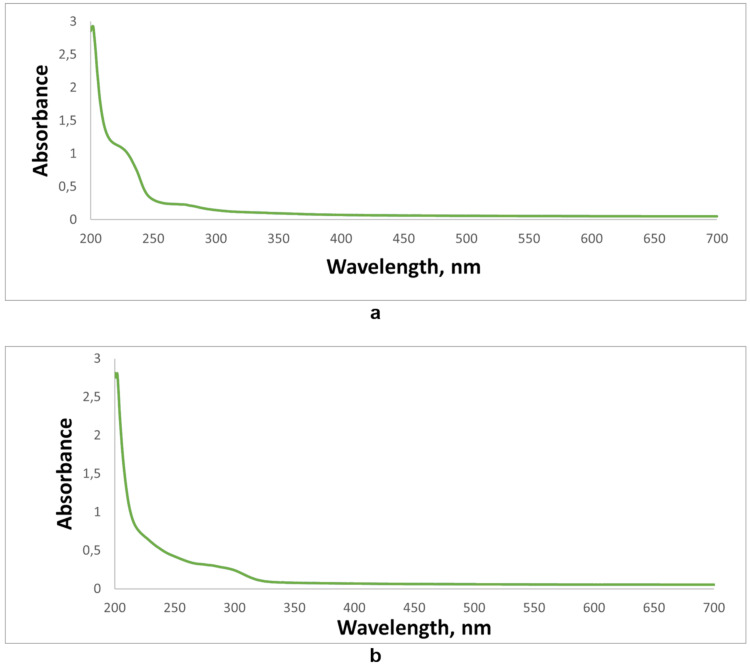
UV–vis spectra of the crude mixture (5.6 mg of the crude mixture was dissolved in 15 mL of methanol and the aliquot (100 µL) was diluted in 900 µL of methanol) (a) and purified product **2a** (*с* = 5 × 10^−5^ M) (b) in methanol at 20 °C.

UV absorption measurements of the same reaction mixture (a) and pure product **2a** (b) dissolved in methanol are presented in Figure 2.

The formation of product **2a** before water and acid were added also follows from the comparison of UV–vis spectra of crude mixture (a) and product **2a** (b). According to the previously reported data [51], the absorption maximum in spectrum (a) at 220 nm is caused by the presence of elemental sulfur S_6_. Probably, the reaction is accompanied by the desulfurization of the oxidized intermediate, which causes the yellow color of the reaction solutions.

The proposed mechanism for the developed transformations is depicted in Scheme 6. The reaction of dihydrothiophene **1a** with sodium ethoxide led to the intermediate **A**. Elimination of ethyl/methyl acetate from intermediate **A** afforded anion **B**. The latter reacted in ethanolic solution with molecular oxygen [52] with the formation of peroxide anion **C**. The protonation of anion **C** with proton sources (residual water or/and solvent or **3a** can serve as a proton source) formed hydroperoxide **D**. On the other hand, competitive reversible proton movement [53] from ethanol to anion **B** formed deacetylated product **3a**. The subsequent reduction of hydroperoxide **D** by the deacetylated product **3a** results in the formation of hydroxy-substituted dihydrothiophene **2a** and oxidized product **2a′**. The latter, probably, undergoes desulfurization into compound **3a′** (for example, base-promoted transformation of 2,5-dihydrothiophenes-1,1-dioxides to 1,3-dienes was reported by S. Zard [54]).

It can be assumed that the higher acidity of methanol in comparison with ethanol makes the proton transfer to anion **B** quasi-irreversible. This could be the cause for the selective formation of deacetylated dihydrothiophene **3a** in methanolic solution.

The observed deacetylation for product **2m** in ethanol on Scheme 2 can be caused by a decrease of the stability of the formed anion due to the stronger donor character of the pyrrolidine moiety due to its planar structure. The formation of the deacetylated products **2n**,**o** (Scheme 2) may be attributed to increased steric hindrance, which makes proton transfer to **B** in Scheme 6 more difficult. The dimethyl-1-yl moiety likely exhibits a lower donor character compared to pyrrolidine, but higher than that of morpholine and piperidine. As a result, a mixture of products **2l** and **2l′** is formed.

Starting dihydrothiophenes **1** form more stable anions **B** in comparison with regioisomers **4** due to the delocalization of the negative charge over the double bond, sulfur and amide group. The difference in the stability of these types of anions results in their distinct reactivity.

## Conclusion

We have reported the solvent dependent transformation of dihydrothiophenes **1** under mild conditions. It was found that, in ethanolic solution in the presence of sodium ethoxide and molecular oxygen at ambient pressure, dihydrothiophenes **1a**,**b**,**d**–**l** were oxidized into hydroxyderivatives **2a**,**b**,**d**–**l**. In methanolic solution, in the presence of sodium methoxide and molecular oxygen, the same dihydrothiophenes **1a**,**b**,**d**–**l** transformed into deacetylated products **3**. Isomeric dihydrothiophenes **4a**–**f** formed only deacetylated products **5a**–**f** when the reaction was performed in an oxygen saturated ethanolic solution in the presence of sodium ethoxide.

## Experimental

### X-ray structure determination of **5g**

**5g**: Crystal data for C_17_H_18_N_2_O_2_S (*M* = 314.40 g/mol): monoclinic, space group *P−*1, *a* = 9.3076 (5) Å, *b* = 9.3243(5) Å, *c* = 10.1072 (4) Å, β = 102.480(4)°, *V* = 776.19(7) Å^3^, *Z* = 2, μ(Mo Kα) = 0.217 mm^−1^, *D*_calc_ = 1.345 g/cm^3^, 4252 reflections measured (7.378° ≤ 2Θ ≤ 61°), 4252 unique (*R*_int_ = 0.0407, *R*_sigma_ = 0.0545) which were used in all calculations. The final *R*_1_ = 0.0596, w*R*_2_ = 0.1470 (*I* >= 2σ(I)) and *R*_1_ = 0.0837, w*R*_2_ = 0.1768 (all data). Largest diff. peak/hole 0.29/−0.55 e^−^Å^−3^.

## Supporting Information

File 1 Full experimental details and characterization data of all new compounds.

File 2 Copies of NMR spectra of all new compounds.

File 3 CIF-file for compound **5g**.

File 4 CheckCIF-file for compound **5g**.

## Data Availability

All data that supports the findings of this study is available in the published article and/or the supporting information of this article.
