# Supplementary material for: Base-promoted deacylation of 2-acetyl-2,5-dihydrothiophenes and their oxygen-mediated hydroxylation
Source: Beilstein J Org Chem. 2026 Jan 28;22:192–204. doi: 10.3762/bjoc.22.13 (PMC12862606; doi:10.3762/bjoc.22.13)
Supplement: File 1 — Full experimental details and characterization data of all new compounds. [file Beilstein_J_Org_Chem-22-192-s001.pdf]

BEILSTEIN JOURNAL OF ORGANIC CHEMISTRY

Supporting Information

for

Base-promoted deacylation of 2-acetyl-2,5-dihydrothiophenes and their oxygen-mediated hydroxylation

Vladimir G. Ilkin, Margarita Likhacheva, Igor V. Trushkov, Tetyana V. Beryozkina, Vera S. Berseneva, Vladimir T. Abaev, Wim Dehaen and Vasiliy A. Bakulev

Beilstein J. Org. Chem. **2026,** *22,* 192–204. doi:10.3762/bjoc.22.13

Full experimental details and characterization data of all new compounds

License and Terms: This is a supporting information file under the terms of the Creative Commons Attribution License (https://creativecommons.org/licenses/by/4.0). Please note that the reuse, redistribution and reproduction in particular requires that the author(s) and source are credited and that individual graphics may be subject to special legal provisions.
The license is subject to the *Beilstein Journal of Organic Chemistry* terms and conditions: (https://www.beilstein-journals.org/bjoc/terms)

Table of contents

Experimental S3

Dihydrothiophenes were used in the research S3

Preparation of starting reagent S4

Optimization study S9

General procedure for derivatization of dihydrothiophenes **1** in ethanolic solution S10

General procedure for deacetylation of dihydrothiophenes **1** in methanolic solution S15

General procedure for derivatization of dihydrothiophenes **4a**–**f** in ethanolic solution.

Method A S19

Method B (one-pot) S19

The procedure for the derivatization of dihydrothiophene **4g** S21

Additional mechanistic studies S22

References S24

Experimental

General. All chemicals were obtained from commercial sources and were used without further purification. Dry solvents (chloroform and alcohols) were obtained according to the literature protocols and stored over molecular sieves. Analytical thin-layer chromatography was performed on aluminum foil plates coated with 0.2 mm of silica gel. Column chromatography was performed by using 230−400 mesh silica gel. Melting points were determined on a Stuart SMP10 melting point apparatus and were uncorrected. All NMR spectra were recorded at 400 or 600 (^{1}H NMR) and 100 or 150 MHz (^{13}C NMR) in $CDCl_3$ or DMSO-d_6. The chemical shifts are given in parts per million (ppm) relative to the resonance of the solvents [^{1}H: δ ($CDCl_3$) = 7.26, δ (DMSO-d_6) = 2.50, ^{13}C: δ ($CDCl_3$) = 77.2, δ (DMSO-d_6) = 39.5 ppm]. Multiplicities were given as s (singlet), d (doublet), t (triplet) and m (multiplet). Coupling constants are reported as the *J* value in Hertz (Hz). High-resolution mass spectra (HRMS) were recorded using an ultrahigh-resolution quadrupole time-of-flight mass spectrometer with an electrospray ionization probe installed coupled with an Agilent 1260 HPLC system.

Dihydrothiophenes were used in the research

Dihydrothiophenes were obtained in the Cu(I)-catalyzed reaction

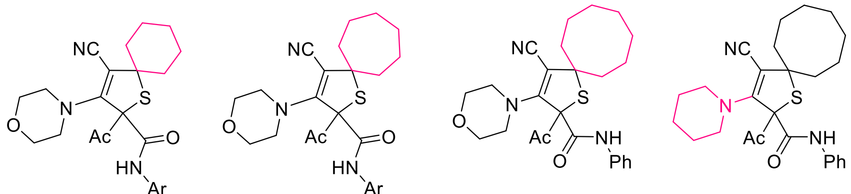

1a, Ar = Ph
1b, Ar = C_6H_5OMe-*p*

1c, Ar = Ph
1d, Ar = C_6H_4OMe-*p*
1e, Ar = C_6H_4Cl-*p*

1f, Ar = Ph
1g, Ar = C_6H_4OMe-*p*
1h, Ar = C_6H_4Cl-*p*
1i, Ar = C_6H_4F-*p*
1j, Ar = C_6H_4Me-*p*

1k

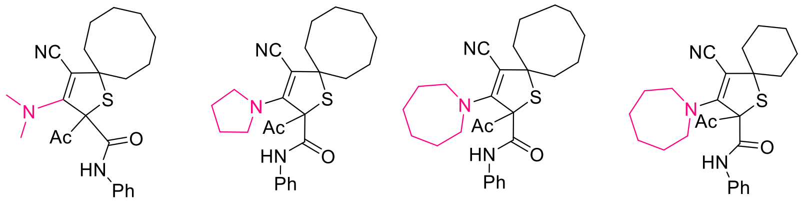

1l **1m** **1n** **1o**

Dihydrothiophenes were obtained in the Rh(II)-catalyzed reaction

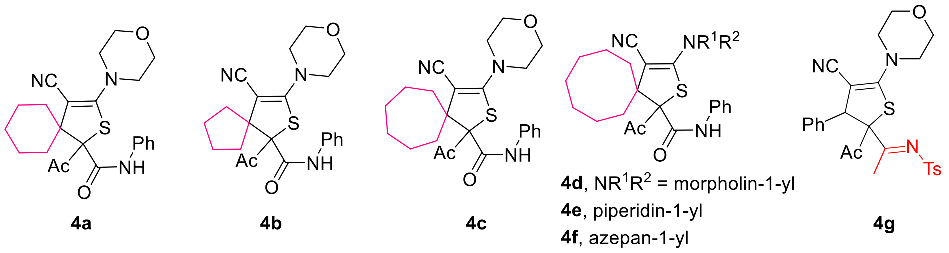

4a **4b** **4c**

4d, NR^1R^2 = morpholin-1-yl
4e, piperidin-1-yl
4f, azepan-1-yl

4g

Preparation of starting reagent

Dihydrothiophenes **1** and **4** were synthesized according to the previous reported procedure [1]. Dihydrothiophenes **1a,c,f,4a–g** were characterized previously [1]. Diazo compounds were synthesized according to the previous reported procedure [2] (2-diazo-3-oxo-*N*-phenylbutanamide, 2-diazo-*N*-(4-fluorophenyl)-3-oxobutanamide, 2-diazo-3-oxo-*N*-(*p*-tolyl)butanamide) or with the use of the another procedure [3] (2-diazo-*N*-(4-methoxyphenyl)-3-oxobutanamide, *N*-(4-chlorophenyl)-2-diazo-3-oxobutanamide).

*2-Acetyl-4-cyano-N-(4-methoxyphenyl)-3-morpholino-1-thiaspiro[4.5]dec-3-ene-2-carboxamide (**1b**).* Product **1b** was obtained from 2-cyclohexylidene-3-morpholino-3-thioxopropanenitrile (200 mg, 1.0 equiv, 0.80 mmol), 2-diazo-*N*-(4-methoxyphenyl)-3-oxobutanamide (373 mg, 2.0 equiv, 1.60 mmol), [Cu(MeCN)$_4$]OTf (60 mg, 10 mol %), chloroform (6.0 mL), 90 °C, reaction time is 24 h. The purification of the crude product by column chromatography on SiO_2 (DCM/EtOAc, gradient 25:0 to 23.5:1.5) afforded **1b** as a yellow oily mass. The latter was triturated and centrifugated at first with hexane/diethyl ether (1:1) and then with hexane (2 mL), affording **1b** as a beige powder (53%, 195 mg), mp 184–186 °C. ^{1}H NMR (400 MHz, $CDCl_3$-*d*): δ 9.20 (s, 1H), 7.44 (d, J = 9.0 Hz, 2H), 6.89 (d, J = 9.0 Hz, 2H), 3.80 (s, 3H), 3.69 – 3.61 (m, 4H), 3.57 – 3.50 (m, 2H), 3.37 – 3.31 (m, 2H), 2.47 (s, 3H), 2.04 – 1.94 (m, 3H), 1.87 – 1.78 (m, 3H), 1.68 – 1.61 (m, 1H), 1.47 – 1.13 (m, 3H). ^{13}C{1H} NMR (100 MHz, $CDCl_3$-*d*): δ 201.7, 165.7, 157.1, 156.0, 130.3, 121.9, 117.7, 114.5, 93.8, 71.1, 66.5, 63.6, 55.7, 50.4, 40.1, 38.9, 26.6, 24.5, 24.4, 23.8. HRMS (ESI) *m/z*: $[M + H]^+$ Calcd for $C_{24}H_{30}N_3O_4S^+$ 456.1951; Found 456.1958.

*2-Acetyl-4-cyano-N-(4-methoxyphenyl)-3-morpholino-1-thiaspiro[4.6]undec-3-ene-2-carboxamide (**1d**).* Product **1d** was obtained from 2-cycloheptylidene-3-morpholino-3-thioxopropanenitrile (200 mg, 1.0 equiv, 0.76 mmol), 2-diazo-*N*-(4-methoxyphenyl)-3-oxobutanamide (353 mg, 2.0 equiv, 1.52 mmol), [Cu(MeCN)$_4$]OTf (57 mg, 10 mol %), chloroform (6.0 mL), 90 °C, reaction time is 24 h. The purification of the crude product by column chromatography on SiO_2 (DCM/EtOAc, gradient 25:0 to 23.5:1.5) afforded **1d** as a yellow oily mass. The latter was triturated and centrifugated at first with hexane/diethyl ether (1:1) and then with hexane (2 mL), affording **1d** as a beige powder (52%, 184 mg), mp 148–150 °C. ^{1}H NMR (400 MHz, $CDCl_3$-*d*): δ 9.15 (s, 1H), 7.43 (d, J = 9.0 Hz, 2H), 6.89 (d, J = 9.0 Hz, 2H), 3.80 (s, 3H), 3.69 – 3.59 (m, 4H), 3.56 – 3.50 (m, 2H), 3.34 – 3.29 (m, 2H), 2.47 (s, 3H), 2.28 – 2.16 (m, 3H), 2.04 – 1.98 (m, 1H), 1.81 – 1.77 (m, 2H), 1.62 – 1.50 (m, 6H). ^{13}C{1H} NMR (100 MHz, $CDCl_3$-*d*): δ 201.6, 165.6, 157.1, 155.6,

130.3, 121.9, 117.9, 114.5, 95.3, 71.6, 66.6, 65.8, 55.7, 50.4, 44.2, 42.6, 27.7, 26.7, 24.6, 24.0. HRMS (ESI) *m/z*: $[M + H]^+$ Calcd for $C_{25}H_{32}N_3O_4S^+$ 470.2111; Found 470.2115.

*2-Acetyl-N-(4-chlorophenyl)-4-cyano-3-morpholino-1-thiaspiro[4.6]undec-3-ene-2-carboxamide (**1e**).* Product **1e** was obtained from 2-cycloheptylidene-3-morpholino-3-thioxopropanenitrile (200 mg, 1.0 equiv, 0.76 mmol), *N*-(4-chlorophenyl)-2-diazo-3-oxobutanamide (359 mg, 2.0 equiv, 1.52 mmol), $[Cu(MeCN)_4]OTf$ (57 mg, 10 mol %), chloroform (6.0 mL), 90 °C, reaction time is 24 h. The purification of the crude product by column chromatography on SiO_2 (DCM/EtOAc, gradient 25:0 to 25:1.0) afforded **1e** as a yellow oily mass. The latter was triturated and centrifugated with hexane/diethyl ether (1:1) and then with hexane (2 mL) affording **1e** as a beige powder (56%, 200 mg), mp 180–182 °C. ^1H NMR (400 MHz, $CDCl_3$-*d*): δ 9.41 (s, 1H), 7.49 (d, *J* = 8.8 Hz, 2H), 7.32 (d, *J* = 8.8 Hz, 2H), 3.69 – 3.60 (m, 4H), 3.52 – 3.47 (m, 2H), 3.34 – 3.28 (m, 2H), 2.48 (s, 3H), 2.26 – 2.19 (m, 3H), 2.06 – 2.00 (m, 1H), 1.83 – 1.76 (m, 2H), 1.64 – 1.48 (m, 6H). $^{13}C\{1H\}$ NMR (100 MHz, $CDCl_3$-*d*): δ 201.8, 166.0, 155.0, 135.8, 130.3, 129.3, 121.5, 117.8, 95.4, 71.0, 66.5, 66.3, 50.2, 44.2, 42.7, 27.7, 27.7, 26.5, 24.5, 24.1. HRMS (ESI) *m/z*: $[M + H]^+$ Calcd for $C_{24}H_{29}ClN_3O_3S^+$ 474.1613; Found 474.1621.

*2-Acetyl-4-cyano-N-(4-methoxyphenyl)-3-morpholino-1-thiaspiro[4.7]dodec-3-ene-2-carboxamide (**1g**).* Product **1g** was obtained from 2-cyclooctylidene-3-morpholino-3-thioxopropanenitrile (300 mg, 1.0 equiv, 1.08 mmol), 2-diazo-*N*-(4-methoxyphenyl)-3-oxobutanamide (503 mg, 2.0 equiv, 2.16 mmol), $[Cu(MeCN)_4]OTf$ (81 mg, 10 mol %), chloroform (7.0 mL), 90 °C, reaction time is 24 h. The purification of the crude product by column chromatography on SiO_2 (DCM/EtOAc, gradient 25:0 to 25:1.2) afforded **1g** as a yellow oily mass. The latter was triturated with hexane/diethyl ether (3:1) until dissolving. After the evaporation of solvents in the hood, the precipitate was formed. It was triturated with hexane (3 mL) and centrifugated, then the procedure was repeated in hexane/diethyl ether (3:0.5) and (3:1) affording **1g** as a colorless powder (58%, 304 mg), mp 155–157 °C. ^1H NMR (400 MHz, $CDCl_3$-*d*): δ 9.16 (s, 1H), 7.43 (d, *J* = 9.0 Hz, 2H), 6.89 (d, *J* = 9.1 Hz, 2H), 3.80 (s, 3H), 3.69 – 3.60 (m, 4H), 3.57 – 3.51 (m, 2H), 3.35 – 3.30 (m, 2H), 2.46 (s, 3H), 2.41 – 2.29 (m, 2H), 2.24 – 2.18 (m, 1H), 2.07 – 2.00 (m, 1H), 1.90 – 1.84 (m, 2H), 1.63 – 1.49 (m, 8H). $^{13}C\{1H\}$ NMR (100 MHz, $CDCl_3$-*d*): δ 201.5, 165.6, 157.1, 156.2, 130.3, 121.9, 118.2, 114.5, 94.5, 71.4, 66.6, 65.6, 55.7, 50.5, 40.8, 38.7, 27.9, 27.8, 26.6, 24.5, 24.1, 23.9. HRMS (ESI) *m/z*: $[M + H]^+$ Calcd for $C_{26}H_{34}N_3O_4S^+$ 484.2264; Found 484.2270.

*2-Acetyl-N-(4-chlorophenyl)-4-cyano-3-morpholino-1-thiaspiro[4.7]dodec-3-ene-2-carboxamide (**1h**).* Product **1h** was obtained from 2-cyclooctylidene-3-morpholino-3-thioxopropanenitrile (200 mg, 1.0 equiv, 0.72 mmol), *N*-(4-chlorophenyl)-2-diazo-3-oxobutanamide (342 mg, 2.0 equiv, 1.44 mmol), [Cu(MeCN)$_4$]OTf (47 mg, 10 mol %), chloroform (6.0 mL), 90 °C, reaction time is 24 h. The purification of the crude product by column chromatography on SiO_2 (DCM/EtOAc, gradient 25:0 to 25:1.0) afforded **1h** as a yellow oily mass. The latter was triturated with hexane/diethyl ether (1:1) and centrifugated, affording **1h** as a beige powder (44%, 156 mg), mp 177–179 °C. ^{1}H NMR (400 MHz, $CDCl_3$-*d*): δ 9.42 (s, 1H), 7.49 (d, J = 8.8 Hz, 2H), 7.32 (d, J = 8.8 Hz, 2H), 3.69 – 3.60 (m, 4H), 3.53 – 3.46 (m, 2H), 3.35 – 3.29 (m, 2H), 2.47 (s, 3H), 2.39 – 2.32 (m, 2H), 2.25 – 2.19 (m, 1H), 2.09 – 2.02 (m, 1H), 1.90 – 1.83 (m, 2H), 1.65 – 1.50 (m, 8H). ^{13}C{1H} NMR (100 MHz, $CDCl_3$-*d*): δ 201.7, 166.0, 155.7, 135.8, 130.3, 129.3, 121.5, 118.1, 94.6, 70.9, 66.5, 66.1, 50.4, 40.8, 38.9, 27.9, 27.7, 26.5, 24.4, 24.1, 24.0. HRMS (ESI) *m/z*: [M + H]$^+$ Calcd for $C_{25}H_{31}ClN_3O_3S^+$ 488.1769; Found 488.1774.

*2-Acetyl-4-cyano-N-(4-fluorophenyl)-3-morpholino-1-thiaspiro[4.7]dodec-3-ene-2-carboxamide (**1i**).* Product **1i** was obtained from 2-cyclooctylidene-3-morpholino-3-thioxopropanenitrile (200 mg, 1.0 equiv, 0.72 mmol), 2-diazo-*N*-(4-fluorophenyl)-3-oxobutanamide (318 mg, 2.0 equiv, 1.44 mmol), [Cu(MeCN)$_4$]OTf (54 mg, 10 mol %), chloroform (6.0 mL), 90 °C, reaction time is 24 h. The purification of the crude product by column chromatography on SiO_2 (DCM/EtOAc, gradient 25:0 to 23.4:1.6), affording **1i** as a yellow oily mass. The latter was triturated with hexane/diethyl ether (2:1) and centrifugated afforded **1i** as a beige powder (64%, 218 mg), mp 178–180 °C. ^{1}H NMR (400 MHz, $CDCl_3$-*d*): δ 9.35 (s, 1H), 7.51 – 7.48 (m, 2H), 7.07 – 7.03 (m, 2H), 3.70 – 3.61 (m, 4H), 3.54 – 3.49 (m, 2H), 3.35 – 3.30 (m, 2H), 2.47 (s, 3H), 2.40 – 2.32 (m, 2H), 2.25 – 2.19 (m, 1H), 2.08 – 2.02 (m, 1H), 1.91 – 1.84 (m, 2H), 1.62 – 1.50 (m, 8H). ^{13}C{1H} NMR (100 MHz, $CDCl_3$-*d*): δ 201.7, 165.9, 159.9 (d, J = 244.9 Hz), 155.8, 133.3 (d, J = 2.9 Hz), 122.0 (d, J = 8.0 Hz), 118.1, 116.0 (d, J = 22.6 Hz), 94.6, 71.0, 66.5, 66.0, 50.4, 40.8, 38.9, 27.9, 27.8, 26.5, 24.4, 24.1, 24.0. HRMS (ESI) *m/z*: [M + H]$^+$ Calcd for $C_{25}H_{31}FN_3O_3S^+$ 472.2065; Found 472.2072.

*2-Acetyl-4-cyano-3-morpholino-N-(p-tolyl)-1-thiaspiro[4.7]dodec-3-ene-2-carboxamide (**1j**).* Product **1j** was obtained from 2-cyclooctylidene-3-morpholino-3-thioxopropanenitrile (180 mg, 1.0 equiv, 0.65 mmol), 2-diazo-3-oxo-*N*-(*p*-tolyl)butanamide (282 mg, 2.0 equiv, 1.30 mmol), [Cu(MeCN)$_4$]OTf (48 mg, 10 mol %), chloroform (6.0 mL), 90 °C, reaction time is 24 h. The purification of the crude product by column chromatography on SiO_2

(DCM/EtOAc, gradient 25:0 to 24.6:0.4), affording **1j** as a yellow oily mass. The latter was triturated with hexane/diethyl ether (1:1) until dissolution. After the evaporation of solvents in the hood, a precipitate was formed. This was triturated with hexane and centrifugated (3×2 mL), affording **1j** as a colorless powder (59%, 179 mg), mp 185–187 °C. ^{1}H NMR (400 MHz, $CDCl_3$-*d*): *δ* 9.19 (s, 1H), 7.40 (d, *J* = 8.4 Hz, 2H), 7.16 (d, *J* = 8.2 Hz, 2H), 3.69 – 3.59 (m, 4H), 3.56 – 3.51 (m, 2H), 3.35 – 3.29 (m, 2H), 2.46 (s, 3H), 2.40 – 2.30 (m, 2H+3H), 2.25 – 2.18 (m, 1H), 2.06 – 2.00 (m, 1H), 1.92 – 1.84 (m, 2H), 1.63 – 1.51 (m, 8H). ^{13}C{1H} NMR (100 MHz, $CDCl_3$-*d*): *δ* 201.5, 165.7, 156.2, 135.0, 134.6, 129.8, 120.2, 118.2, 94.5, 71.5, 66.6, 65.6, 50.5, 40.8, 38.7, 27.9, 27.8, 26.6, 24.5, 24.1, 23.9, 21.0. HRMS (ESI) *m/z*: $[M + H]^+$ Calcd for $C_{26}H_{34}N_3O_3S^+$ 468.2315; Found 468.2322.

*2-Acetyl-4-cyano-N-phenyl-3-(piperidin-1-yl)-1-thiaspiro[4.7]dodec-3-ene-2-carboxamide (**1k**).* Product **1k** was obtained from 2-cyclooctylidene-3-(piperidin-1-yl)-3-thioxopropanenitrile (300 mg, 1.0 equiv, 1.09 mmol), 2-diazo-3-oxo-*N*-phenylbutanamide (443 mg, 2.0 equiv, 2.18 mmol), $[Cu(MeCN)_4]OTf$ (82 mg, 10 mol %), chloroform (6.0 mL), 90 °C, reaction time is 24 h. The purification of the crude product by column chromatography on SiO_2 (PE/EtOAc, gradient 25:0 to 32:18), affording **1k** as a yellow oily mass. The latter was triturated with hexane/diethyl ether (1:1) and centrifugated, affording **1k** as a colorless powder (54%, 250 mg), mp 180–182 °C. ^{1}H NMR (400 MHz, $CDCl_3$-*d*): *δ* 9.57 (s, 1H), 7.55 (d, *J* = 7.7 Hz, 2H), 7.34 (t, *J* = 7.9 Hz, 2H), 7.14 (t, *J* = 7.4 Hz, 1H), 3.42 – 3.39 (m, 2H), 3.34 – 3.29 (m, 2H), 2.47 (s, 3H), 2.41 – 2.34 (m, 2H), 2.29 – 2.23 (m, 1H), 2.10 – 2.04 (m, 1H), 1.90 – 1.81 (m, 2H), 1.66 – 1.49 (m, 14H). ^{13}C{1H} NMR (100 MHz, $CDCl_3$-*d*): *δ* 202.3, 166.1, 156.1, 137.6, 129.2, 124.9, 120.3, 118.9, 92.1, 70.3, 66.1, 51.4, 40.8, 39.2, 28.0, 27.8, 26.4, 25.9, 24.4, 24.2, 24.2, 23.6. HRMS (ESI) *m/z*: $[M + H]^+$ Calcd for $C_{26}H_{34}N_3O_2S^+$ 452.2366; Found 452.2371.

*2-Acetyl-4-cyano-3-(dimethylamino)-N-phenyl-1-thiaspiro[4.7]dodec-3-ene-2-carboxamide (**1l**)*. Product **1l** was obtained from 2-cyano-2-cyclooctylidene-*N*,*N*-dimethylethanethioamide (133 mg, 1.0 equiv, 0.56 mmol), 2-diazo-3-oxo-*N*-phenylbutanamide (229 mg, 2.0 equiv, 1.12 mmol), $[Cu(MeCN)_4]OTf$ (42 mg, 10 mol %), chloroform (6.0 mL), 85 °C, reaction time is 24 h. The purification of the crude product by column chromatography on SiO_2 (PE/EtOAc, gradient 25:0 to 25:25) afforded **1l** as a yellow oily mass. The latter was triturated with hexane/diethyl ether (1:1) and centrifugated; then the procedure was repeated in hexane (2 mL), affording **1l** as a colorless powder (41%, 94 mg), mp 185–187 °C. ^{1}H NMR (400 MHz, $CDCl_3$-*d*): *δ* 9.77 (s, 1H), 7.56 (d, *J* = 8.6 Hz, 2H), 7.34 (t, *J* = 7.9 Hz, 2H), 7.13 (t, *J* = 7.4 Hz, 1H), 3.06 (s,

6H), 2.48 (s, 3H), 2.47 – 2.25 (m, 3H), 2.16 – 2.08 (m, 1H), 1.91 – 1.84 (m, 2H), 1.66 – 1.47 (m, 8H). $^{13}C\{1H\}$ NMR (100 MHz, $CDCl_3$-*d*): *δ* 202.4, 166.1, 155.8, 137.6, 129.2, 125.0, 120.4, 119.3, 91.7, 69.6, 66.7, 42.9, 41.1, 39.4, 28.1, 27.7, 26.0, 24.2, 24.1. HRMS (ESI) *m/z*: $[M + H]^+$ Calcd for $C_{23}H_{30}N_3O_2S^+$ 412.2053; Found 412.2057.

*2-Acetyl-4-cyano-N-phenyl-3-(pyrrolidin-1-yl)-1-thiaspiro[4.7]dodec-3-ene-2-carboxamide (**1m**)*. Product **1m** was obtained from 2-cyclooctylidene-3-(pyrrolidin-1-yl)-3-thioxopropanenitrile (300 mg, 1.0 equiv, 1.14 mmol), 2-diazo-3-oxo-*N*-phenylbutanamide (463 mg, 2.0 equiv, 2.28 mmol), $[Cu(MeCN)_4]OTf$ (86 mg, 10 mol %), chloroform (6.0 mL), 90 °C, reaction time is 24 h. The purification of the crude product by column chromatography on SiO_2 (DCM) afforded **1m** as a yellow oily mass. The latter was triturated with hexane/diethyl ether (1:1) and centrifugated, affording **1m** as a colorless powder (55%, 269 mg), mp 136–138 °C. ^1H NMR (400 MHz, $CDCl_3$-*d*): *δ* 9.84 (s, 1H), 7.56 (d, *J* = 7.6 Hz, 2H), 7.34 (t, *J* = 7.9 Hz, 2H), 7.13 (t, *J* = 7.4 Hz, 1H), 3.59 – 3.53 (m, 2H), 3.47 – 3.42 (m, 2H), 2.50 (s, 3H), 2.47 – 2.36 (m, 2H), 2.34 – 2.27 (m, 1H), 2.17 – 2.11 (m, 1H), 1.92 – 1.83 (m, 6H), 1.66 – 1.52 (m, 8H). $^{13}C\{1H\}$ NMR (100 MHz, $CDCl_3$-*d*): *δ* 202.5, 166.1, 153.4, 137.7, 129.2, 124.9, 120.3, 119.9, 89.8, 69.7, 67.1, 50.8, 41.3, 39.6, 28.2, 27.7, 26.0, 25.6, 24.3, 24.3, 24.2. HRMS (ESI) *m/z*: $[M + H]^+$ Calcd for $C_{25}H_{32}N_3O_2S^+$ 438.2210; Found 438.2215.

*2-Acetyl-3-(azepan-1-yl)-4-cyano-N-phenyl-1-thiaspiro[4.7]dodec-3-ene-2-carboxamide (**1n**).* Product **1n** was obtained from 3-(azepan-1-yl)-2-cyclooctylidene-3-thioxopropanenitrile (300 mg, 1.0 equiv, 1.03 mmol), 2-diazo-3-oxo-*N*-phenylbutanamide (419 mg, 2.0 equiv, 2.06 mmol), $[Cu(MeCN)_4]OTf$ (78 mg, 10 mol %), chloroform (6.0 mL), 90 °C, reaction time is 24 h. The purification of the crude product by column chromatography on SiO_2 (DCM/PE, gradient 0:25 to 10:40), affording **1n** as a yellow oily mass. The latter was triturated with hexane/diethyl ether (1:1) and centrifugated, affording **1n** as a colorless powder (54%, 261 mg), mp 124–126 °C. ^1H NMR (400 MHz, $CDCl_3$-*d*): *δ* 9.93 (s, 1H), 7.56 (d, *J* = 7.7 Hz, 2H), 7.33 (t, *J* = 7.9 Hz, 2H), 7.13 (t, *J* = 7.4 Hz, 1H), 3.50 – 3.38 (m, 4H), 2.50 (s, 3H), 2.48 – 2.28 (m, 3H), 2.15 – 2.09 (m, 1H), 1.91 – 1.79 (m, 6H), 1.62 – 1.51 (m, 12H). $^{13}C\{1H\}$ NMR (100 MHz, $CDCl_3$-*d*): *δ* 202.4, 166.2, 155.0, 137.7, 129.2, 124.8, 120.4, 89.2, 69.6, 66.8, 52.9, 41.1, 39.7, 29.0, 28.2, 27.7, 27.1, 26.0, 24.2, 24.1. HRMS (ESI) *m/z*: $[M + H]^+$ Calcd for $C_{27}H_{36}N_3O_2S^+$ 466.2523; Found 466.2530.

*2-Acetyl-3-(azepan-1-yl)-4-cyano-N-phenyl-1-thiaspiro[4.5]dec-3-ene-2-carboxamide (**1o**).* Product **1o** was obtained from 3-(azepan-1-yl)-2-cyclohexylidene-3-

thioxopropanenitrile (186 mg, 1.0 equiv, 0.71 mmol), 2-diazo-3-oxo-*N*-phenylbutanamide (288 mg, 2.0 equiv, 1.42 mmol), [Cu(MeCN)$_4$]OTf (53 mg, 10 mol %), chloroform (6.0 mL), 90 °C, reaction time is 24 h. The purification of the crude product by column chromatography on SiO$_2$ (DCM/PE, gradient 0:25 to 40:10), affording **1o** as a yellow oily mass. The latter was additionally purified on neutral Al$_2$O$_3$ (EtOAc/PE, gradient 0:25 to 5:45) afforded **1o** as pale-yellow oil (54%, 167 mg). ^{1}H NMR (400 MHz, CDCl$_3$-*d*): δ 9.96 (s, 1H), 7.57 (d, *J* = 7.6 Hz, 2H), 7.34 (t, *J* = 7.9 Hz, 2H), 7.13 (t, *J* = 7.4 Hz, 1H), 3.51 – 3.38 (m, 4H), 2.51 (s, 3H), 2.17 – 2.09 (m, 2H), 2.01 – 1.93 (m, 2H), 1.82 – 1.73 (m, 6H), 1.66 – 1.61 (m, 5H), 1.47 – 1.39 (m, 1H), 1.36 – 1.27 (m, 1H), 1.22 – 1.14 (m, 1H). ^{13}C{1H} NMR (100 MHz, CDCl$_3$-*d*): δ 202.5, 166.1, 154.7, 137.7, 129.2, 124.8, 120.3, 119.2, 88.4, 69.4, 64.7, 52.7, 40.1, 39.8, 29.0, 27.1, 26.0, 24.5, 24.1. HRMS (ESI) *m/z*: [M + H]$^+$ Calcd for $C_{25}H_{32}N_3O_2S^+$ 438.2209; Found 438.2213.

Optimization study

Table 1: Optimization of the transformation of dihydrothiophene **1a**.

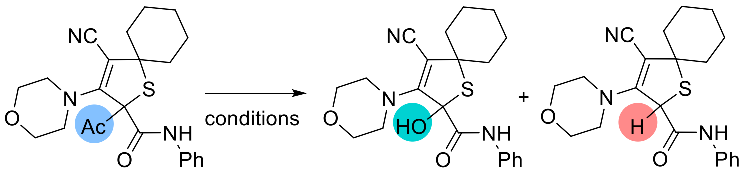

1a 2a 3a

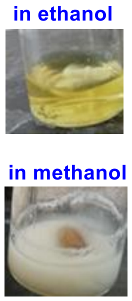

Entry	[M] or Base (equiv)	Solvent (mL)	[O]	Acid (mL)	Yields of **2a/3a**, %
1	Na (1)	EtOH (2)	O_2	–	22/36[1]
2	Na (2)	EtOH (2)	O_2	–	21/27[1]
3	Na (2)	EtOH (2)	O_2	HCl (0.25)	30/35[1]
4	Na (5)	EtOH (2)	O_2	HCl (0.25)	41/0
**5**	**Na (5)**	**EtOH (2)**	**O_2**	**HCl (0.25)**	**51/0[2]**
6	Na (5)	EtOH (2)	O_2	–	28/trace
7	Na (5)	EtOH (2)	O_2	HCl (0.25)	44/0[2,3]
8	Na (5)	MeOH (2)	O_2	HCl (0.25)	trace/71[2]
**9**	**Na (5)**	**MeOH (2)**	**O_2**	**HCl (0.25)**	**0/78**
10	Na (5)	*i*-PrOH (2)	O_2	HCl (0.25)	35/0[2]
11	Na (5)	*n*-BuOH (2)	O_2	HCl (0.25)	46/0[2]

12	Na (5)	TFE	O_2	HCl (0.25)	0/75
13	Na (5)	EtOH (2)	O_2	H_2SO_4 (0.25)	38/0[2]
14	K (5)	EtOH (2)	O_2	HCl (0.25)	40/0[2]
15	Na *tert*-pentoxide (5)	EtOH (2)	O_2	HCl (0.25)	37/0[2]
16	NaOH (5)	EtOH (2)	38% H_2O_2 (0.5)	–	0/62[2]
17	NaOH (5)	EtOH (2)	38% H_2O_2 (0.05)	–	0/73[2]
18	NaOH (5)	EtOH (2)	O_2	HCl (0.25)	23/0[2]
19	–	EtOH (2)	3-CPBA (1.1 equiv)	–	NR[2]

Conditions: dihydrothiophene **1a** (0.12mmol), dry solvent, rt, 1 h. Water (2 mL) or/and acid were added after evaporation of solvent. Isolated yields after centrifugation in Et_2O (2×1 mL). [1]Products were isolated as a mixture. [2]Oxygen was bubbled (1 min) after Na dissolving. [3]Ice bath. TFE – trifluoroethanol.

General procedure for derivatization of dihydrothiophenes 1 in ethanolic solution

Dihydrothiophene **1** (1.0 equiv) was dissolved in a freshly prepared solution of sodium ethoxide from sodium (5.0 equiv) and dry ethanol. The formed yellow solution was stirred at room temperature for 1 h and concentrated under reduced pressure. Water (2.0 mL), then concentrated HCl (0.25 mL) were added to the residue. The formed suspension was dissolved in DCM (3 mL), diluted with water (3 mL) and extracted twice. The organic layer was washed with water (5 mL), dried with anhydrous sodium sulfate, and concentrated under reduced pressure. The formed precipitate was centrifugated with cold diethyl ether, affording the derivatized products **2a-p** as a colorless powder.

*4-Cyano-2-hydroxy-3-morpholino-N-phenyl-1-thiaspiro[4.5]dec-3-ene-2-carboxamide (**2a**).* Dihydrothiophene **1a** (50 mg, 1.0 equiv, 0.12 mmol), sodium (13.0 mg, 5.0 equiv, 0.60 mmol) and dry ethanol (2.0 mL). Yield of **2a** 51% (24 mg), mp 248–250 °C. ^{1}H NMR (400 MHz, DMSO-d_6): δ 9.82 (s, 1H), 8.25 (s, 1H), 7.69 (d, J = 7.6 Hz, 2H), 7.30 (t, J = 7.4 Hz, 2H), 7.08 (t, J = 7.5 Hz, 1H), 3.66 – 3.58 (m, 4H), 3.48 – 3.44 (m, 2H), 3.37 – 3.34 (m, 2H), 1.98 – 1.82 (m, 3H), 1.74 – 1.65 (m, 3H), 1.60 – 1.56 (m, 1H), 1.34 – 1.08 (m, 3H). ^{13}C{1H} NMR (100 MHz, DMSO-d_6): δ 168.8, 158.0, 138.3, 128.5, 123.9, 120.4, 118.3, 90.3, 87.9, 65.7, 58.0, 48.8, 38.4, 24.4, 24.2, 23.4. HRMS (ESI) *m/z*: [M + Na]$^+$ Calcd for $C_{21}H_{25}N_3O_3SNa^+$ 422.1509; Found 422.1503.

*Scale-up synthesis of product **2a**.* Dihydrothiophene **1** (500 mg, 1.0 equiv, 1.17 mmol) was dissolved in a freshly prepared solution of sodium ethoxide from sodium (135 mg, 5.0 equiv, 5.87 mmol) and dry ethanol (5 mL). The formed yellow solution was stirred at room temperature for 1 h and concentrated under reduced pressure. Water (4.0 mL), then

concentrated HCl (1.5 mL) were added to the residue. The formed suspension was dissolved in DCM (10 mL), diluted with water (5 mL) and extracted twice. The organic layer was washed with water (10 mL), dried with anhydrous sodium sulfate, and concentrated under reduced pressure. The formed precipitate was centrifugated with cold diethyl ether (5 mL), affording product **2a** as a colorless powder (54%, 252 mg).

*4-Cyano-2-hydroxy-N-(4-methoxyphenyl)-3-morpholino-1-thiaspiro[4.5]dec-3-ene-2-carboxamide (**2b**).* Dihydrothiophene **1b** (80 mg, 1.0 equiv, 0.17 mmol), sodium (20.0 mg, 5.0 equiv, 0.85 mmol) and dry ethanol (2.5 mL). Yield of **2b** 41% (31 mg), mp 198–200 °C. ^{1}H NMR (400 MHz, DMSO-d_6): δ 9.69 (s, 1H), 8.15 (s, 1H), 7.58 (d, J = 9.0 Hz, 2H), 6.88 (d, J = 9.0 Hz, 2H), 3.72 (s, 3H), 3.65 – 3.58 (m, 4H), 3.49 – 3.43 (m, 2H), 3.39 – 3.33 (m, 2H), 1.94 – 1.83 (m, 3H), 1.74 – 1.65 (m, 3H), 1.60 – 1.57 (m, 1H), 1.32 – 1.23 (m, 2H), 1.15 – 1.07 (m, 1H). ^{13}C{1H} NMR (100 MHz, DMSO-d_6): δ 168.4, 158.1, 155.7, 131.3, 121.9, 118.4, 113.7, 90.2, 87.9, 65.7, 58.0, 55.2, 48.7, 38.4, 24.4, 24.2, 23.4. HRMS (ESI) *m/z*: [M + H]$^+$ Calcd for $C_{24}H_{30}N_3O_4S^+$ 456.1951; Found 456.1958.

*4-Cyano-2-hydroxy-3-morpholino-N-phenyl-1-thiaspiro[4.6]undec-3-ene-2-carboxamide (**2c**).* Dihydrothiophene **1c** (62 mg, 1.0 equiv, 0.14 mmol), sodium (16 mg, 5.0 equiv, 0.70 mmol) and dry ethanol (2.0 mL). Yield of **2c** 52% (30 mg), mp 244–245 °C. ^{1}H NMR (600 MHz, DMSO-d_6): δ 9.83 (s, 1H, NH), 8.25 (s, 1H, OH), 7.69 (d, J = 8.0 Hz, 2H), 7.30 (t, J = 7.5 Hz, 2H), 7.08 (t, J = 7.3 Hz, 1H), 3.64 – 3.58 (m, 4H), 3.44 – 3.42 (m, 2H), 3.34 – 3.31 (m, 2H), 2.16 – 2.02 (m, 3H), 1.91 – 1.87 (m, 1H), 1.71 – 1.65 (m, 2H), 1.61 – 1.35 (m, 6H). ^{13}C{1H} NMR (150 MHz, DMSO-d_6): δ 168.8, 157.6, 138.3, 128.6, 123.9, 120.4, 118.7, 90.4, 89.3, 65.7, 60.3, 48.8, 43.4, 42.5, 27.2, 27.2, 24.4, 23.3. HRMS (ESI) *m/z*: [M + H]$^+$ Calcd for $C_{22}H_{28}N_3O_3S^+$ 414.1846; Found 414.1844.

*4-Cyano-2-hydroxy-N-(4-methoxyphenyl)-3-morpholino-1-thiaspiro[4.6]undec-3-ene-2-carboxamide (**2d**).* Dihydrothiophene **1d** (90 mg, 1.0 equiv, 0.19 mmol), sodium (22 mg, 5.0 equiv, 0.95 mmol) and dry ethanol (3.0 mL). Yield of **2d** 57% (48 mg), mp 175–177 °C. ^{1}H NMR (400 MHz, DMSO-d_6): δ 9.71 (s, 1H), 8.17 (s, 1H), 7.58 (d, J = 9.1 Hz, 2H), 6.87 (d, J = 9.1 Hz, 2H), 3.72 (s, 3H), 3.65 – 3.56 (m, 4H), 3.45 – 3.41 (m, 2H), 3.36 – 3.30 (m, 2H), 2.16 – 2.01 (m, 3H), 1.92 – 1.86 (m, 1H), 1.72 – 1.36 (m, 8H). ^{13}C{1H} NMR (100 MHz, DMSO-d_6): δ 168.4, 157.6, 155.7, 131.3, 121.9, 118.7, 113.7, 90.4, 89.3, 65.7, 60.3, 55.2, 48.8, 43.4, 42.5, 27.2, 27.1, 24.3, 23.3. HRMS (ESI) *m/z*: [M + H]$^+$ Calcd for $C_{23}H_{30}N_3O_4S^+$ 444.1951; Found 444.1957.

*N-(4-Chlorophenyl)-4-cyano-2-hydroxy-3-morpholino-1-thiaspiro[4.6]undec-3-ene-2-carboxamide (**2e**).* Dihydrothiophene **1e** (80 mg, 1.0 equiv, 0.17 mmol), sodium (19 mg, 5.0 equiv, 0.84 mmol) and dry ethanol (2.5 mL). Yield of **2e** 62% (47 mg), mp 239–241 °C. ^{1}H NMR (400 MHz, DMSO-d_6): δ 10.00 (s, 1H), 8.27 (s, 1H), 7.74 (d, J = 9.0 Hz, 2H), 7.36 (d, J = 8.9 Hz, 2H), 3.65 – 3.56 (m, 4H), 3.45 – 3.39 (m, 2H), 3.36 – 3.29 (m, 2H), 2.17 – 2.01 (m, 3H), 1.92 – 1.86 (m, 1H), 1.72 – 1.35 (m, 9H). ^{13}C{1H} NMR (100 MHz, DMSO-d_6): δ 169.0, 157.4, 137.3, 128.4, 127.6, 122.0, 118.6, 90.4, 89.4, 65.7, 60.3, 48.8, 43.3, 42.5, 27.2, 27.1, 24.4, 23.3. HRMS (ESI) *m/z*: [M + H]$^+$ Calcd for $C_{22}H_{27}ClN_3O_3S^+$ 448.1456; Found 448.1461.

*4-Cyano-2-hydroxy-3-morpholino-N-phenyl-1-thiaspiro[4.7]dodec-3-ene-2-carboxamide (**2f**).* Dihydrothiophene **1f** (82 mg, 1.0 equiv, 0.18 mmol), sodium (21 mg, 5.0 equiv, 0.90 mmol) and dry ethanol (2.5 mL). Yield of **2f** 60% (46 mg), mp 225–228 °C. ^{1}H NMR (400 MHz, DMSO-d_6): δ 9.80 (s, 1H), 8.19 (s, 1H), 7.68 (d, J = 7.9 Hz, 2H), 7.30 (d, J = 7.4 Hz, 2H), 7.08 (t, J = 7.2 Hz, 1H), 3.63 – 3.59 (m, 4H), 3.47 – 3.36 (m, 4H), 2.28 – 2.22 (m, 1H), 2.15 – 2.04 (m, 3H), 1.81 – 1.75 (m, 2H), 1.59 – 1.48 (m, 8H). ^{13}C{1H} NMR (100 MHz, DMSO-d_6): δ 168.8, 158.1, 138.3, 128.5, 123.9, 120.4, 119.0, 90.4, 88.7, 65.7, 60.2, 48.9, 38.4, 27.5, 24.2, 23.4, 23.3. HRMS (ESI) *m/z*: [M + H]$^+$ Calcd for $C_{23}H_{30}N_3O_3S^+$ 428.2002; Found 428.2001.

*4-Cyano-2-hydroxy-N-(4-methoxyphenyl)-3-morpholino-1-thiaspiro[4.7]dodec-3-ene-2-carboxamide (**2g**).* Dihydrothiophene **1g** (80 mg, 1.0 equiv, 0.16 mmol), sodium (19.0 mg, 5.0 equiv, 0.80 mmol) and dry ethanol (2.5 mL). Yield of **2g** 61% (46 mg), mp 215–217 °C. ^{1}H NMR (400 MHz, DMSO-d_6): δ 9.69 (s, 1H), 8.11 (s, 1H), 7.58 (d, J = 9.0 Hz, 2H), 6.87 (d, J = 9.1 Hz, 2H), 3.72 (s, 3H), 3.67 – 3.56 (m, 4H), 3.46 – 3.41 (m, 2H), 3.37 – 3.31 (m, 2H), 2.27 – 2.22 (m, 1H), 2.14 – 2.09 (m, 3H), 1.83 – 1.72 (m, 2H), 1.58 – 1.48 (m, 8H). ^{13}C{1H} NMR (100 MHz, DMSO-d_6): δ 168.4, 158.2, 155.7, 131.3, 121.9, 119.0, 113.7, 90.4, 88.6, 65.7, 60.1, 55.2, 48.9, 38.4, 27.5, 24.2, 23.4, 23.3. HRMS (ESI) *m/z*: [M + H]$^+$ Calcd for $C_{24}H_{32}N_3O_4S^+$ 458.2108; Found 458.2110.

*N-(4-Chlorophenyl)-4-cyano-2-hydroxy-3-morpholino-1-thiaspiro[4.7]dodec-3-ene-2-carboxamide (**2h**).* Dihydrothiophene **1h** (80 mg, 1.0 equiv, 0.16 mmol), sodium (19.0 mg, 5.0 equiv, 0.80 mmol) and dry ethanol (2.5 mL). Yield of **2h** 54% (41 mg), mp 226–228 °C. ^{1}H NMR (400 MHz, DMSO-d_6): δ 9.99 (s, 1H), 8.24 (s, 1H), 7.73 (d, J = 8.9 Hz, 2H), 7.36 (d, J = 8.9 Hz, 2H), 3.66 – 3.56 (m, 4H), 3.45 – 3.40 (m, 2H), 3.36 – 3.33 (m, 2H), 2.28 – 2.22 (m, 1H), 2.14 – 2.02 (m, 3H), 1.83 – 1.70 (m, 2H), 1.59 – 1.44 (m, 8H). ^{13}C{1H} NMR (100 MHz, DMSO-d_6): δ 169.0, 158.1, 137.3, 128.5, 127.7, 122.0, 119.0, 90.4, 88.7,

65.7, 60.2, 48.9, 38.4, 27.5, 24.2, 23.4, 23.3. HRMS (ESI) *m/z*: $[M + H]^+$ Calcd for $C_{23}H_{29}ClN_3O_3S^+$ 462.1612; Found 462.1619.

*4-Cyano-N-(4-fluorophenyl)-2-hydroxy-3-morpholino-1-thiaspiro[4.7]dodec-3-ene-2-carboxamide (**2i**).* Dihydrothiophene **1i** (80 mg, 1.0 equiv, 0.17 mmol), sodium (20.0 mg, 5.0 equiv, 0.85 mmol) and dry ethanol (2.5 mL). Yield of **2i** 55% (41 mg), mp 243–245 °C. ^{1}H NMR (600 MHz, DMSO-d_6): δ 9.93 (s, 1H), 8.22 (s, 1H), 7.72 – 7.70 (m, 2H), 7.16 – 7.13 (m, 2H), 3.64 – 3.58 (m, 4H), 3.45 – 3.41 (m, 2H), 3.35 – 3.32 (m, 2H), 2.26 – 2.23 (m, 1H), 2.13 – 2.06 (m, 3H), 1.82 – 1.73 (m, 2H), 1.57 – 1.43 (m, 8H). ^{13}C{1H} NMR (150 MHz, DMSO-d_6): δ 168.9, 158.5 (d, J = 240.7 Hz), 158.1, 134.71 (d, J = 2.0 Hz), 122.3 (d, J = 7.8 Hz), 119.0, 115.1 (d, J = 22.2 Hz), 89.5 (d, J = 261.8 Hz), 65.8, 60.2, 48.9, 38.4, 27.5, 24.2, 23.4, 23.3. HRMS (ESI) *m/z*: $[M + H]^+$ Calcd for $C_{23}H_{29}FN_3O_3S^+$ 446.1908; Found 446.1912.

*4-Cyano-2-hydroxy-3-morpholino-N-(p-tolyl)-1-thiaspiro[4.7]dodec-3-ene-2-carboxamide (**2j**).* Dihydrothiophene **1j** (80 mg, 1.0 equiv, 0.17 mmol), sodium (20.0 mg, 5.0 equiv, 0.85 mmol) and dry ethanol (2.5 mL). Yield of **2j** 55% (41 mg), mp 214–217 °C. ^{1}H NMR (400 MHz, DMSO-d_6): δ 9.70 (s, 1H), 8.14 (s, 1H), 7.55 (d, J = 8.2 Hz, 2H), 7.10 (d, J = 8.1 Hz, 2H), 3.64 – 3.57 (m, 4H), 3.46 – 3.40 (m, 2H), 3.37 – 3.34 (m, 2H), 2.25 – 2.22 (m, 4H), 2.14 – 2.07 (m, 3H), 1.80 – 1.71 (m, 2H), 1.56 – 1.43 (m, 8H). ^{13}C{1H} NMR (100 MHz, DMSO-d_6): δ 168.6, 158.2, 135.7, 132.9, 128.9, 120.4, 119.0, 90.4, 88.7, 65.7, 60.2, 48.9, 38.4, 27.5, 24.2, 23.4, 23.3, 20.4. HRMS (ESI) *m/z*: $[M + H]^+$ Calcd for $C_{24}H_{32}N_3O_3S^+$ 442.2159; Found 442.2164.

*4-Cyano-2-hydroxy-N-phenyl-3-(piperidin-1-yl)-1-thiaspiro[4.7]dodec-3-ene-2-carboxamide (**2k**).* Dihydrothiophene **1k** (60 mg, 1.0 equiv, 0.13 mmol), sodium (15 mg, 5.0 equiv, 0.65 mmol) and dry ethanol (2.5 mL). Yield of **2k** 53%, (30 mg), mp 253–255 °C (decomp.). ^{1}H NMR (400 MHz, DMSO-d_6): δ 9.76 (s, 1H), 8.06 (s, 1H), 7.69 (d, J = 7.8 Hz, 2H), 7.30 (t, J = 7.9 Hz, 2H), 7.08 (t, J = 7.3 Hz, 1H), 3.45 – 3.40 (m, 2H), 3.34 (br s, 2H), 2.27 – 2.22 (m, 1H), 2.14 – 2.07 (m, 3H), 1.82 – 1.71 (m, 2H), 1.54 – 1.48 (m, 14H). ^{13}C{1H} NMR (100 MHz, DMSO-d_6): δ 168.9, 158.1, 138.4, 128.5, 123.8, 120.4, 119.5, 90.4, 86.7, 60.0, 49.9, 38.5, 27.5, 25.5, 24.2, 23.4, 23.3. HRMS (ESI) *m/z*: $[M + H]^+$ Calcd for $C_{24}H_{32}N_3O_2S^+$ 426.2210; Found 426.2212.

*4-Cyano-3-(dimethylamino)-2-hydroxy-N-phenyl-1-thiaspiro[4.7]dodec-3-ene-2-carboxamide (**2l**) and 4-cyano-3-(dimethylamino)-N-phenyl-1-thiaspiro[4.7]dodec-3-ene-2-carboxamide (**2l'**).* Dihydrothiophene **1l** (74 mg, 1.0 equiv, 0.18 mmol), sodium (21.0

mg, 5.0 equiv, 0.90 mmol) and dry ethanol (2.5 mL). The products were isolated as a mixture (38 mg) in ratio **2l**:**2l'** = 1.0:1.25 Yield of **2l** 25% (17 mg) and **2l'** 32% (21 mg), mp 182–184 °C. ^{1}H NMR (400 MHz, DMSO-d_6): δ 10.23 (s, 1H), 9.78 (s, 1H), 8.05 (s, 1H), 7.69 (d, J = 7.9 Hz, 2H), 7.55 (d, J = 7.8 Hz, 2H), 7.34 – 7.28 (m, 4H), 7.08 (t, J = 7.4 Hz, 2H), 4.90 (s, 1H), 3.04 (s, 6H), 3.02 (s, 6H), 2.27 – 2.05 (m, 9H), 1.81 – 1.67 (m, 4H), 1.51 – 1.48 (m, 15H). ^{13}C{1H} NMR (100 MHz, DMSO-d_6): δ 168.7, 168.0, 158.3, 156.7, 138.7, 138.3, 128.9, 128.5, 123.9, 123.8, 120.4, 120.1, 119.8, 119.3, 90.8, 87.1, 85.7, 64.4, 60.5, 52.3, 41.7, 41.7, 28.0, 27.5, 27.5, 27.1, 24.2, 24.0, 23.7, 23.4, 23.3, 23.2. HRMS (ESI) *m/z*: [M + H]$^+$ Calcd for $C_{21}H_{28}N_3O_2S^+$ 386.1897; Found 386.1900; [M + H]$^+$ Calcd for $C_{21}H_{28}N_3OS^+$ 370.1947; Found 370.1952.

*4-Cyano-N-phenyl-3-(pyrrolidin-1-yl)-1-thiaspiro[4.7]dodec-3-ene-2-carboxamide (**2m**).* Dihydrothiophene **1m** (90 mg, 1.0 equiv, 0.21 mmol), sodium (24 mg, 5.0 equiv, 1.05 mmol) and dry ethanol (3.0 mL). Yield of **2m** 67%, (56 mg), mp 123–125 °C. ^{1}H NMR (400 MHz, $CDCl_3$-d): δ 8.22 (s, 1H), 7.50 (d, J = 7.9 Hz, 2H), 7.34 (t, J = 7.8 Hz, 2H), 7.14 (t, J = 7.4 Hz, 1H), 4.61 (s, 1H), 3.71 – 3.65 (m, 4H), 2.41 – 2.29 (m, 2H), 2.19 – 2.13 (m, 1H), 2.07 – 2.01 (m, 1H), 1.94 – 1.82 (m, 6H), 1.72 – 1.49 (m, 9H). ^{13}C{1H} NMR (100 MHz, $CDCl_3$-d): δ 167.4, 153.6, 137.4, 129.3, 125.1, 120.7, 120.0, 85.5, 65.8, 54.6, 50.8, 40.8, 40.1, 28.2, 27.8, 25.6, 24.8, 24.2, 23.7. HRMS (ESI) *m/z*: [M + H]$^+$ Calcd for $C_{23}H_{30}N_3OS^+$ 396.2104; Found 396.2107.

*3-(Azepan-1-yl)-4-cyano-N-phenyl-1-thiaspiro[4.7]dodec-3-ene-2-carboxamide (**2n**).* Dihydrothiophene **1n** (74 mg, 1.0 equiv, 0.16 mmol), sodium (18 mg, 5.0 equiv, 0.80 mmol) and dry ethanol (2.5 mL). Yield of **2n** 60%, (40 mg), mp 127–131 °C. ^{1}H NMR (400 MHz, $CDCl_3$-d): δ 8.12 (s, 1H), 7.49 (d, J = 7.8 Hz, 2H), 7.34 (t, J = 7.6 Hz, 2H), 7.14 (t, J = 7.1 Hz, 1H), 4.67 (s, 1H), 3.70 – 3.60 (m, 4H), 2.43 – 2.37 (m, 1H), 2.34 – 2.28 (m, 1H), 2.17 – 2.11 (m, 1H), 2.04 – 1.98 (m, 1H), 1.89 – 1.83 (m, 4H), 1.76 – 1.48 (m, 16H). ^{13}C{1H} NMR (100 MHz, $CDCl_3$-d): δ 167.6, 154.8, 137.5, 129.3, 125.0, 120.3, 120.0, 85.6, 65.5, 54.0, 53.0, 40.6, 40.3, 29.2, 28.2, 27.7, 27.0, 24.8, 24.1, 23.6. HRMS (ESI) *m/z*: [M + H]$^+$ Calcd for $C_{25}H_{34}N_3OS^+$ 424.2417; Found 424.2417.

*3-(Azepan-1-yl)-4-cyano-N-phenyl-1-thiaspiro[4.5]dec-3-ene-2-carboxamide (**2o**).* Dihydrothiophene **1o** (60 mg, 1.0 equiv, 0.14 mmol), sodium (16 mg, 5.0 equiv, 0.70 mmol) and dry ethanol (2.5 mL). Yield of **2o** 66%, (36 mg), mp 186–188 °C. ^{1}H NMR (400 MHz, $CDCl_3$-d): δ 8.08 (s, 1H), 7.49 (d, J = 7.8 Hz, 2H), 7.33 (t, J = 7.9 Hz, 2H), 7.14 (t, J = 7.4 Hz, 1H), 4.67 (s, 1H), 3.68 – 3.57 (m, 4H), 2.13 – 2.06 (m, 1H), 1.95 – 1.93 (m, 2H), 1.85 – 1.74 (m, 7H), 1.65 – 1.51 (m, 6H), 1.39 – 1.29 (m, 1H), 1.22 – 1.11 (m, 1H). ^{13}C{1H}

NMR (100 MHz, $CDCl_3$-*d*): *δ* 167.9, 154.5, 137.5, 129.3, 125.0, 120.0, 84.8, 63.2, 54.2, 52.8, 40.5, 40.0, 29.1, 26.9, 24.7, 23.8. HRMS (ESI) *m/z*: $[M + H]^+$ Calcd for $C_{23}H_{30}N_3OS^+$ 396.2104; Found 396.2110.

General procedure for deacetylation of dihydrothiophenes 1 in methanolic solution

Dihydrothiophene **1** (1.0 equiv) was dissolved in a freshly prepared solution of sodium methoxide from sodium (5.0 equiv) and dry methanol. The formed colorless suspension was stirred at room temperature for 1 h and concentrated under reduced pressure. Water (2.0 mL), then concentrated HCl (0.25 mL) were added to the residue. The formed suspension was dissolved in DCM (3 mL), diluted with water (3 mL) and extracted twice. The organic layer was washed with water (5 mL), dried with anhydrous sodium sulfate, and concentrated under reduced pressure. The formed precipitate was centrifugated with cold diethyl ether and hexane (1:1), affording the deacetylated product **3a**–**k, 2o,m,n** as a colorless powder.

*4-Cyano-3-morpholino-N-phenyl-1-thiaspiro[4.5]dec-3-ene-2-carboxamide (**3a**).* Dihydrothiophene **1a** (47 mg, 1.0 equiv, 0.11 mmol), sodium (13 mg, 5.0 equiv, 0.55 mmol) and dry methanol (2.0 mL). Yield of **3a** 78%, (33 mg), mp 193–195 °C. ^{1}H NMR (400 MHz, DMSO-d_6): *δ* 10.21 (s, 1H), 7.55 (d, *J* = 7.7 Hz, 2H), 7.33 (t, *J* = 7.9 Hz, 2H), 7.09 (t, *J* = 7.4 Hz, 1H), 4.93 (s, 1H), 3.66 – 3.56 (m, 4H), 3.45 – 3.43 (m, 4H), 2.03 (d, *J* = 12.9 Hz, 1H), 1.94 – 1.87 (m, 1H), 1.80 – 1.56 (m, 5H), 1.47 – 1.36 (m, 1H), 1.24 – 1.05 (m, 2H). ^{13}C{1H} NMR (100 MHz, DMSO-d_6): *δ* 167.9, 156.0, 138.6, 128.9, 123.8, 119.4, 118.5, 87.8, 65.5, 62.1, 52.0, 48.8, 24.3, 23.8, 23.5. HRMS (ESI) *m/z*: $[M + H]^+$ Calcd for $C_{21}H_{26}N_3O_2S^+$ 384.1740; Found 384.1743.

*4-Cyano-N-(4-methoxyphenyl)-3-morpholino-1-thiaspiro[4.5]dec-3-ene-2-carboxamide (**3b**).* Dihydrothiophene **1b** (50 mg, 1.0 equiv, 0.11 mmol), sodium (13 mg, 5.0 equiv, 0.55 mmol) and dry methanol (2.5 mL). Yield of **3b** 73%, (33 mg), mp 84–86 °C. ^{1}H NMR (400 MHz, $CDCl_3$-*d*): *δ* 8.19 (s, 1H), 7.37 (d, *J* = 9.0 Hz, 2H), 6.89 (d, *J* = 9.0 Hz, 1H), 4.74 (s, 1H), 3.80 (s, 3H), 3.77 – 3.69 (m, 6H), 3.56 – 3.48 (m, 2H), 2.09 – 1.93 (m, 2H), 1.89 – 1.80 (m, 4H), 1.71 – 1.59 (m, 2H), 1.44 – 1.33 (m, 1H), 1.22 – 1.17 (m, 1H). ^{13}C{1H} NMR (100 MHz, $CDCl_3$-*d*): *δ* 166.6, 157.2, 155.8, 130.2, 121.8, 118.3, 114.5, 89.9, 66.6, 62.9, 55.7, 54.2, 50.1, 40.4, 39.9, 24.7, 24.3, 24.0. HRMS (ESI) *m/z*: $[M + H]^+$ Calcd for $C_{22}H_{28}N_3O3S^+$ 414.1846; Found 414.1852.

*3-(Azepan-1-yl)-4-cyano-N-phenyl-1-thiaspiro[4.5]dec-3-ene-2-carboxamide (**2o**).* Dihydrothiophene **1o** (60 mg, 1.0 equiv, 0.14 mmol), sodium (16 mg, 5.0 equiv, 0.68 mmol) and dry methanol (2.5 mL). Yield of **2o** 67%, (36 mg), mp 186–188 °C. ^{1}H NMR (400 MHz, $CDCl_3$-*d*): *δ* 8.08 (s, 1H), 7.49 (d, *J* = 7.8 Hz, 2H), 7.33 (t, *J* = 7.9 Hz, 2H), 7.14 (t, *J* = 7.4 Hz, 1H), 4.67 (s, 1H), 3.68 – 3.57 (m, 4H), 2.13 – 2.06 (m, 1H), 1.95 – 1.93 (m, 2H), 1.85 – 1.74 (m, 7H), 1.65 – 1.51 (m, 6H), 1.39 – 1.29 (m, 1H), 1.22 – 1.11 (m, 1H).

*4-Cyano-3-morpholino-N-phenyl-1-thiaspiro[4.6]undec-3-ene-2-carboxamide (**3c**).* Dihydrothiophene **1c** (61 mg, 1.0 equiv, 0.14 mmol), sodium (16 mg, 5.0 equiv, 0.70 mmol) and dry methanol (2.5 mL). The product **3c** was purified on SiO_2 (eluent DCM/EtOAc, gradient 25:0 to 22:3). Yield of **3c** 75%, (41 mg), mp 167–169 °C. ^{1}H NMR (400 MHz, $CDCl_3$-*d*): *δ* 8.41 (s, 1H), 7.50 (d, *J* = 7.8 Hz, 2H), 7.38 (t, *J* = 7.9 Hz, 2H), 7.19 (t, *J* = 7.4 Hz, 1H), 4.79 (s, 1H), 3.81 – 3.70 (m, 6H), 3.57 – 3.50 (m, 2H), 2.32 – 2.11 (m, 3H), 2.06 – 2.00 (m, 1H), 1.85 – 1.79 (m, 2H), 1.73 – 1.54 (m, 6H). ^{13}C{1H} NMR (100 MHz, $CDCl_3$-*d*): *δ* 166.6, 155.3, 137.2, 129.4, 125.2, 119.9, 118.6, 91.4, 66.6, 65.2, 54.3, 50.1, 44.7, 43.0, 27.9, 27.7, 24.6, 24.0. HRMS (ESI) *m/z*: $[M + H]^+$ Calcd for $C_{22}H_{28}N_3O_2S^+$ 398.1897; Found 398.1902.

*4-Cyano-N-(4-methoxyphenyl)-3-morpholino-1-thiaspiro[4.6]undec-3-ene-2-carboxamide (**3d**).* Dihydrothiophene **1d** (57 mg, 1.0 equiv, 0.12 mmol), sodium (14 mg, 5.0 equiv, 0.61 mmol) and dry methanol (2.5 mL). Yield of **3d** 66%, (34 mg), mp 115–117 °C. ^{1}H NMR (400 MHz, $CDCl_3$-*d*): *δ* 8.31 (s, 1H), 7.37 (d, *J* = 8.9 Hz, 2H), 6.87 (d, *J* = 8.9 Hz, 2H), 4.74 (s, 1H), 3.79 – 3.67 (m, 3H+6H), 3.51 – 3.48 (m, 2H), 2.29 – 2.22 (m, 1H), 2.12 (br s, 2H), 2.01 – 1.96 (m, 1H), 1.79 (br s, 2H), 1.66 – 1.54 (m, 6H). ^{13}C{1H} NMR (100 MHz, $CDCl_3$-*d*): *δ* 166.6, 157.1, 155.4, 130.3, 121.8, 114.5, 91.3, 66.6, 65.2, 55.6, 54.0, 50.1, 44.6, 43.0, 27.9, 27.7, 24.6, 24.0. HRMS (ESI) *m/z*: $[M + H]^+$ Calcd for $C_{23}H_{30}N_3O_3S^+$ 428.2002; Found 428.2007.

*N-(4-Chlorophenyl)-4-cyano-3-morpholino-1-thiaspiro[4.6]undec-3-ene-2-carboxamide (**3e**).* Dihydrothiophene **1e** (50 mg, 1.0 equiv, 0.11 mmol), sodium (12 mg, 5.0 equiv, 0.55 mmol) and dry methanol (2.5 mL). Yield of **3e** 74%, (35 mg), mp 143–145 °C. ^{1}H NMR (400 MHz, $CDCl_3$-*d*): *δ* 8.46 (s, 1H), 7.44 (d, *J* = 8.8 Hz, 2H), 7.32 (d, *J* = 8.8 Hz, 2H), 4.77 (s, 1H), 3.78 – 3.67 (m, 6H), 3.54 – 3.49 (m, 2H), 2.30 – 2.23 (m, 1H), 2.20 – 1.97 (m, 3H), 1.83 – 1.77 (m, 2H), 1.70 – 1.55 (m, 6H). ^{13}C{1H} NMR (100 MHz, $CDCl_3$-*d*): *δ* 166.7, 155.1, 135.9, 130.2, 129.4, 121.1, 118.5, 91.6, 66.6, 65.3, 54.1, 50.2, 49.4, 44.7, 43.0, 27.9, 27.7, 24.7, 24.0. HRMS (ESI) *m/z*: $[M + H]^+$ Calcd for $C_{22}H_{27}ClN_3O_2S^+$ 432.1507; Found 432.1513.

4-Cyano-3-morpholino-N-phenyl-1-thiaspiro[4.7]dodec-3-ene-2-carboxamide ***(3f).*** Dihydrothiophene **1f** (80 mg, 1.0 equiv, 0.18 mmol), sodium (20 mg, 5.0 equiv, 0.88 mmol) and dry methanol (3.0 mL). Yield of **3f** 76%, (55 mg), mp 107–109 °C. ^{1}H NMR (400 MHz, $CDCl_3$-*d*): δ 8.52 (s, 1H), 7.49 (d, *J* = 7.7 Hz, 2H), 7.36 (t, *J* = 7.9 Hz, 2H), 7.16 (t, *J* = 7.4 Hz, 1H), 4.78 (s, 1H), 3.81 – 3.66 (m, 6H), 3.56 – 3.47 (m, 2H), 2.42 – 2.36 (m, 1H), 2.32 – 2.25 (m, 1H), 2.18 – 2.12 (m, 1H), 2.07 – 2.01 (m, 1H), 1.91 – 1.82 (m, 2H), 1.79 – 1.70 (m, 1H), 1.67 – 1.52 (m, 7H). ^{13}C{1H} NMR (100 MHz, $CDCl_3$-*d*): δ 166.5, 156.1, 137.3, 129.3, 125.2, 119.9, 118.8, 90.9, 66.6, 65.0, 54.0, 50.3, 41.1, 39.2, 28.2, 27.7, 24.5, 24.1, 23.9. HRMS (ESI) *m/z*: $[M + H]^+$ Calcd for $C_{23}H_{30}N_3O_2S^+$ 412.2053; Found 412.2058.

4-Cyano-N-(4-methoxyphenyl)-3-morpholino-1-thiaspiro[4.7]dodec-3-ene-2-carboxamide ***(3g).*** Dihydrothiophene **1g** (80 mg, 1.0 equiv, 0.16 mmol), sodium (19 mg, 5.0 equiv, 0.83 mmol) and dry methanol (3.0 mL). The product **3g** was purified on neutral Al_2O_3 (eluent PE/EtOAc, gradient 25:0 to 20:30). Yield of **3g** 55% (40 mg), mp 154–156 °C. ^{1}H NMR (400 MHz, $CDCl_3$-*d*): δ 8.43 (s, 1H), 7.38 (d, *J* = 9.0 Hz, 2H), 6.88 (d, *J* = 9.0 Hz, 2H), 4.76 (s, 1H), 3.79 (s, 3H), 3.78 – 3.77 (m, 1H), 3.76 – 3.74 (m, 1H), 3.73 – 3.67 (m, 4H), 3.53 – 3.48 (m, 2H), 2.41 – 2.36 (m, 1H), 2.31 – 2.24 (m, 1H), 2.18 – 2.11 (m, 1H), 2.06 – 2.00 (m, 1H), 1.90 – 1.81 (m, 2H), 1.79 – 1.71 (m, 1H), 1.65 – 1.52 (m, 7H). ^{13}C{1H} NMR (100 MHz, $CDCl_3$-*d*): δ 166.4, 157.1, 156.2, 130.3, 121.8, 118.9, 114.5, 90.7, 66.6, 65.0, 55.7, 53.8, 50.3, 41.0, 39.2, 28.2, 27.7, 24.5, 24.1, 23.9. HRMS (ESI) *m/z*: $[M + H]^+$ Calcd for $C_{24}H_{32}N_3O_3S^+$ 442.2159; Found 442.2164.

N-(4-Chlorophenyl)-4-cyano-3-morpholino-1-thiaspiro[4.7]dodec-3-ene-2-carboxamide ***(3h).*** Dihydrothiophene **1h** (66 mg, 1.0 equiv, 0.14 mmol), sodium (16 mg, 5.0 equiv, 0.70 mmol) and dry methanol (2.5 mL). Yield of **3h** 71%, (43 mg), mp 167–169 °C. ^{1}H NMR (400 MHz, $CDCl_3$-*d*): δ 8.55 (s, 1H), 7.45 (d, *J* = 8.6 Hz, 2H), 7.31 (d, *J* = 8.6 Hz, 2H), 4.77 (s, 1H), 3.79 – 3.68 (m, 6H), 3.52 – 3.47 (m, 2H), 2.41 – 2.35 (m, 1H), 2.29 – 2.23 (m, 1H), 2.17 – 2.09 (m, 1H), 2.06 – 2.00 (m, 1H), 1.88 – 1.84 (m, 2H), 1.76 – 1.71 (m, 1H), 1.66 – 1.52 (m, 7H). ^{13}C{1H} NMR (100 MHz, $CDCl_3$-*d*): δ 166.5, 156.0, 135.8, 130.2, 129.4, 121.1, 118.8, 90.9, 66.6, 65.1, 53.8, 50.3, 41.1, 39.2, 28.2, 27.6, 24.5, 24.1, 23.8. HRMS (ESI) *m/z*: $[M + H]^+$ Calcd for $C_{23}H_{29}ClN_3O_2S^+$ 446.1663; Found 446,1672.

4-Cyano-N-(4-fluorophenyl)-3-morpholino-1-thiaspiro[4.7]dodec-3-ene-2-carboxamide ***(3i).*** Dihydrothiophene **1i** (70 mg, 1.0 equiv, 0.15 mmol), sodium (17 mg, 5.0 equiv, 0.74 mmol) and dry methanol (2.5 mL). Yield of **3i** 68%, (43 mg), mp 207–209 °C. ^{1}H NMR (600 MHz, $CDCl_3$-*d*): δ 8.54 (s, 1H), 7.46 – 7.44 (m, 2H), 7.04 (t, *J* = 8.5 Hz, 2H), 4.76 (s, 1H), 3.79 – 3.74 (m, 2H), 3.72 – 3.68 (m, 4H), 3.53 – 3.47 (m, 2H), 2.39 – 2.36 (m, 1H),

2.28 – 2.24 (m, 1H), 2.15 – 2.11 (m, 1H), 2.04 – 2.00 (m, 1H), 1.88 – 1.81 (m, 2H), 1.76 – 1.70 (m, 1H), 1.64 – 1.51 (m, 7H). $^{13}C\{1H\}$ NMR (150 MHz, $CDCl_3$-*d*): δ 166.6, 159.8 (d, J = 244.8 Hz), 156.1, 133.3 (d, J = 2.6 Hz), 121.8 (d, J = 7.9 Hz), 118.9, 116.0 (d, J = 22.6 Hz), 90.7, 66.6, 65.0, 53.6, 50.2, 40.9, 39.1, 28.1, 27.6, 24.5, 24.0, 23.8. HRMS (ESI) *m/z*: $[M + H]^+$ Calcd for $C_{23}H_{29}FN_3O_2S^+$ 430.1959; Found 430.1965.

4-Cyano-3-morpholino-N-(p-tolyl)-1-thiaspiro[4.7]dodec-3-ene-2-carboxamide ***(3j)****.* Dihydrothiophene **1j** (50 mg, 1.0 equiv, 0.11 mmol), sodium (13 mg, 5.0 equiv, 0.55 mmol) and dry methanol (2.5 mL). Yield of **3j** 64%, (29 mg), mp 110–113 °C. ^1H NMR (400 MHz, DMSO-d_6): δ 10.12 (s, 1H), 7.43 (d, J = 6.8 Hz, 2H), 7.13 (d, J = 6.9 Hz, 2H), 4.95 (s, 1H), 3.60 (br s, 4H), 3.42 (br s, 4H), 2.26 – 2.19 (m, 6H), 1.98 – 1.92 (m, 1H), 1.79 – 1.70 (m, 2H), 1.49 (br s, 7H). $^{13}C\{1H\}$ NMR (100 MHz, DMSO-d_6): δ 167.8, 156.6, 136.1, 132.8, 129.2, 119.4, 119.1, 88.5, 65.6, 64.0, 52.0, 48.9, 30.7, 27.9, 27.1, 23.9, 23.7, 23.2, 20.4. HRMS (ESI) *m/z*: $[M + H]^+$ Calcd for $C_{24}H_{32}N_3O_2S^+$ 426.2210; Found 426.2214.

4-Cyano-N-phenyl-3-(pyrrolidin-1-yl)-1-thiaspiro[4.7]dodec-3-ene-2-carboxamide ***(2m)****.* Dihydrothiophene **1m** (60 mg, 1.0 equiv, 0.14 mmol), sodium (16 mg, 5.0 equiv, 0.70 mmol) and dry methanol (2.5 mL). Yield of **2m** 66%, (36 mg), mp 123–125 °C. ^1H NMR (400 MHz, $CDCl_3$-*d*): δ 8.22 (s, 1H), 7.50 (d, J = 7.9 Hz, 2H), 7.34 (t, J = 7.8 Hz, 2H), 7.14 (t, J = 7.4 Hz, 1H), 4.61 (s, 1H), 3.71 – 3.65 (m, 4H), 2.41 – 2.29 (m, 2H), 2.19 – 2.13 (m, 1H), 2.07 – 2.01 (m, 1H), 1.94 – 1.82 (m, 6H), 1.72 – 1.49 (m, 8H).

3-(Azepan-1-yl)-4-cyano-N-phenyl-1-thiaspiro[4.7]dodec-3-ene-2-carboxamide ***(2n)****.* Dihydrothiophene **1n** (60 mg, 1.0 equiv, 0.13 mmol), sodium (15 mg, 5.0 equiv, 0.65 mmol) and dry methanol (2.5 mL). Yield of **2n** 60%, (32 mg), mp 128–130 °C. ^1H NMR (400 MHz, $CDCl_3$-*d*): δ 8.12 (s, 1H), 7.49 (d, J = 7.8 Hz, 2H), 7.34 (t, J = 7.6 Hz, 2H), 7.14 (t, J = 7.1 Hz, 1H), 4.67 (s, 1H), 3.70 – 3.60 (m, 4H), 2.43 – 2.37 (m, 1H), 2.34 – 2.28 (m, 1H), 2.17 – 2.11 (m, 1H), 2.04 – 1.98 (m, 1H), 1.89 – 1.83 (m, 4H), 1.76 – 1.48 (m, 14H).

4-Cyano-N-phenyl-3-(piperidin-1-yl)-1-thiaspiro[4.7]dodec-3-ene-2-carboxamide ***(3k)****.* Dihydrothiophene **1k** (60 mg, 1.0 equiv, 0.13 mmol), sodium (15 mg, 5.0 equiv, 0.65 mmol) and dry methanol (2.5 mL). Yield of **3k** 70%, (38 mg), mp 177–178 °C. ^1H NMR (400 MHz, $CDCl_3$-*d*): δ 8.29 (s, 1H), 7.49 (d, J = 7.8 Hz, 2H), 7.34 (t, J = 7.6 Hz, 2H), 7.14 (t, J = 7.3 Hz, 1H), 4.77 (s, 1H), 3.61 – 3.58 (m, 2H), 3.50 – 3.41 (m, 2H), 2.40 – 2.26 (m, 2H), 2.18 – 2.12 (m, 1H), 2.06 – 2.00 (m, 1H), 1.89 – 1.80 (m, 2H), 1.73 – 1.50 (m, 14H). $^{13}C\{1H\}$ NMR (100 MHz, $CDCl_3$-*d*): δ 167.3, 156.0, 137.5, 129.2, 125.0, 120.0, 119.6,

88.9, 64.8, 54.3, 51.4, 41.1, 39.6, 28.2, 27.7, 26.1, 24.5, 24.2, 24.0, 23.9. HRMS (ESI) *m/z*: $[M + H]^+$ Calcd for $C_{24}H_{32}N_3OS^+$ 410.2261; Found 410.2267.

General procedure for derivatization of dihydrothiophenes 4a–f in ethanolic solution. Method A

Dihydrothiophene **4** (1.0 equiv) was dissolved in a freshly prepared solution of sodium ethoxide from sodium (5.0 equiv) and dry ethanol. The formed solution was stirred at room temperature for 1 h and concentrated under reduced pressure. Water (2.0 mL), then concentrated HCl (0.25 mL) were added to the residue. The formed suspension was dissolved in DCM (3 mL), diluted with water (3 mL) and extracted twice. The organic layer was washed with water (5 mL), dried with anhydrous sodium sulfate, and concentrated under reduced pressure. The formed precipitate was centrifugated with solvent (cold diethyl ether for **5a**, hexane for **5b**, hexane/ diethyl ether 1:1 for **5c**–**f**), affording the derivatized product **5a**–**f** as colorless powders.

Method B (one-pot)

A mixture of thioamide (1.0 equiv), 2-diazo-3-oxo-*N*-phenylbutanamide (1.5 equiv) and $Rh_2(Piv)_4$ (0.5 mol %) in chloroform (1.5 mL) was stirred at room temperature for 14 h. After the completion of the reaction, the solvent was evaporated, and the residue was dissolved in a freshly prepared solution of sodium ethoxide from sodium (5.0 equiv) and dry ethanol. The formed solution was stirred at room temperature for 1 h and concentrated under reduced pressure. Water (2.0 mL), then concentrated HCl (0.25 mL) was added to the residue. The formed suspension was dissolved in DCM (3 mL), diluted with water (3 mL) and extracted twice. The organic layer was washed with water (5 mL), dried with anhydrous sodium sulfate and concentrated under reduced pressure. The purification of the crude product by column chromatography on SiO_2 afforded product **5a** as a colorless powder.

*4-Cyano-3-morpholino-N-phenyl-2-thiaspiro[4.5]dec-3-ene-1-carboxamide (**5a**).*

Method A. Dihydrothiophene **4a** (80 mg, 1.0 equiv, 0.19 mmol), sodium (22 mg, 5.0 equiv, 0.94 mmol) and dry ethanol (3.0 mL). Yield of **5a** 74% (53 mg), mp 122–124 °C. ^{1}H NMR (400 MHz, DMSO-*d*$_6$): δ 9.80 (s, 1H), 8.19 (s, 1H), 7.68 (d, *J* = 7.9 Hz, 2H), 7.30 (d, *J* = 7.4 Hz, 2H), 7.08 (t, *J* = 7.2 Hz, 1H), 3.63 – 3.59 (m, 4H), 3.47 – 3.36 (m, 4H), 2.28 – 2.22 (m, 1H), 2.15 – 2.04 (m, 3H), 1.81 – 1.75 (m, 2H), 1.59 – 1.48 (m, 8H). ^{13}C{1H} NMR (100 MHz, $CDCl_3$-*d*): δ 167.4, 162.6, 137.2, 129.2, 125.1, 120.5, 119.1, 80.3, 77.4,

66.4, 55.3, 54.5, 51.1, 35.2, 32.3, 25.3, 22.9. HRMS (ESI) *m/z*: $[M + H]^+$ Calcd for $C_{21}H_{26}N_3O_2S^+$ 384.1740; Found 384.1740.

Method B. 2-Cyclohexylidene-3-morpholino-3-thioxopropanenitrile (64 mg, 1.0 equiv, 0.25 mmol), 2-diazo-3-oxo-*N*-phenylbutanamide (78 mg, 1.5 equiv, 0.38 mmol), $Rh_2(Piv)_4$ (1.17 mg), chloroform (1.5 mL). Then sodium (9.2 mg, 2.0 equiv, 0.40 mmol) and dry ethanol (2.0 mL). After the purification of the crude product by column chromatography on SiO_2 (DCM/EtOAc, gradient 25:0 to 20:5) and evaporation of the solvent under reduced pressure, the obtained residue was triturated with *n*-hexane and centrifugated, affording **5a** as a colorless powder (64%, 63 mg), mp 122–124 °C.

*4-Cyano-3-morpholino-N-phenyl-2-thiaspiro[4.4]non-3-ene-1-carboxamide (**5b**).* Dihydrothiophene **4b** (80 mg, 1.0 equiv, 0.19 mmol), sodium (22 mg, 5.0 equiv, 0.97 mmol) and dry ethanol (3.0 mL). Yield of **5b** 58% (42 mg), mp 151–152 °C. ^{1}H NMR (400 MHz, $CDCl_3$-*d*): *δ* 7.85 (s, 1H), 7.47 (d, *J* = 7.8 Hz, 2H), 7.35 (t, *J* = 7.7 Hz, 2H), 7.16 (t, *J* = 7.6 Hz, 1H), 3.84 (s, 1H), 3.77 – 3.61 (m, 8H), 2.16 – 2.12 (m, 1H), 2.01 – 1.95 (m, 1H), 1.84 – 1.73 (m, 5H). ^{13}C{1H} NMR (100 MHz, $CDCl_3$-*d*): *δ* 167.1, 162.0, 137.1, 129.3, 125.2, 120.5, 118.8, 78.9, 66.4, 62.0, 58.9, 51.0, 39.7, 33.8, 24.7, 23.7. HRMS (ESI) *m/z*: $[M + H]^+$ Calcd for $C_{20}H_{24}N_3O_2S^+$ 370.1584; Found 370.1590.

*4-Cyano-3-morpholino-N-phenyl-2-thiaspiro[4.6]undec-3-ene-1-carboxamide (**5c**).* Dihydrothiophene **4c** (98 mg, 1.0 equiv, 0.22 mmol), sodium (26 mg, 5.0 equiv, 1.11 mmol) and dry ethanol (3.0 mL). Yield of **5c** 90% (80 mg), mp 178–180 °C. ^{1}H NMR (400 MHz, $CDCl_3$-*d*): *δ* 7.88 (s, 1H), 7.48 (d, *J* = 7.8 Hz, 2H), 7.34 (t, *J* = 7.8 Hz, 2H), 7.15 (t, *J* = 7.4 Hz, 1H), 3.87 (s, 1H), 3.76 – 3.74 (m, 4H), 3.67 – 3.57 (m, 4H), 2.23 – 2.17 (m, 1H), 2.11 – 2.00 (m, 2H), 1.90 – 1.84 (m, 1H), 1.75 – 1.59 (m, 5H), 1.52 – 1.40 (m, 3H). ^{13}C{1H} NMR (100 MHz, $CDCl_3$-*d*): *δ* 166.8, 162.0, 137.1, 129.3, 125.2, 120.4, 119.2, 81.4, 66.4, 59.4, 57.7, 51.1, 39.1, 34.9, 31.1, 30.6, 23.8, 23.7. HRMS (ESI) *m/z*: $[M + H]^+$ Calcd for $C_{22}H_{28}N_3O_2S^+$ 398.1897; Found 398.1908.

*4-Cyano-3-morpholino-N-phenyl-2-thiaspiro[4.7]dodec-3-ene-1-carboxamide (**5d**).* Dihydrothiophene **4d** (60 mg, 1.0 equiv, 0.13 mmol), sodium (15 mg, 5.0 equiv, 0.66 mmol) and dry ethanol (2.5 mL). Yield of **5d** 67% (36 mg), mp 131–133 °C. ^{1}H NMR (400 MHz, DMSO-d_6): *δ* 10.05 (s, 1H), 7.55 (d, *J* = 7.6 Hz, 2H), 7.32 (t, *J* = 7.9 Hz, 2H), 7.08 (t, *J* = 7.4 Hz, 1H), 4.04 (s, 1H), 3.66 – 3.64 (m, 4H), 3.57 – 3.45 (m, 4H), 2.03 – 1.89 (m, 4H), 1.71 – 1.55 (m, 8H), 1.46 – 1.35 (m, 2H). ^{13}C{1H} NMR (100 MHz, DMSO-d_6): *δ* 166.9, 163.5, 138.4, 128.8, 123.8, 119.7, 119.5, 78.2, 65.6, 56.9, 56.8, 50.6, 33.4, 29.4,

29.1, 27.1, 24.6, 22.7, 22.4. HRMS (ESI) *m/z*: $[M + H]^+$ Calcd for $C_{23}H_{29}N_3O_2S^+$ 412.2053; Found 412.2052.

4-Cyano-N-phenyl-3-(piperidin-1-yl)-2-thiaspiro[4.7]dodec-3-ene-1-carboxamide (***5e***). Dihydrothiophene **4e** (70 mg, 1.0 equiv, 0.15 mmol), sodium (17 mg, 5.0 equiv, 0.75 mmol) and dry ethanol (2.5 mL). Yield of **5e** 71% (45 mg), mp 112–115 °C. ^{1}H NMR (400 MHz, DMSO-d_6): δ 10.03 (s, 1H), 7.55 (d, J = 7.5 Hz, 2H), 7.32 (t, J = 7.0 Hz, 2H), 7.08 (t, J = 6.5 Hz, 1H), 4.00 (s, 1H), 3.52 (br s, 4H), 2.01 – 1.93 (m, 4H), 1.57 (br s, 14H), 1.42 – 1.37 (m, 2H). ^{13}C{1H} NMR (100 MHz, DMSO-d_6): δ 167.1, 162.9, 138.4, 128.8, 128.3, 123.8, 120.1, 119.5, 95.4, 76.4, 56.9, 56.6, 51.8, 33.6, 29.5, 29.2, 27.1, 25.5, 24.6, 23.4, 22.7, 22.4. HRMS (ESI) *m/z*: $[M + H]^+$ Calcd for $C_{24}H_{32}N_3OS^+$ 410.2261; Found 410.2266.

3-(Azepan-1-yl)-4-cyano-N-phenyl-2-thiaspiro[4.7]dodec-3-ene-1-carboxamide (***5f***). Dihydrothiophene **4f** (74 mg, 1.0 equiv, 0.16 mmol), sodium (18 mg, 5.0 equiv, 0.80 mmol) and dry ethanol (2.5 mL). Yield of **5f** 89% (60 mg), mp 127–131 °C. ^{1}H NMR (400 MHz, DMSO-d_6): δ 10.03 (s, 1H), 7.55 (d, J = 7.6 Hz, 2H), 7.32 (t, J = 7.9 Hz, 2H), 7.08 (t, J = 7.4 Hz, 1H), 4.00 (s, 1H), 3.59 – 3.56 (m, 4H), 2.03 – 1.87 (m, 4H), 1.78 – 1.34 (m, 18H). ^{13}C{1H} NMR (100 MHz, DMSO-d_6): δ 167.1, 161.7, 138.5, 128.8, 123.8, 120.6, 119.5, 73.5, 57.3, 56.9, 53.3, 33.6, 29.7, 29.3, 28.1, 27.1, 26.0, 24.6, 22.8, 22.5. HRMS (ESI) *m/z*: $[M + H]^+$ Calcd for $C_{25}H_{34}N_3OS^+$ 424.2417; Found 424.2421.

The procedure for the derivatization of dihydrothiophene 4g

N-(1-(2-Acetyl-4-cyano-5-morpholino-3-phenyl-2,3-dihydrothiophen-2-yl)ethylidene)-4-methylbenzenesulfonamide (**4g**, 160 mg, 1.0 equiv, 0.31 mmol) was subjected to column chromatography on neutral alumina (eluent EtOAc:PE, gradient up to 3:2) to afford 5-acetyl-2-morpholino-4-phenyl-4,5-dihydrothiophene-3-carbonitrile (**5g**) as a yellow powder. Yield of **5g** 66% (65 mg), mp 141–142 °C. ^{1}H NMR (400 MHz, $CDCl_3$-*d*): δ 7.38–7.28 (m, 5H), 4.73 (d, J = 3.5 Hz, 1H), 4.05 (d, J = 3.5 Hz, 1H), 3.77–3.73 (m, 4H), 3.68–3.62 (m, 4H), 2.27 (s, 3H). ^{13}C{1H} NMR (100 MHz, $CDCl_3$-*d*): δ 201.1, 161.9, 140.6, 129.2, 128.1, 127.2, 118.7, 74.2, 66.3, 60.7, 53.9, 50.9, 27.5. HRMS (ESI) *m/z*: $[M + H]^+$ Calcd for $C_{17}H_{19}N_2O_2S^+$ 315.1162; Found 315.1165.

Additional mechanistic studies

The control experiment was performed for dihydrothiophene **1a** with the use of the deuterated water (Scheme S1). In the presence of D_2O O−H (N−H) deuteration product **9a'** was obtained.

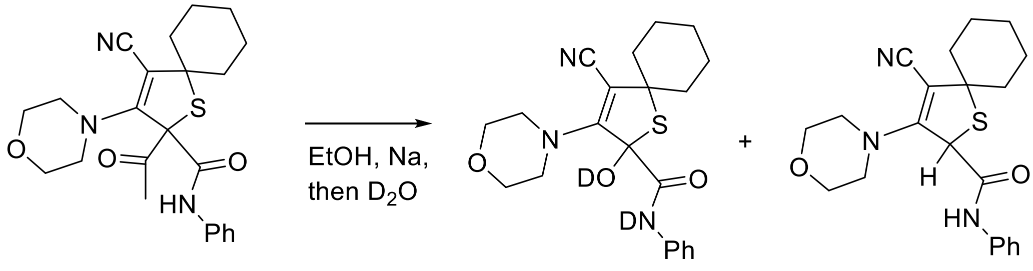

1a **2a**, 23% **3a**, 3%

Scheme S1. Conditions: dihydrothiophene **1a** (0.12 mmol, 5 equiv), sodium (0.59 mmol, 1 equiv), EtOH (2 mL), rt, 1 h, then D_2O (2.0 mL). Isolated yield after trituration and centrifugation in Et_2O.

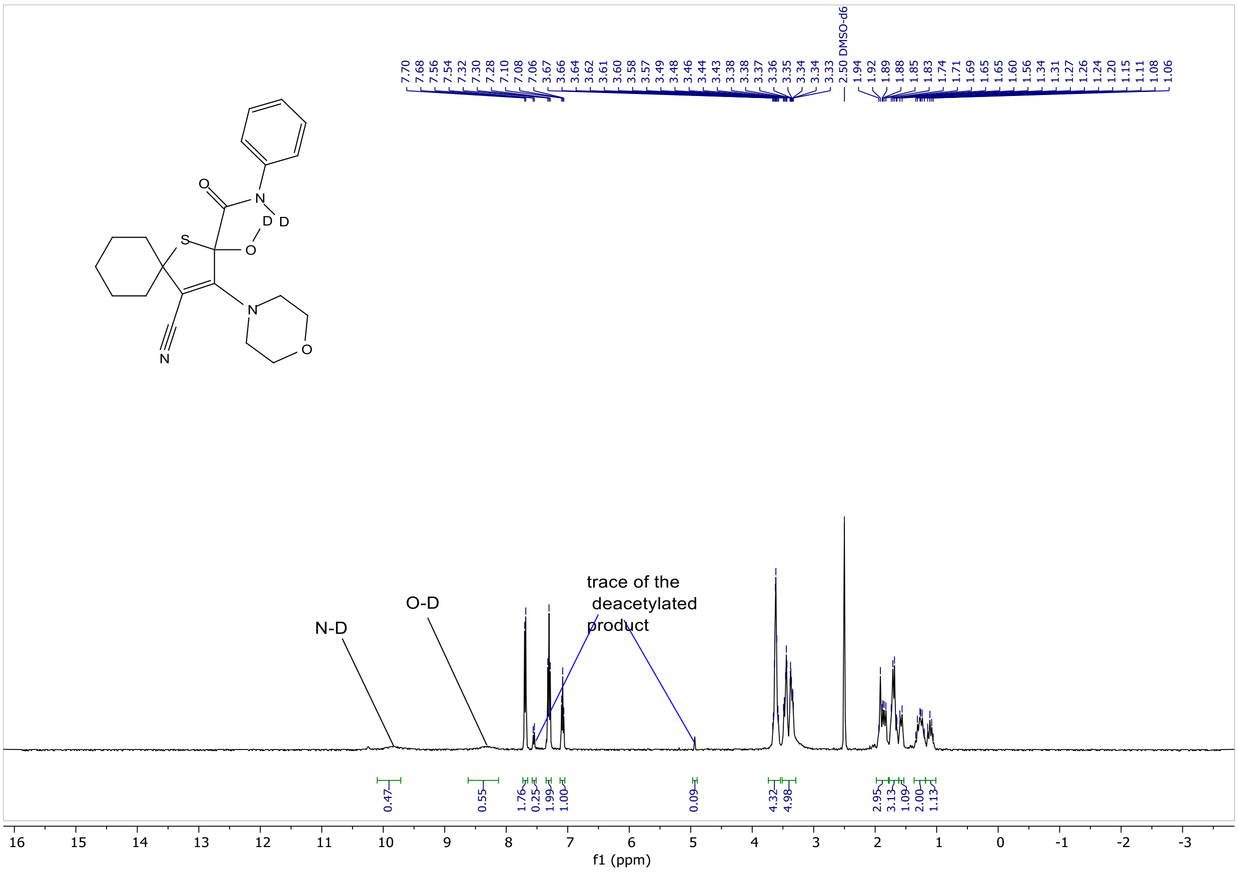

Figure S1. ^{1}H NMR spectrum of the deuterated product.

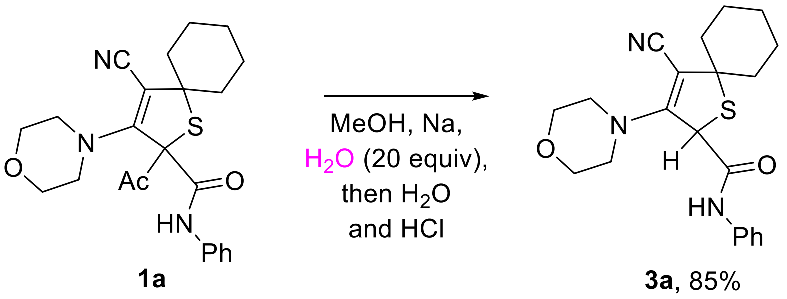

Scheme S2. Control experiment. Conditions: dihydrothiophene **1a** (0.12 mmol, 5 equiv), sodium (0.59 mmol, 1 equiv), MeOH (2 mL), H_2O (2.4 mmol, 20 equiv), rt, 1 h, then H_2O (2.0 mL), HCl (0.25 mL). Isolated yield after trituration and centrifugation in Et_2O.

References

1. Ilkin, V. G.; Filimonov, V. O.; Utepova, I. A.; Beryozkina, T. V.; Slepukhin, P. A.; Tumashov, A. A.; Dehaen, W.; Bakulev, V. A. *Org. Chem. Front.* **2024**, *11*, 3537−3545. doi:10.1039/D3QO02025H

2. Ilkin, V. G.; Filimonov, V. O.; Seliverstova, E. A.; Novikov, M.S.; Beryozkina, T. V.; Gagarin, A. A.; Belskaya, N. P.; Muthipeedika, N. J.; Bakulev, V. A; Dehaen, W. *J. Org. Chem.* **2022**, *87*, 12196–12213. doi:10.1021/acs.joc.2c01352

3. Wang, Z.; Bi, X.; Liao, P.; Zhang, R.; Lianga, Y.; Dong, D. *Chem. Comm.* **2012**, *4*8, 7076–7078. doi:10.1039/C2CC33157H
