# Supplementary material for: Base-promoted deacylation of 2-acetyl-2,5-dihydrothiophenes and their oxygen-mediated hydroxylation
Source: Beilstein J Org Chem. 2026 Jan 28;22:192–204. doi: 10.3762/bjoc.22.13 (PMC12862606; doi:10.3762/bjoc.22.13)
Supplement: File 2 — Copies of NMR spectra of all new compounds. [file Beilstein_J_Org_Chem-22-192-s002.pdf]

BEILSTEIN JOURNAL OF ORGANIC CHEMISTRY

Supporting Information

for

Base-promoted deacylation of 2-acetyl-2,5-dihydrothiophenes and their oxygen-mediated hydroxylation

Vladimir G. Ilkin, Margarita Likhacheva, Igor V. Trushkov, Tetyana V. Beryozkina, Vera S. Berseneva, Vladimir T. Abaev, Wim Dehaen and Vasiliy A. Bakulev

Beilstein J. Org. Chem. **2026,** *22,* 192–204. doi:10.3762/bjoc.22.13

Copies of NMR spectra of all new compounds

License and Terms: This is a supporting information file under the terms of the Creative Commons Attribution License (https://creativecommons.org/licenses/by/4.0). Please note that the reuse, redistribution and reproduction in particular requires that the author(s) and source are credited and that individual graphics may be subject to special legal provisions.
The license is subject to the *Beilstein Journal of Organic Chemistry* terms and conditions: (https://www.beilstein-journals.org/bjoc/terms)

Table of contents

Copies of NMR spectra S3

Copies of NMR spectra

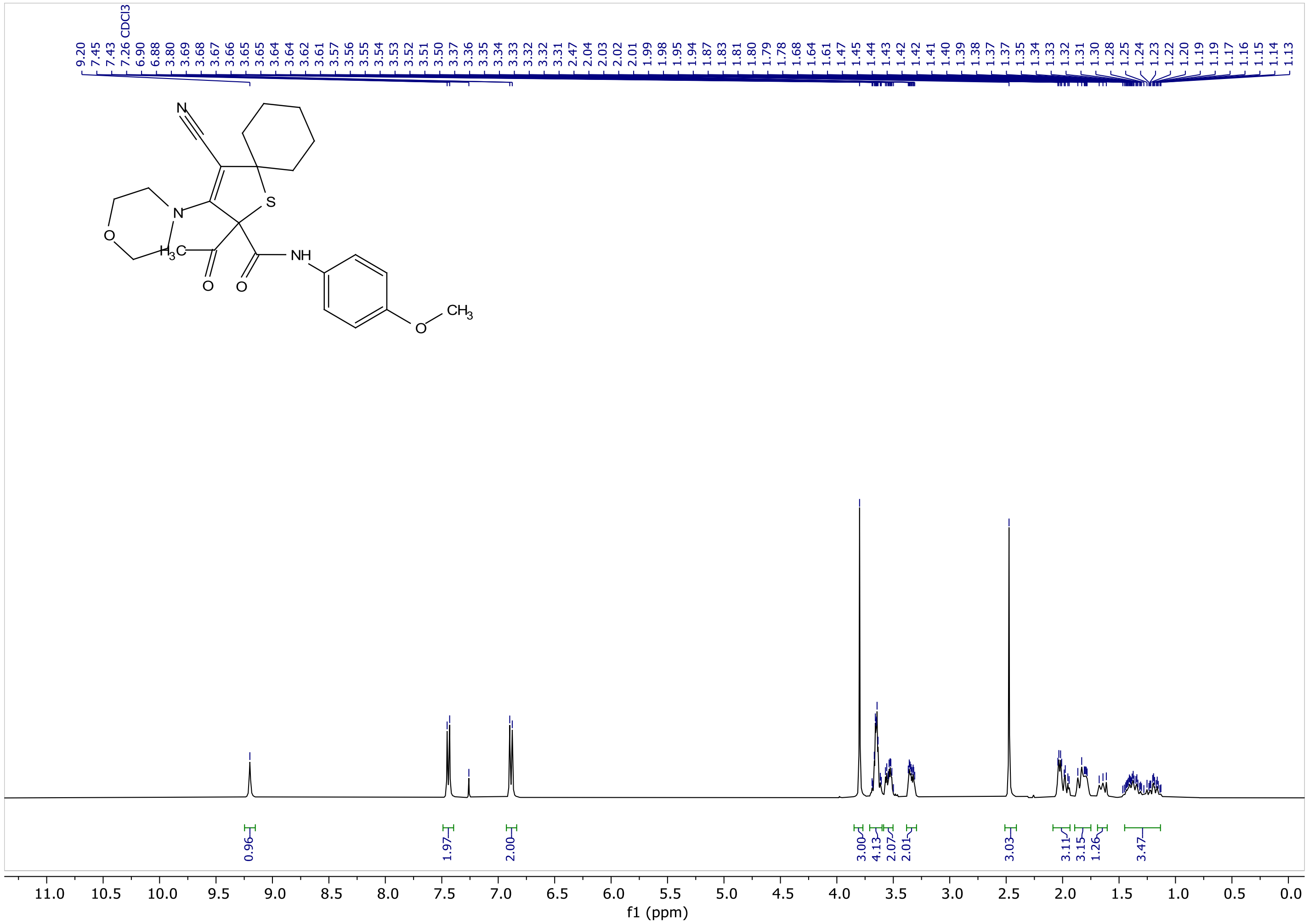

^1H NMR (400 MHz, $CDCl_3$-*d*) of **1b**

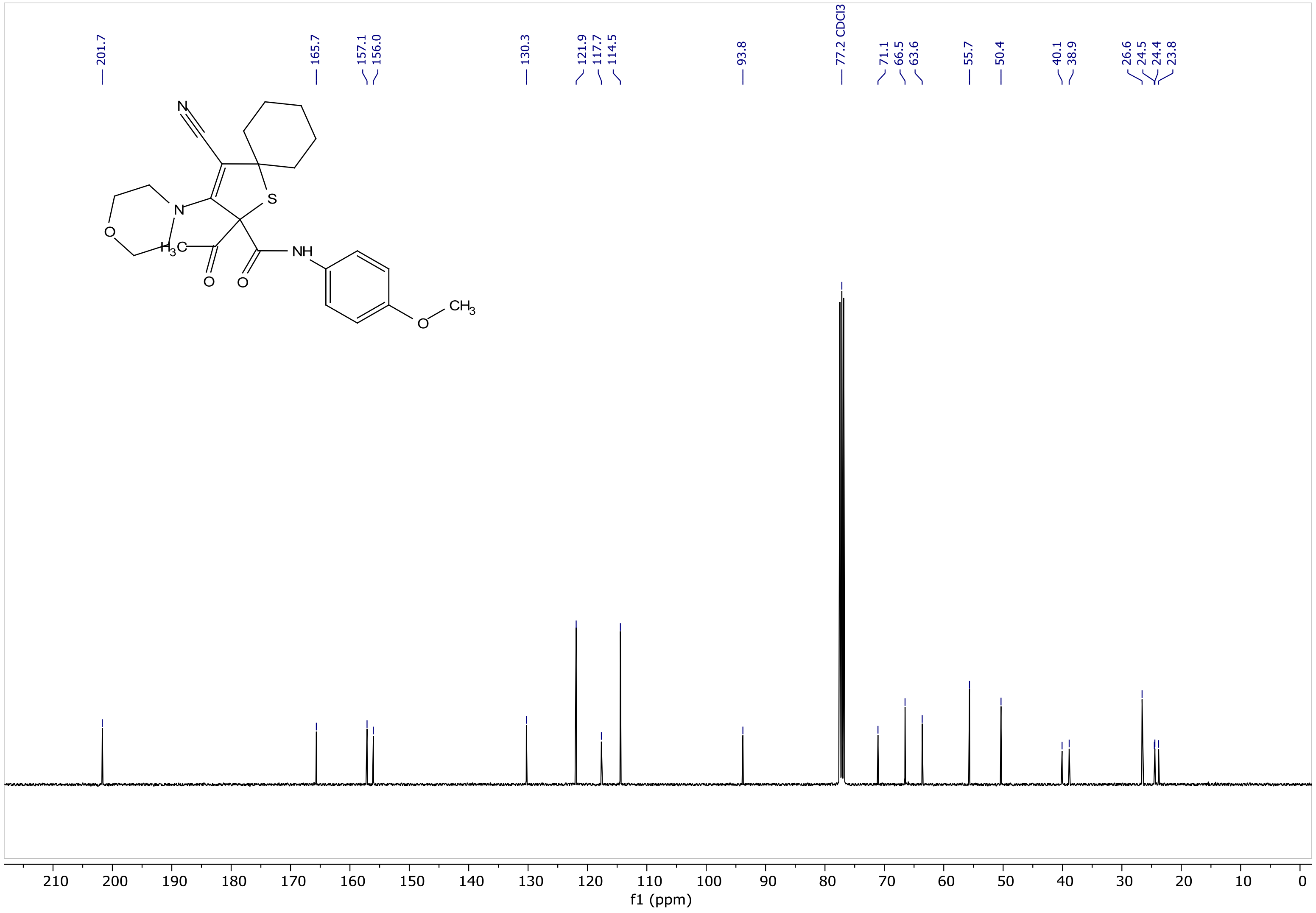

^{13}C NMR (100 MHz, $CDCl_3$-*d*) of **1b**

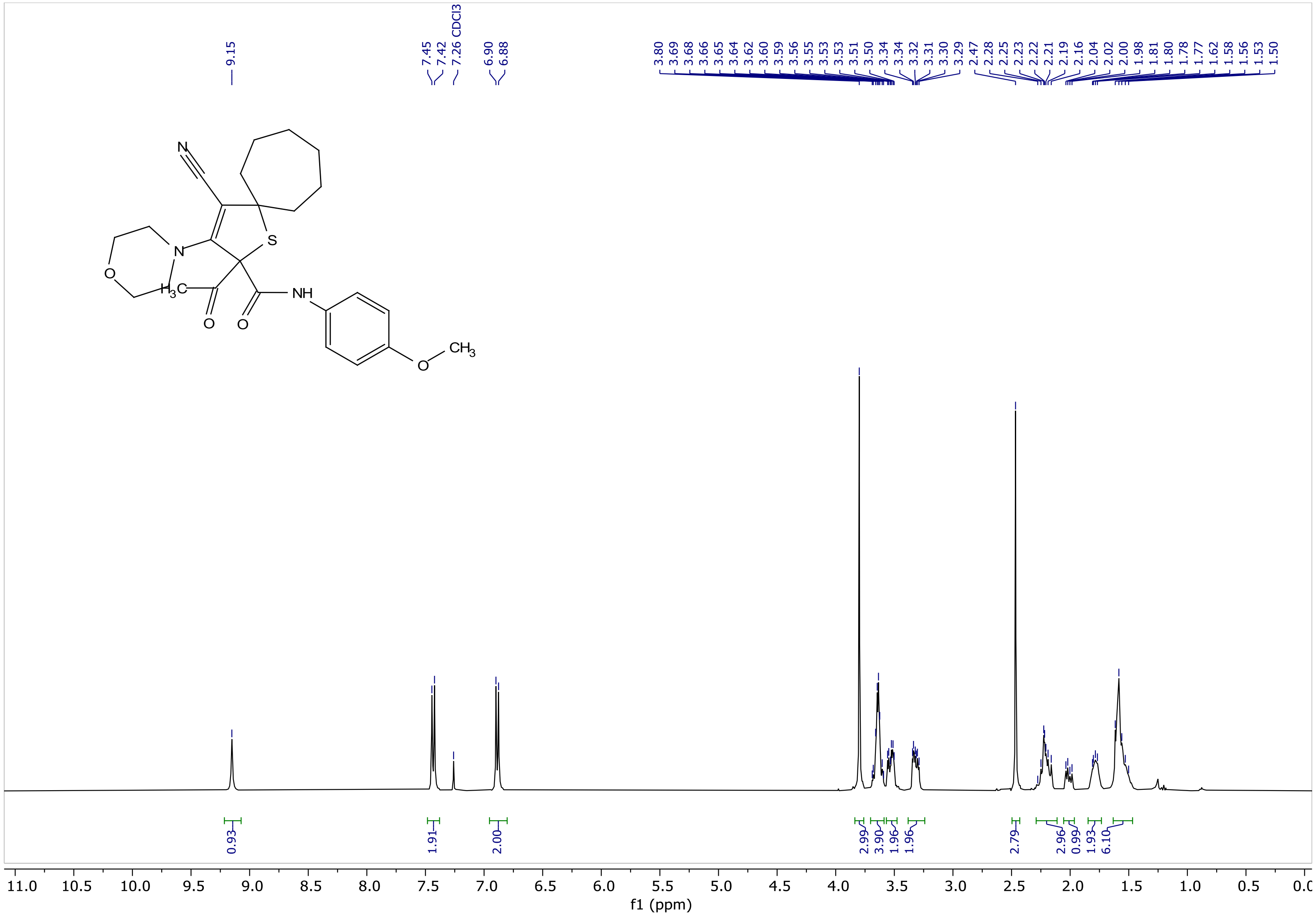

^{1}H NMR (400 MHz, $CDCl_3$-*d*) of **1d**

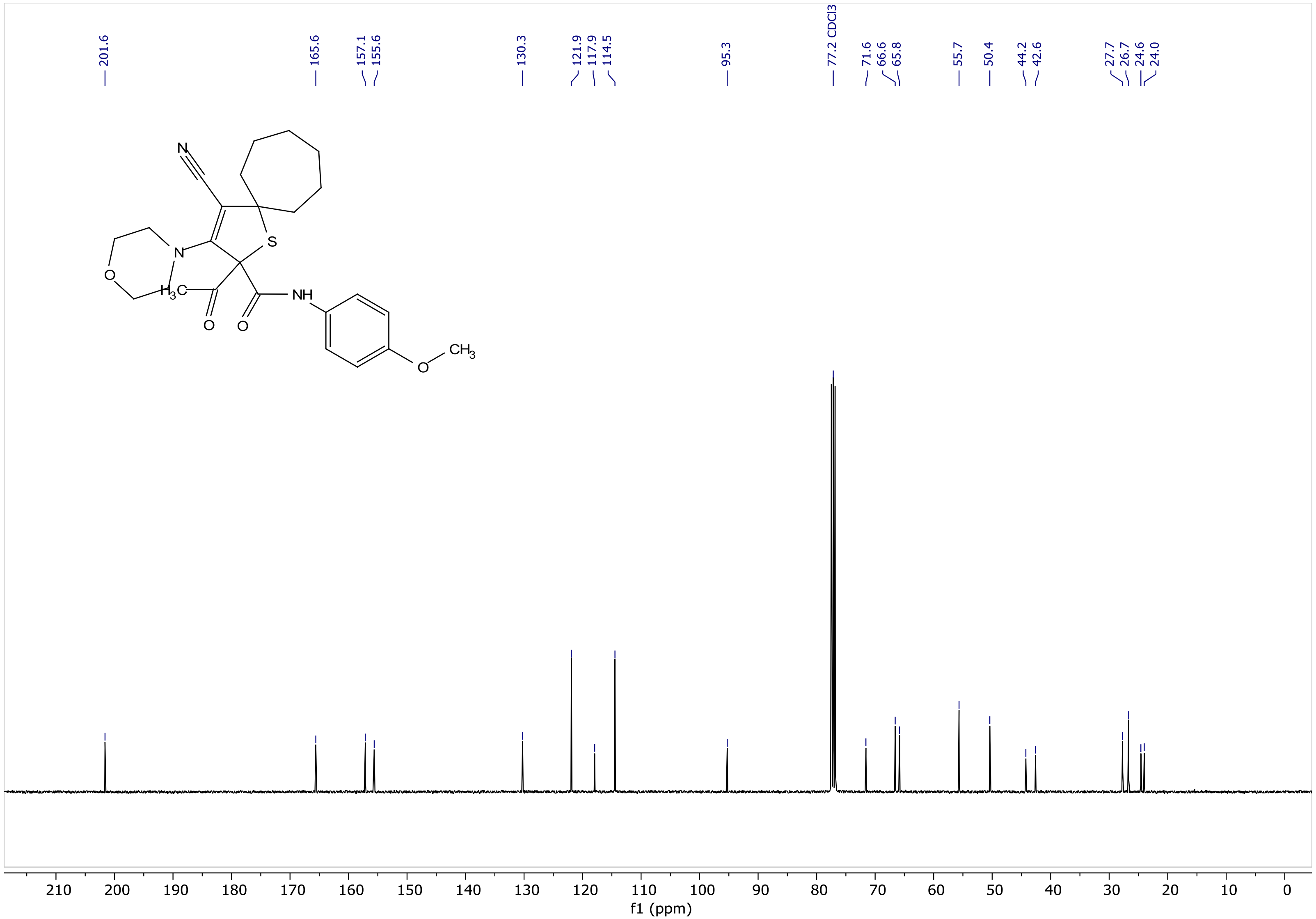

^{13}C NMR (100 MHz, $CDCl_3$-*d*) of **1d**

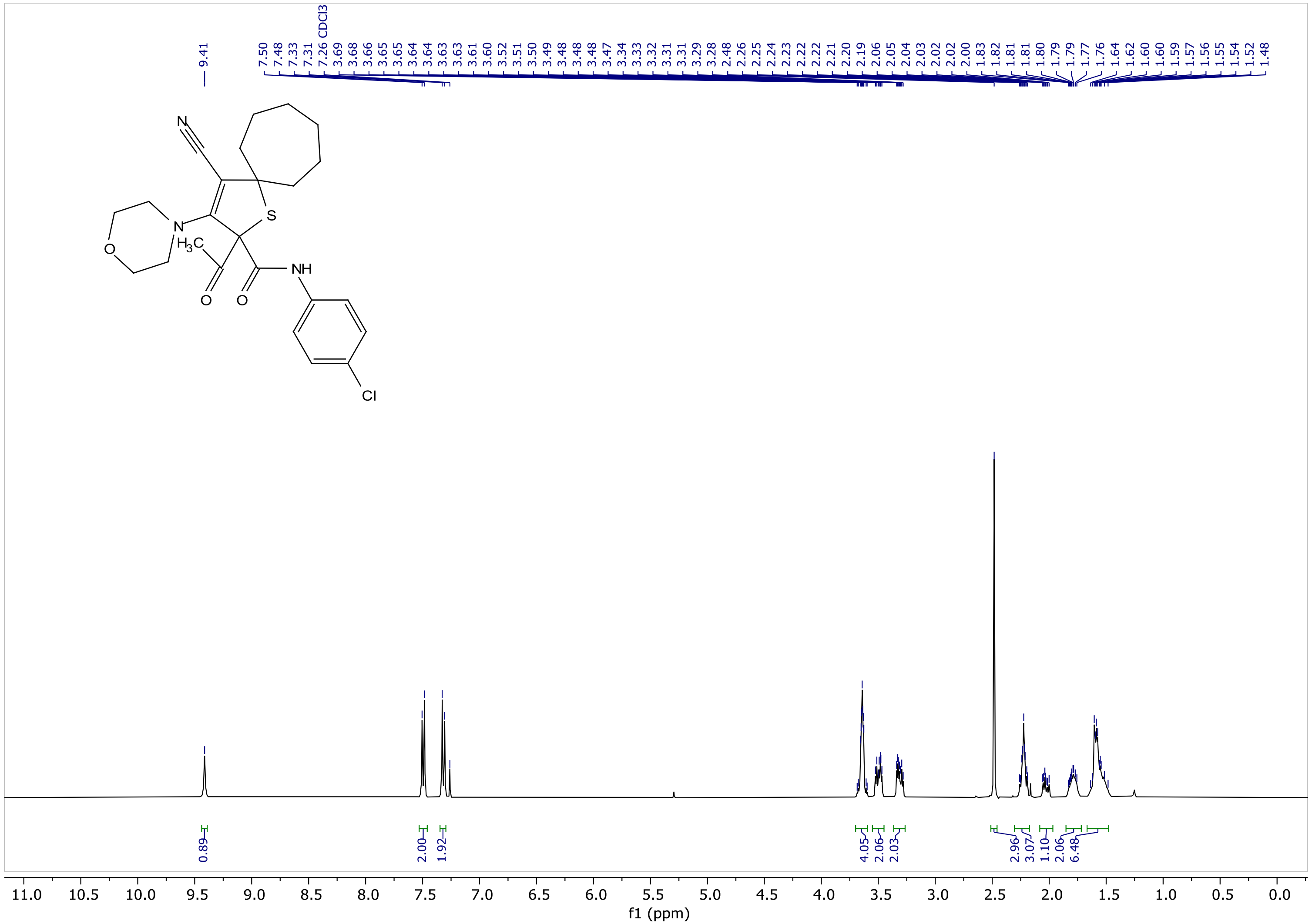

^{1}H NMR (400 MHz, $CDCl_3$-*d*) of **1e**

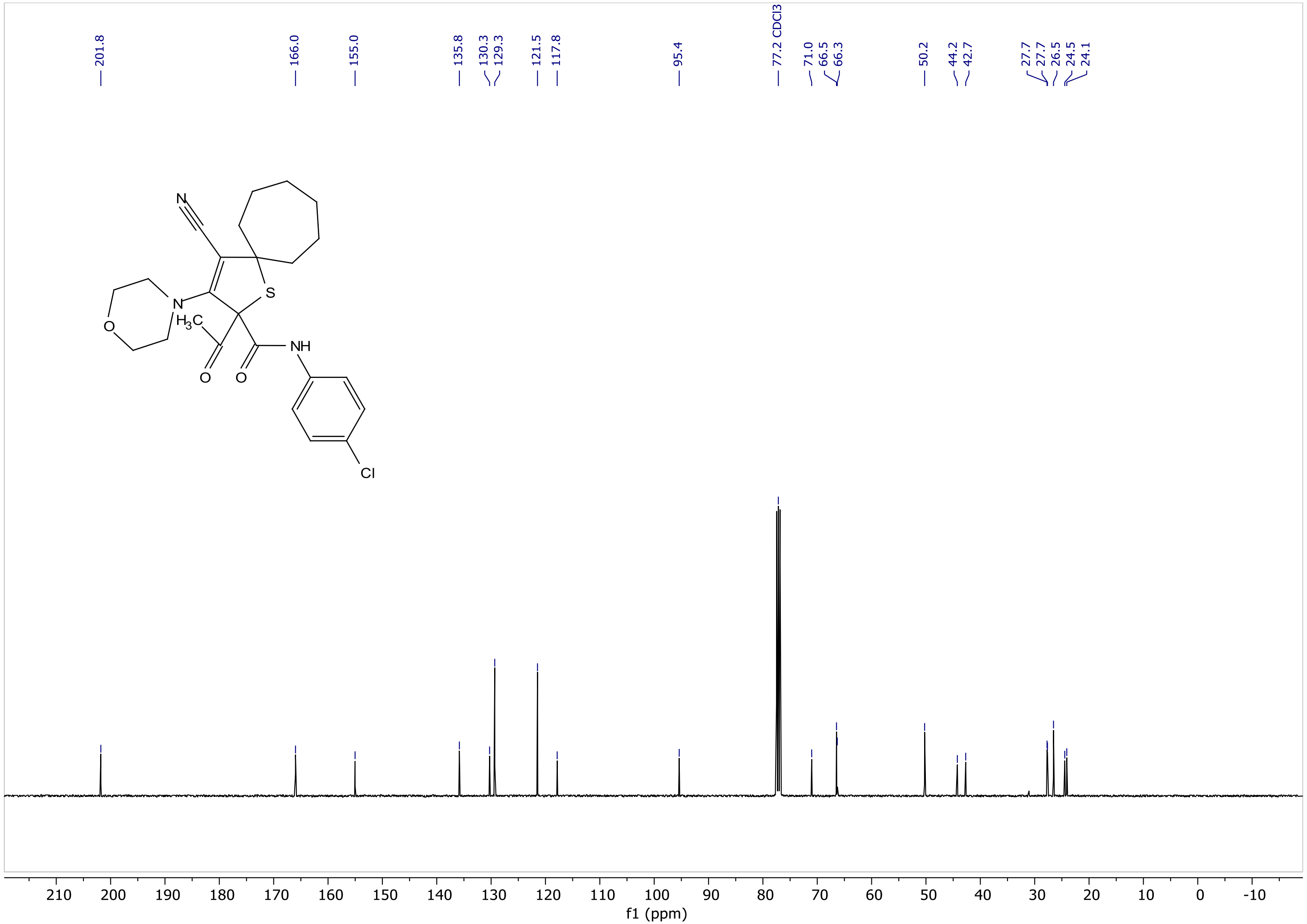

^{13}C NMR (100 MHz, $CDCl_3$-*d*) of **1e**

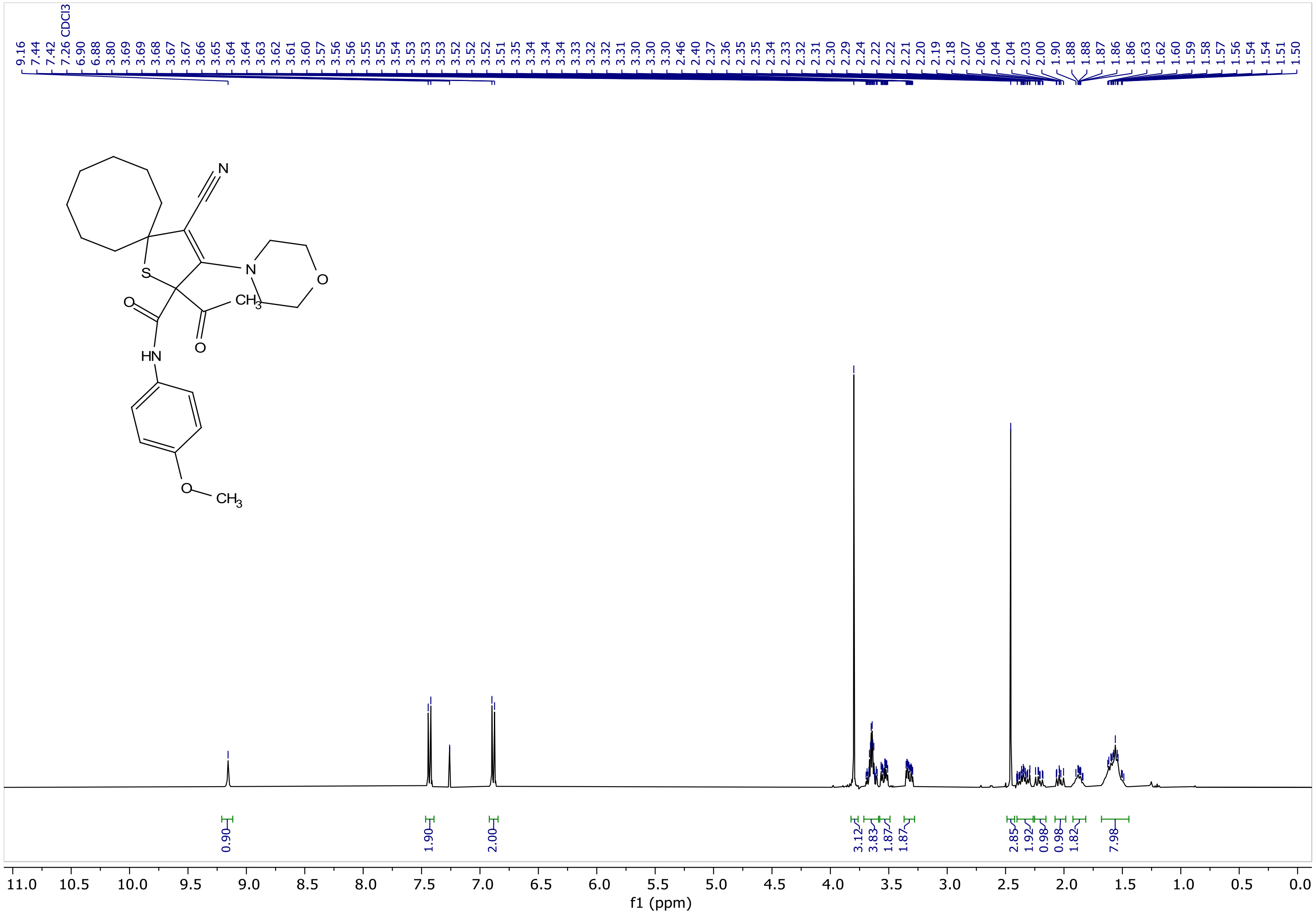

^{1}H NMR (400 MHz, $CDCl_3$-*d*) of **1g**

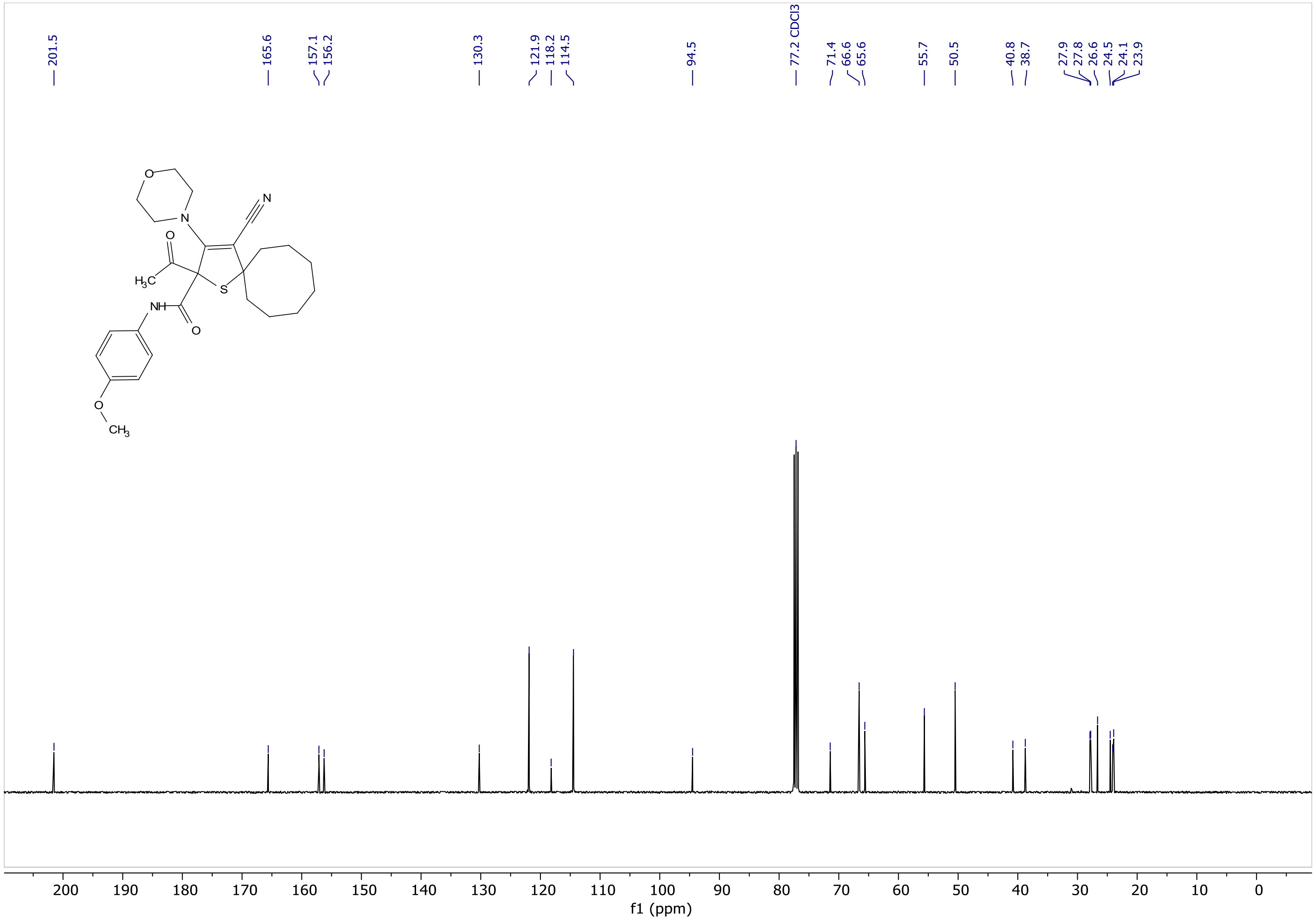

^{13}C NMR (100 MHz, $CDCl_3$-*d*) of **1g**

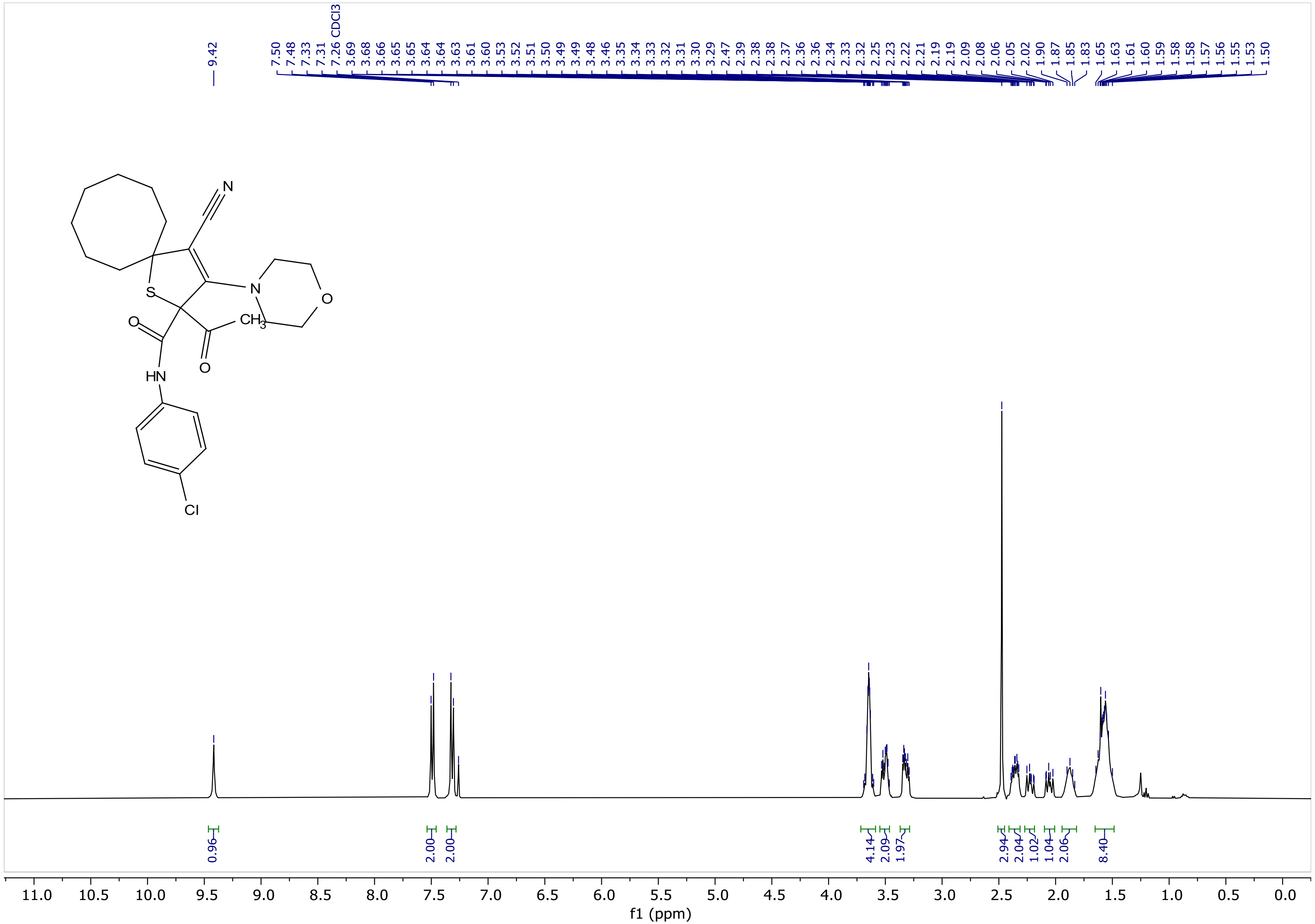

^{1}H NMR (400 MHz, $CDCl_3$-*d*) of **1h**

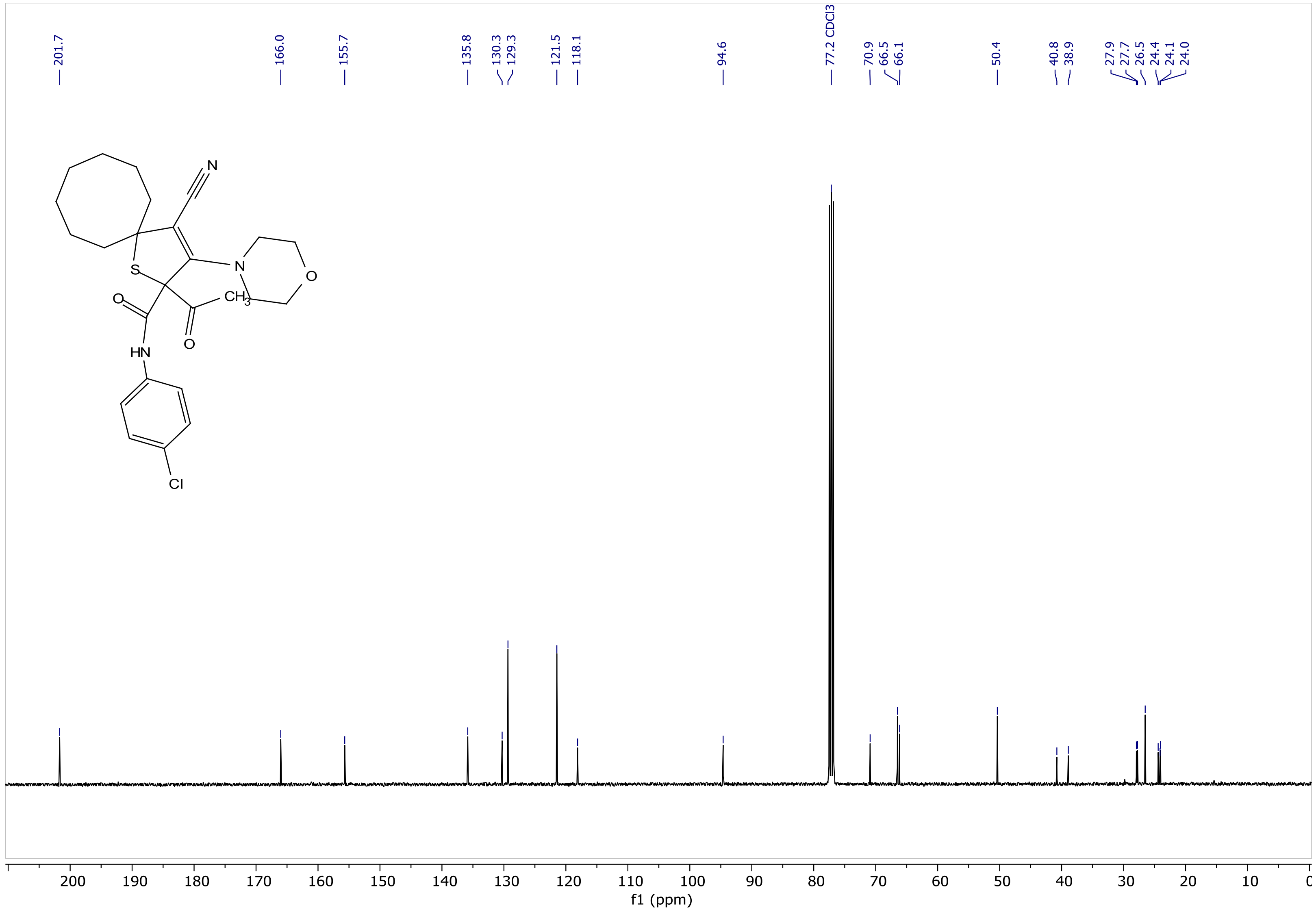

^{13}C NMR (100 MHz, $CDCl_3$-*d*) of **1h**

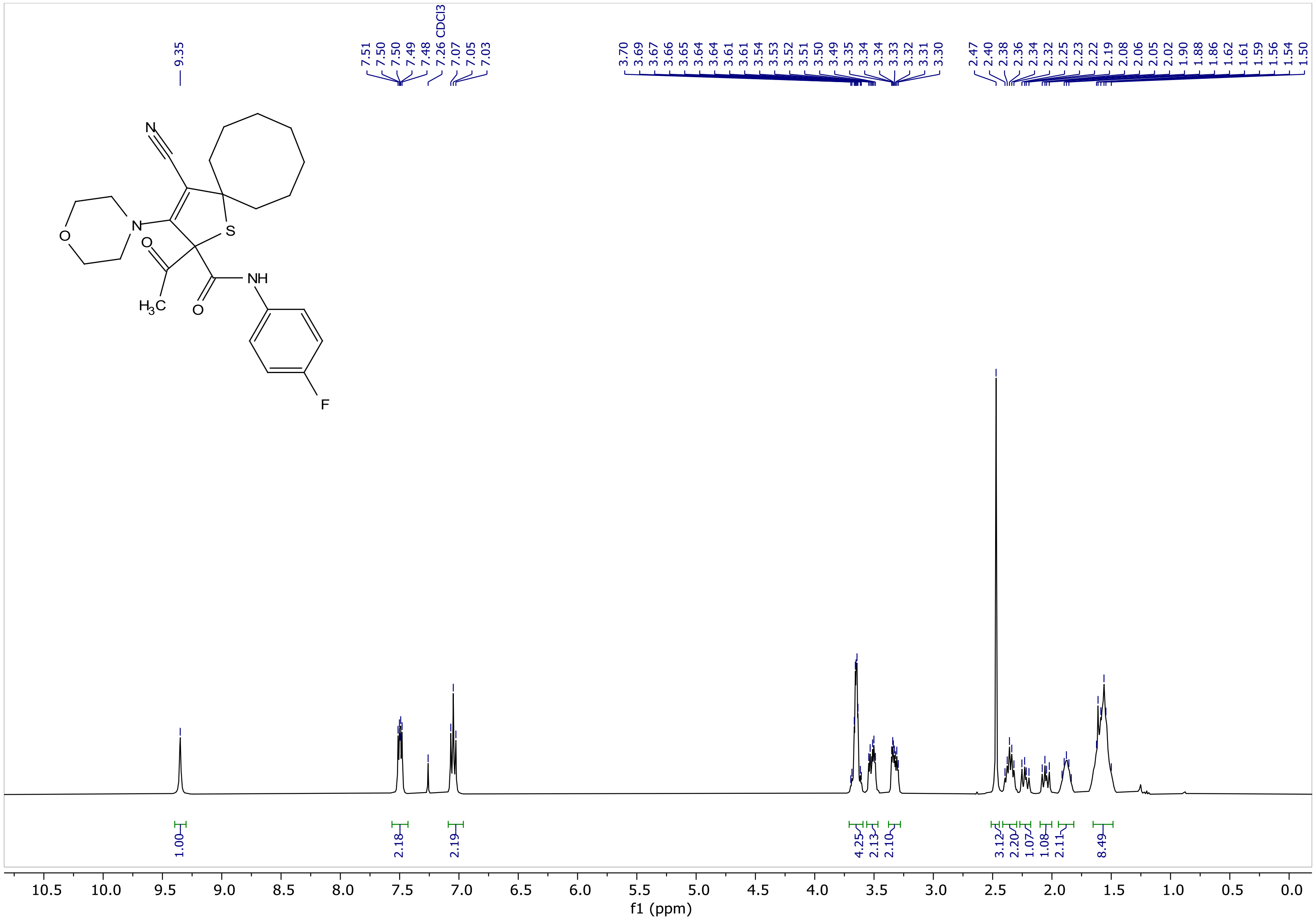

^{1}H NMR (400 MHz, CDCl$_3$-*d*) of **1i**

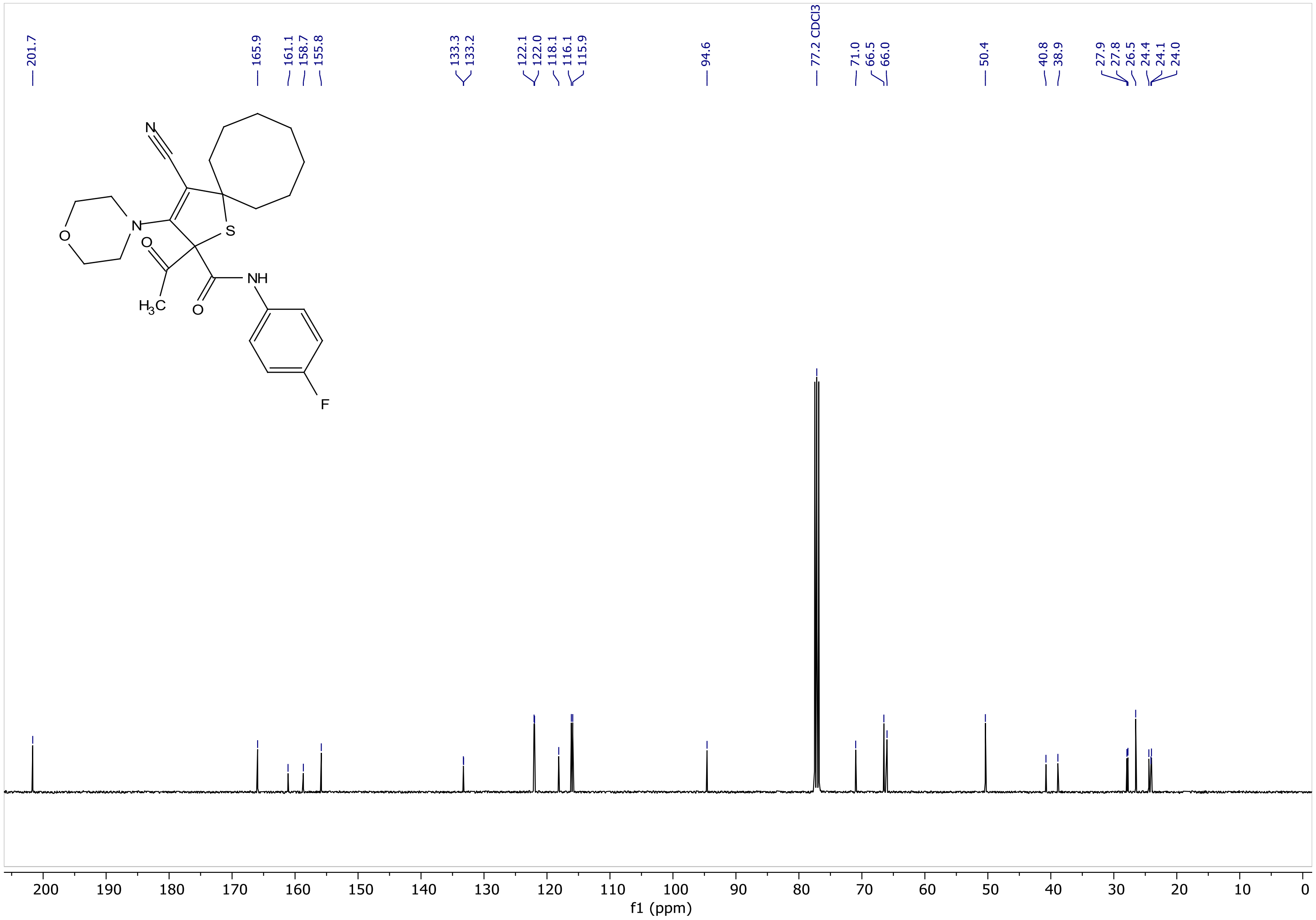

^{13}C NMR (100 MHz, $CDCl_3$-*d*) of **1i**

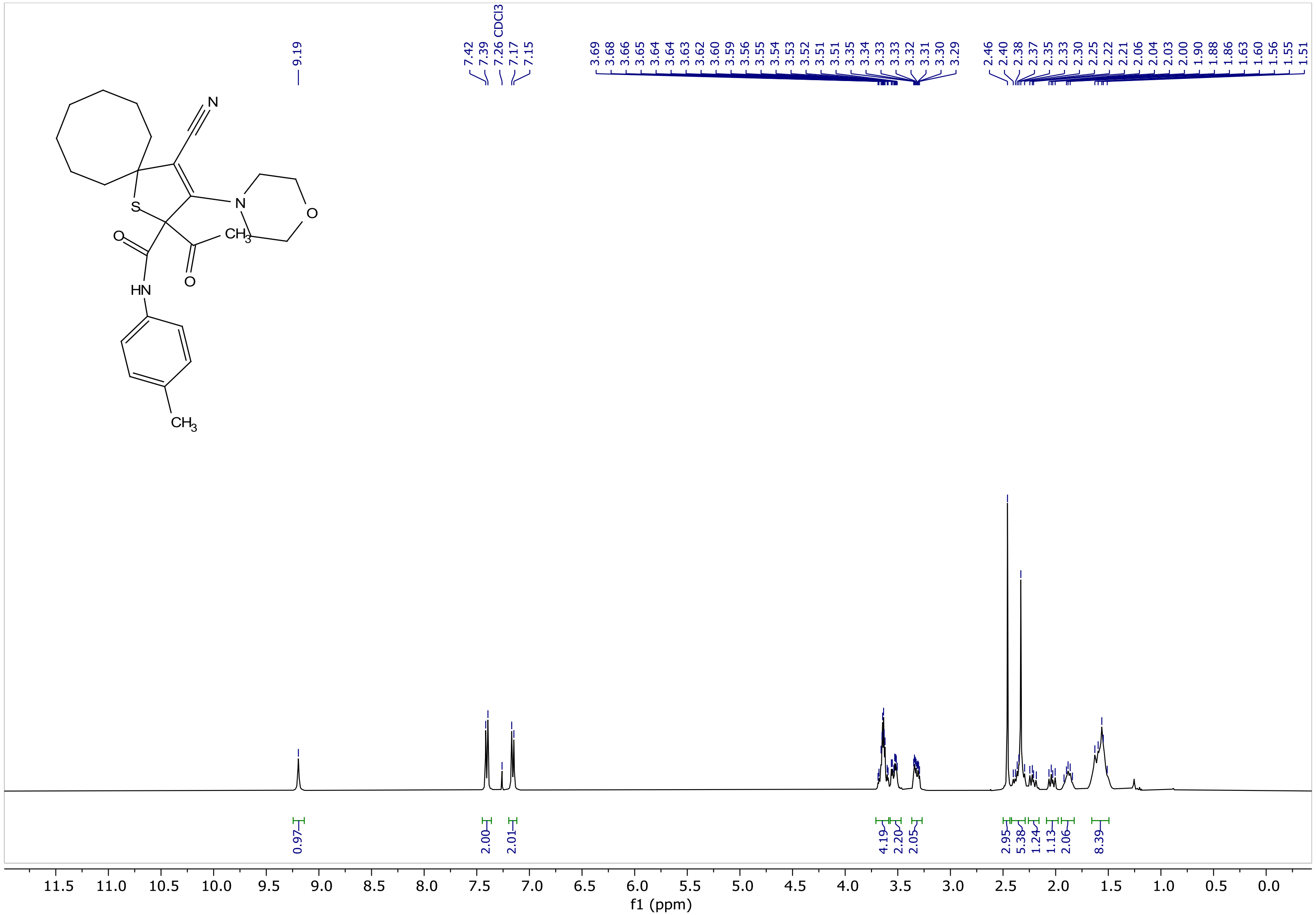

^{1}H NMR (400 MHz, $CDCl_3$-*d*) of **1j**

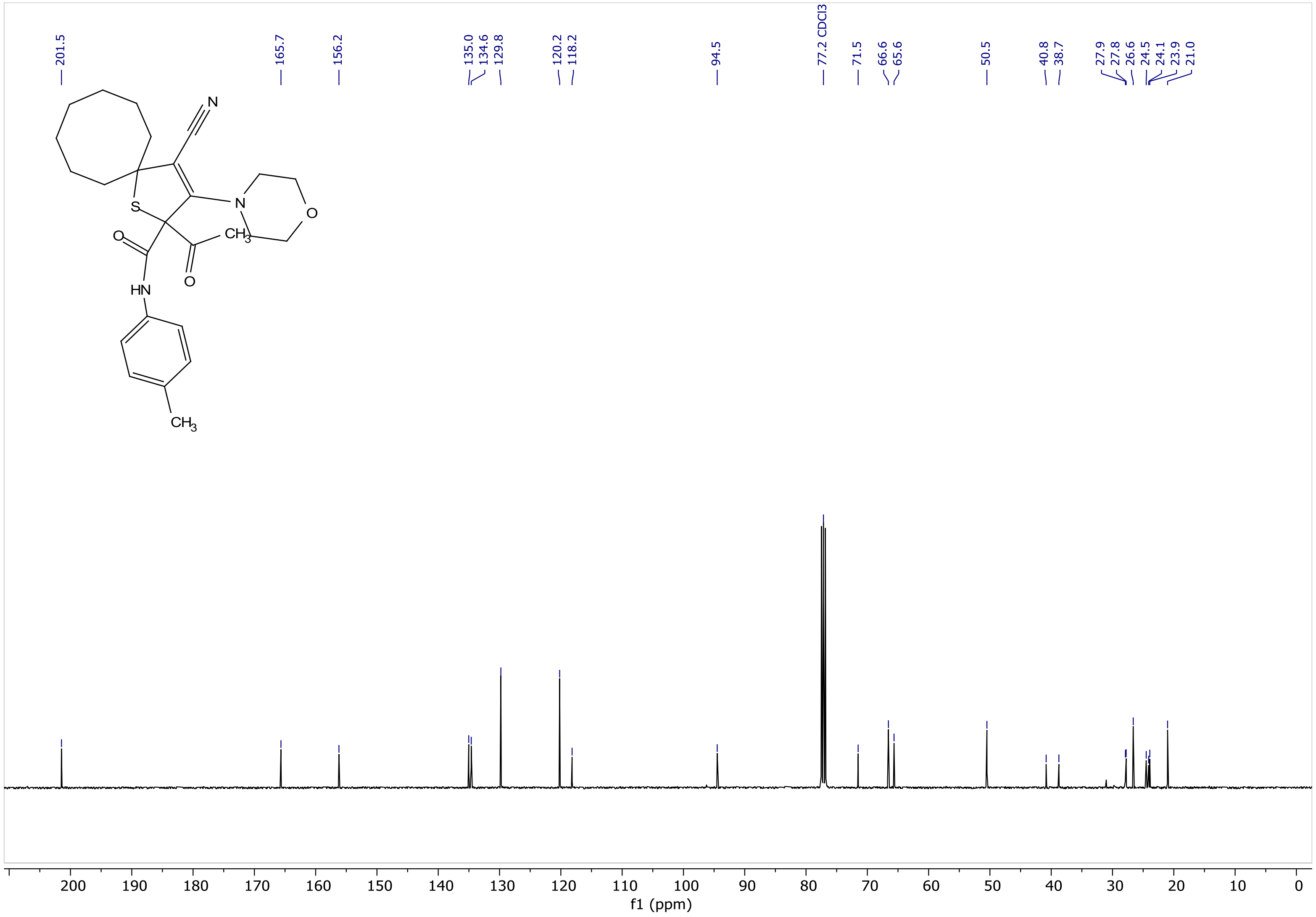

^{13}C NMR (100 MHz, $CDCl_3$-*d*) of **1j**

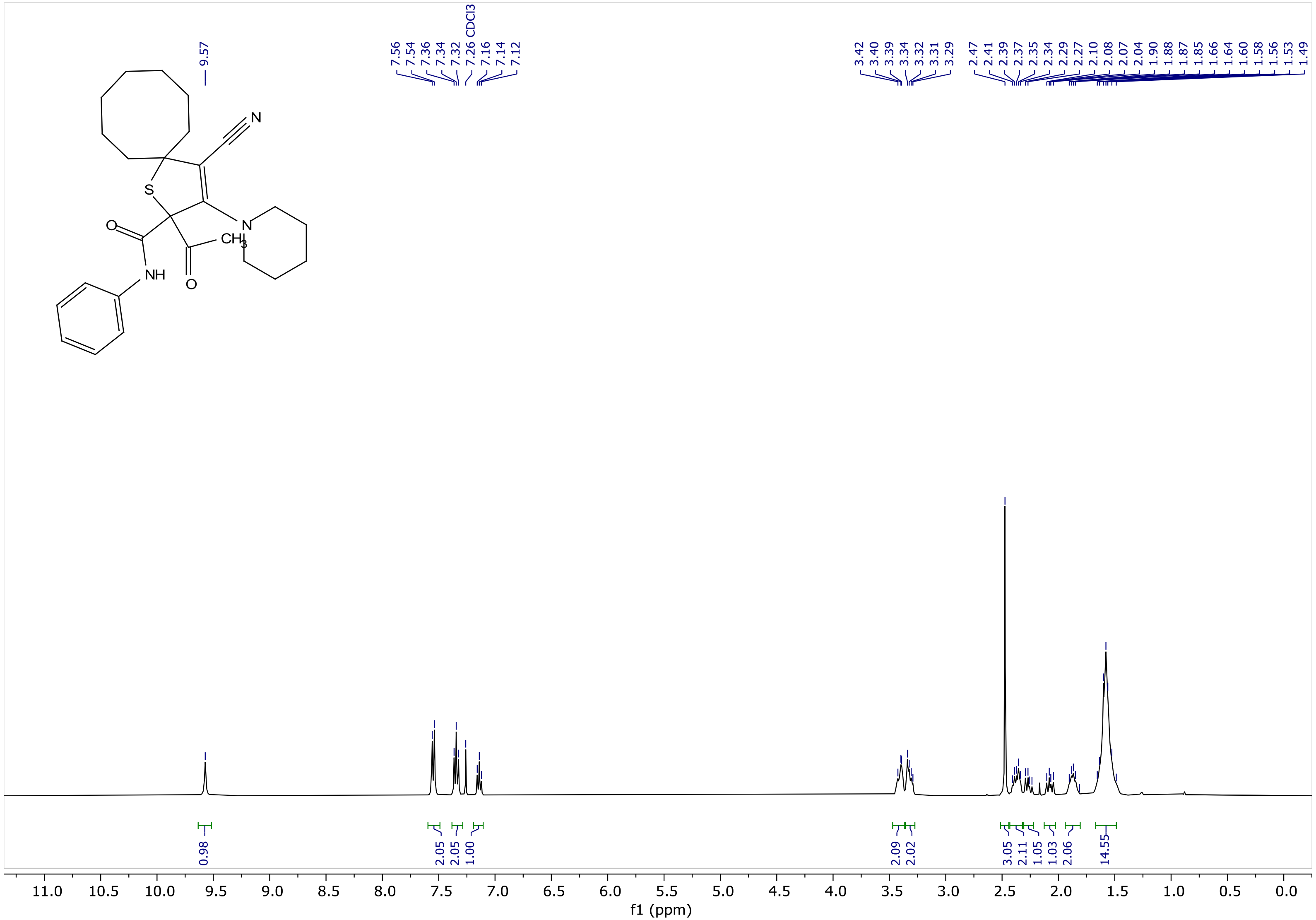

^{1}H NMR (400 MHz, $CDCl_3$-*d*) of **1k**

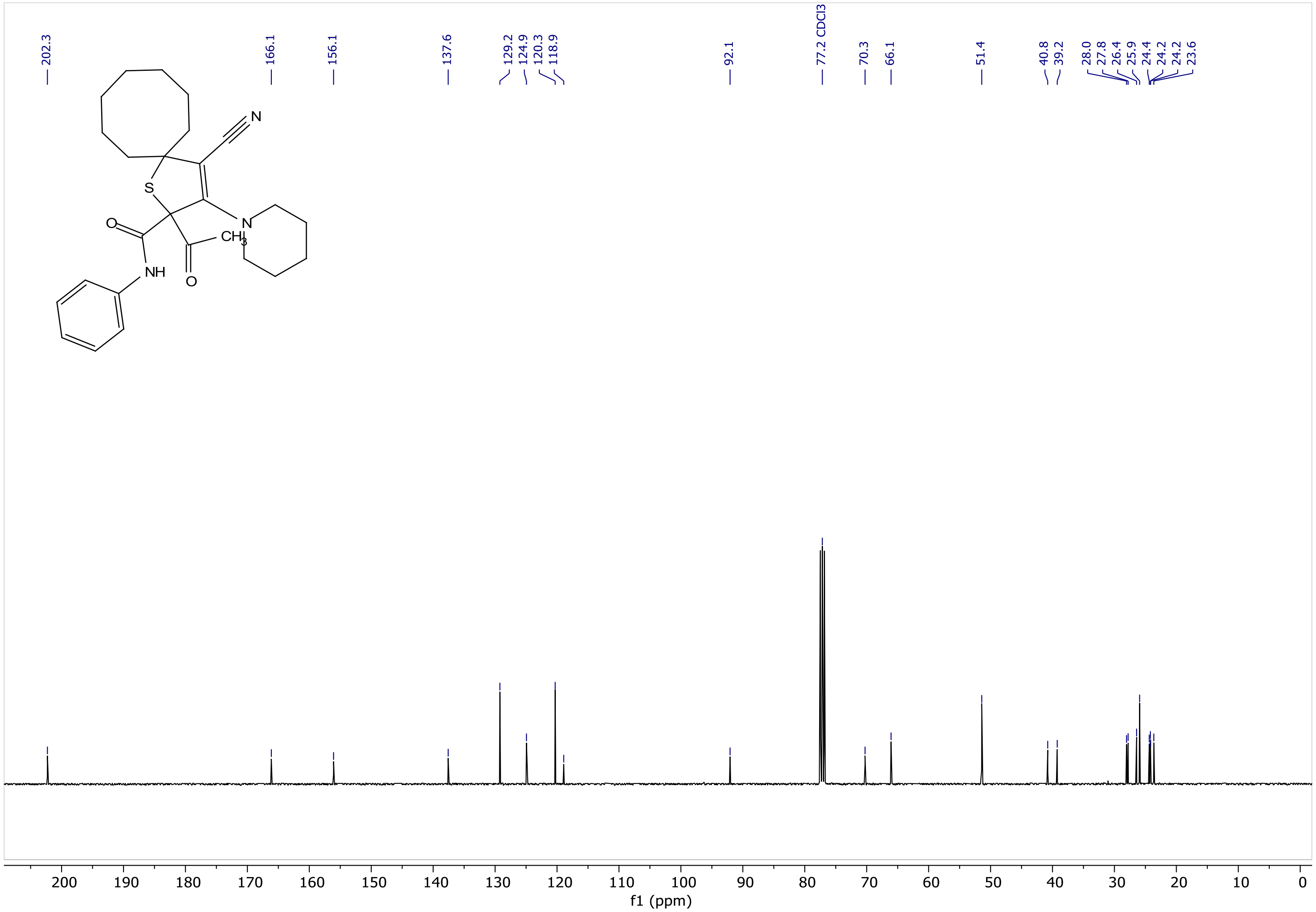

^{13}C NMR (100 MHz, $CDCl_3$-*d*) of **1k**

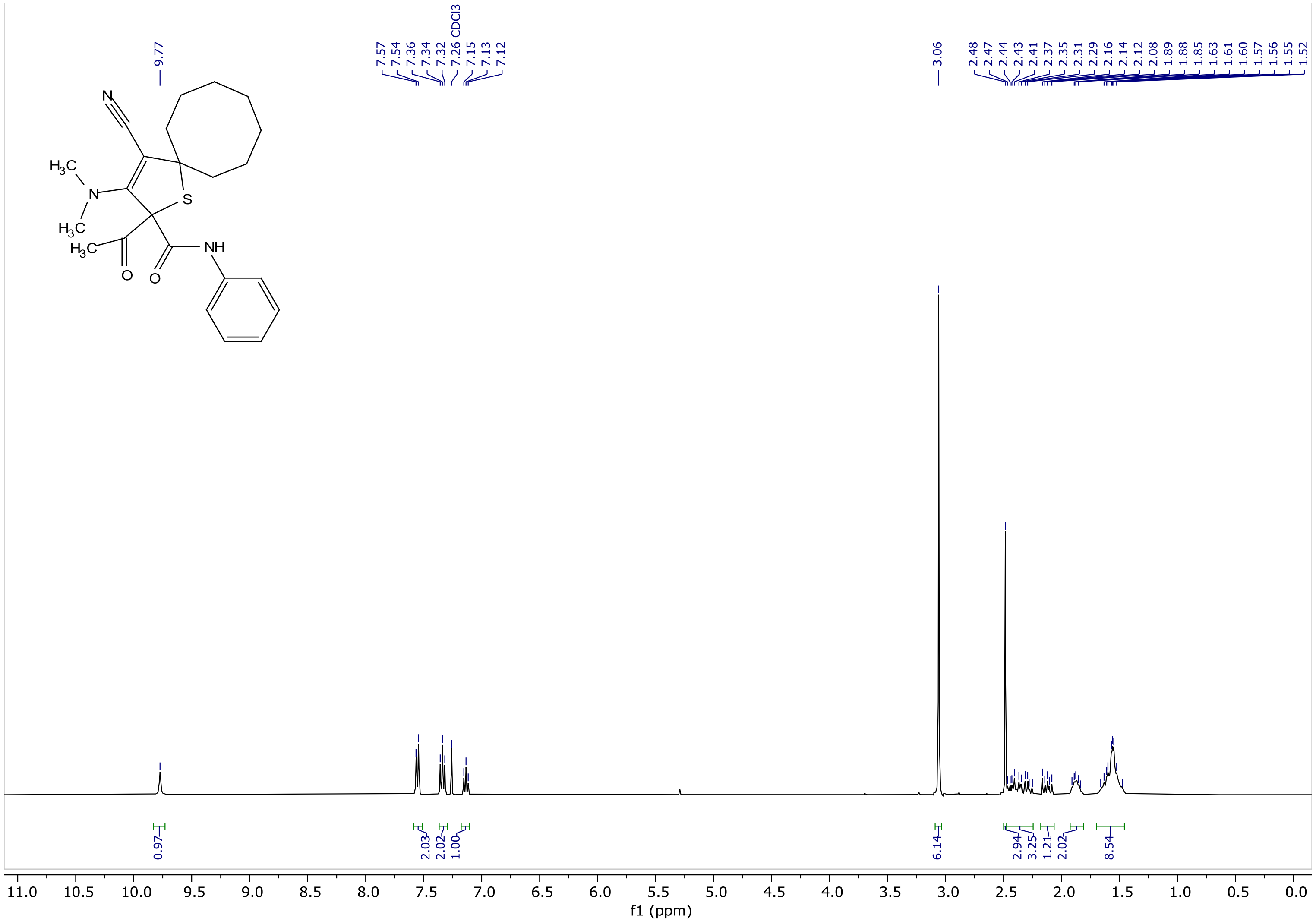

^{1}H NMR (400 MHz, $CDCl_3$-*d*) of **1l**

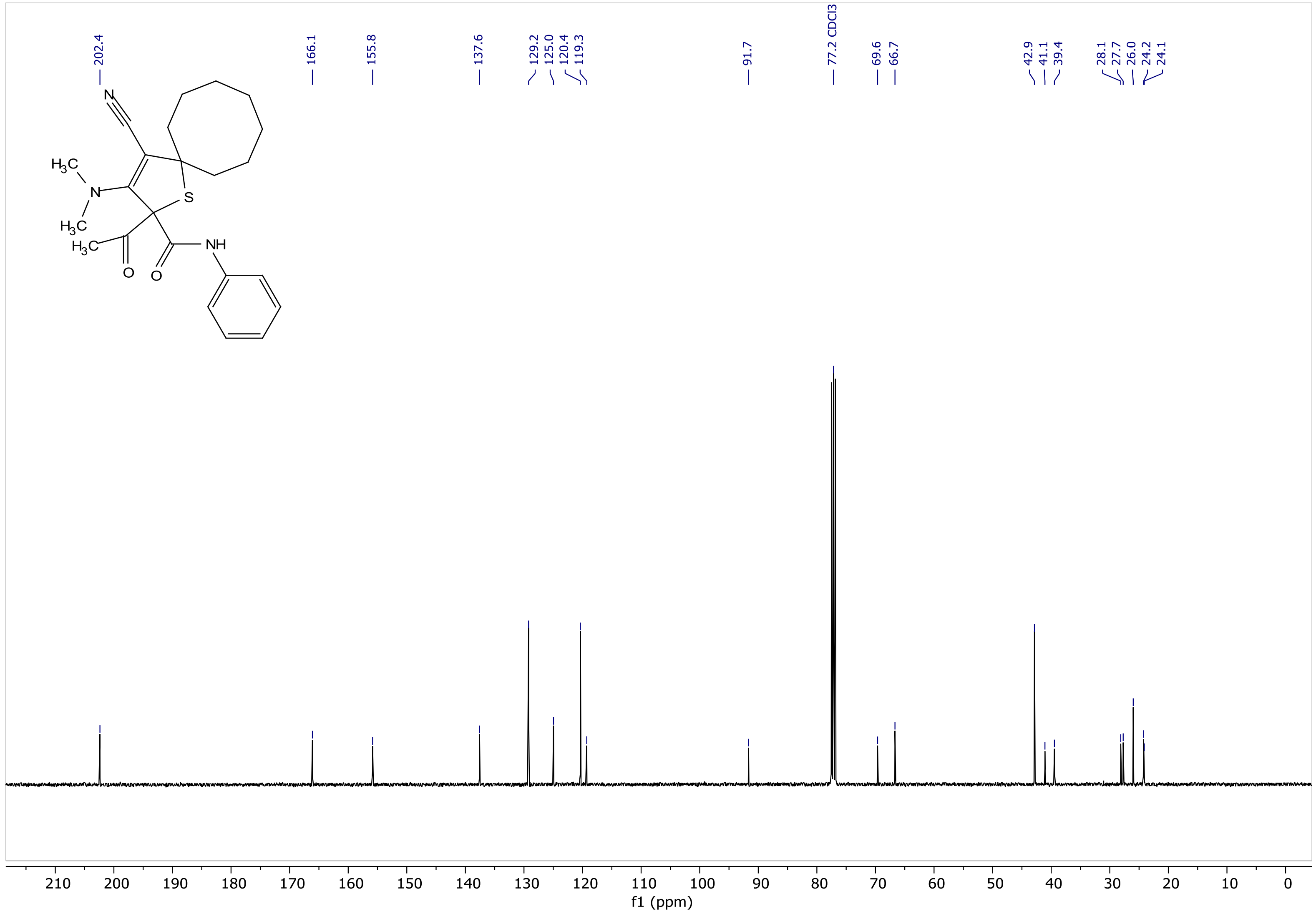

^{13}C NMR (100 MHz, $CDCl_3$-*d*) of **1l**

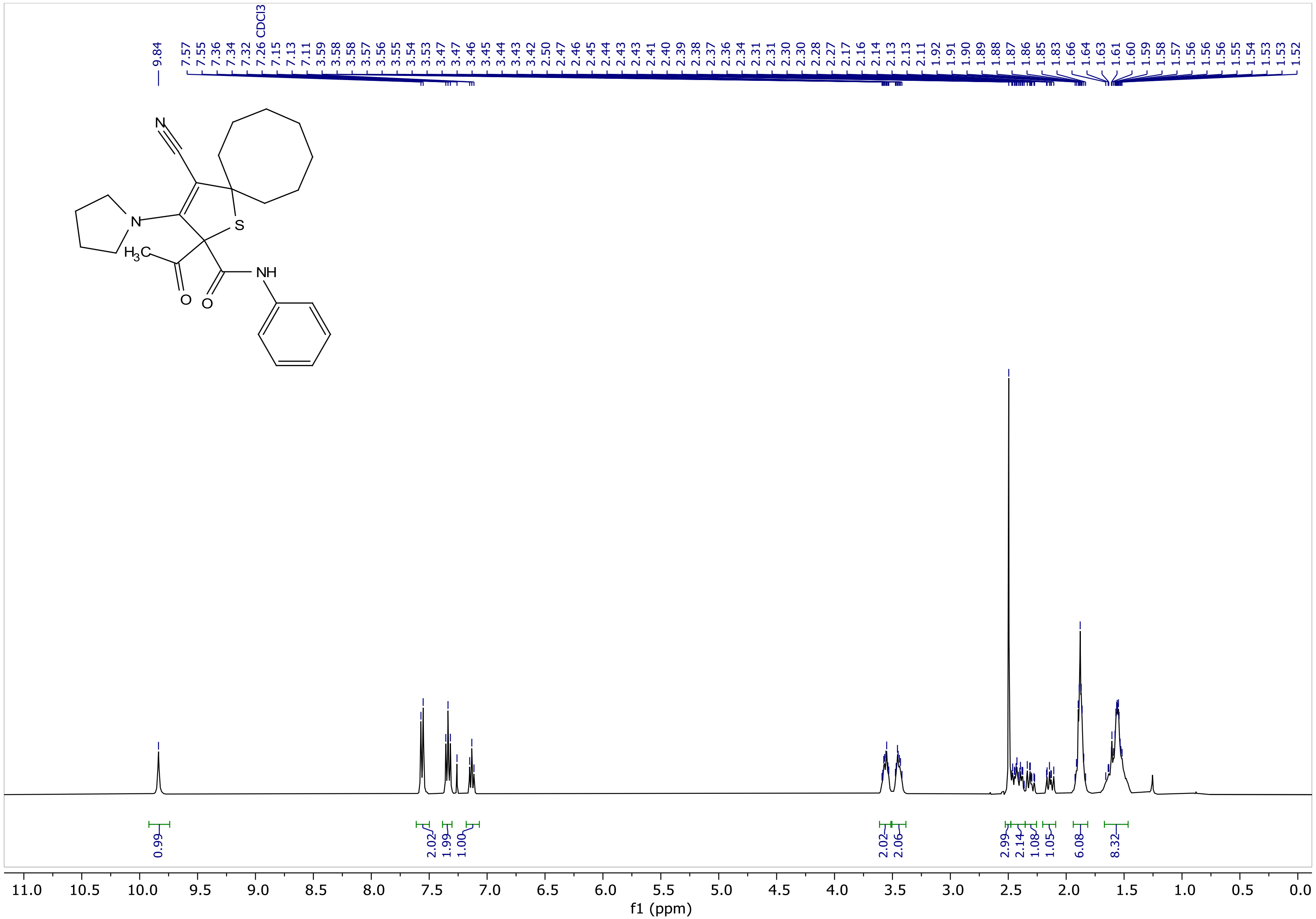

^{1}H NMR (400 MHz, $CDCl_3$-*d*) of **1m**

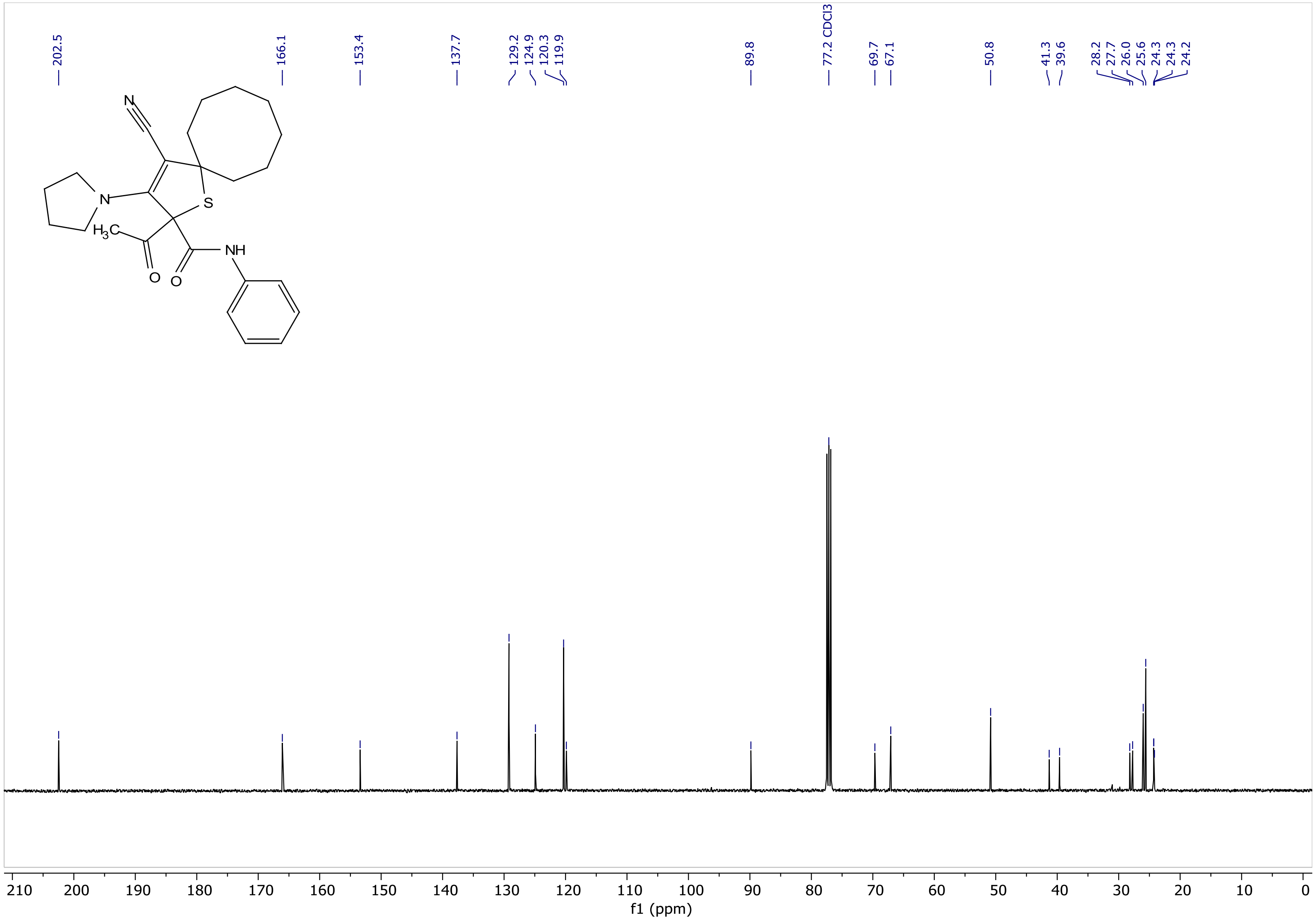

^{13}C NMR (100 MHz, $CDCl_3$-*d*) of **1m**

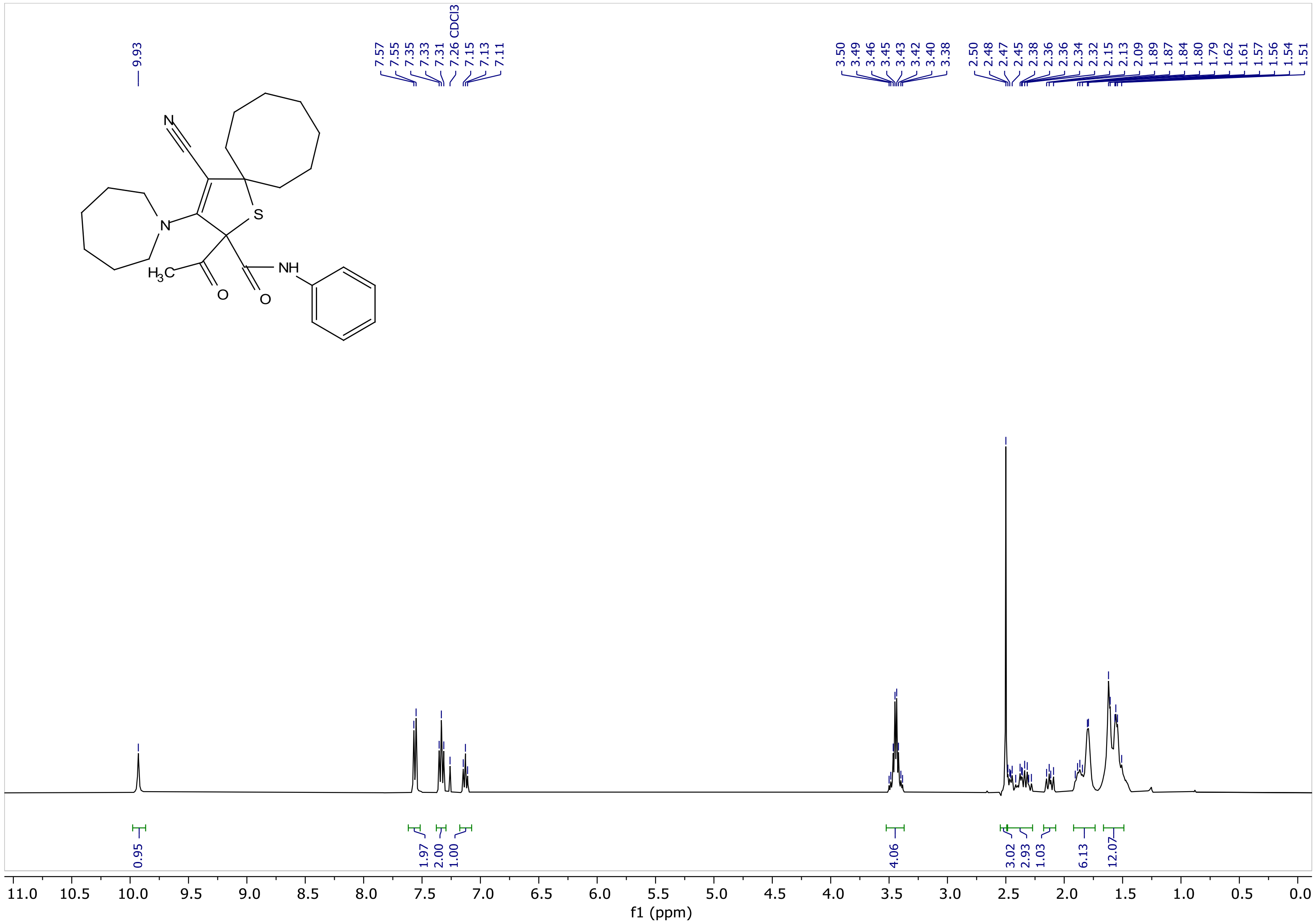

^{1}H NMR (400 MHz, $CDCl_3$-*d*) of **1n**

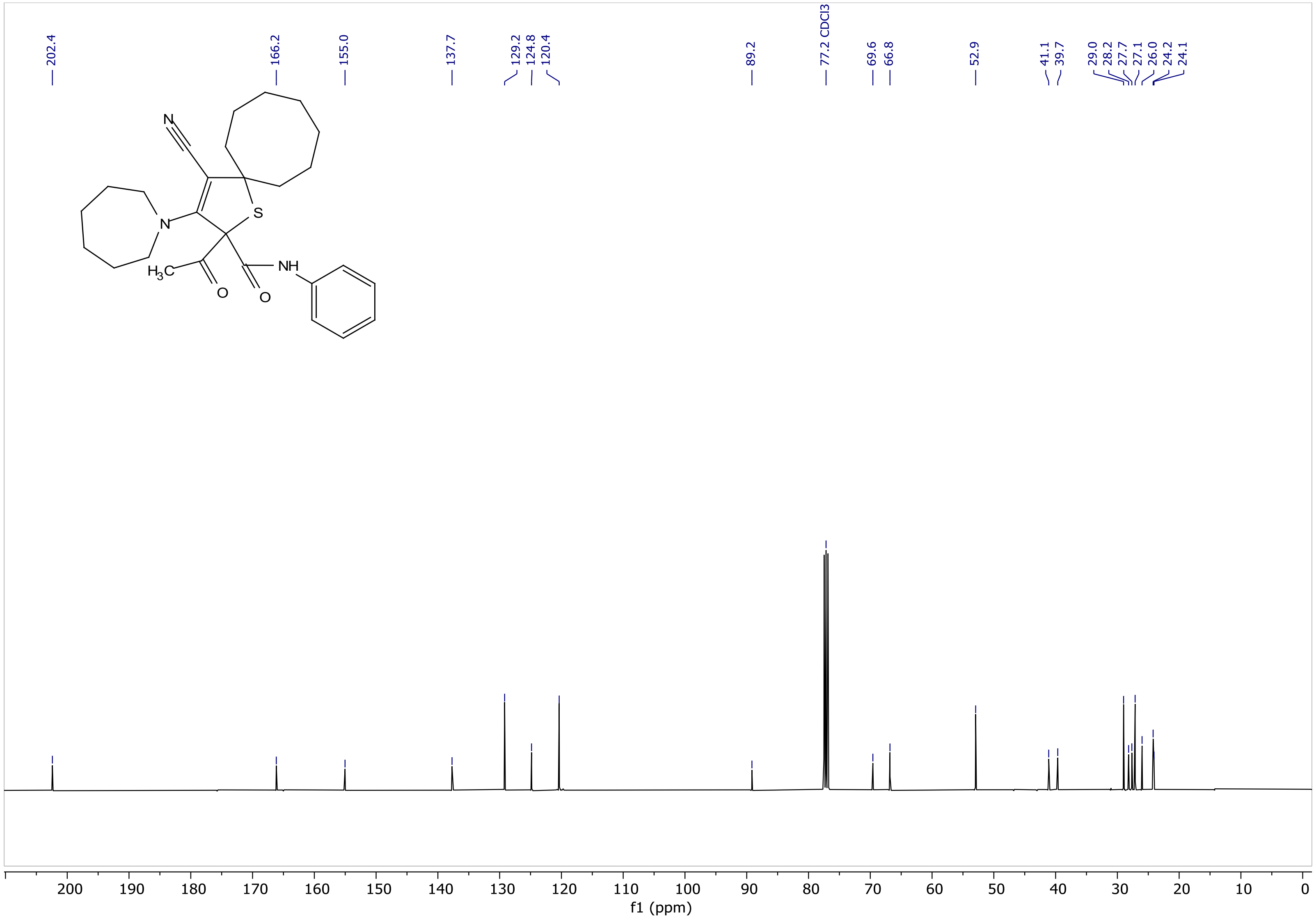

^{13}C NMR (100 MHz, $CDCl_3$-*d*) of **1n**

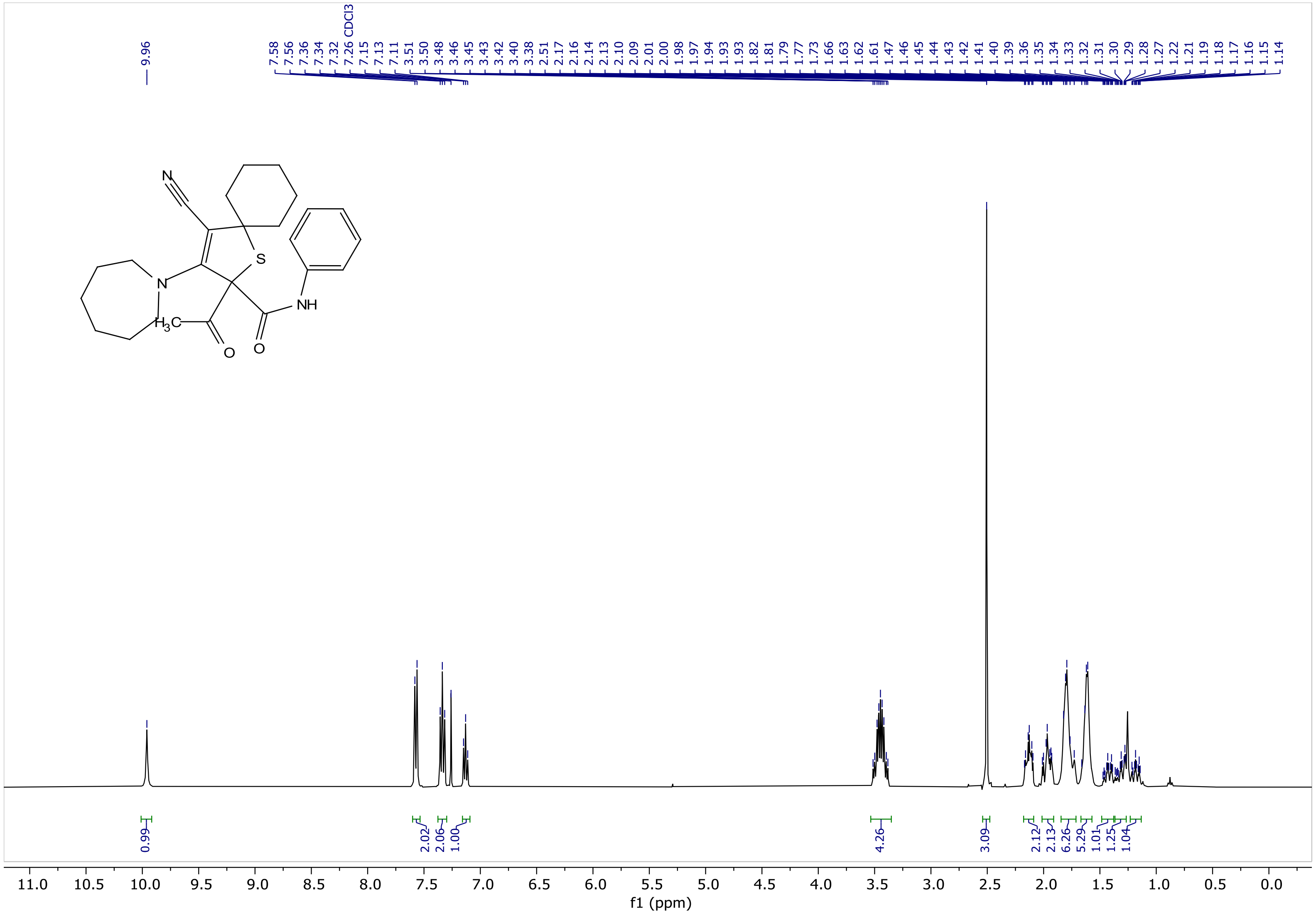

^{1}H NMR (400 MHz, $CDCl_3$-*d*) of **1o**

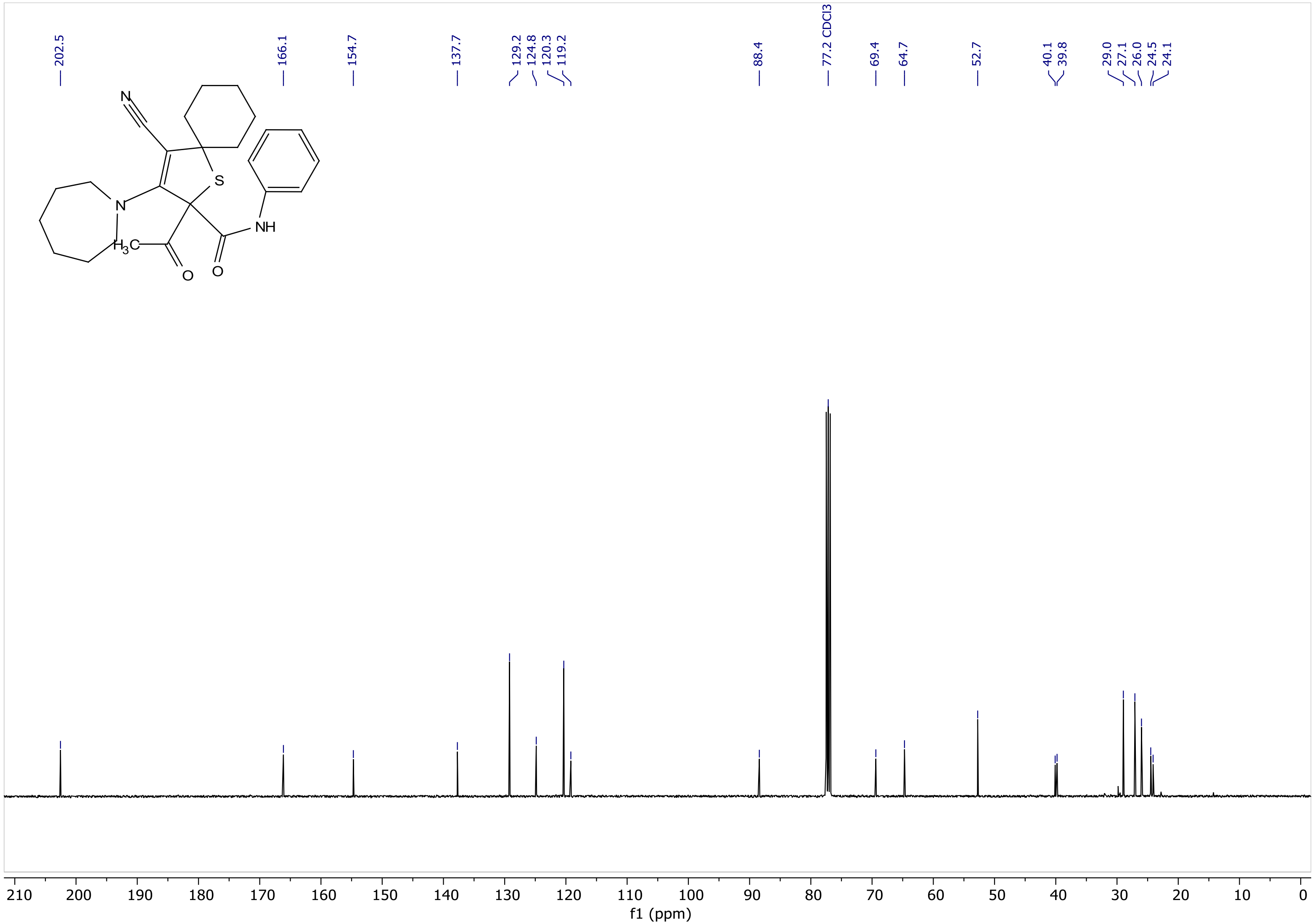

^{13}C NMR (100 MHz, CDCl$_3$-*d*) of **1o**

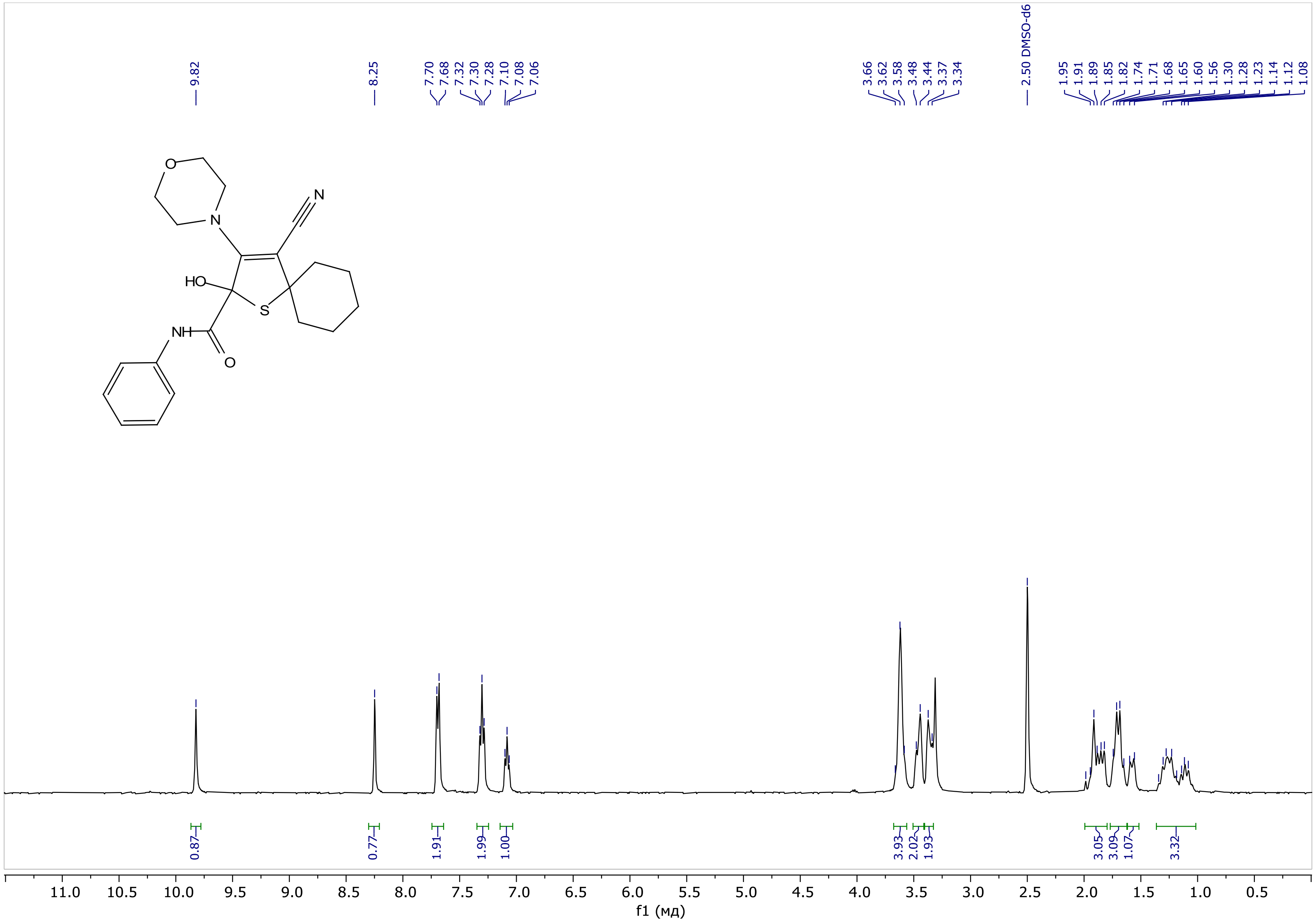

^{1}H NMR (400 MHz, DMSO-d_6) of **2a**

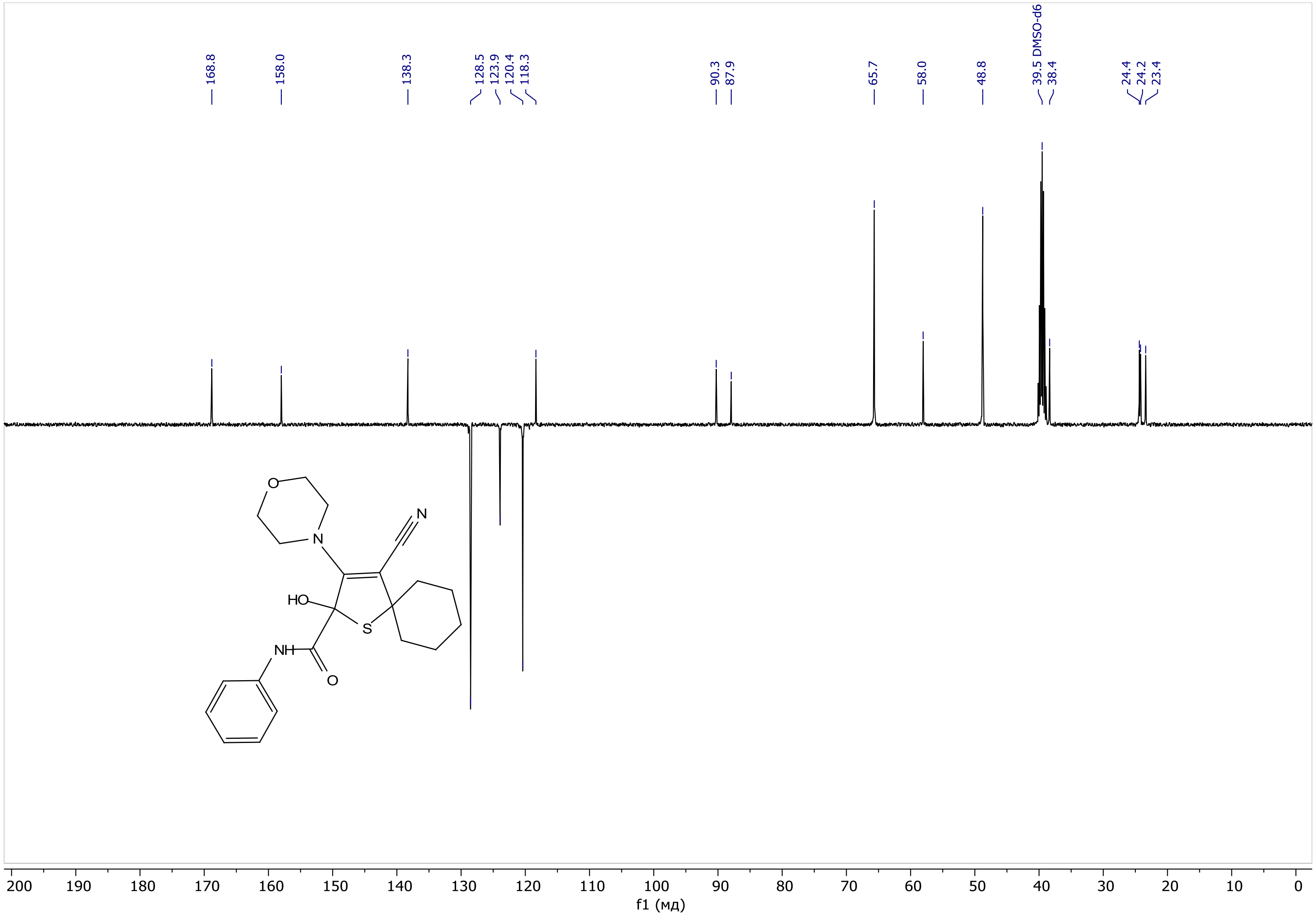

^{13}C NMR (100 MHz, DMSO-d_6) of **2a**

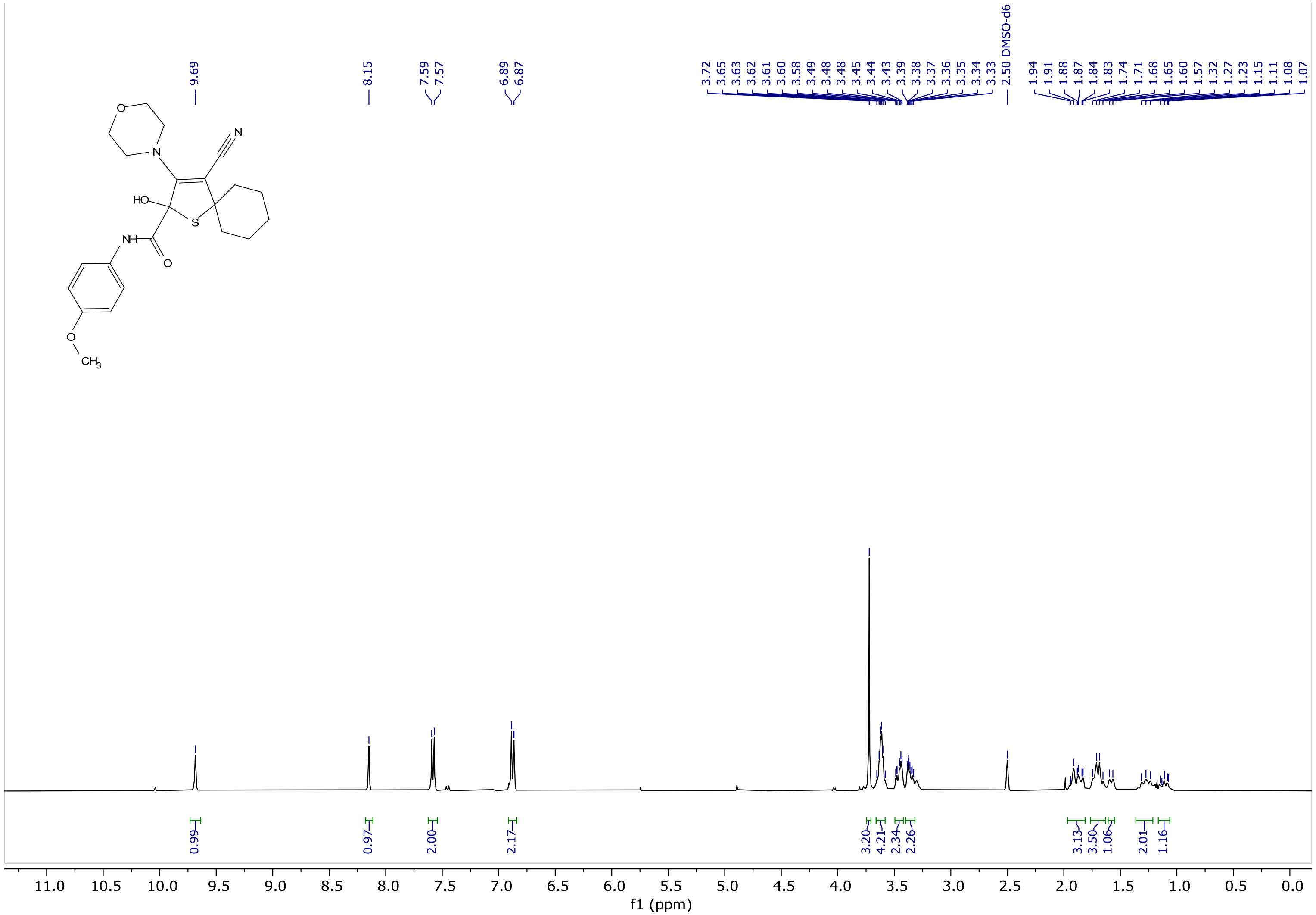

^{1}H NMR (400 MHz, DMSO-d_6) of **2b**

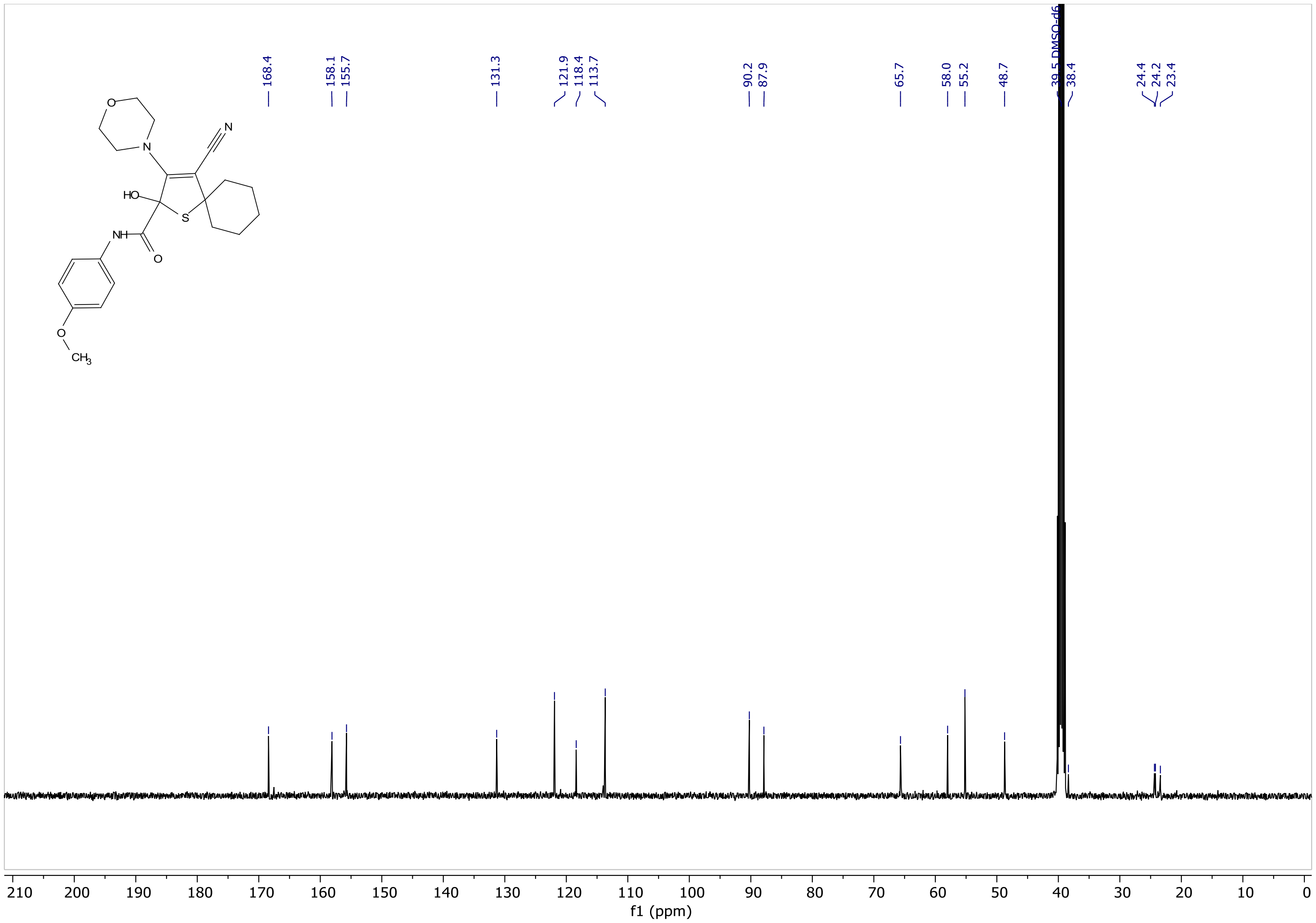

^{13}C NMR (100 MHz, DMSO-d_6) of **2b**

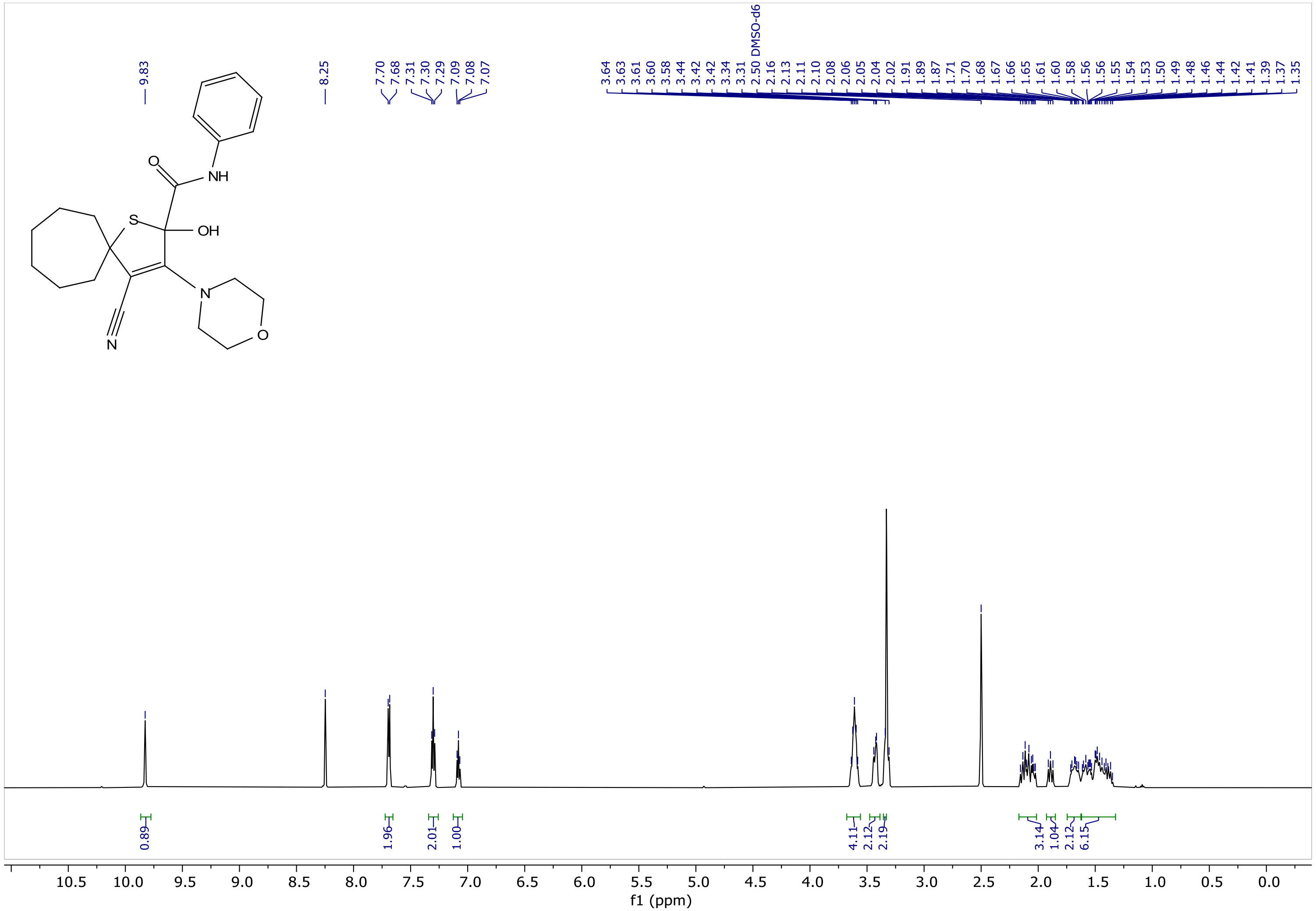

^{1}H NMR (400 MHz, DMSO-d_6) of **2c**

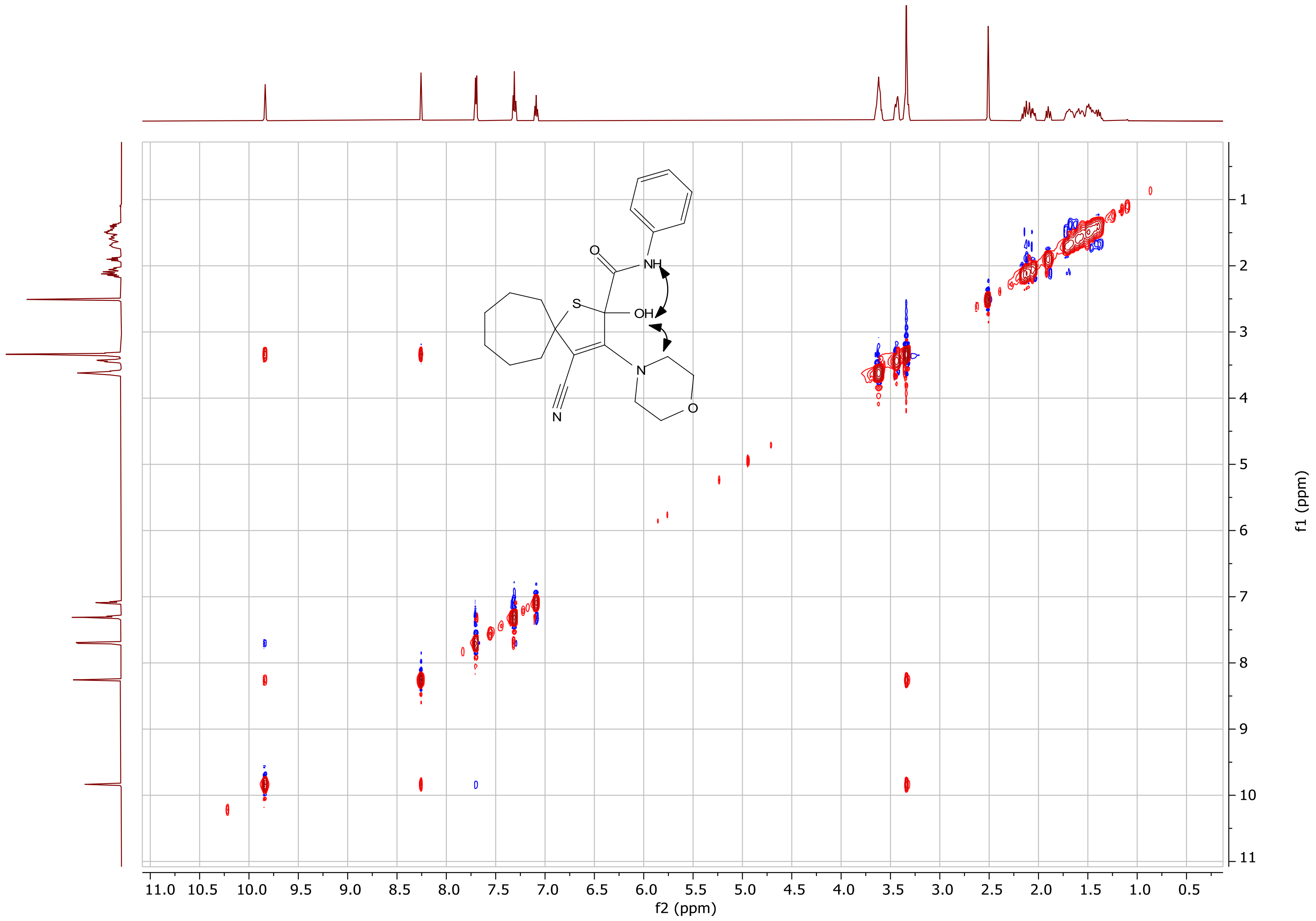
O
NH
S
OH
N
O
N
f2 (ppm)
f1 (ppm)

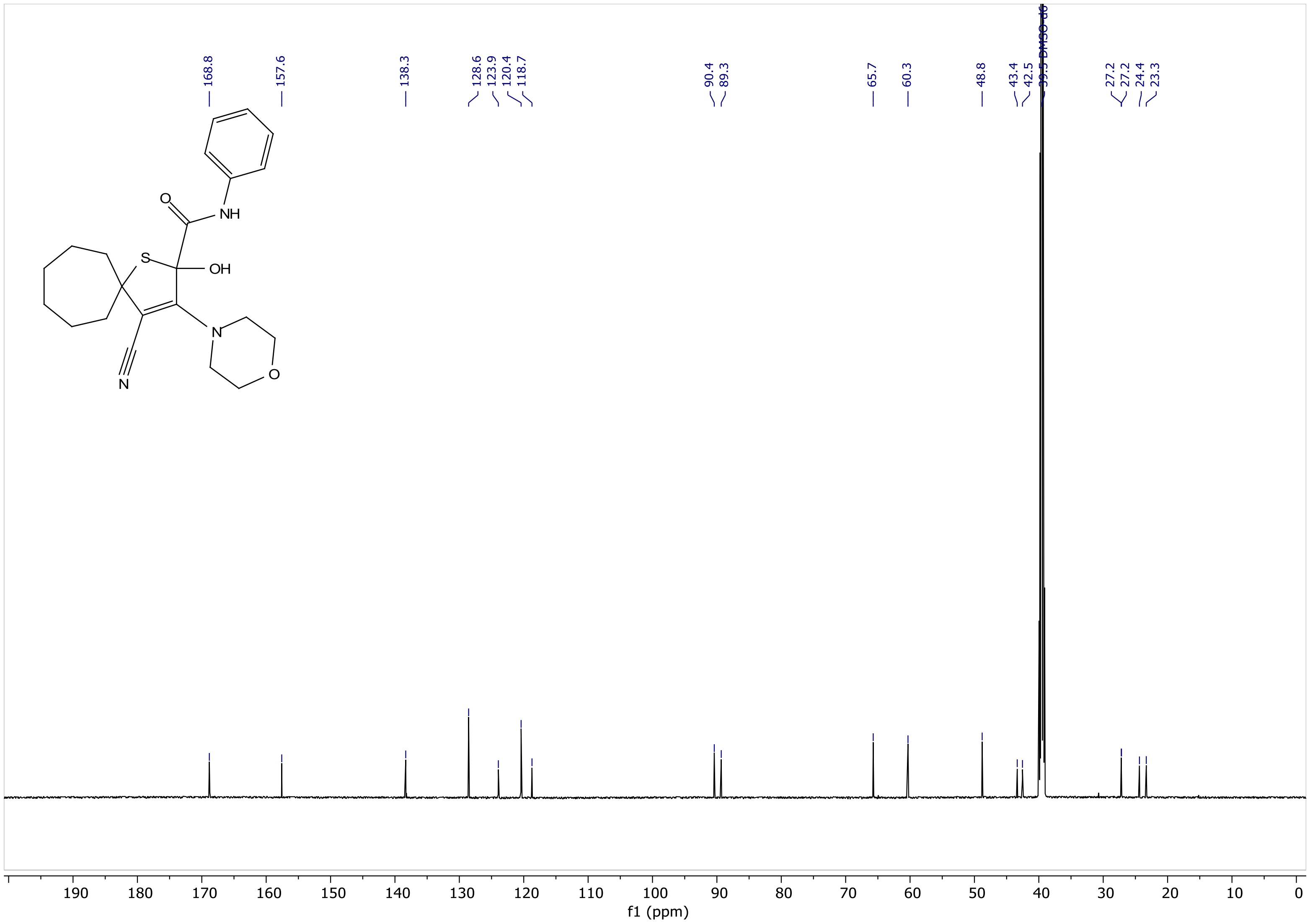

^{13}C NMR (100 MHz, DMSO-d_6) of **2c**

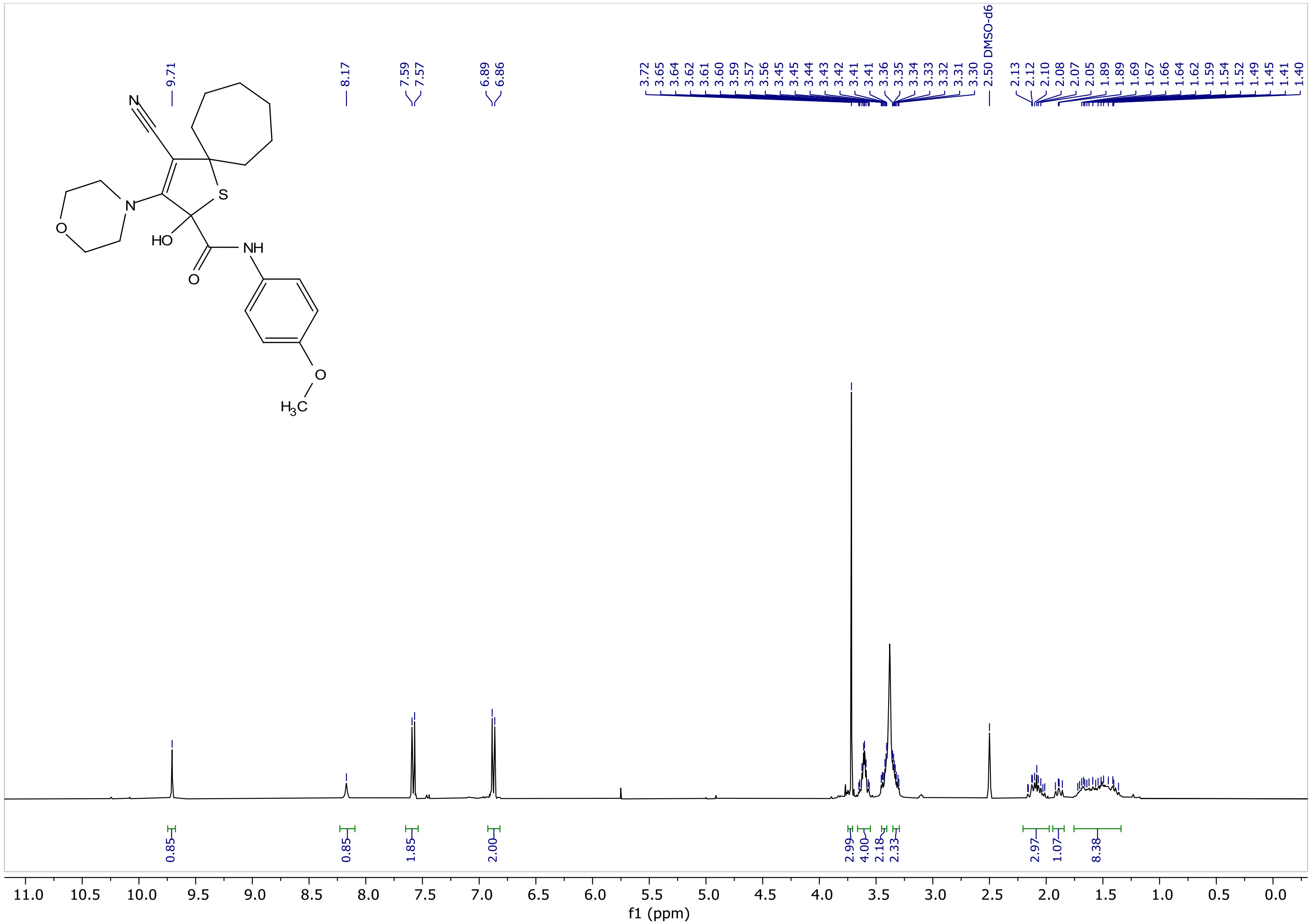

^{1}H NMR (400 MHz, DMSO-d_6) of **2d**

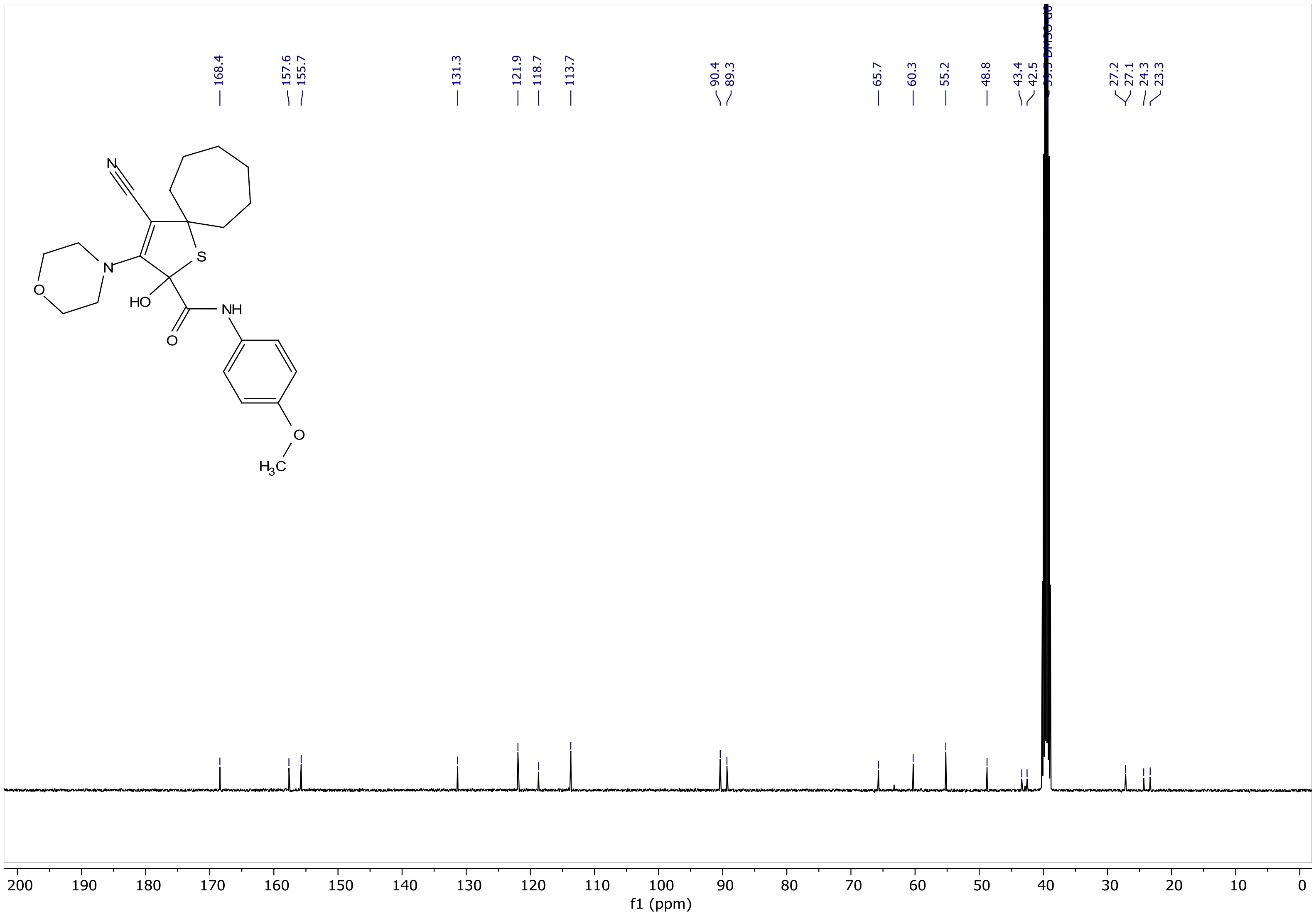

^{13}C NMR (100 MHz, DMSO-d_6) of **2d**

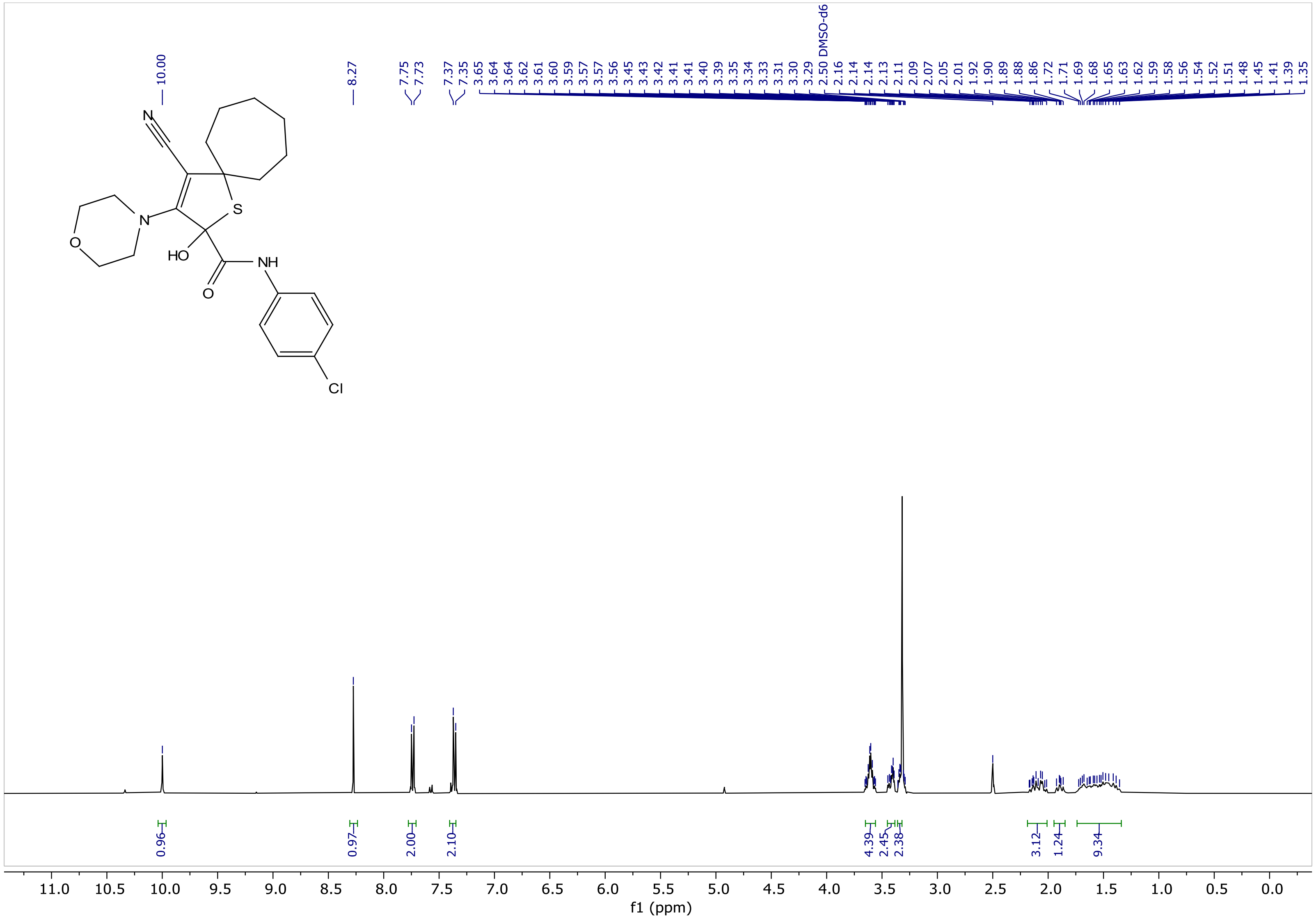

^{1}H NMR (400 MHz, DMSO-d_6) of **2e**

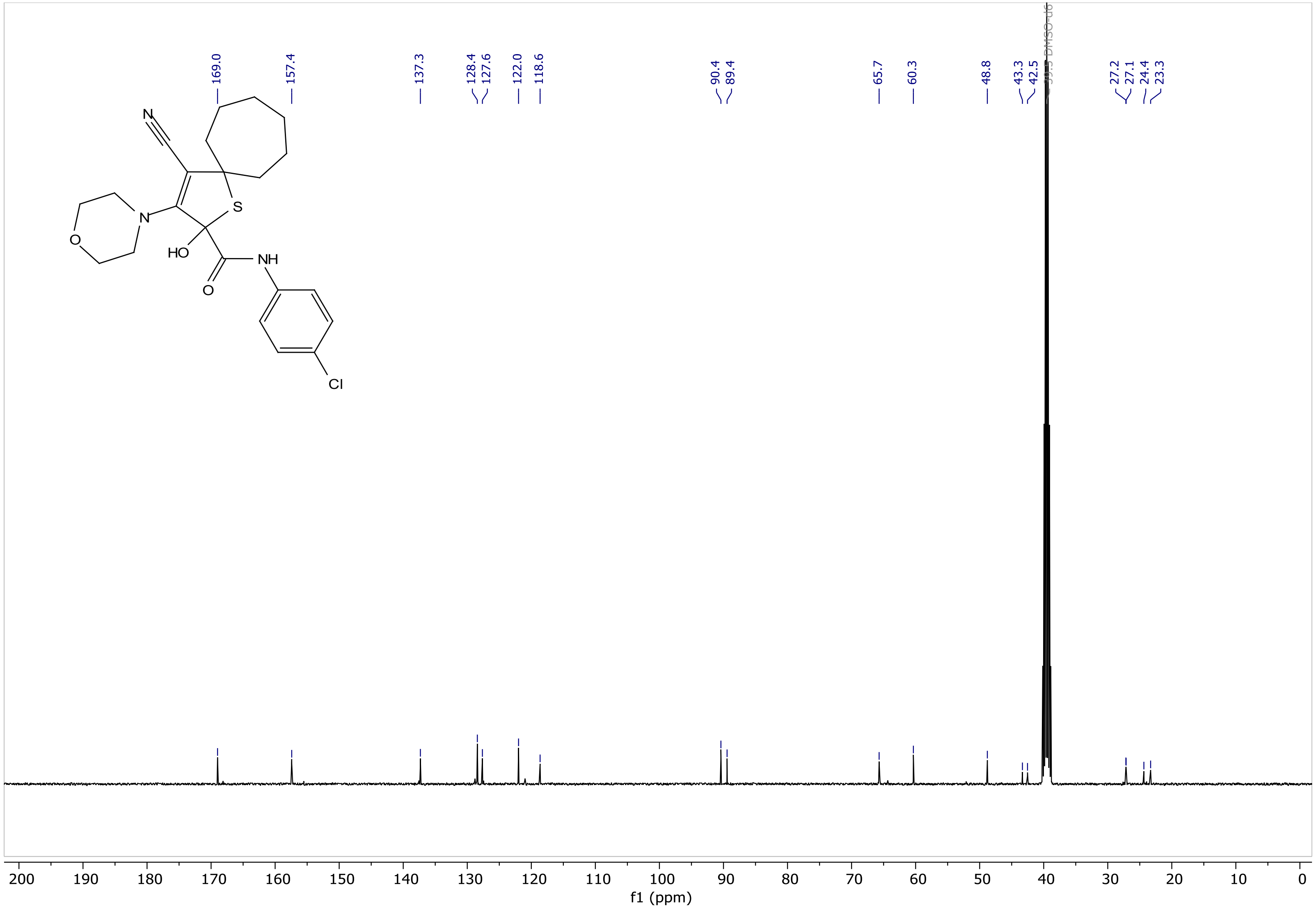

^{13}C NMR (100 MHz, DMSO-d_6) of **2e**

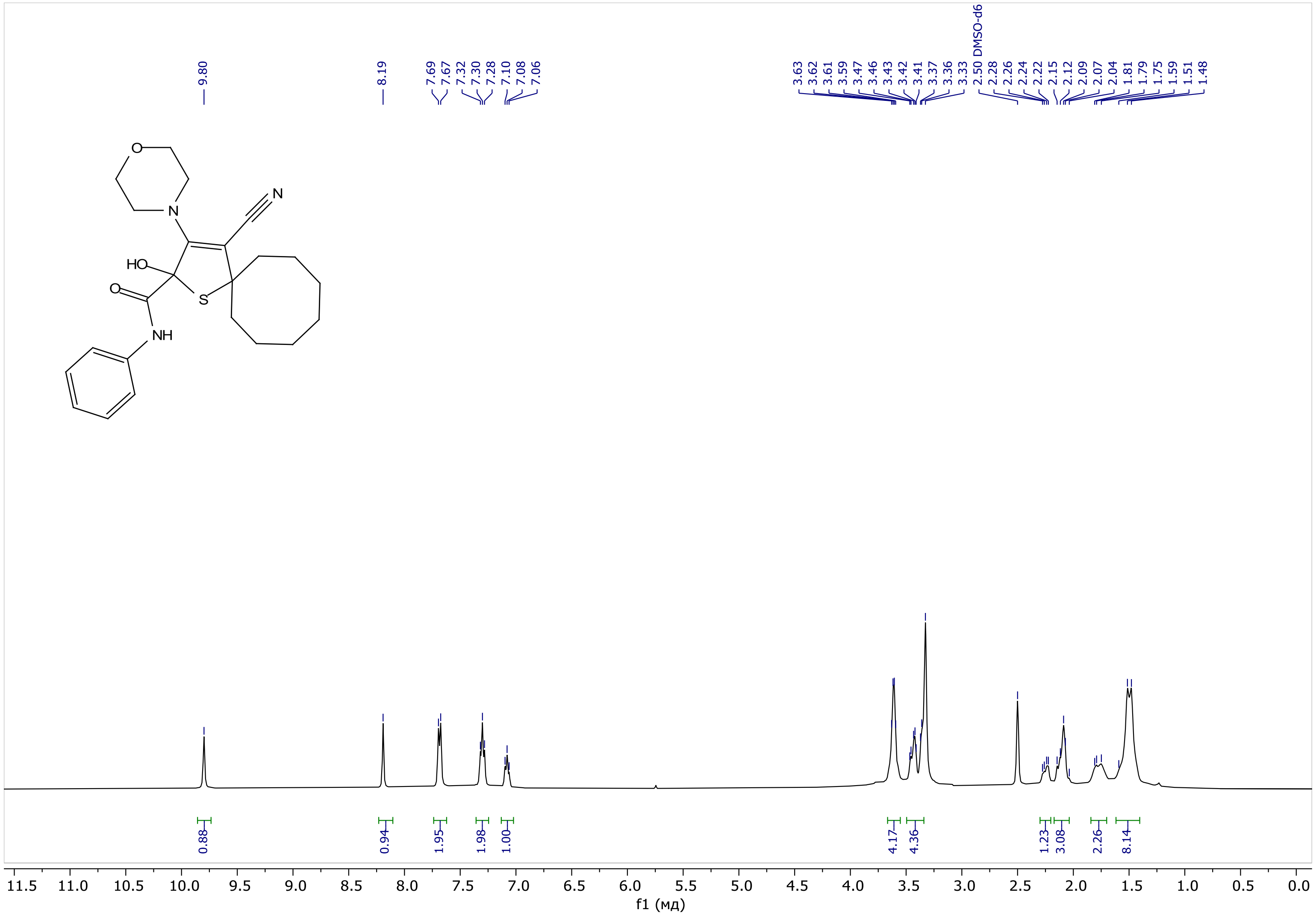

^{1}H NMR (400 MHz, DMSO-d_6) of **2f**

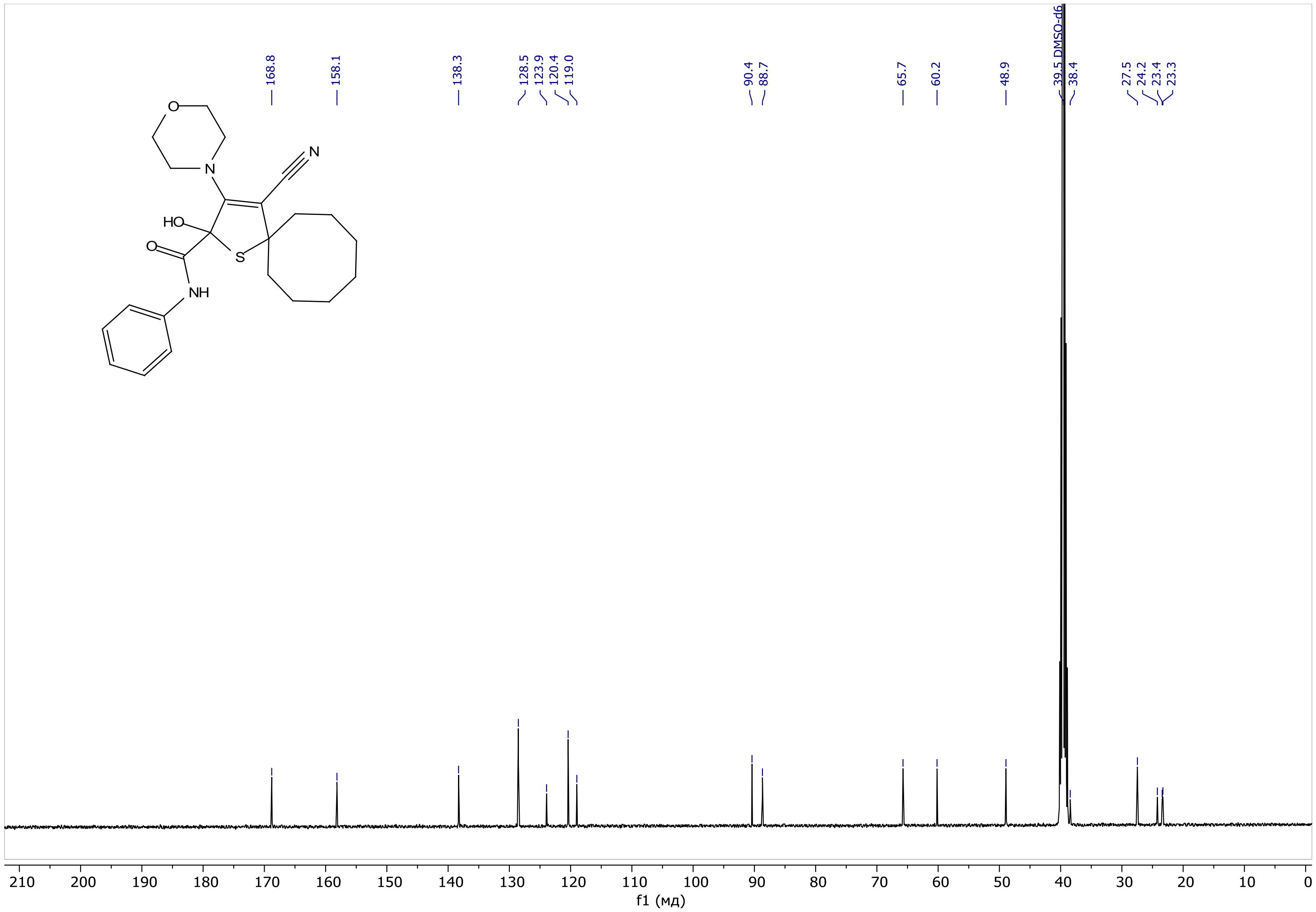

^{13}C NMR (100 MHz, DMSO-d_6) of **2f**

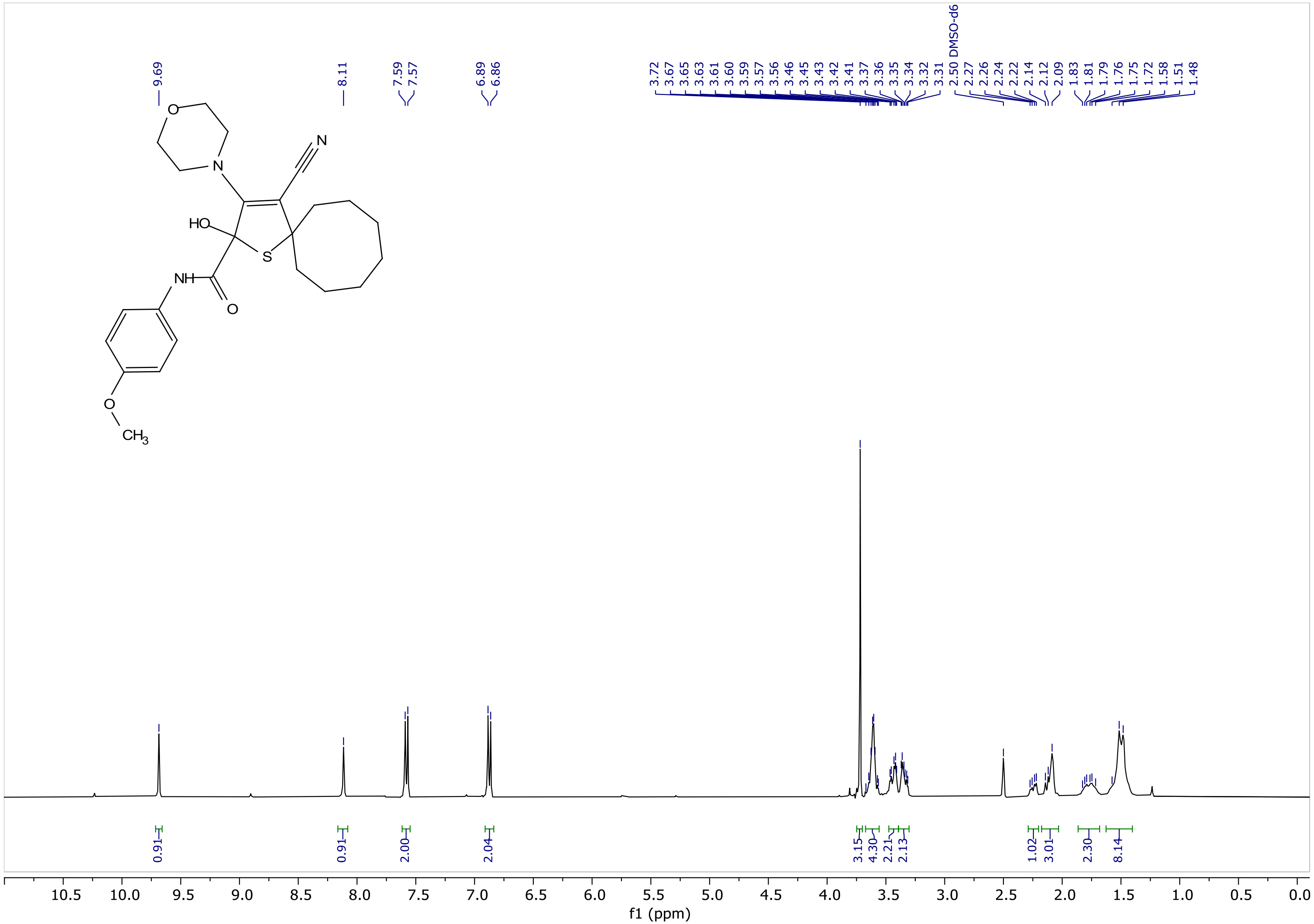

^{1}H NMR (400 MHz, DMSO-d_6) of **2g**

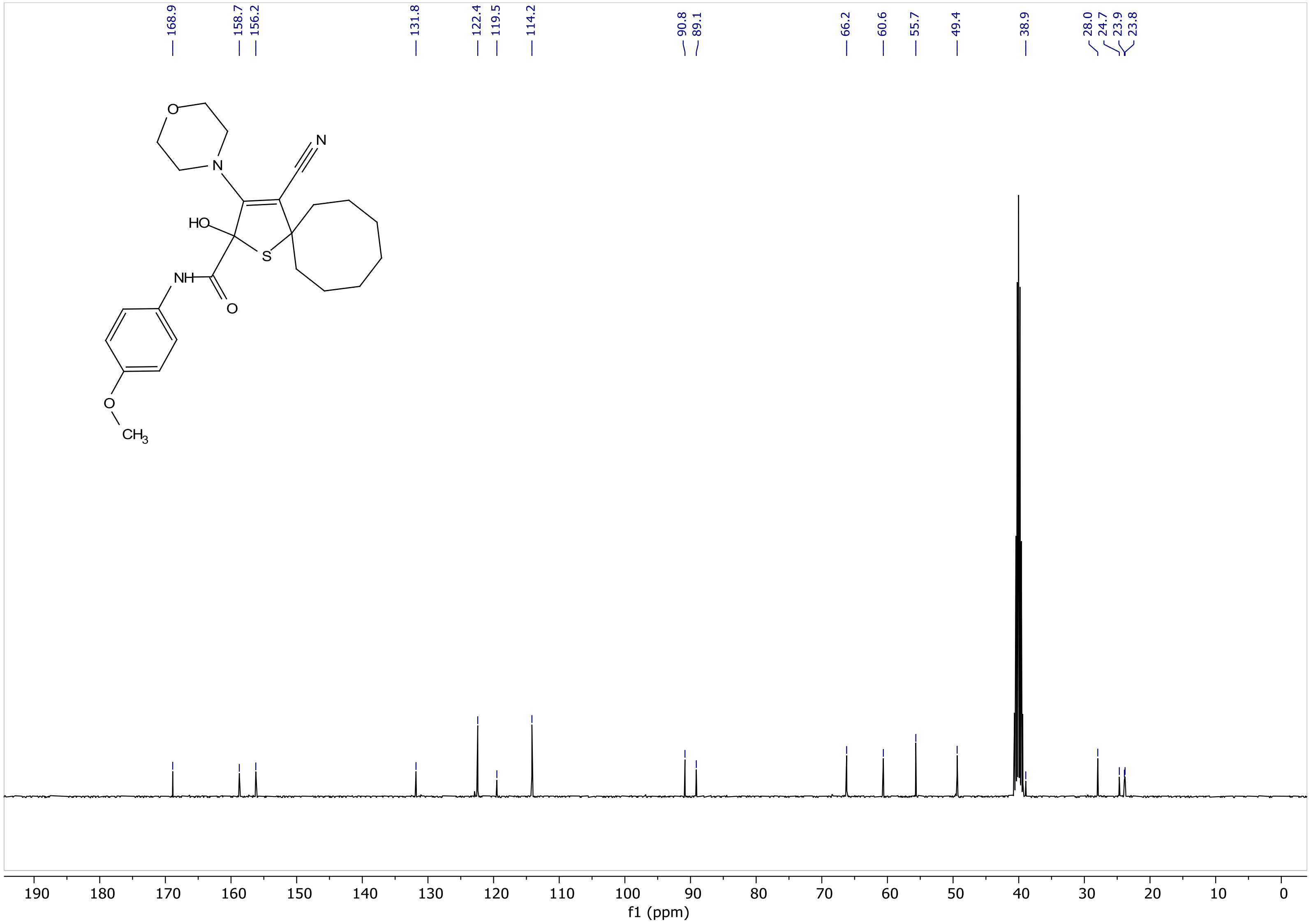

^{13}C NMR (100 MHz, DMSO-d_6) of **2g**

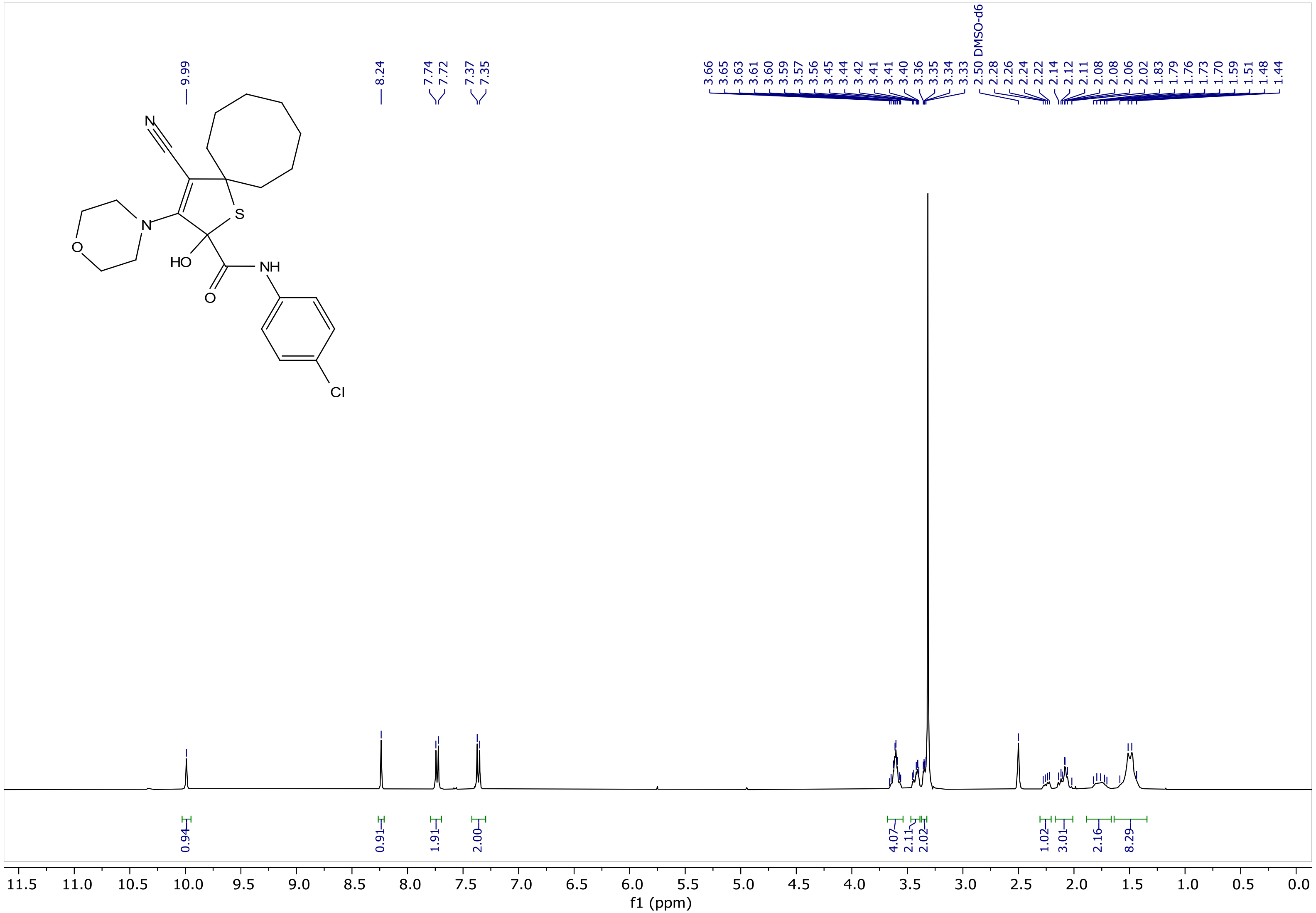

^{1}H NMR (400 MHz, DMSO-d_6) of **2h**

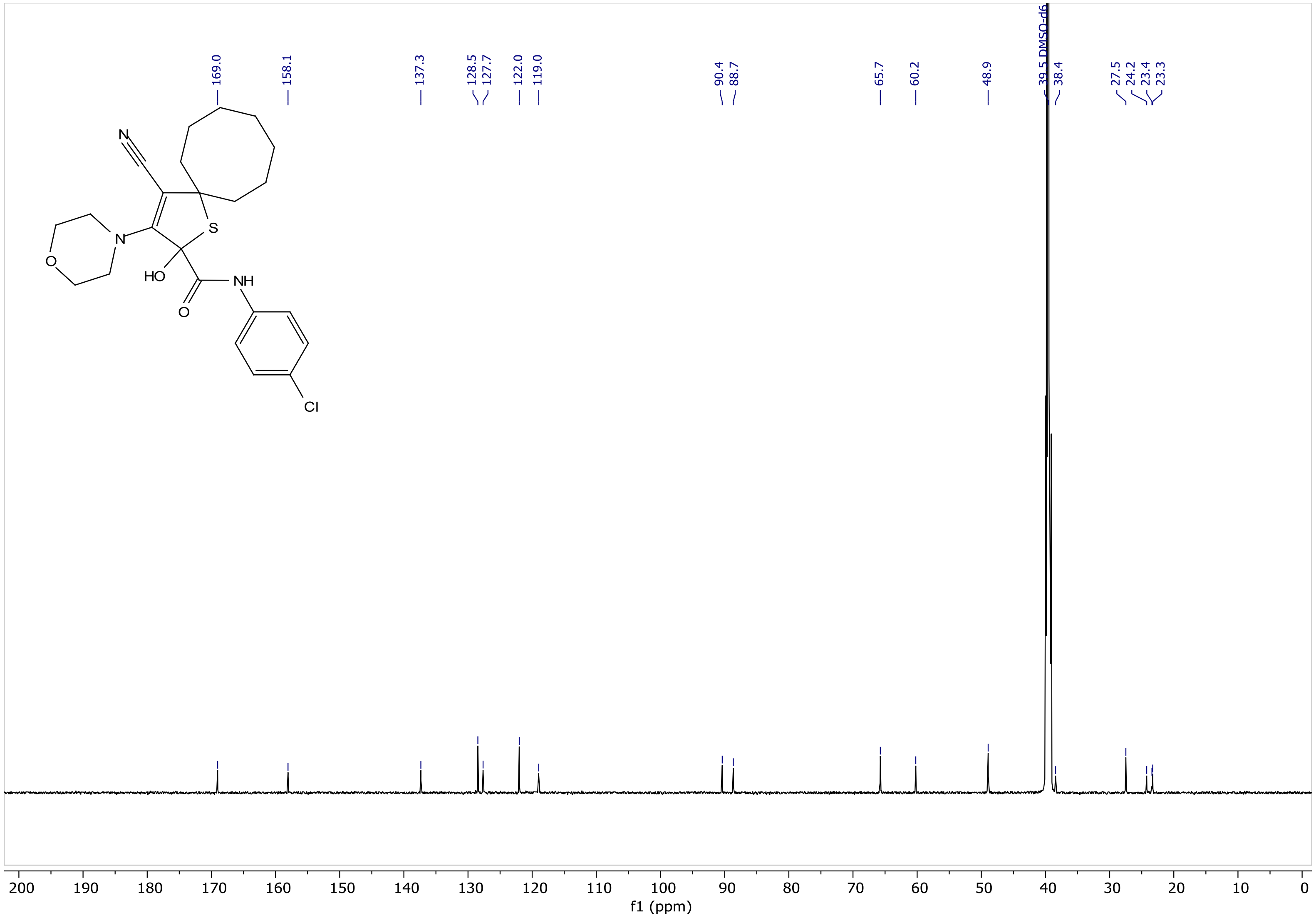

^{13}C NMR (100 MHz, DMSO-d_6) of **2h**

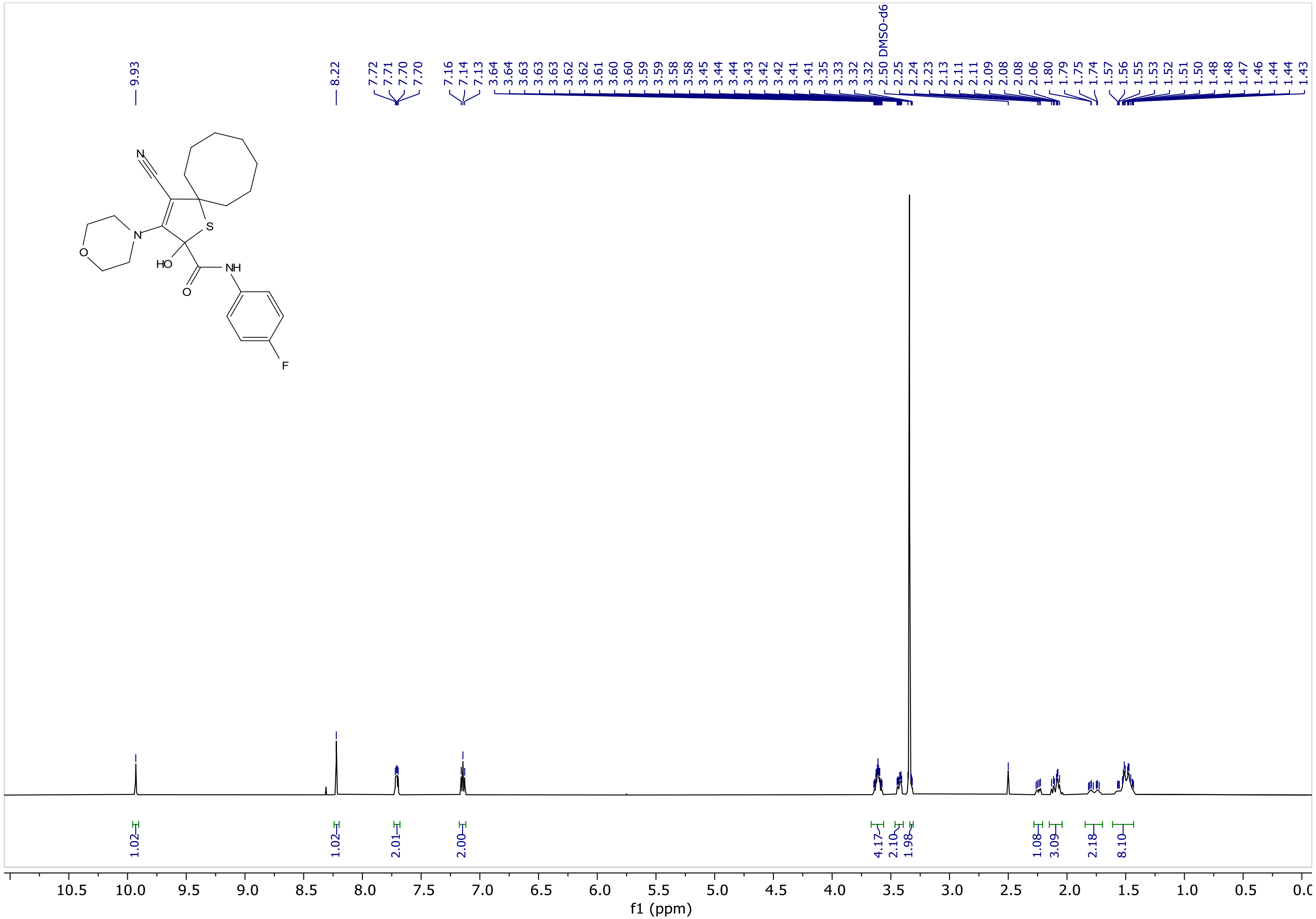

^{1}H NMR (600 MHz, DMSO-d_6) of **2i**

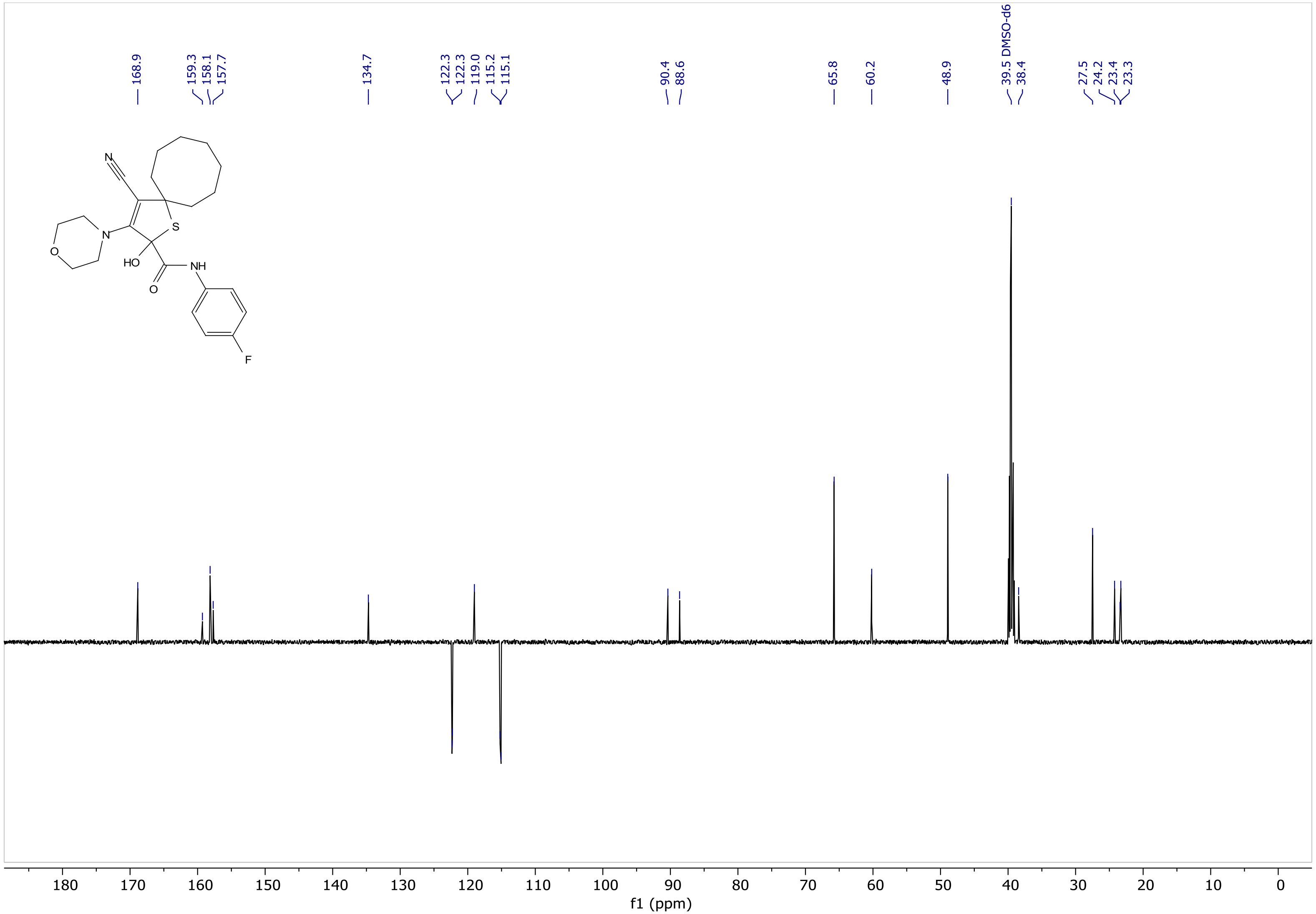

^{13}C NMR (100 MHz, DMSO-d_6) of **2i**

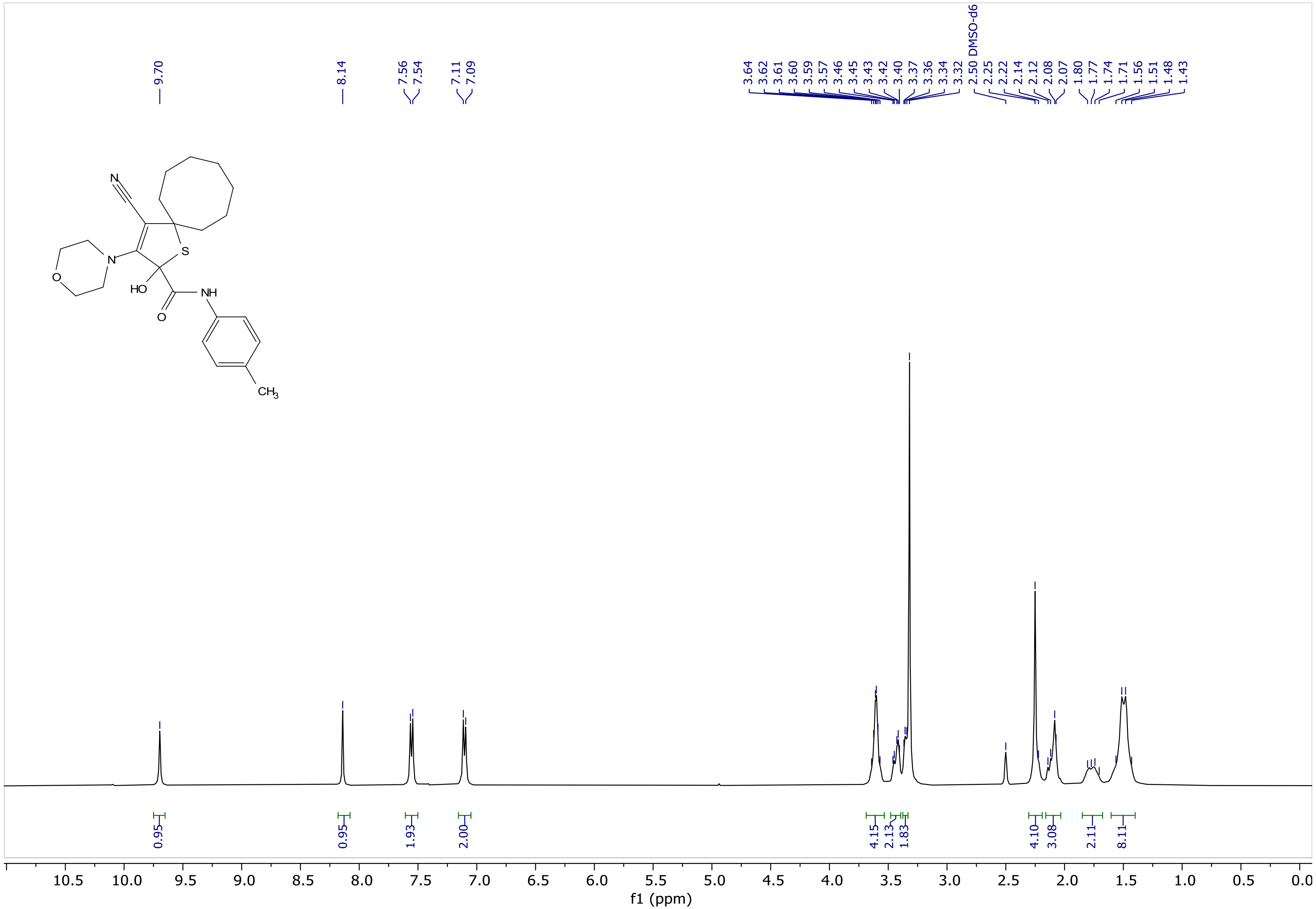

^{1}H NMR (400 MHz, DMSO-d_6) of **2j**

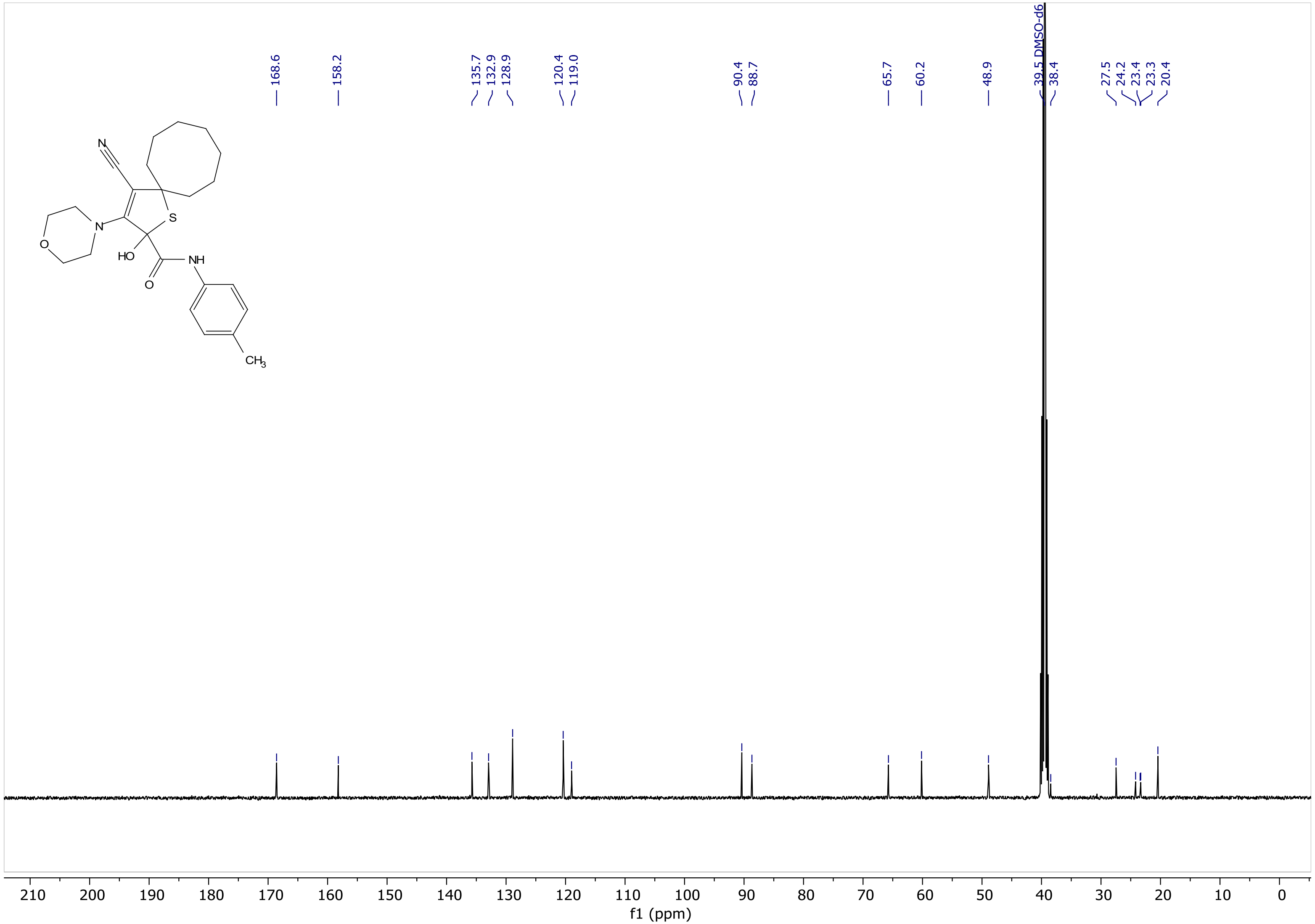

^{13}C NMR (100 MHz, DMSO-d_6) of **2j**

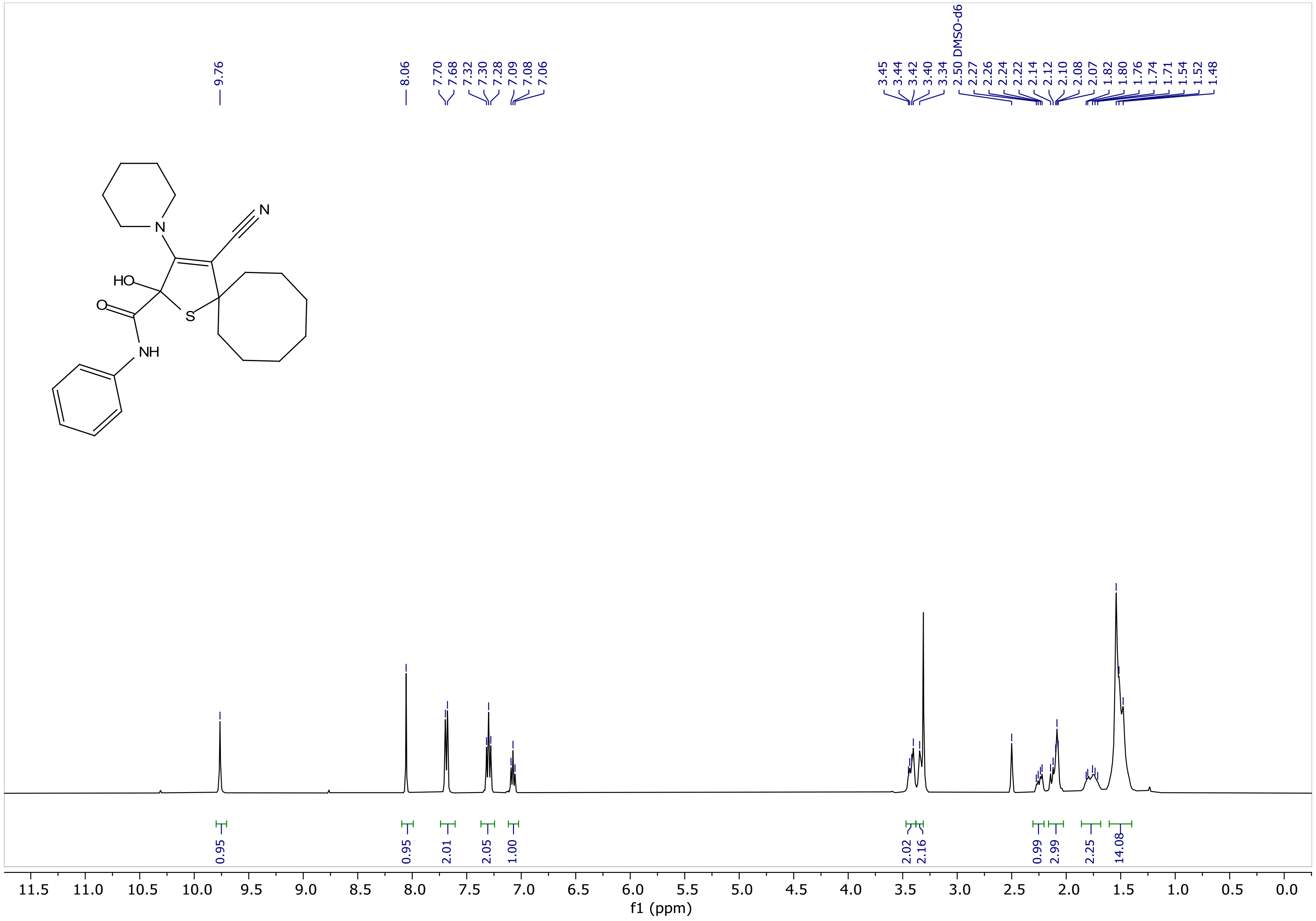

^{1}H NMR (400 MHz, DMSO-d_6) of **2k**

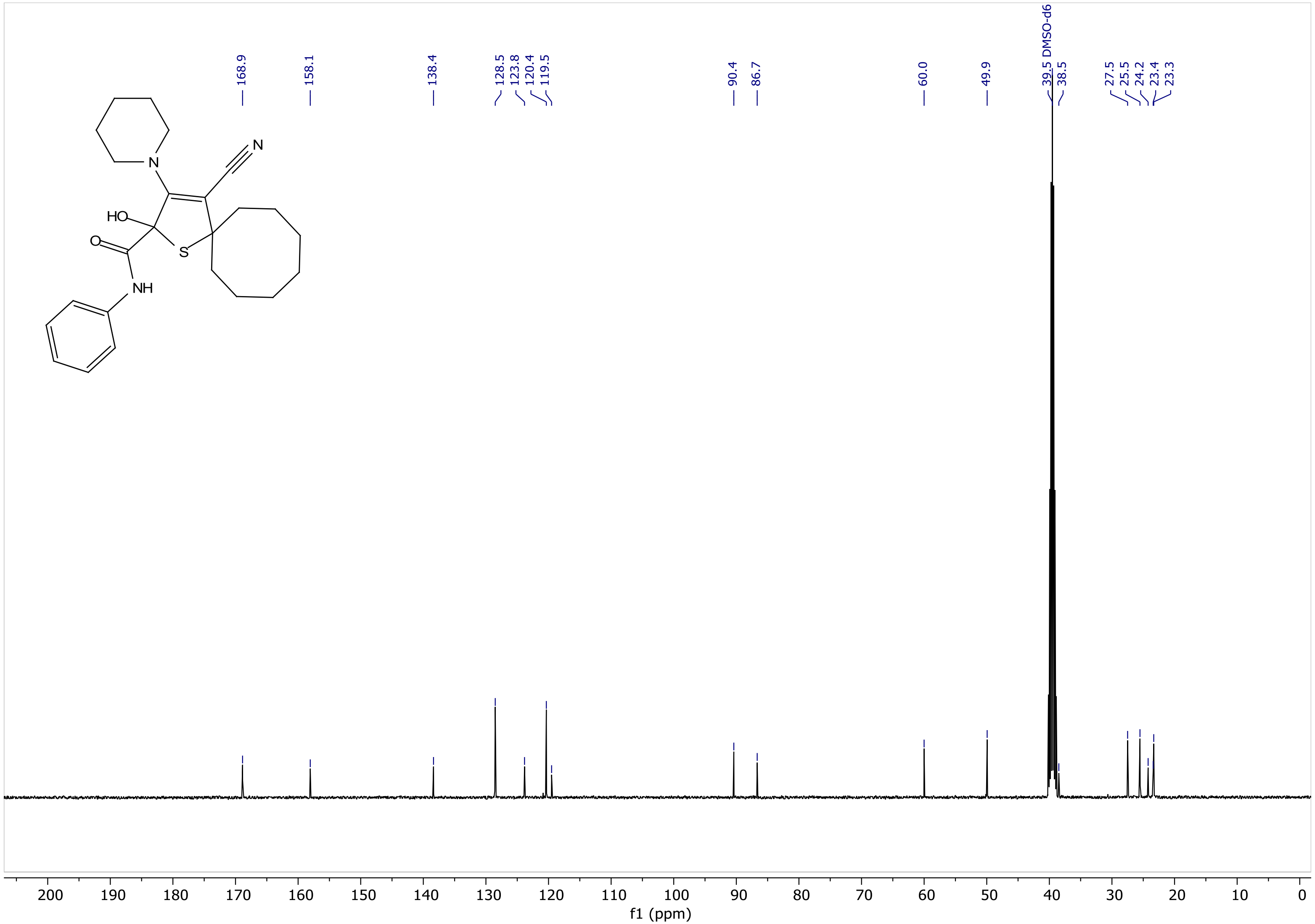

^{13}C NMR (100 MHz, DMSO-d_6) of **2k**

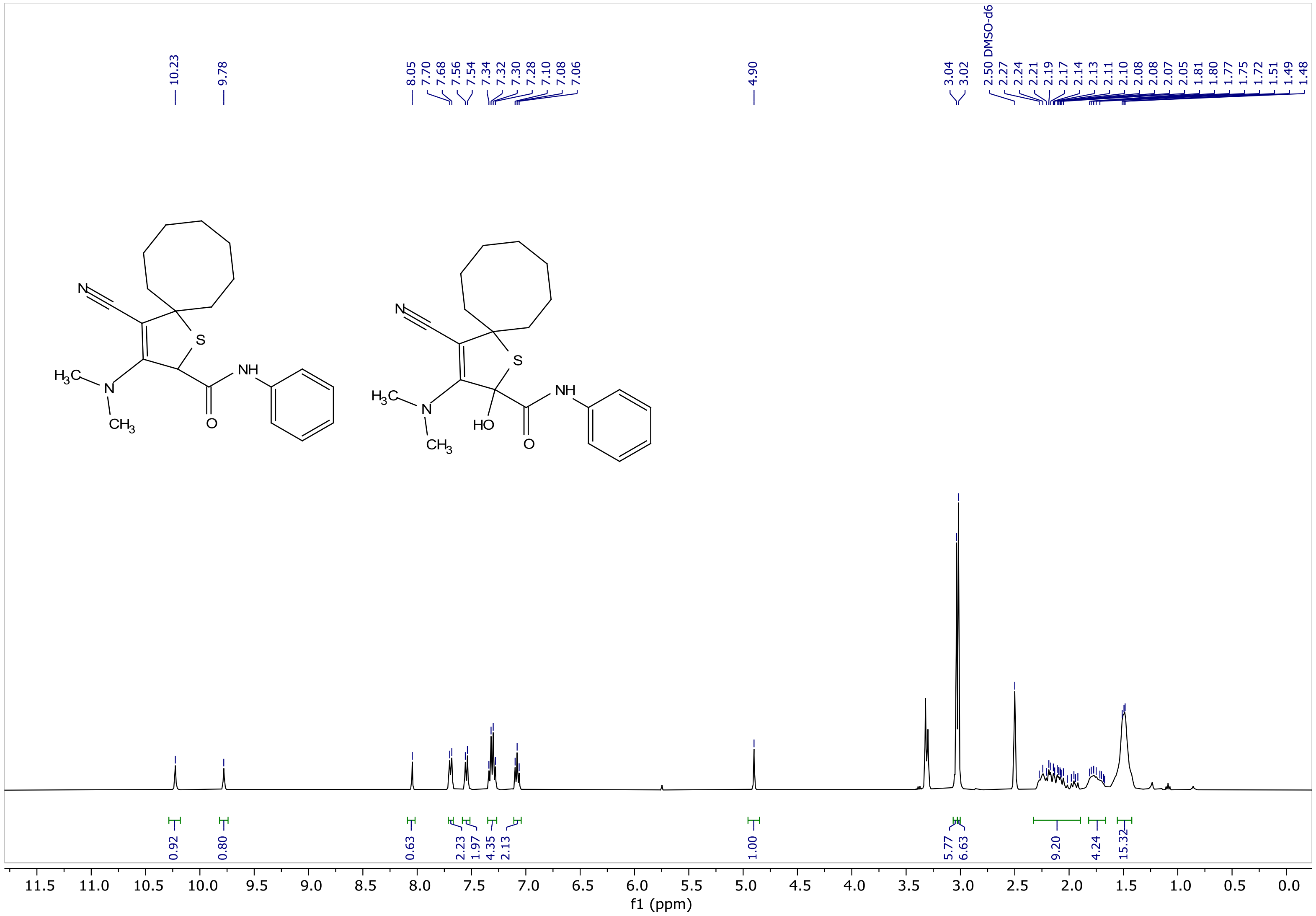

^{1}H NMR (400 MHz, DMSO-d_6) of **2l**, **l´**

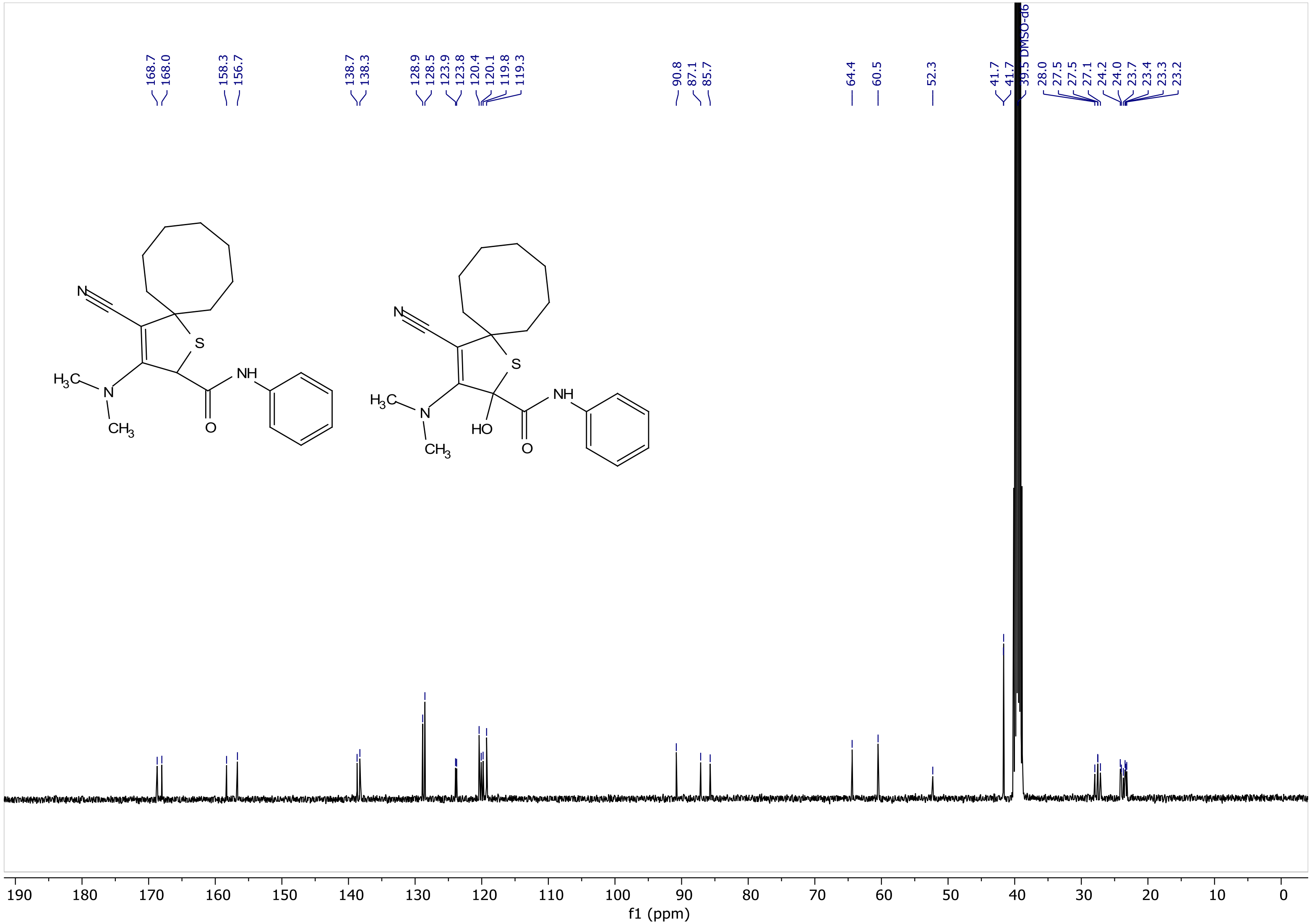

^{13}C NMR (100 MHz, DMSO-d_6) of **2l, l′**

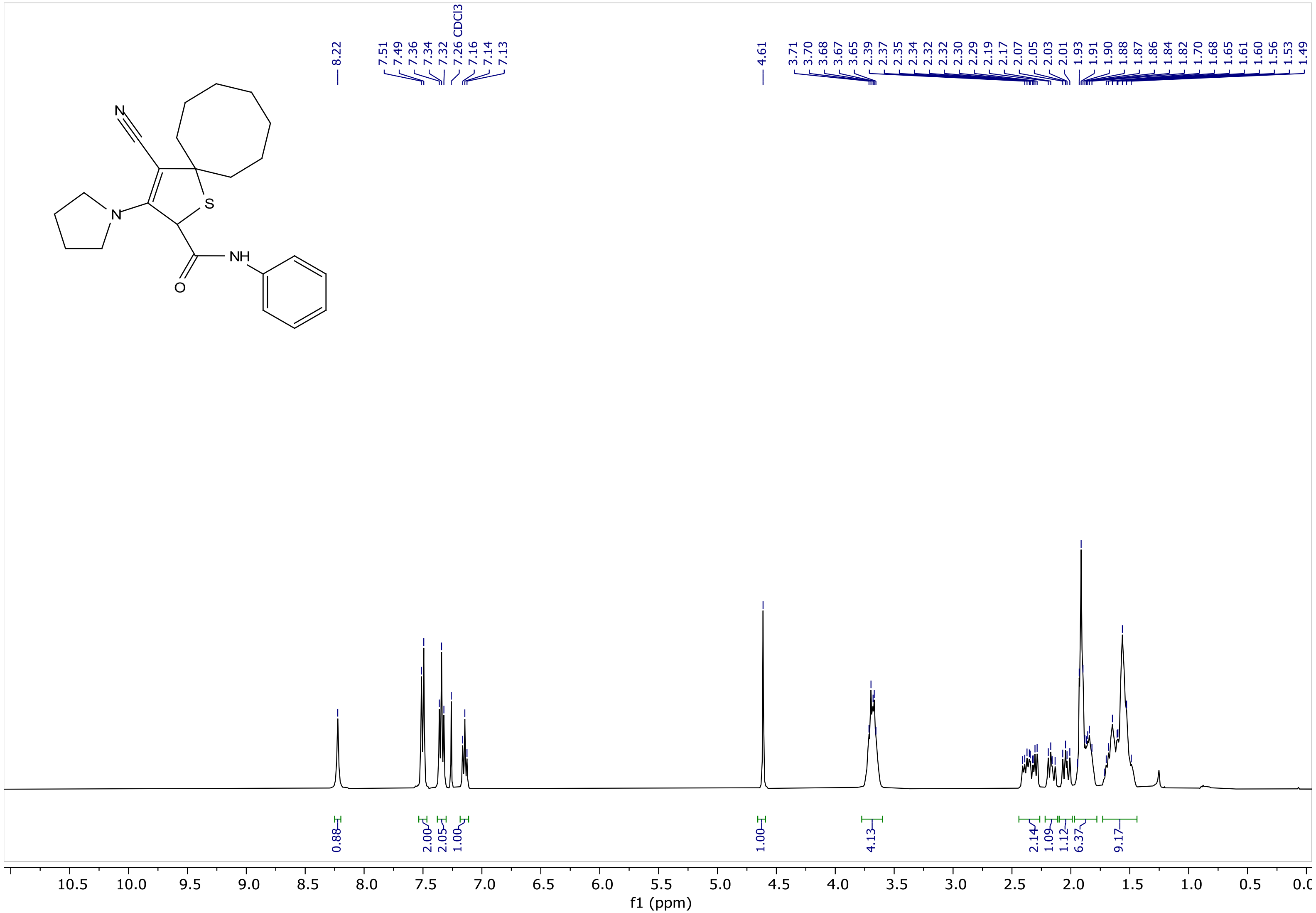

^{1}H NMR (400 MHz, $CDCl_3$-*d*) of **2m**

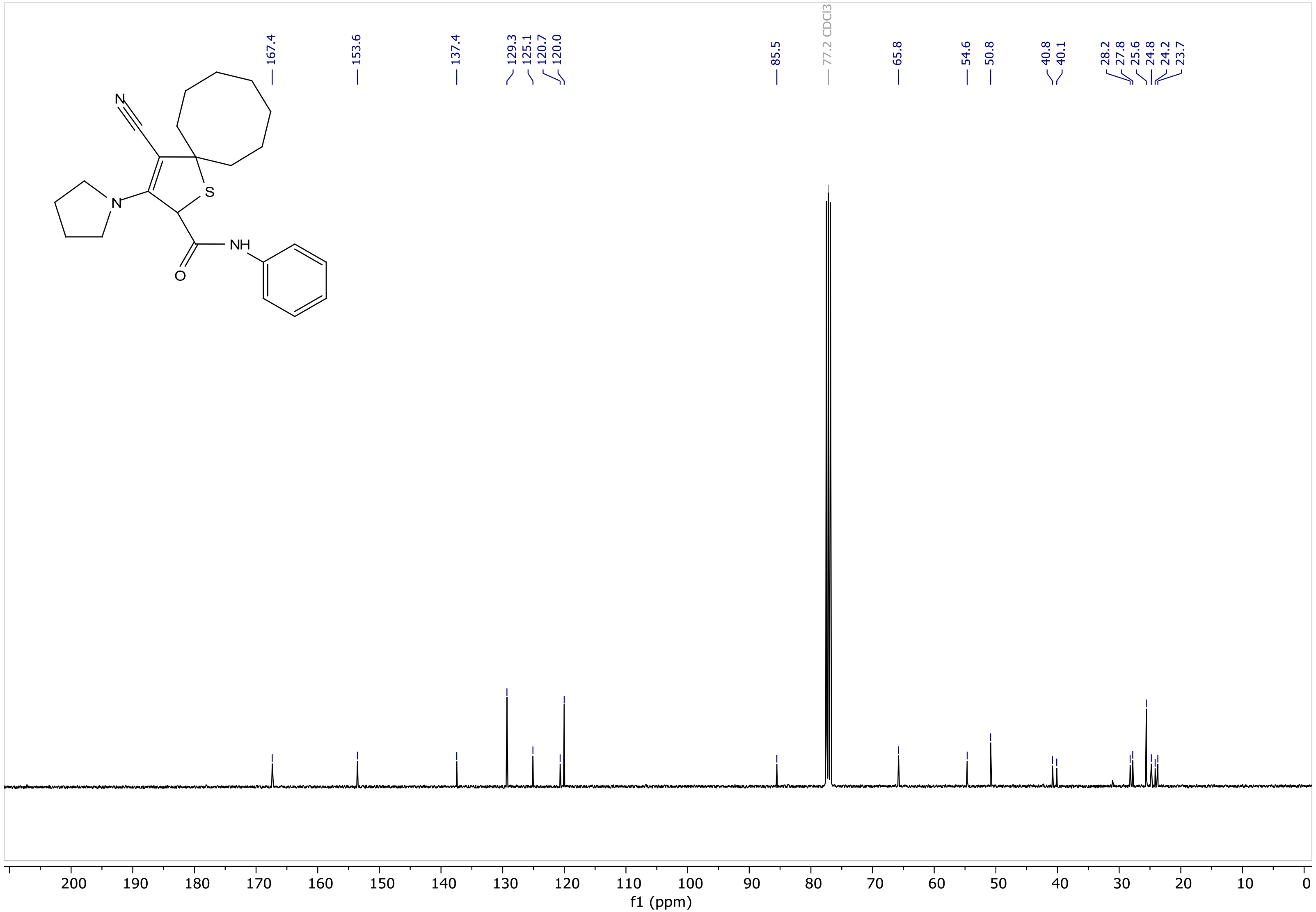

^{13}C NMR (100 MHz, $CDCl_3$-*d*) of **2m**

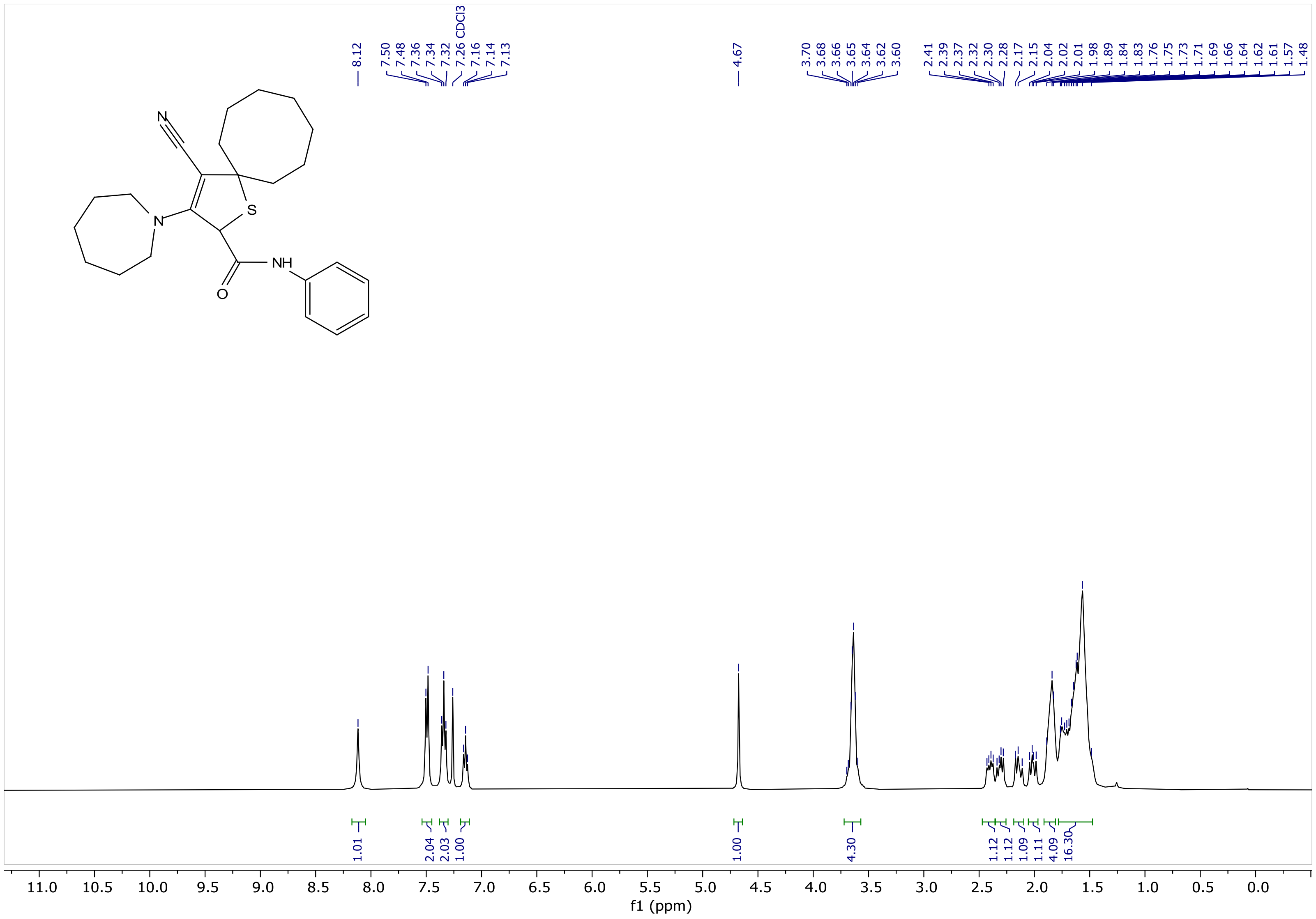

^{1}H NMR (400 MHz, $CDCl_3$-*d*) of **2n**

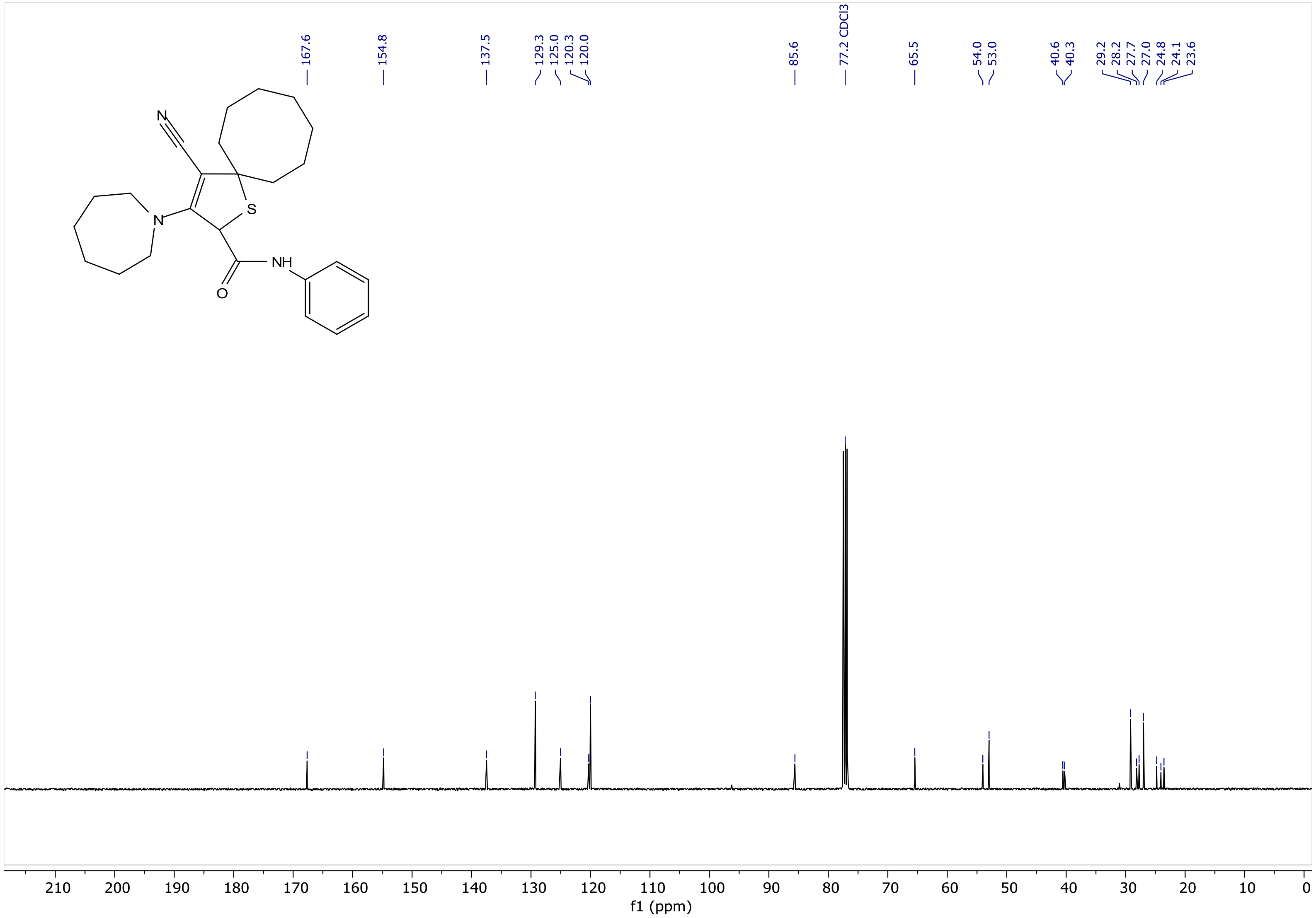

^{13}C NMR (100 MHz, $CDCl_3$-*d*) of **2n**

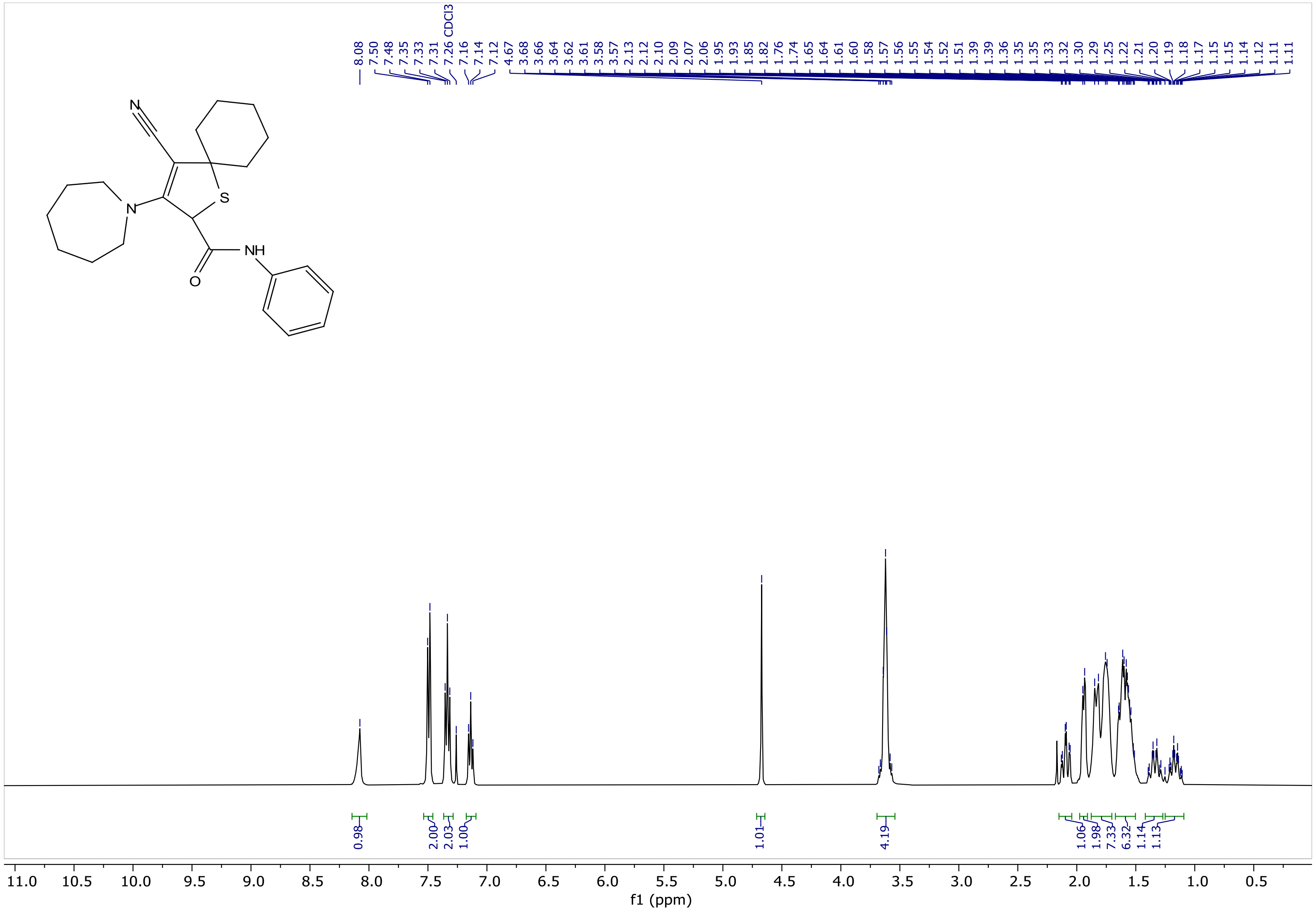

^{1}H NMR (400 MHz, $CDCl_3$-*d*) of **2o**

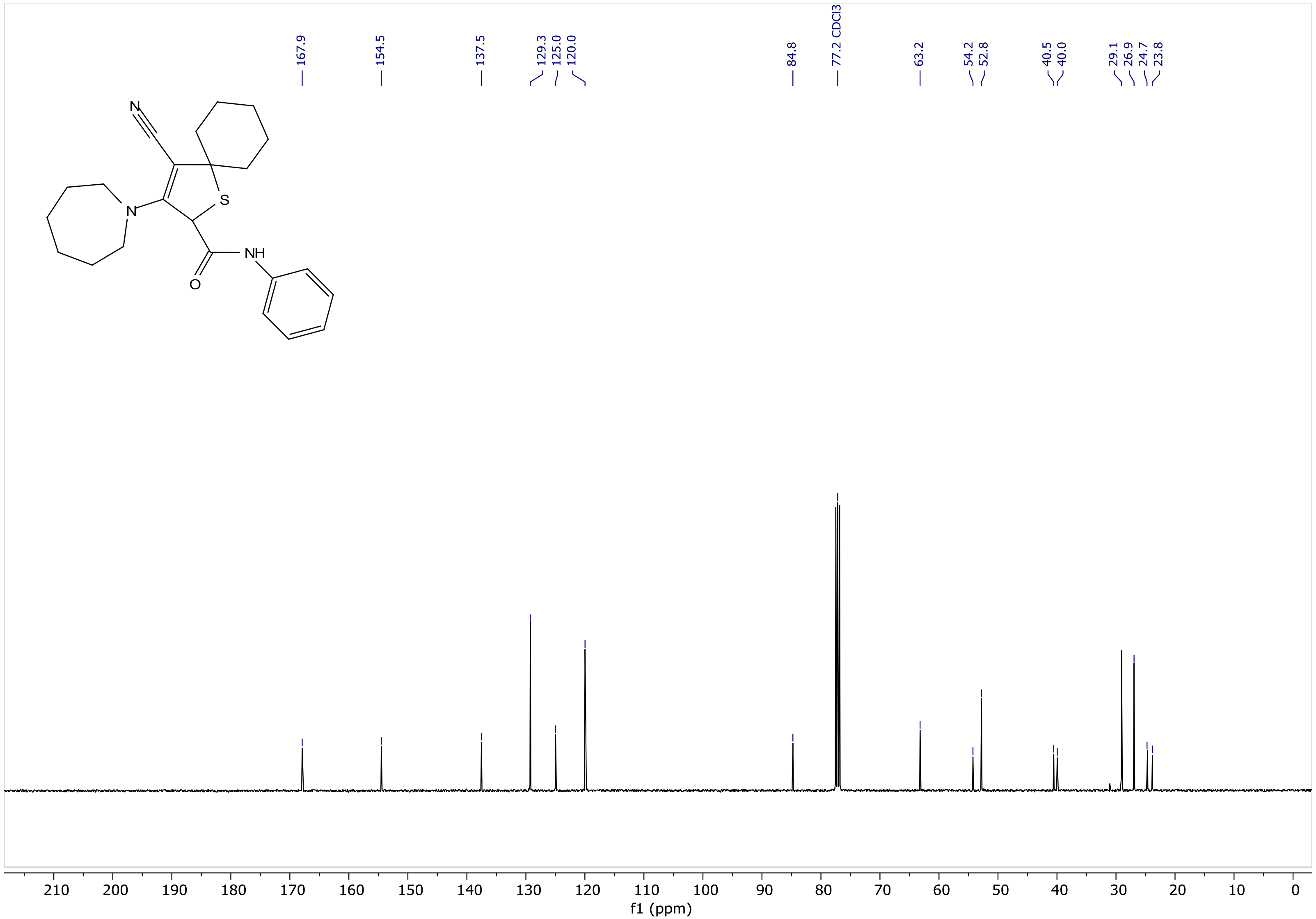

^{13}C NMR (100 MHz, $CDCl_3$-*d*) of **2o**

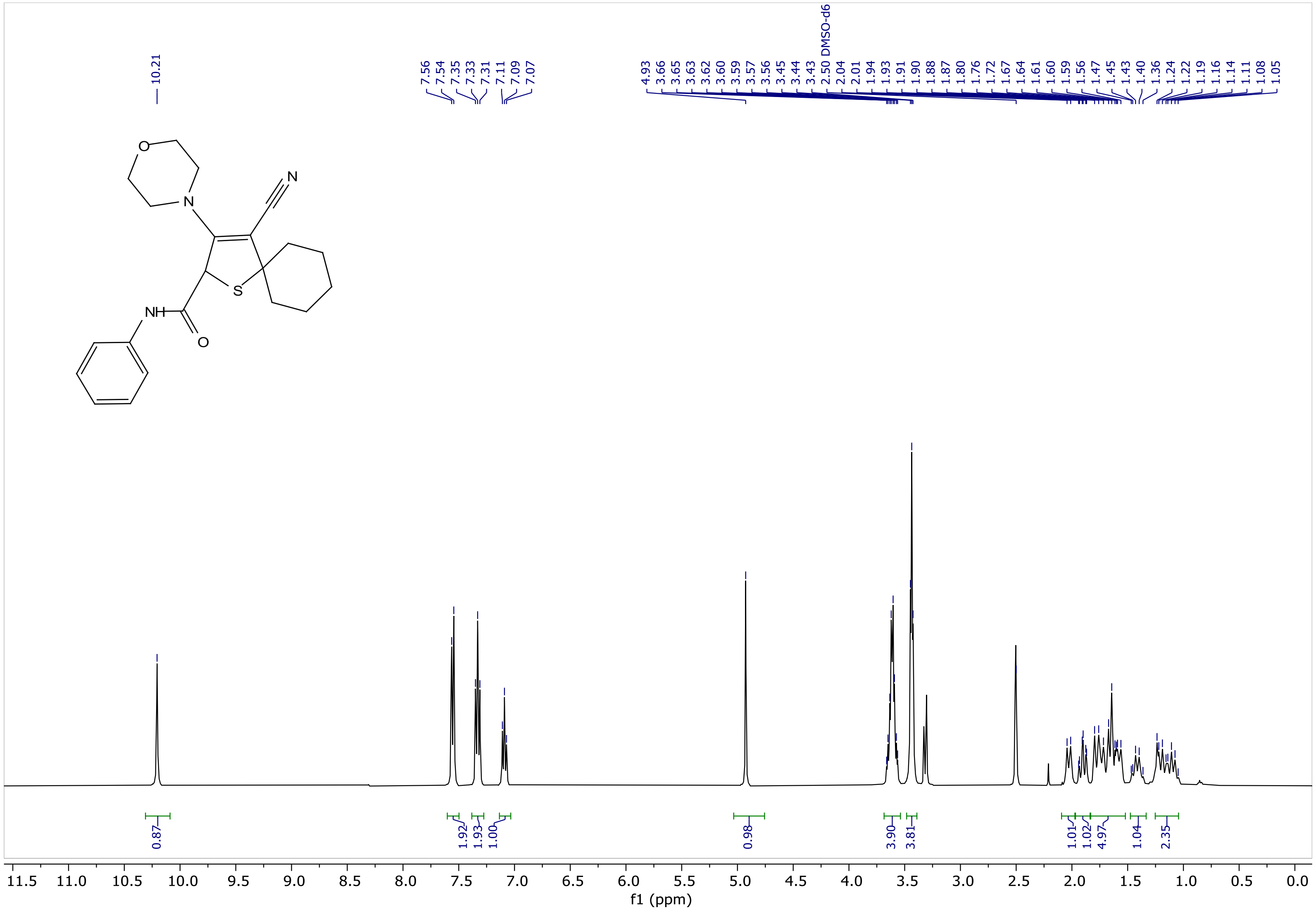

^{1}H NMR (400 MHz, DMSO-d_6) of **3a**

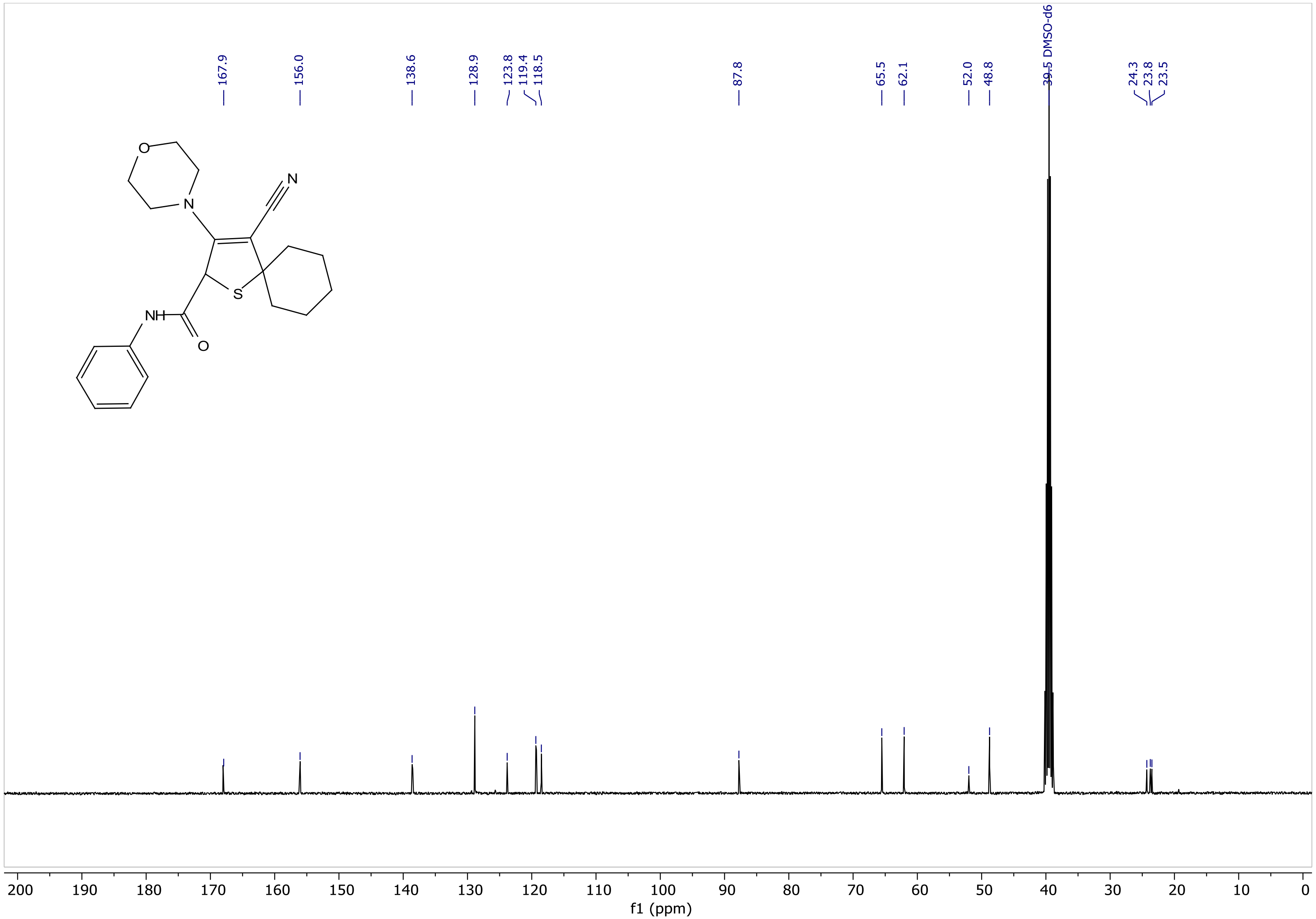

^{13}C NMR (100 MHz, DMSO-d_6) of **3a**

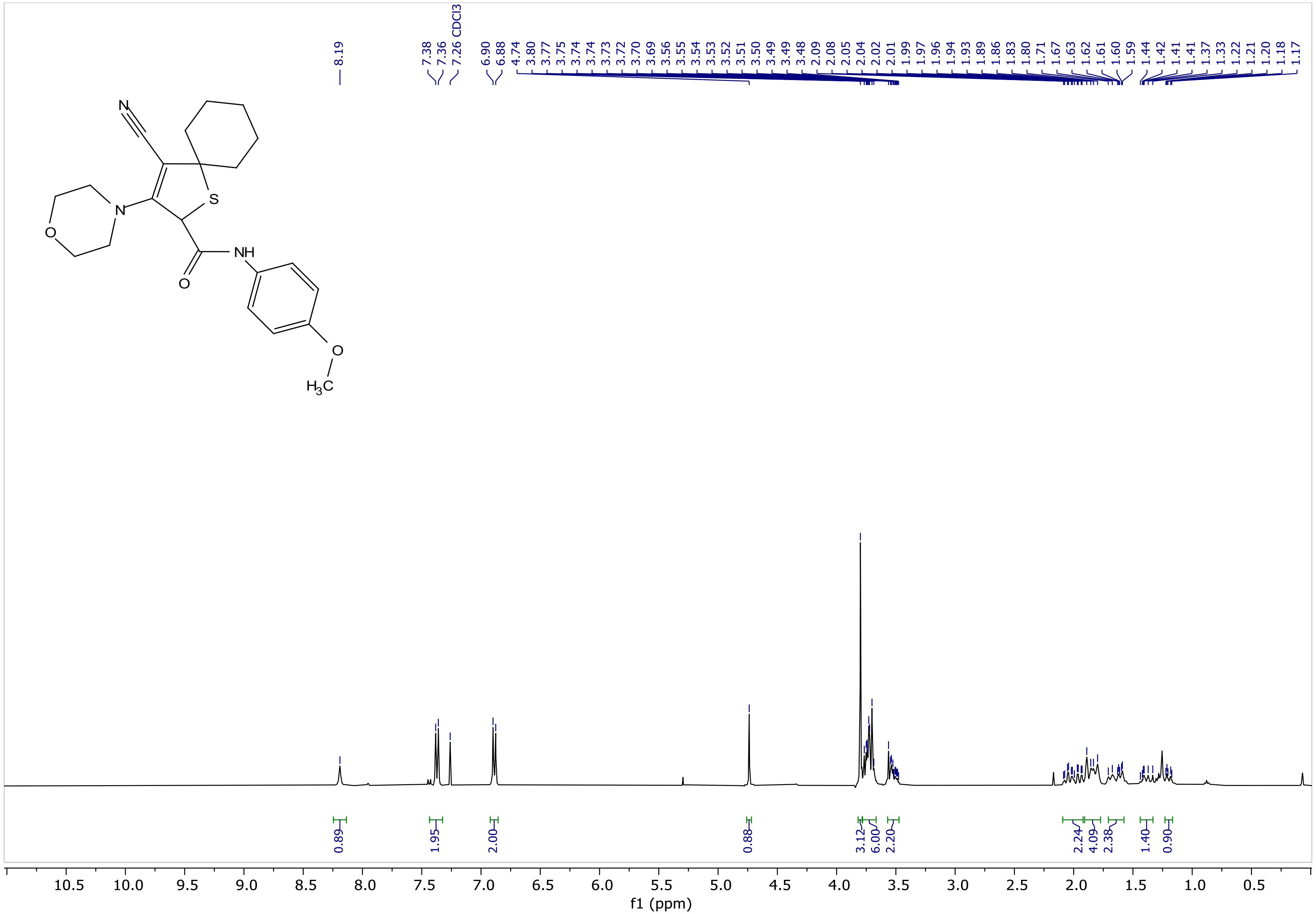

^{1}H NMR (400 MHz, $CDCl_3$-*d*) of **3b**

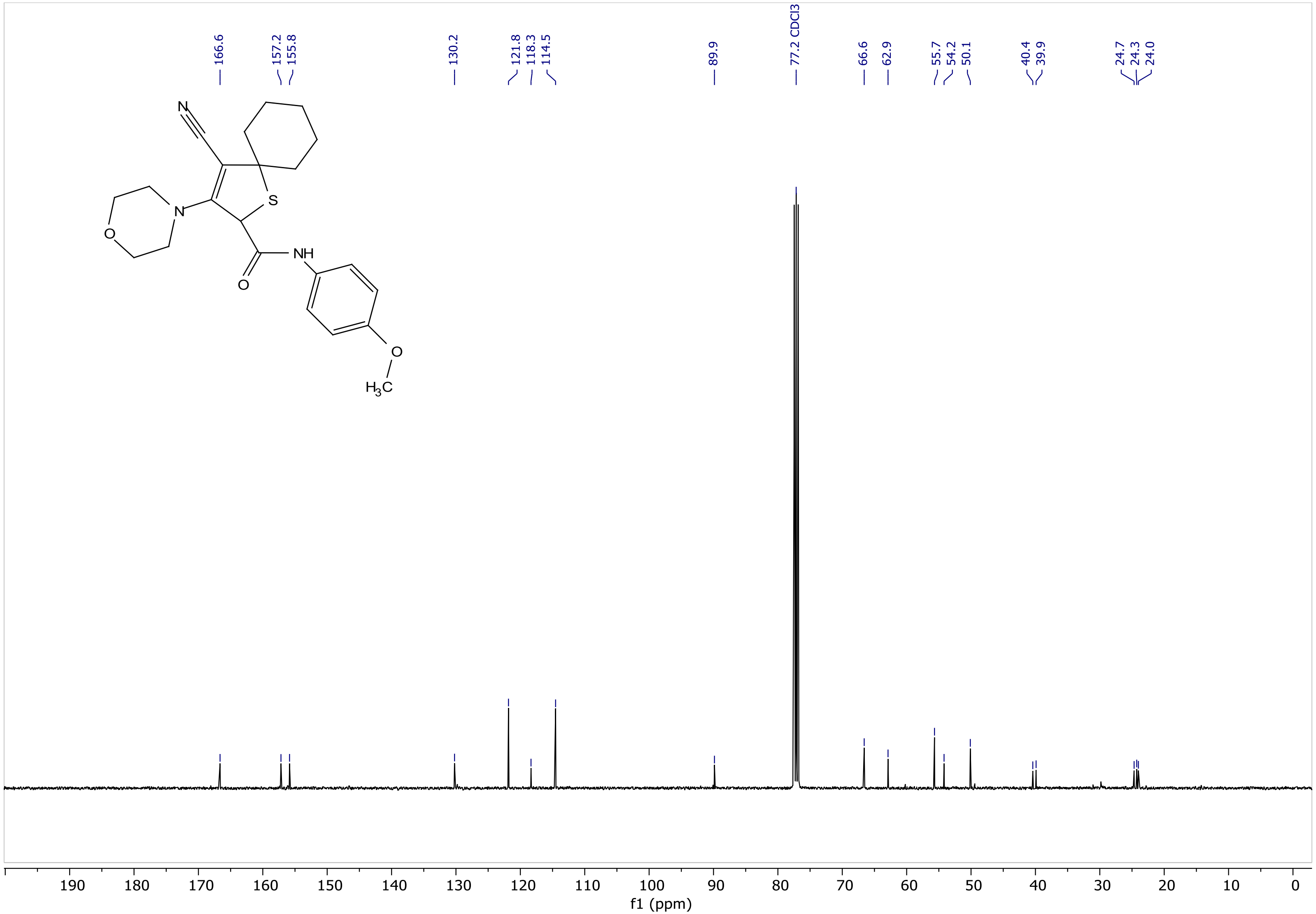

^{13}C NMR (100 MHz, CDCl$_3$-*d*) of **3b**

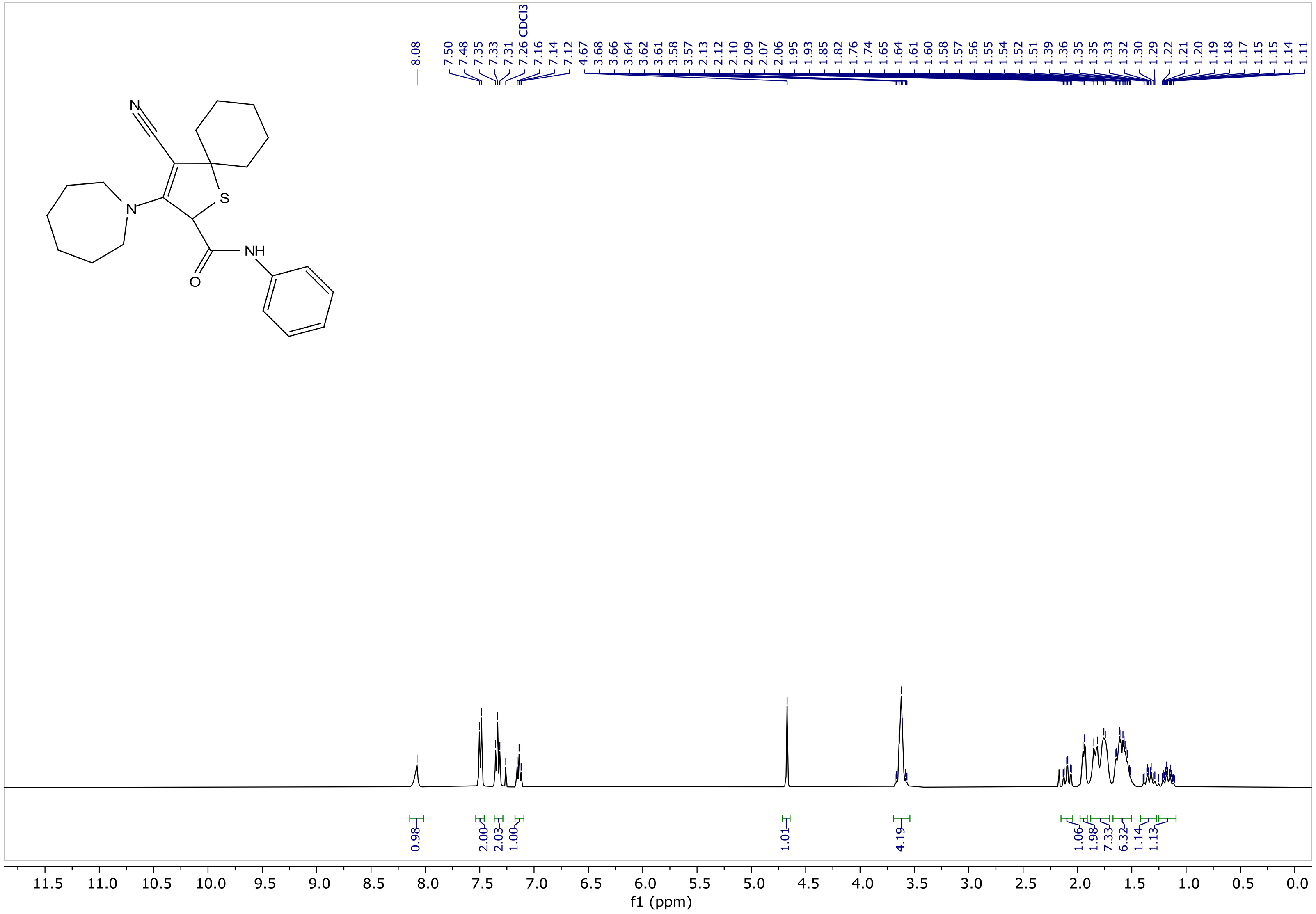

^{1}H NMR (400 MHz, $CDCl_3$-*d*) of **2o**

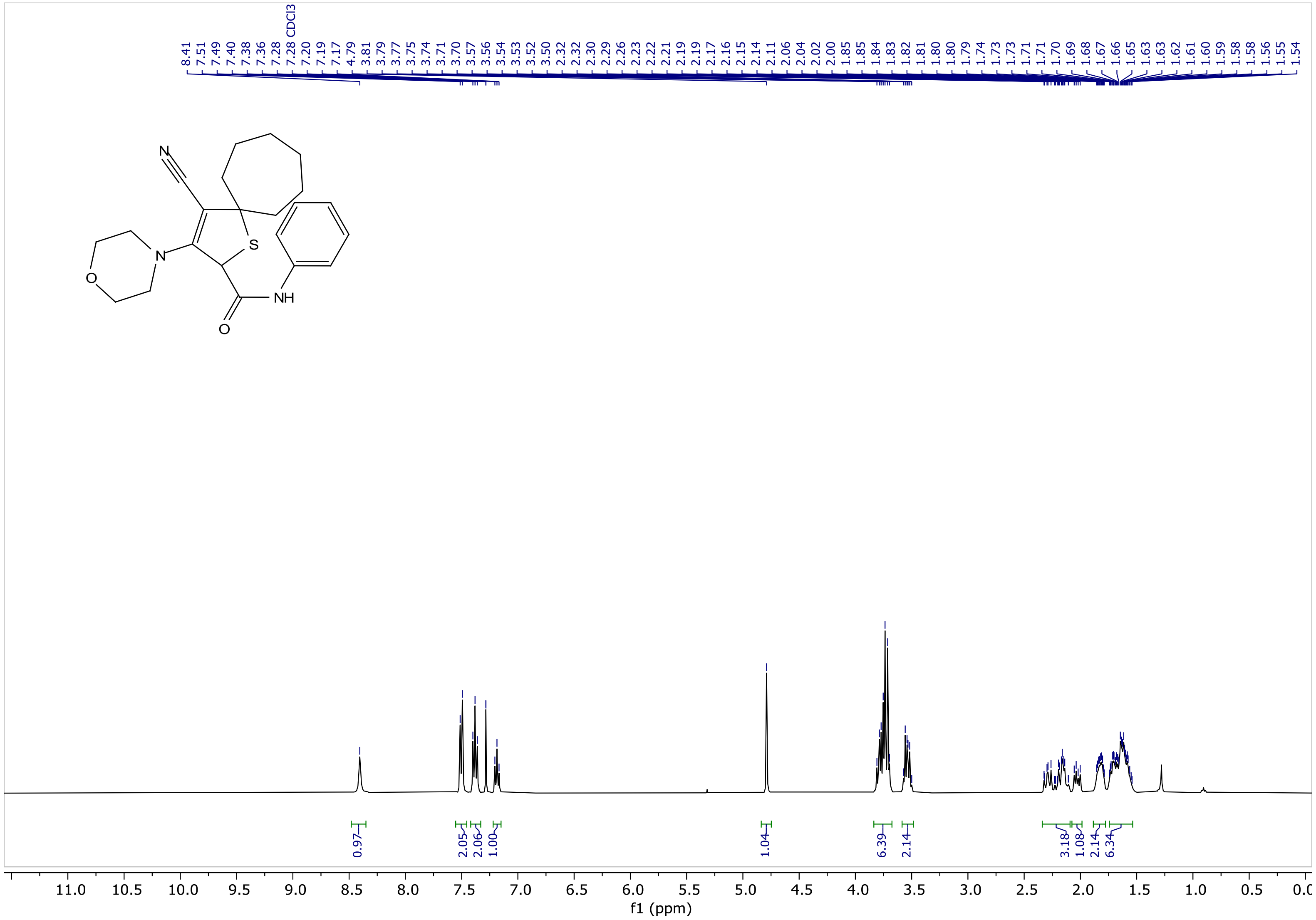

^{1}H NMR (400 MHz, $CDCl_3$-*d*) of **3c**

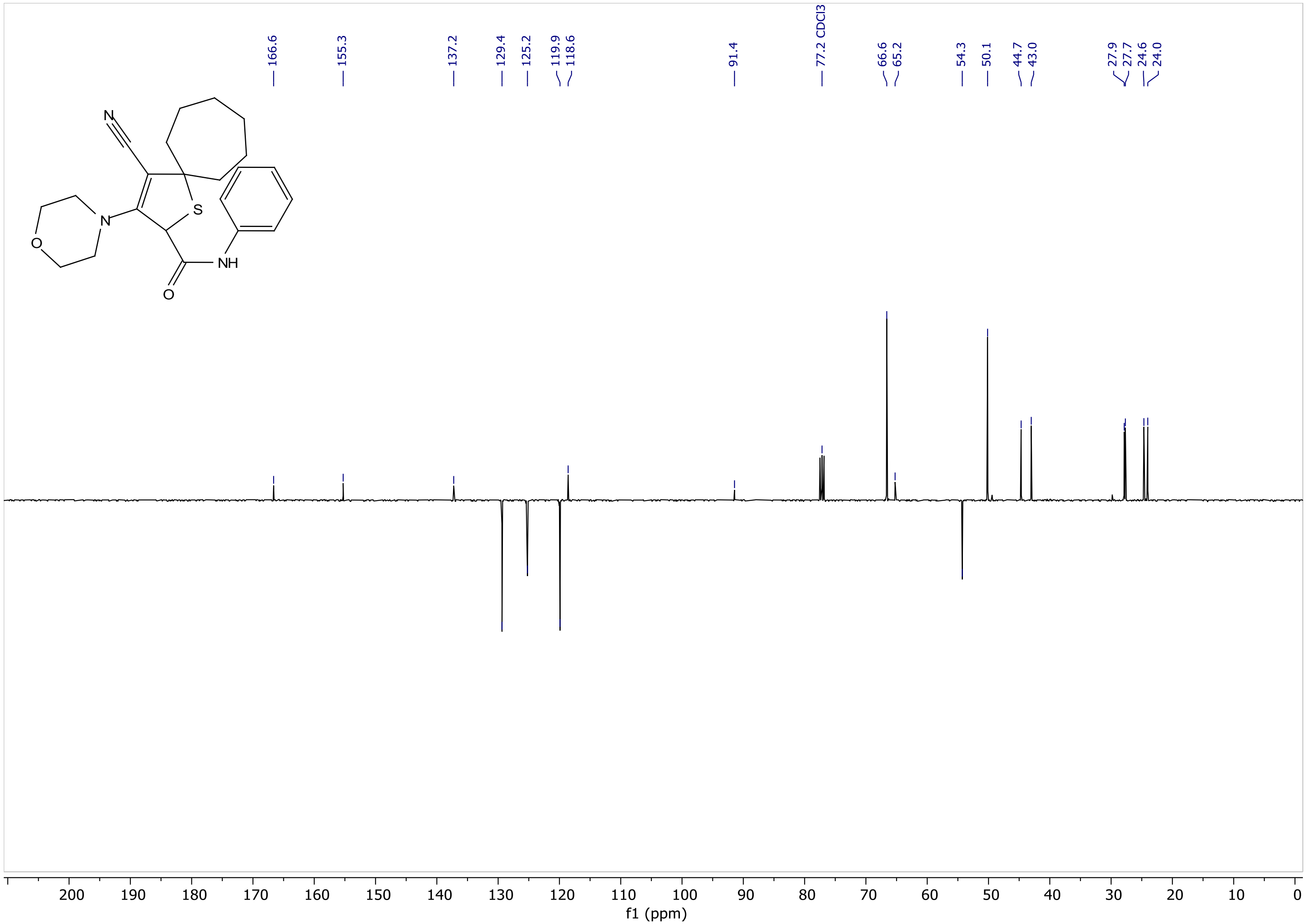

^{13}C NMR (100 MHz, $CDCl_3$-*d*) of **3c**

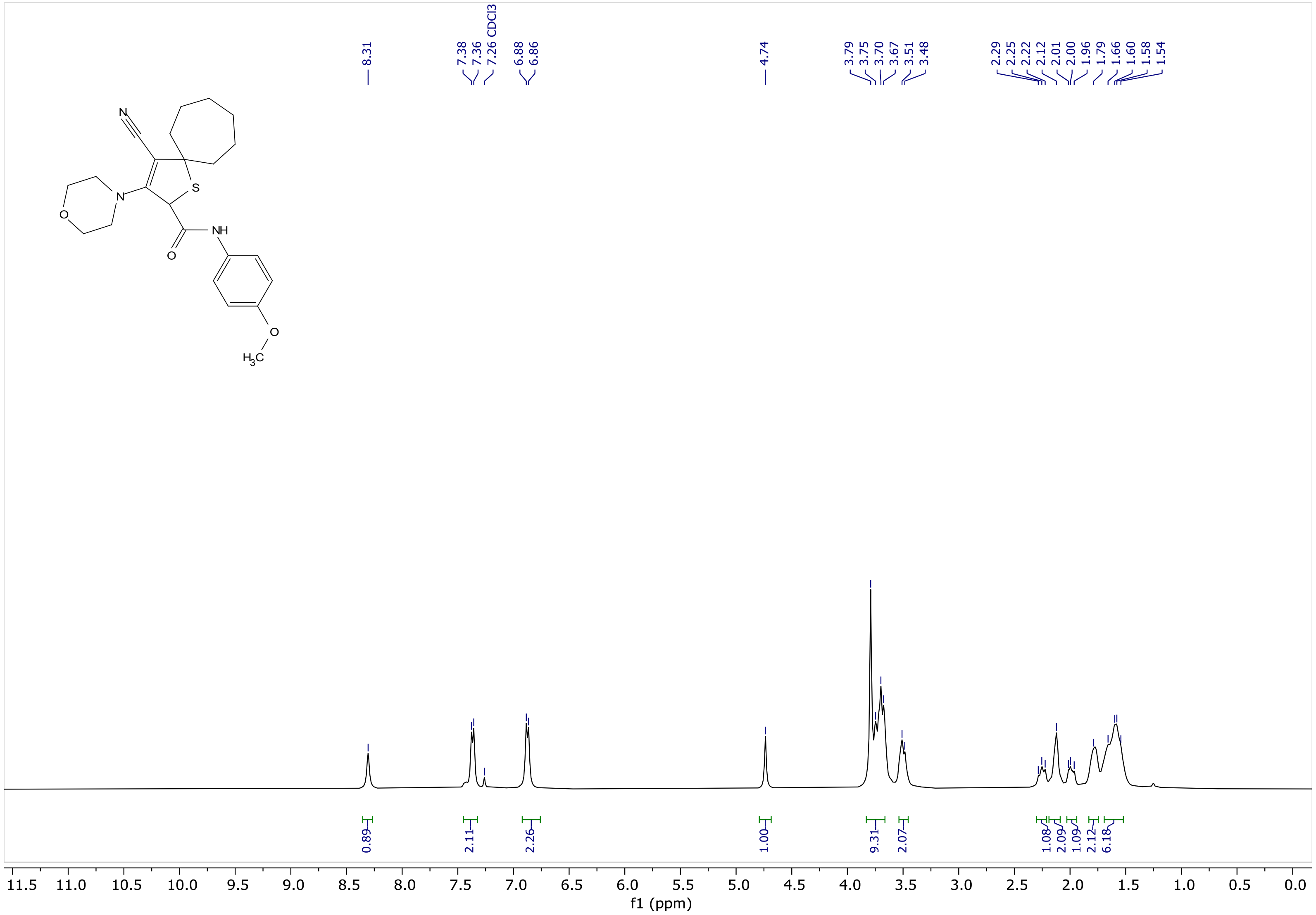

^{1}H NMR (400 MHz, $CDCl_3$-*d*) of **3d**

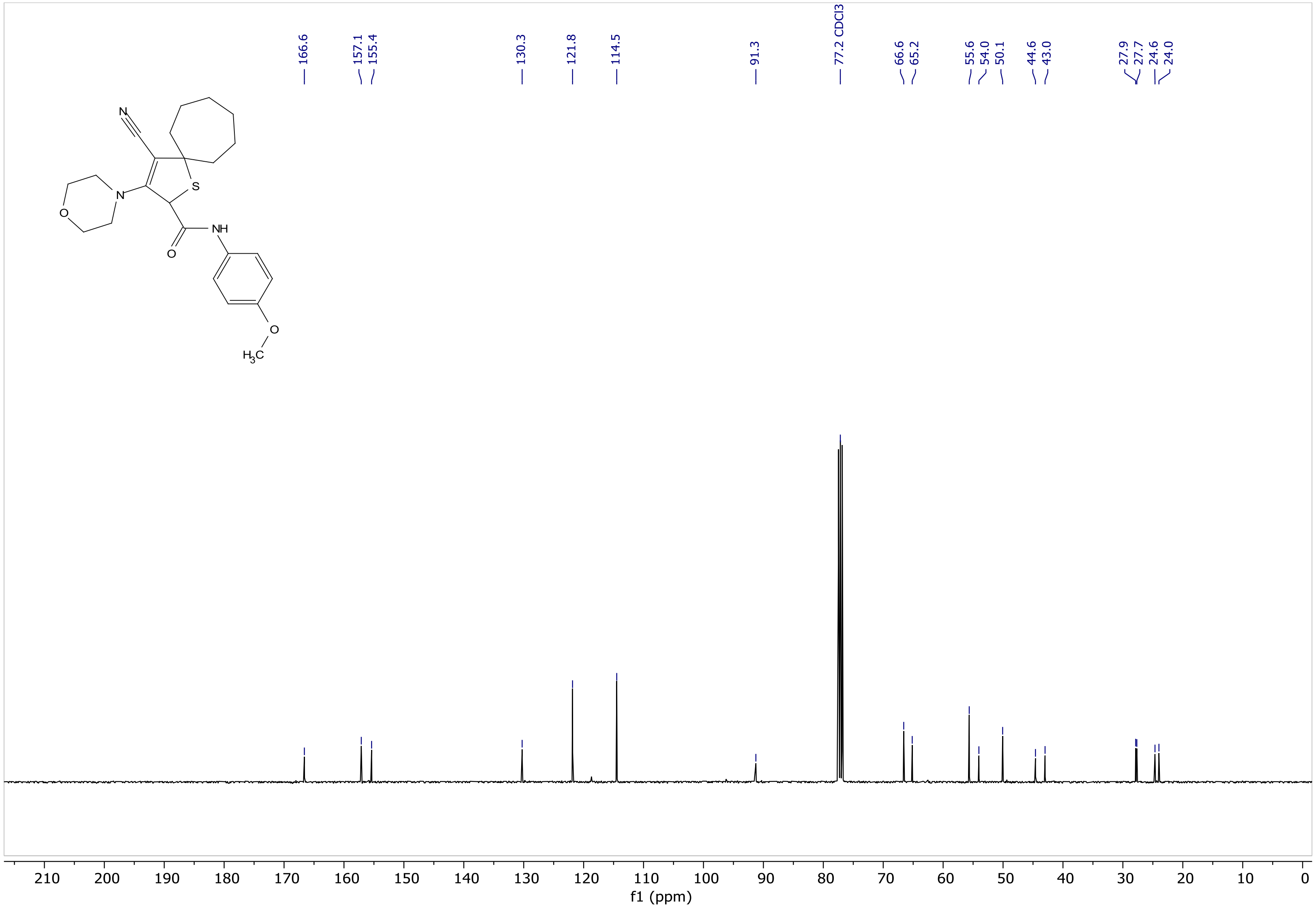

^{13}C NMR (100 MHz, $CDCl_3$-*d*) of **3d**

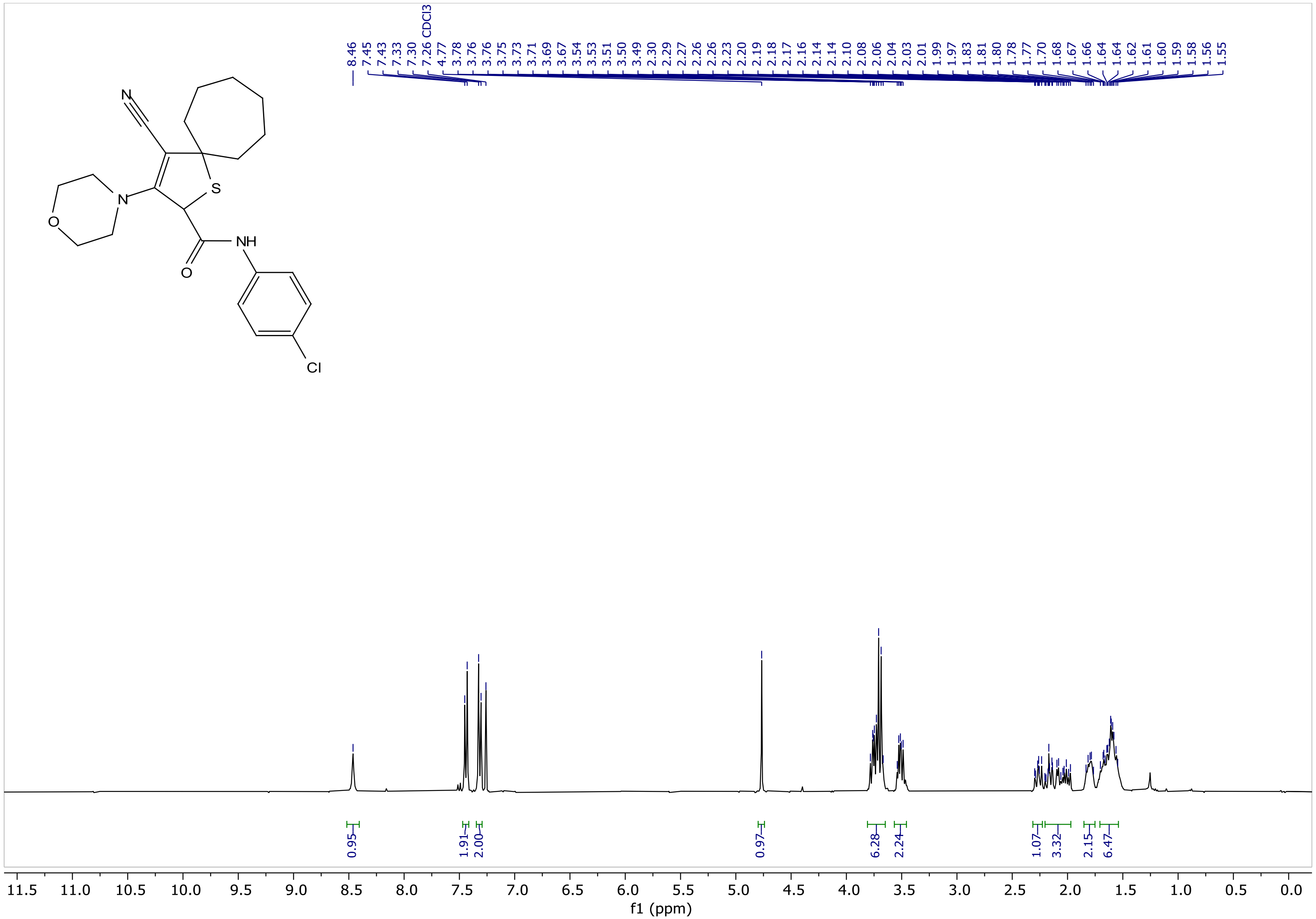

^{1}H NMR (400 MHz, $CDCl_3$-*d*) of **3e**

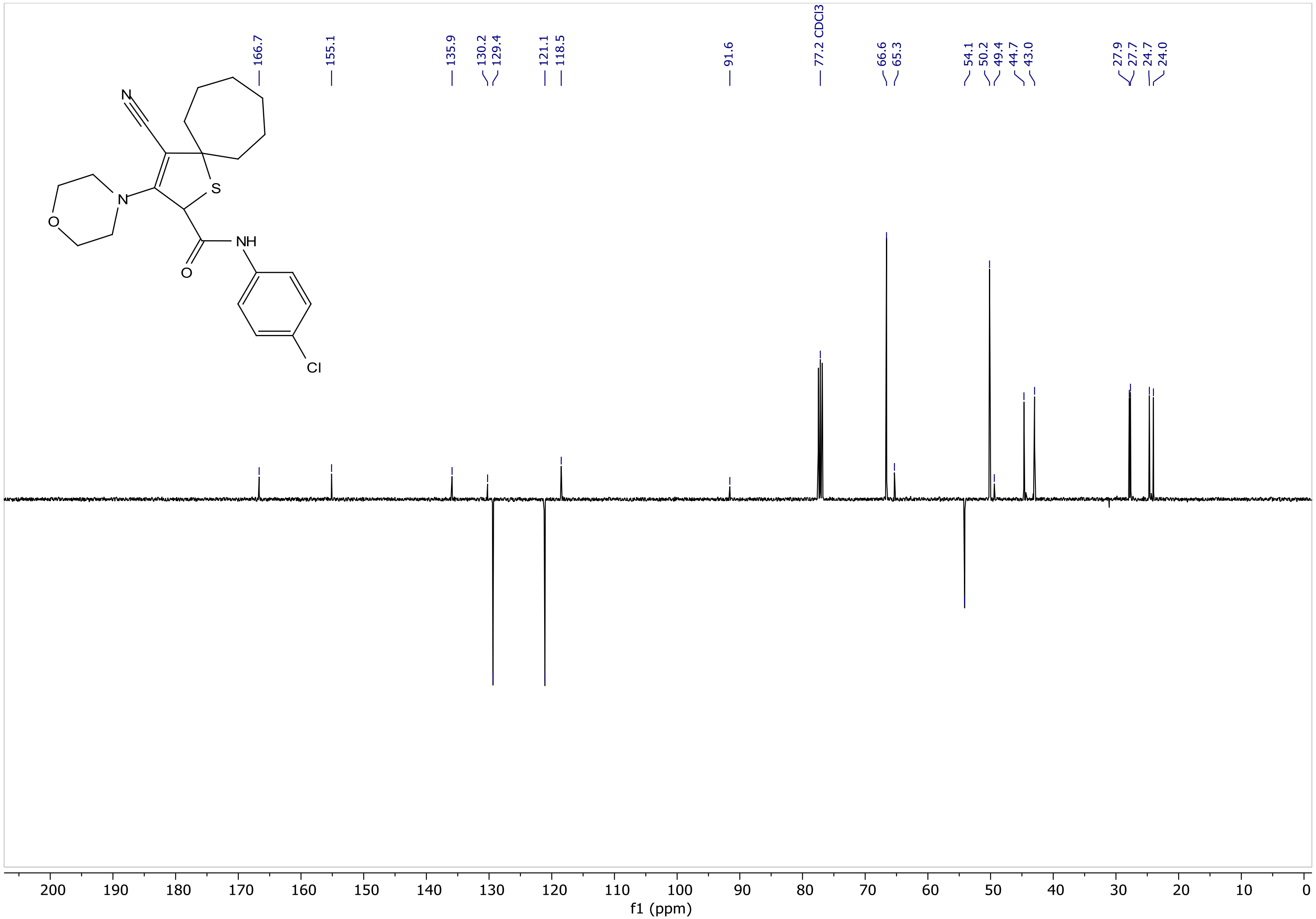

^{13}C NMR (100 MHz, $CDCl_3$-*d*) of **3e**

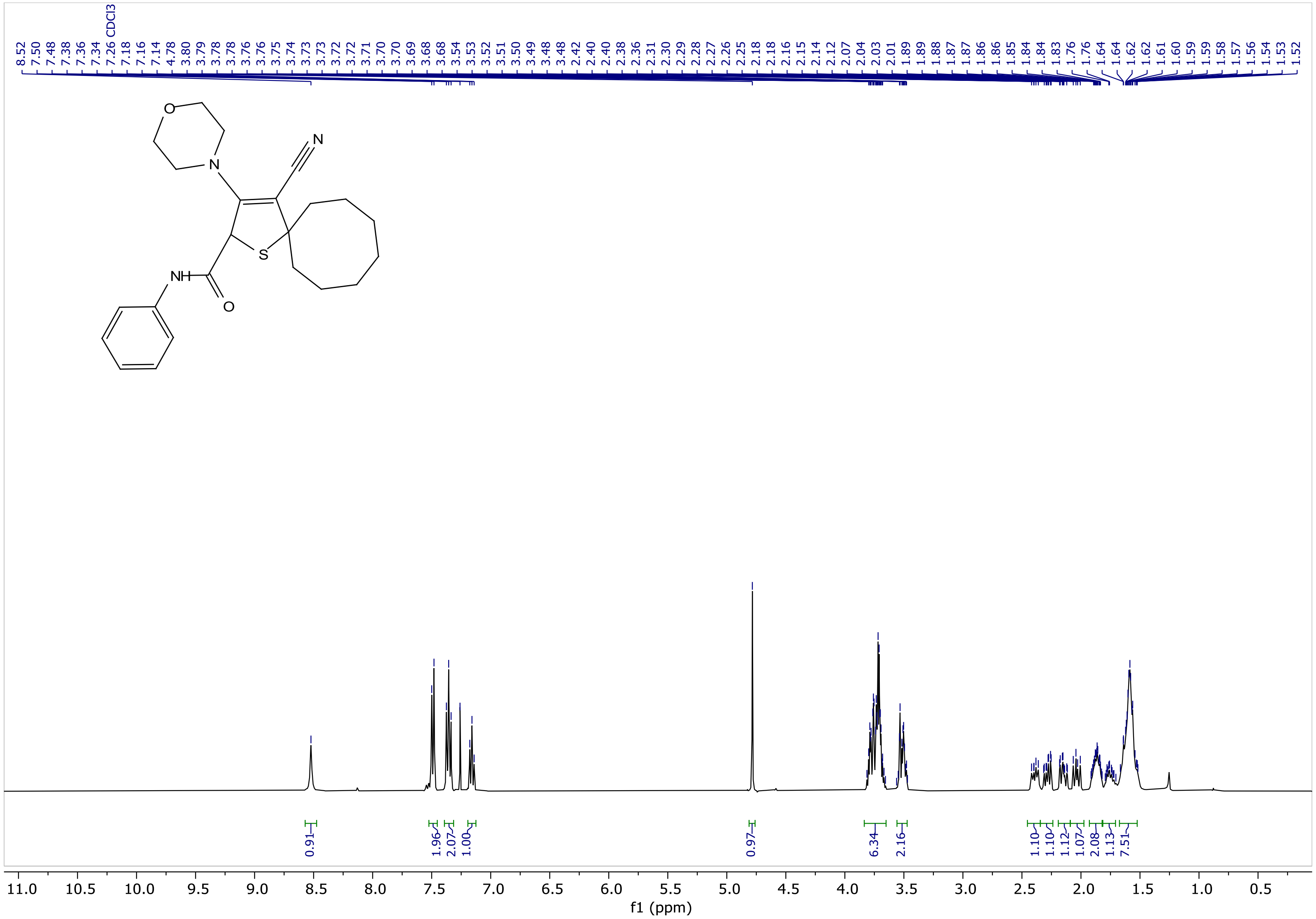

^{1}H NMR (400 MHz, $CDCl_3$-*d*) of **3f**

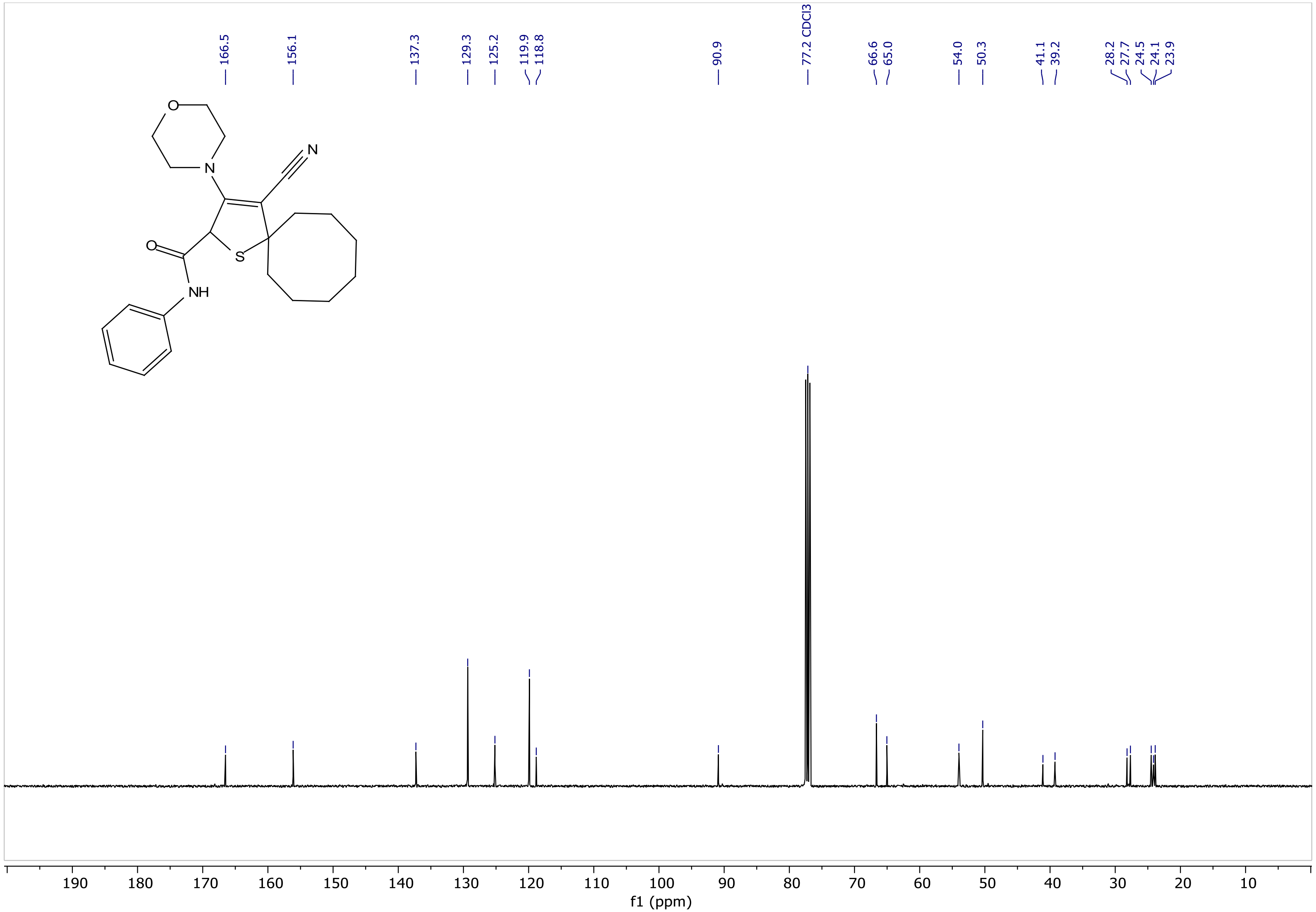

^{13}C NMR (100 MHz, $CDCl_3$-*d*) of **3f**

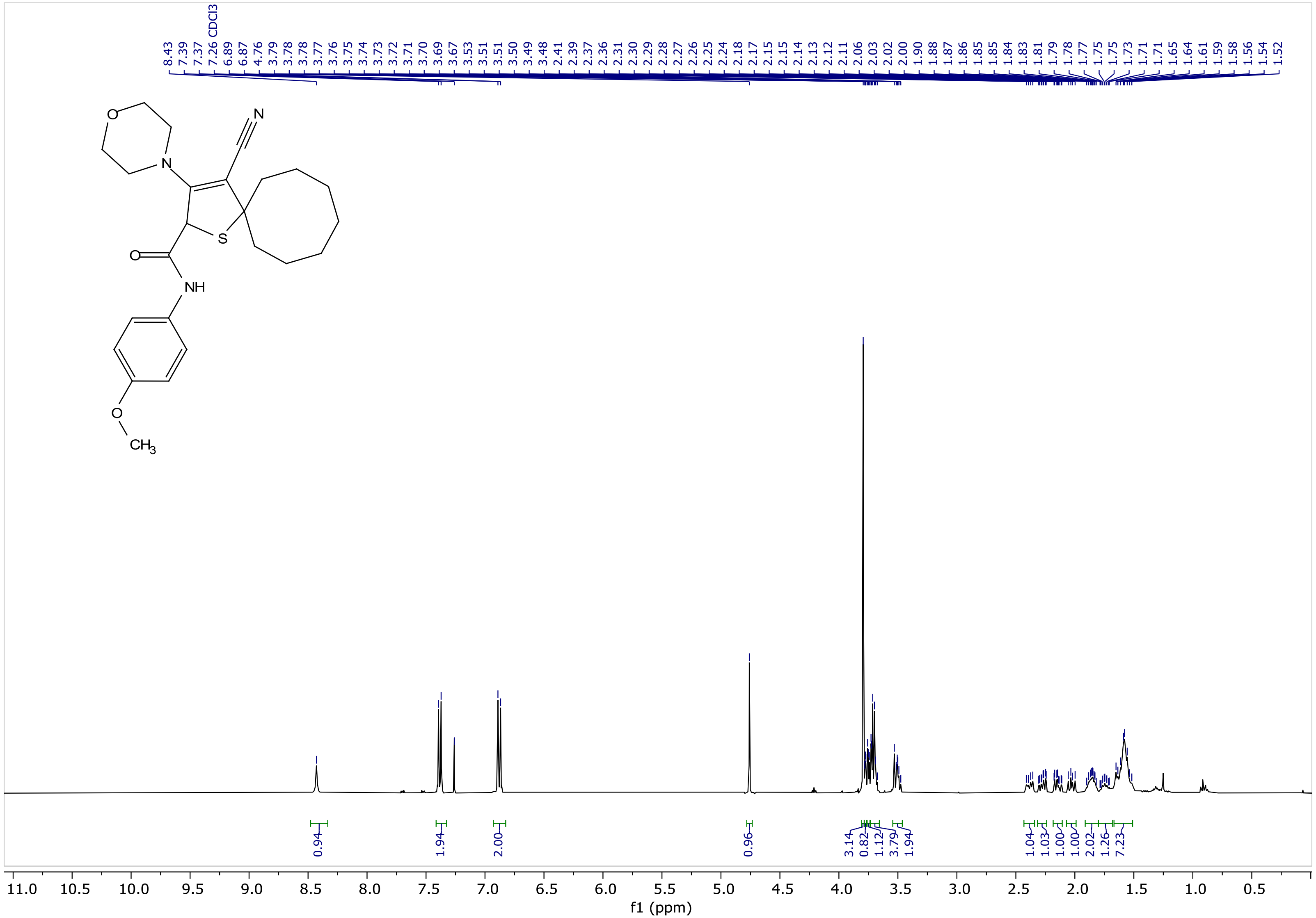

^{1}H NMR (400 MHz, $CDCl_3$-*d*) of **3g**

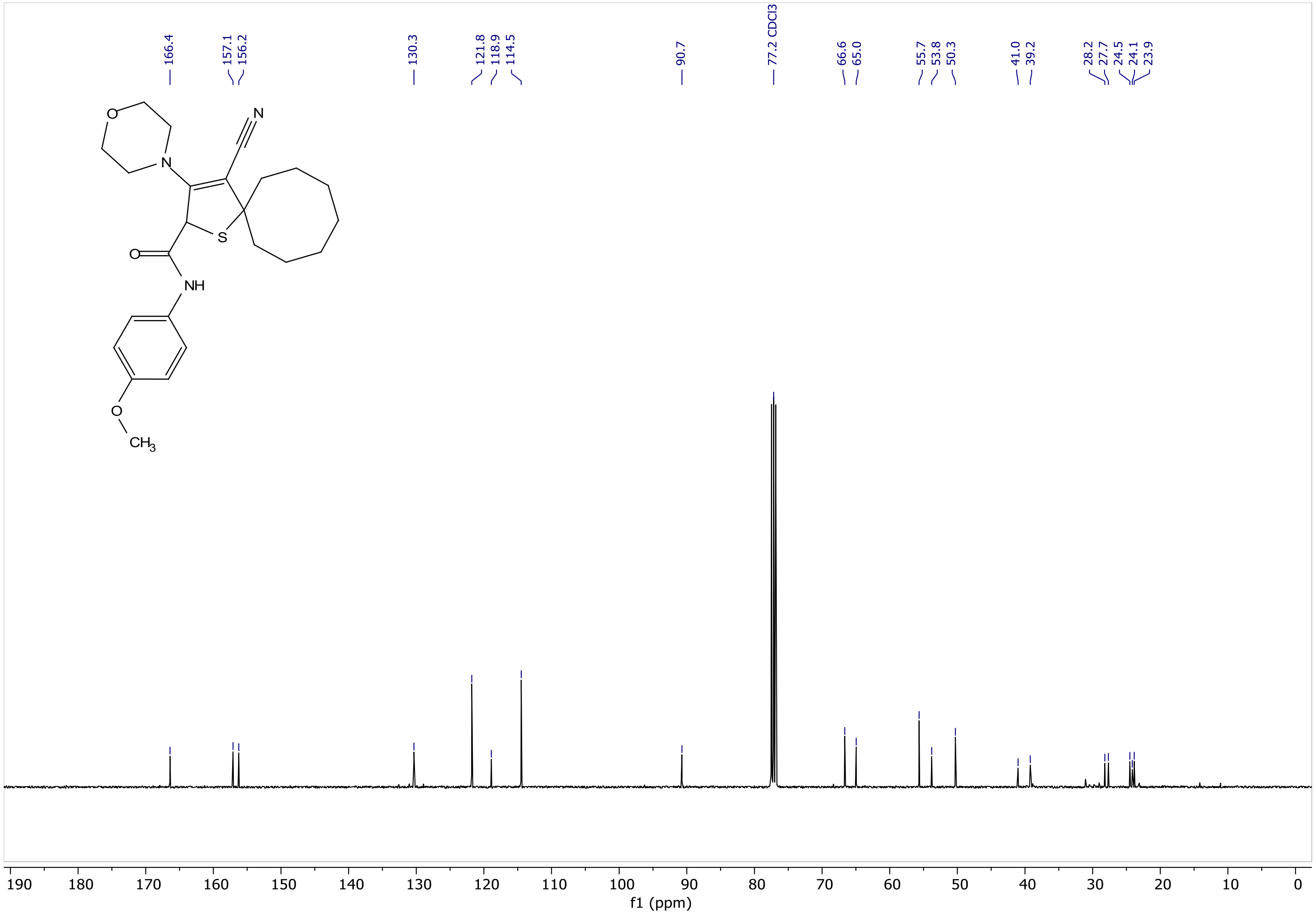

^{13}C NMR (100 MHz, $CDCl_3$-*d*) of **3g**

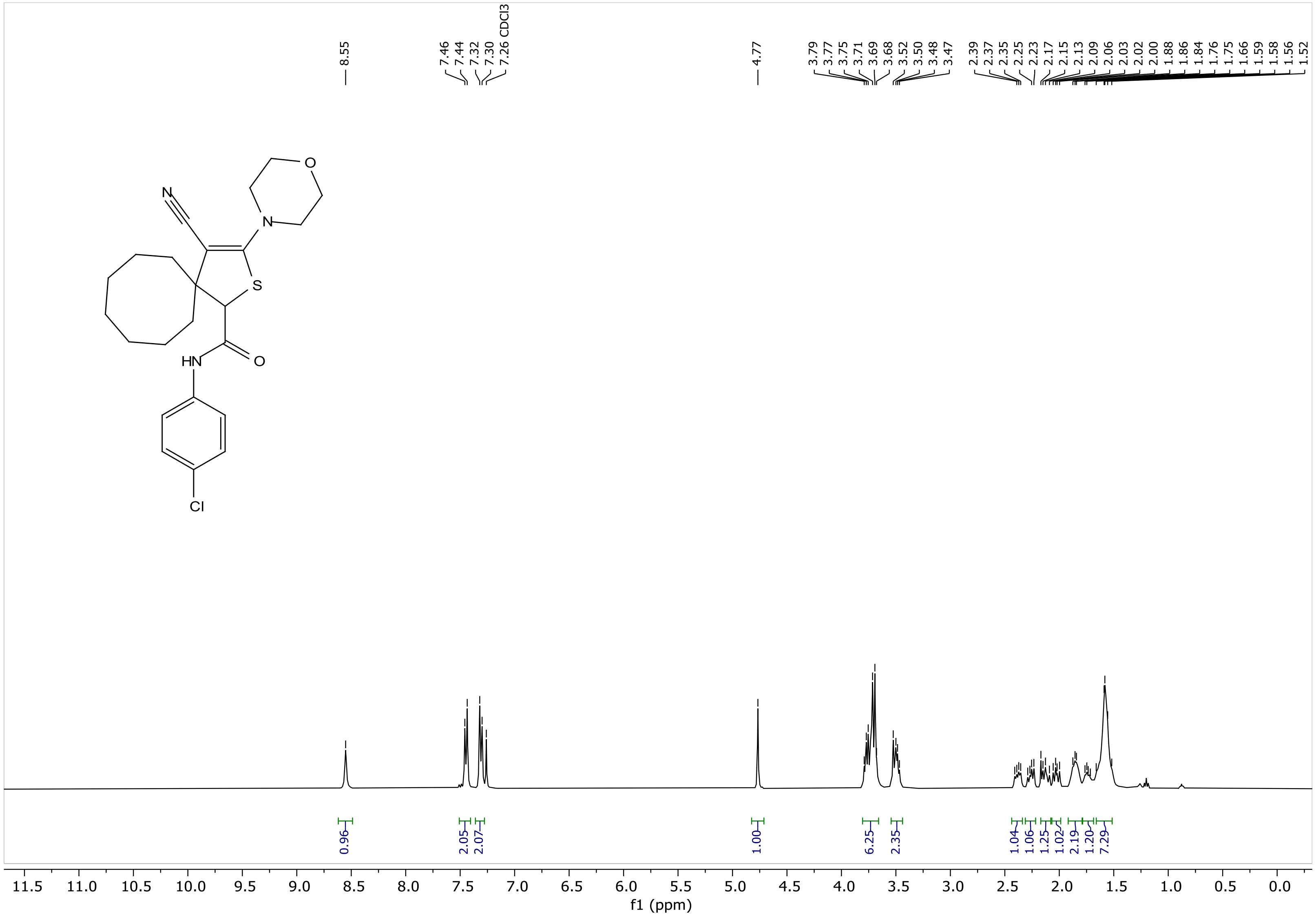

^{1}H NMR (400 MHz, $CDCl_3$-*d*) of **3h**

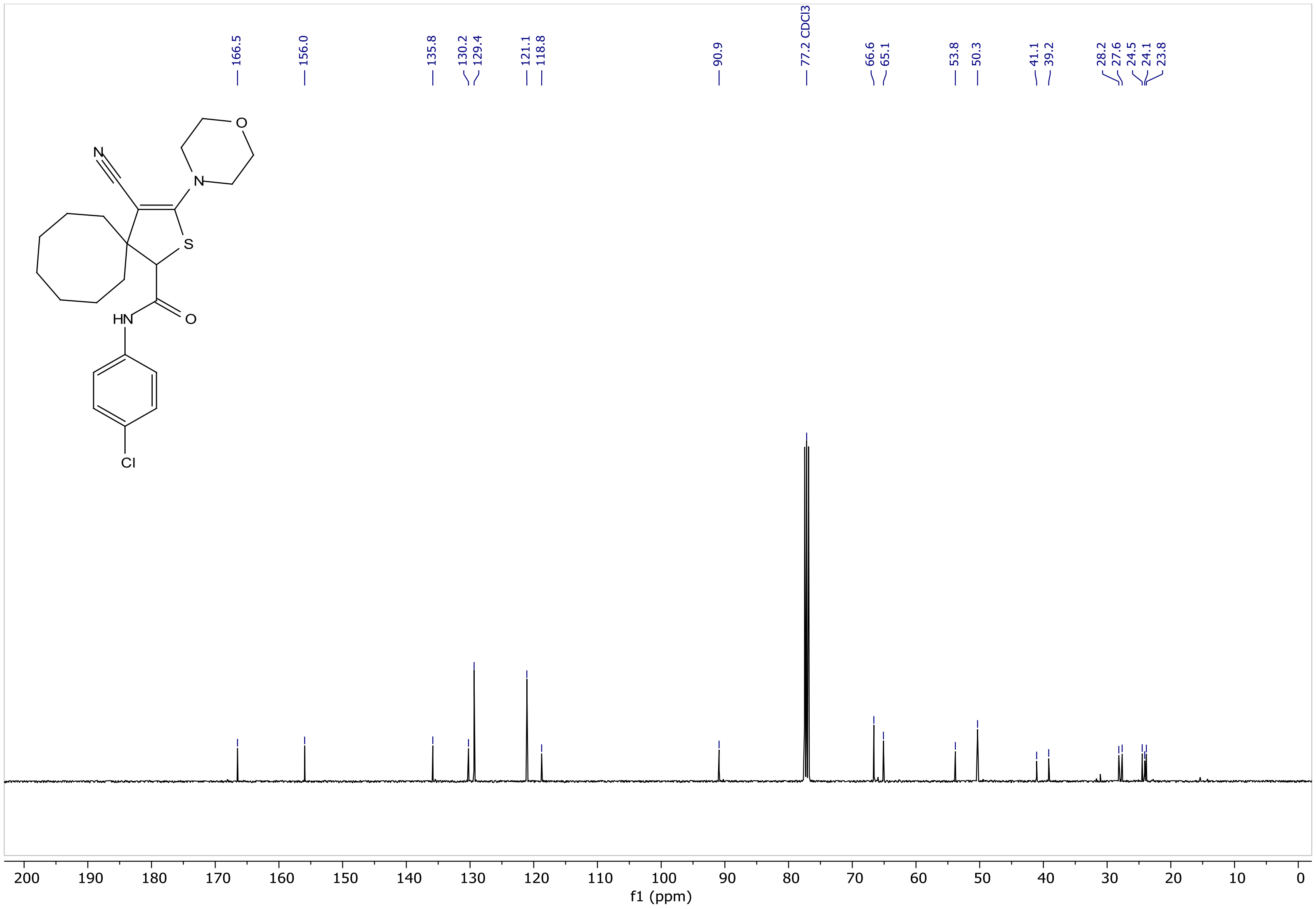

^{13}C NMR (100 MHz, $CDCl_3$-*d*) of **3h**

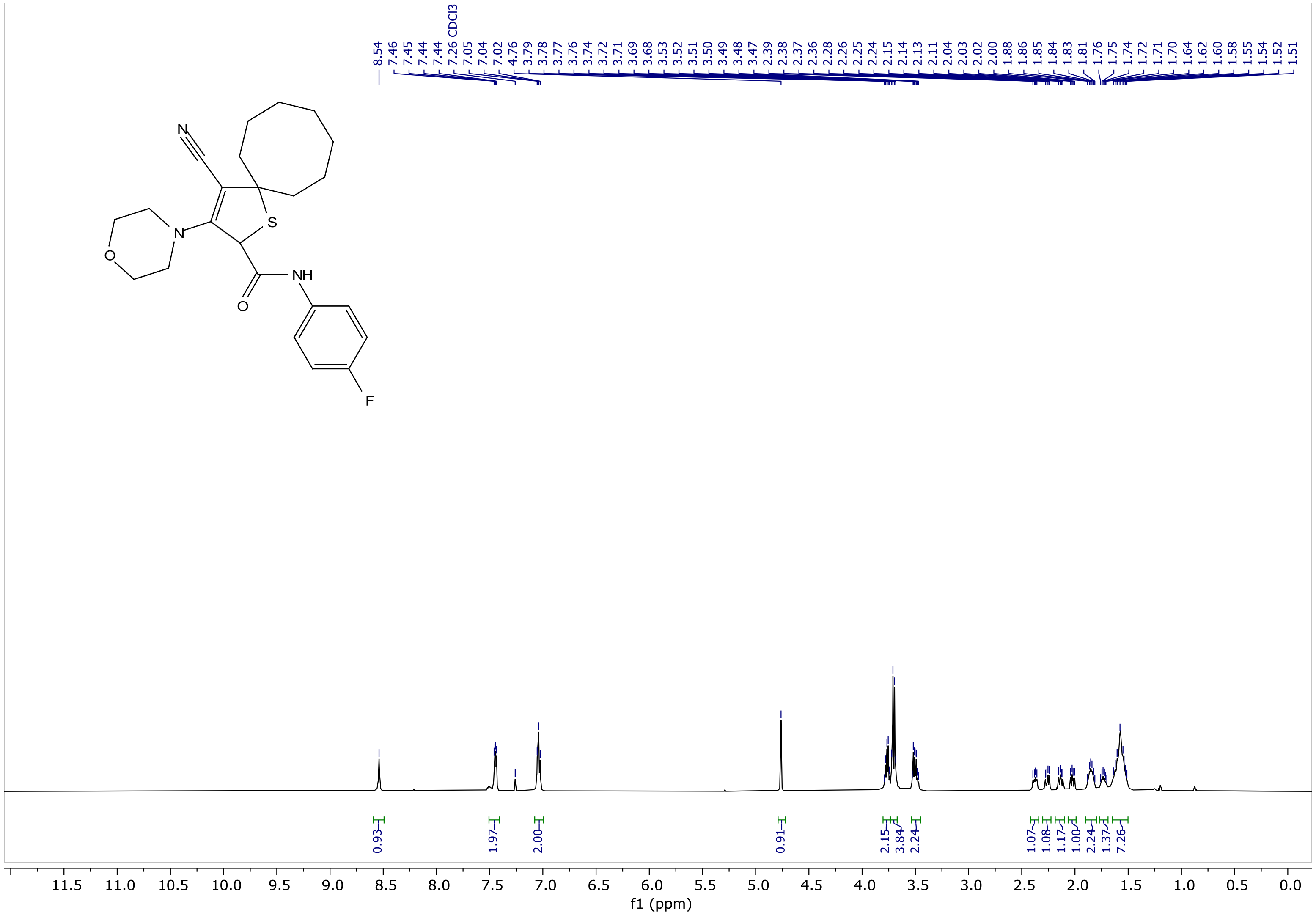

^{1}H NMR (600 MHz, $CDCl_3$-*d*) of **3i**

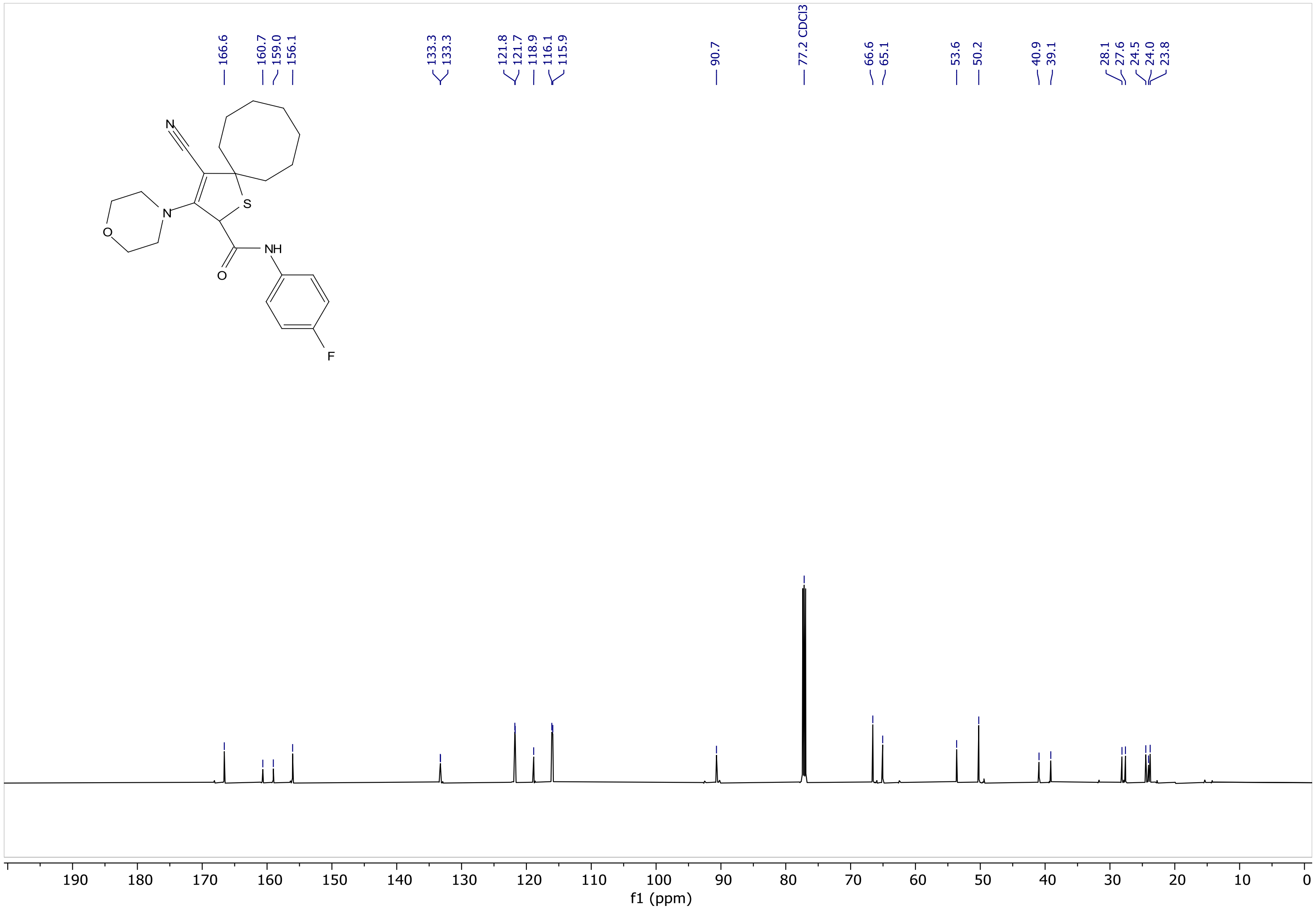

^{13}C NMR (150 MHz, $CDCl_3$-*d*) of **3i**

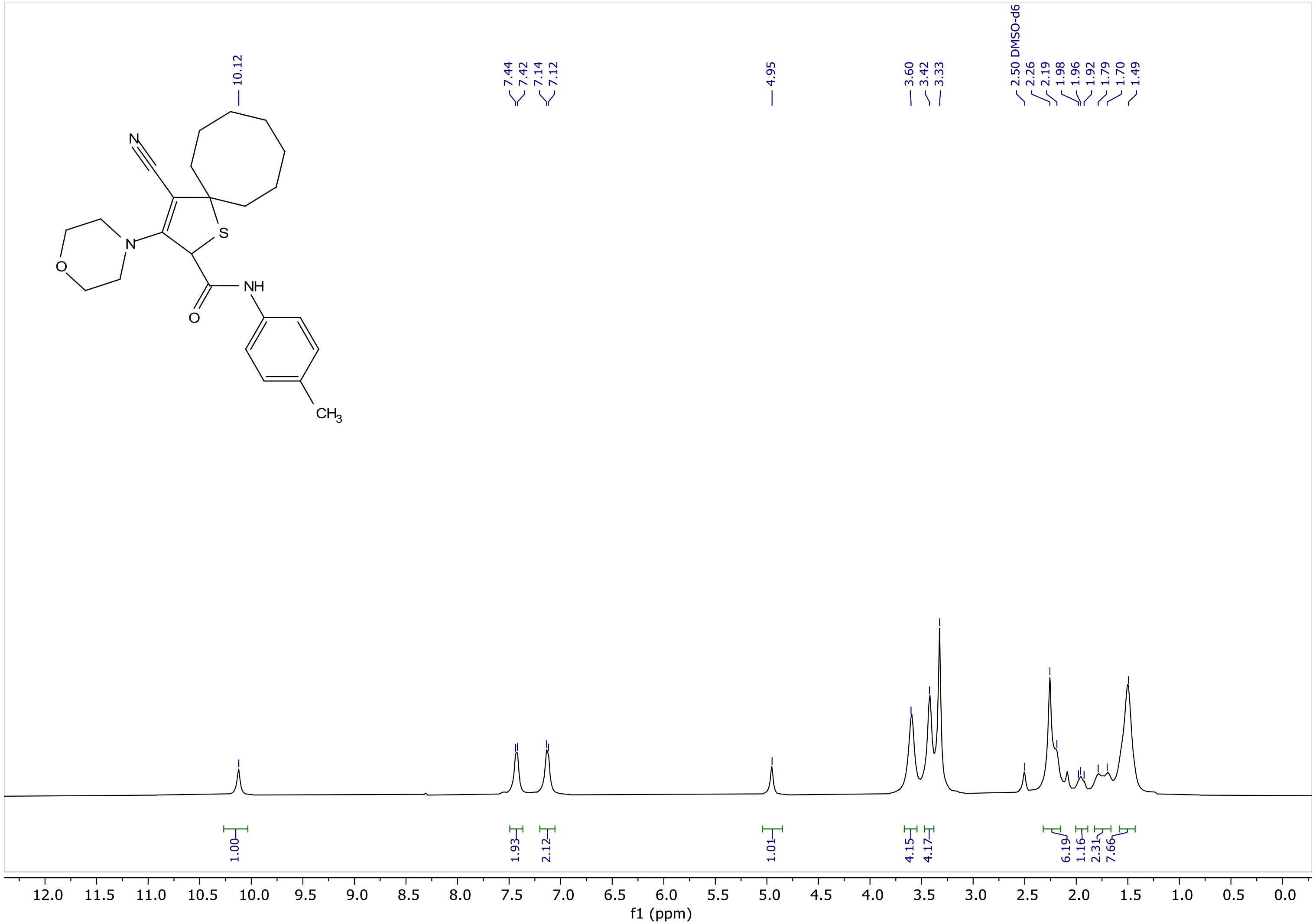

^{1}H NMR (400 MHz, DMSO-d_6) of **3j**

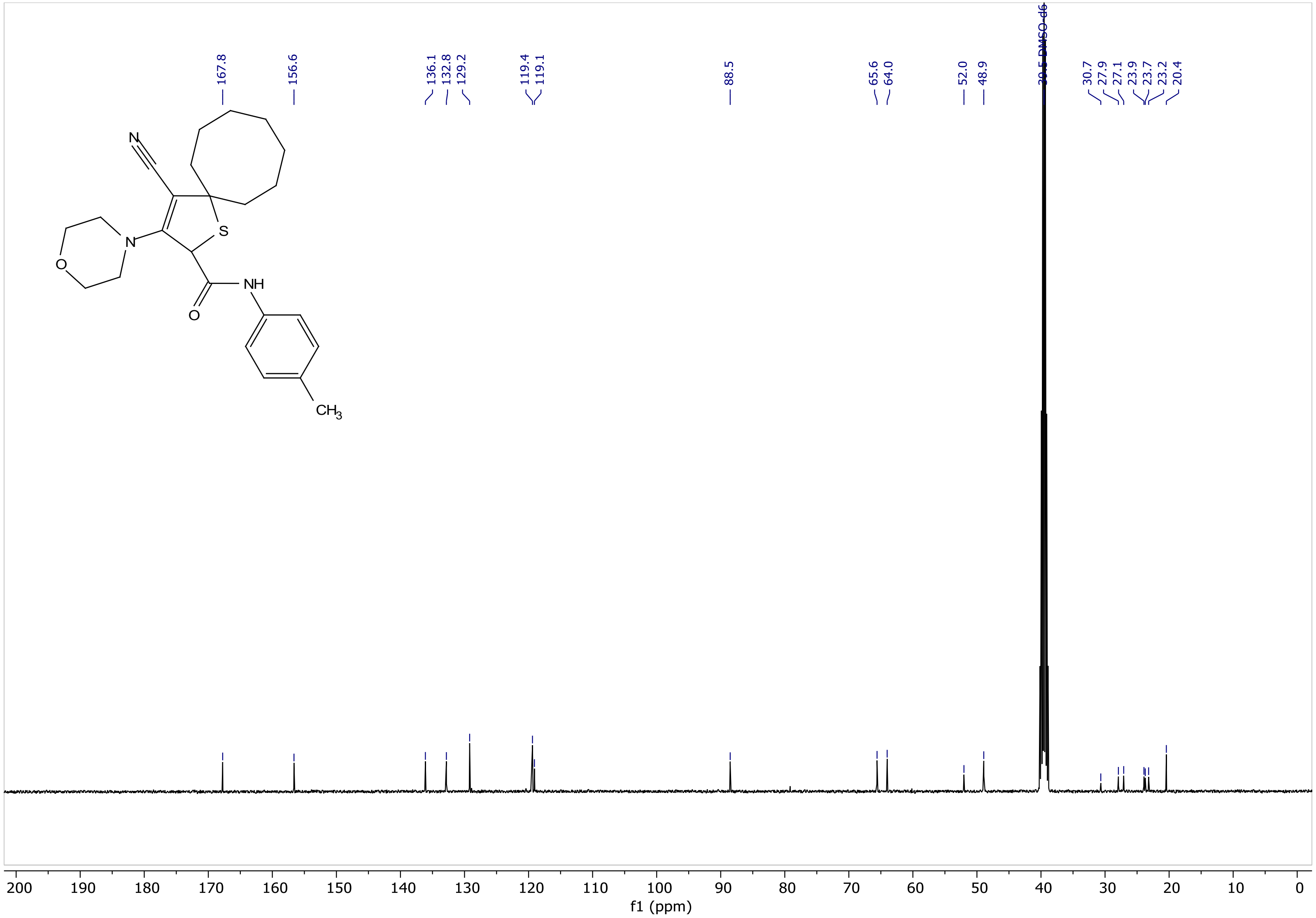

^{13}C NMR (100 MHz, DMSO-d_6) of **3j**

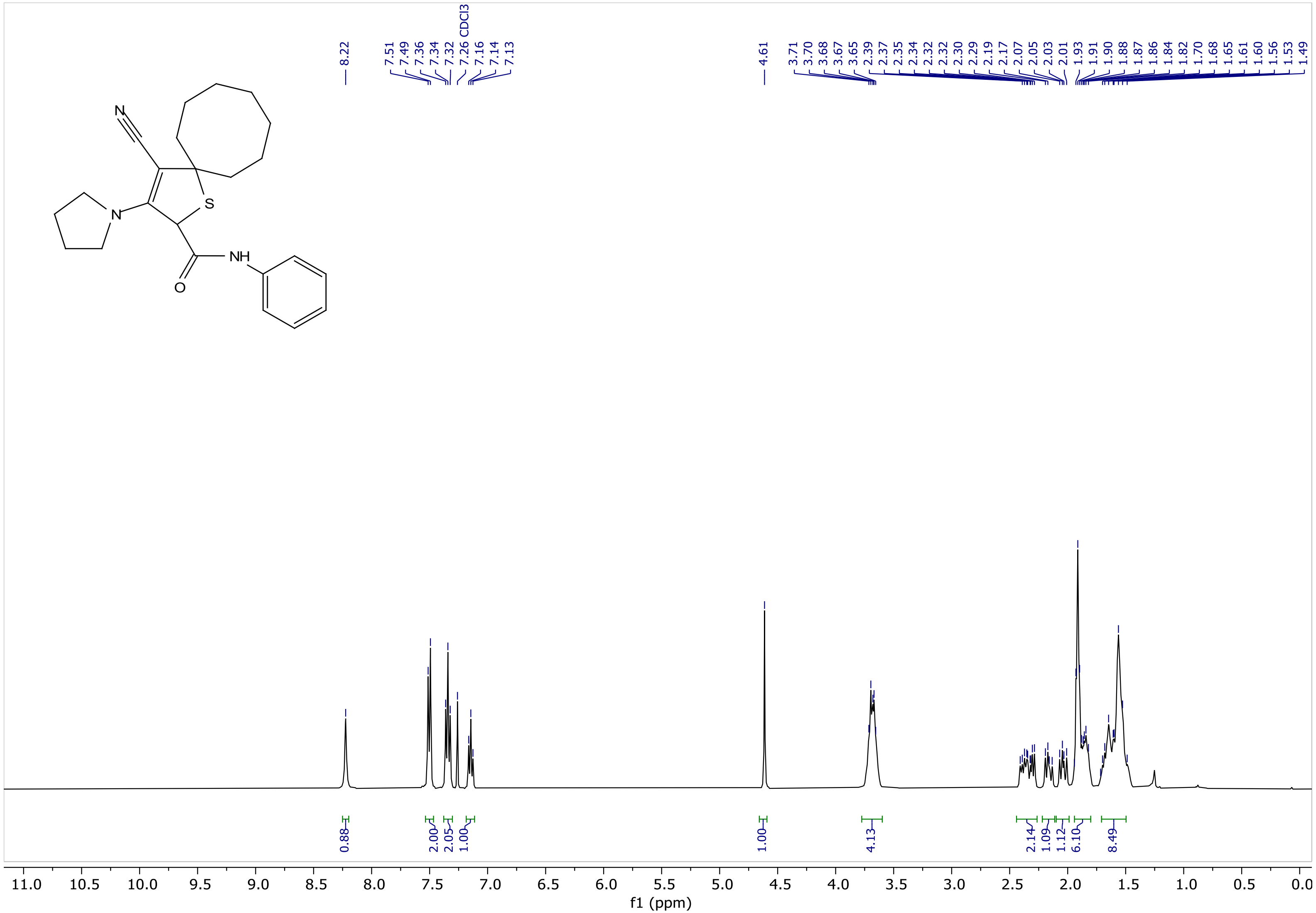

^{1}H NMR (400 MHz, $CDCl_3$-*d*) of **2m**

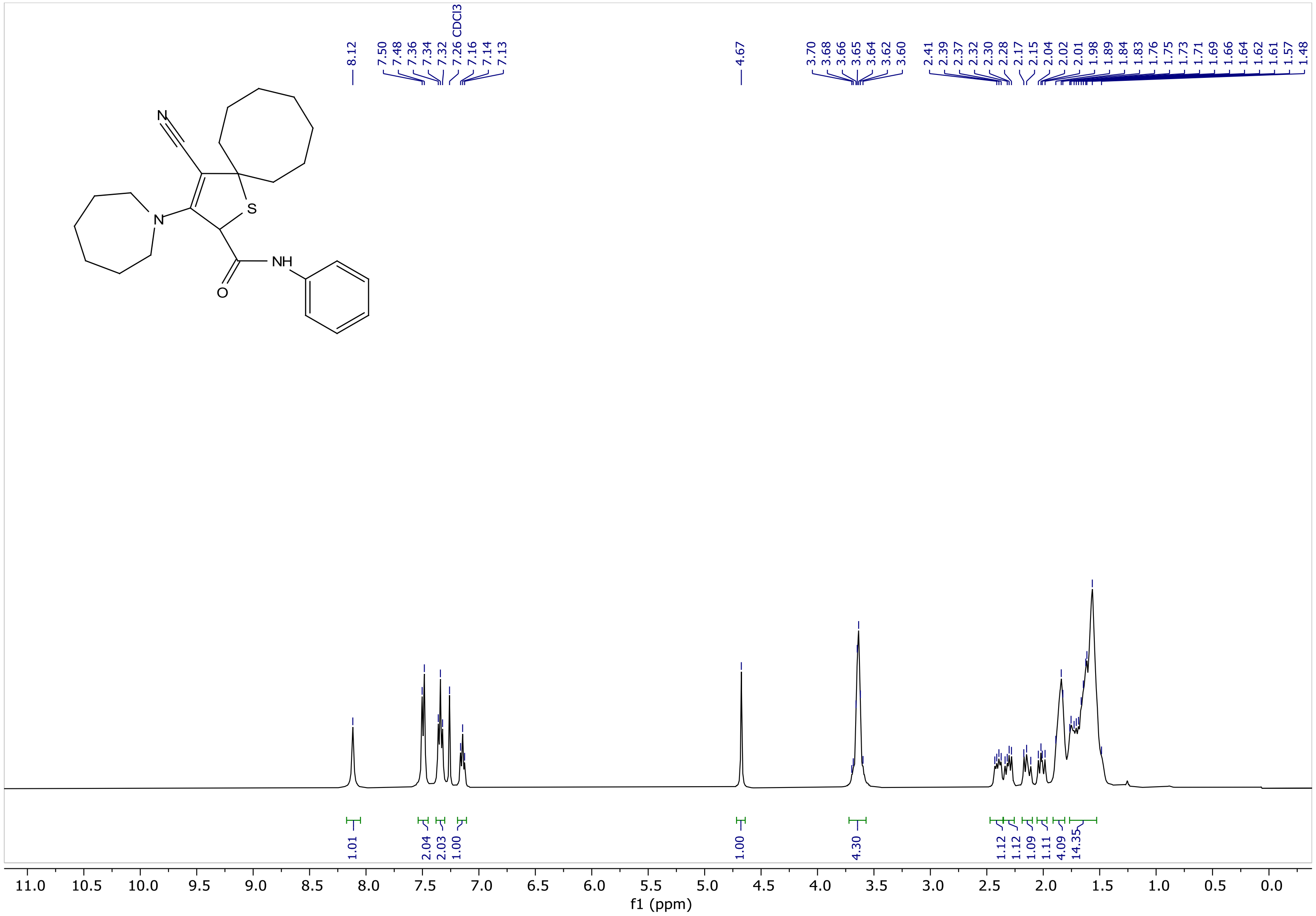

^{1}H NMR (400 MHz, $CDCl_3$-*d*) of **2n**

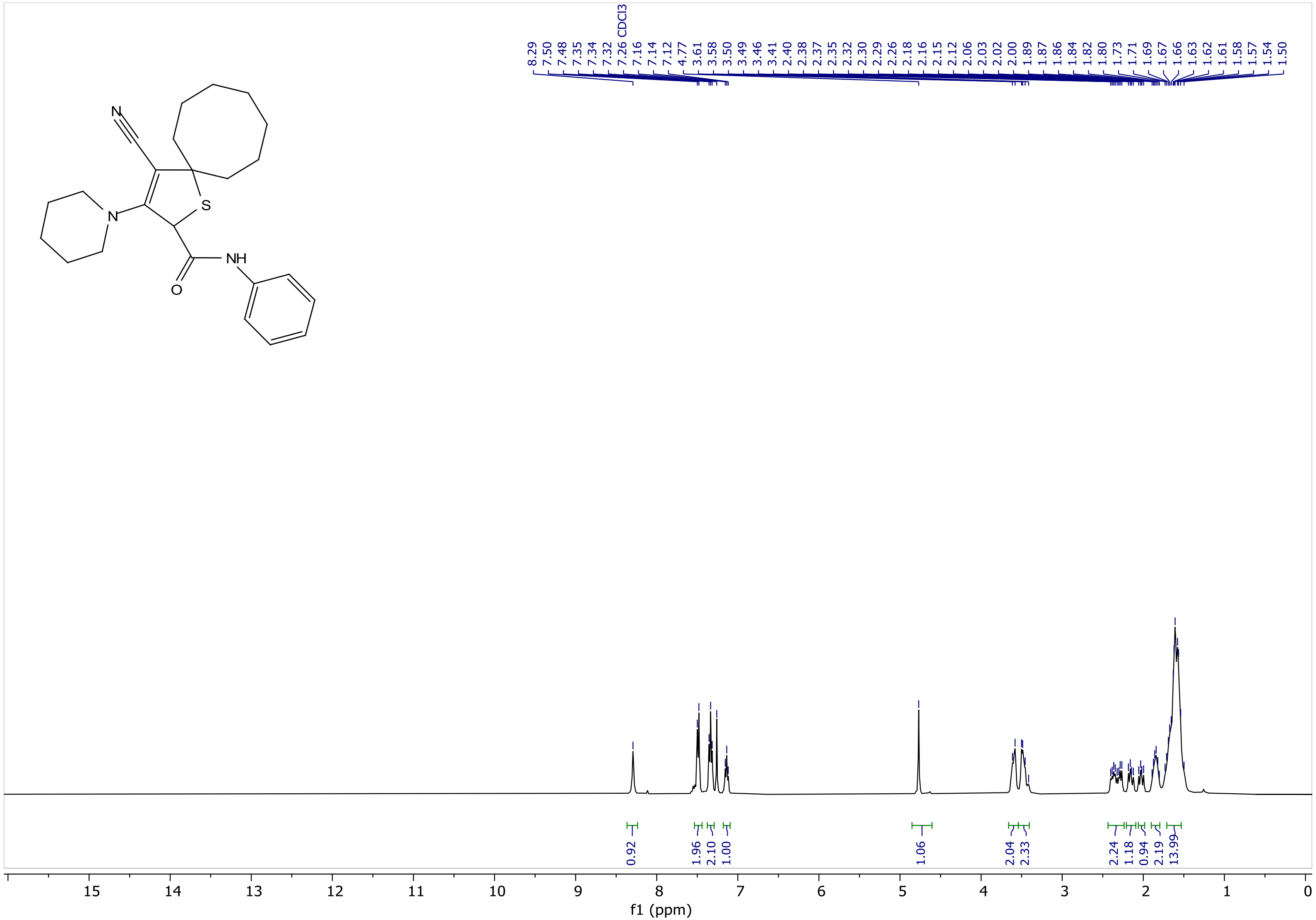

^1H NMR (400 MHz, $CDCl_3$-*d*) of **3k**

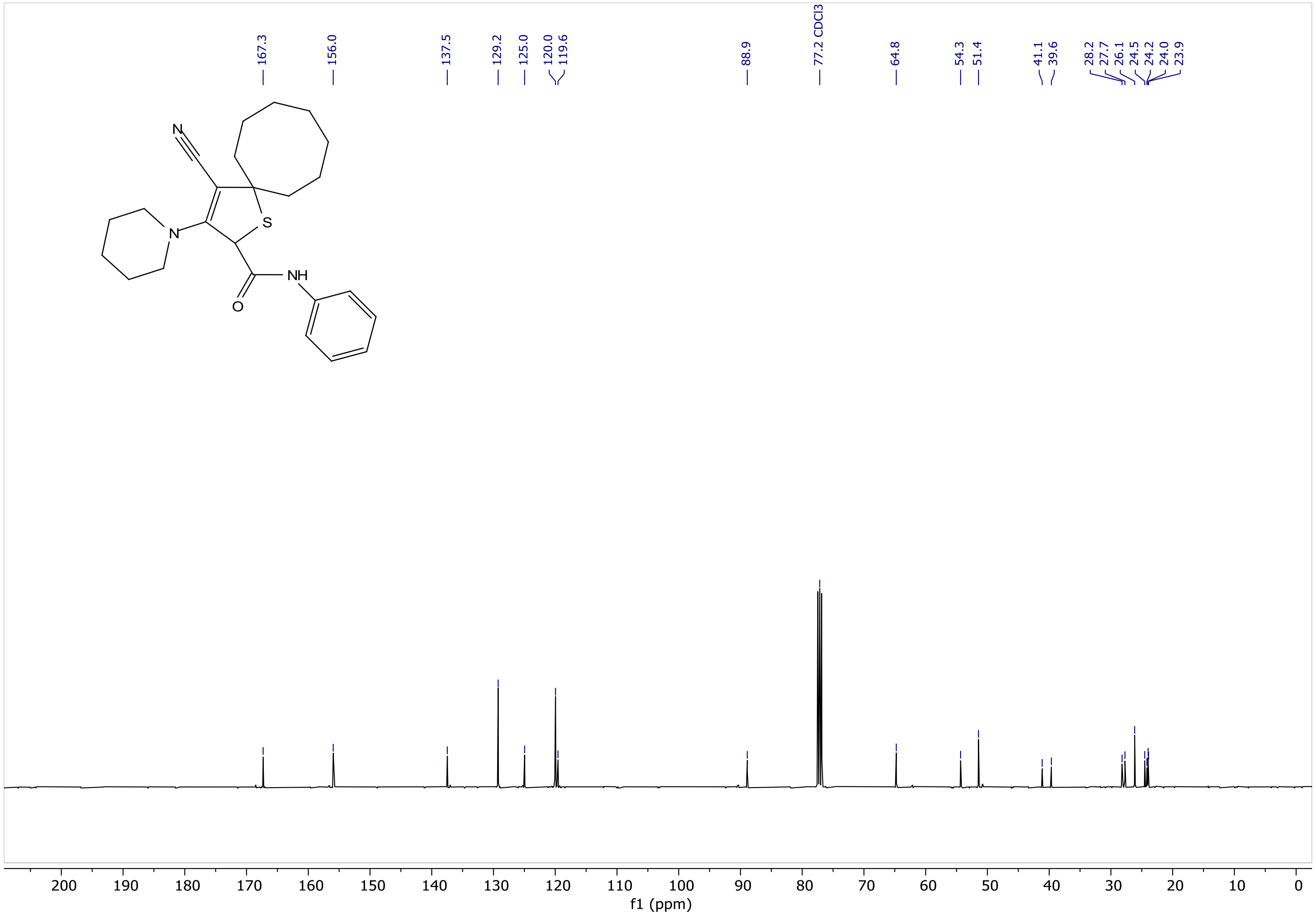

^{13}C NMR (100 MHz, $CDCl_3$-*d*) of **3k**

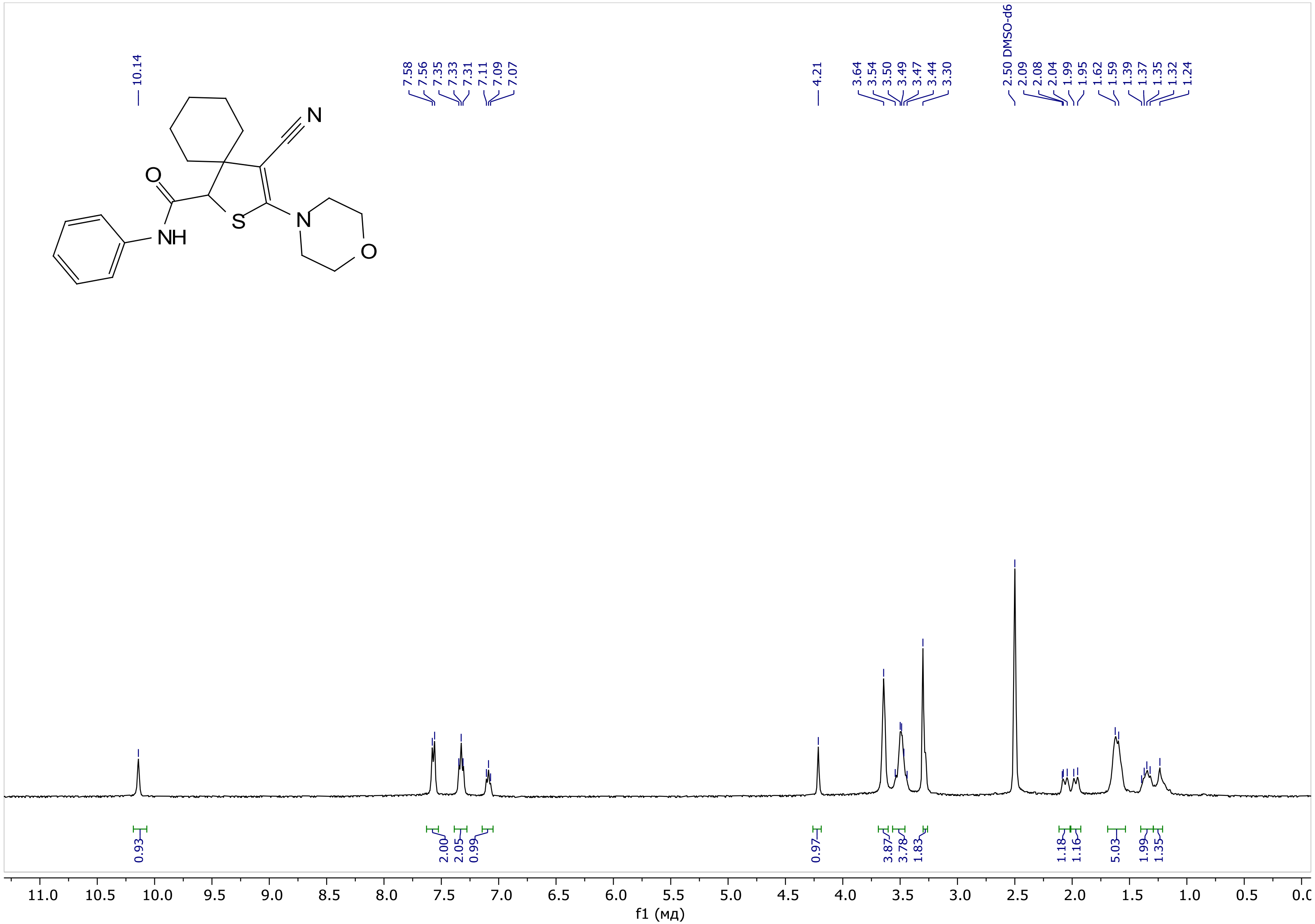

^{1}H NMR (400 MHz, DMSO-d_6) of **5a**

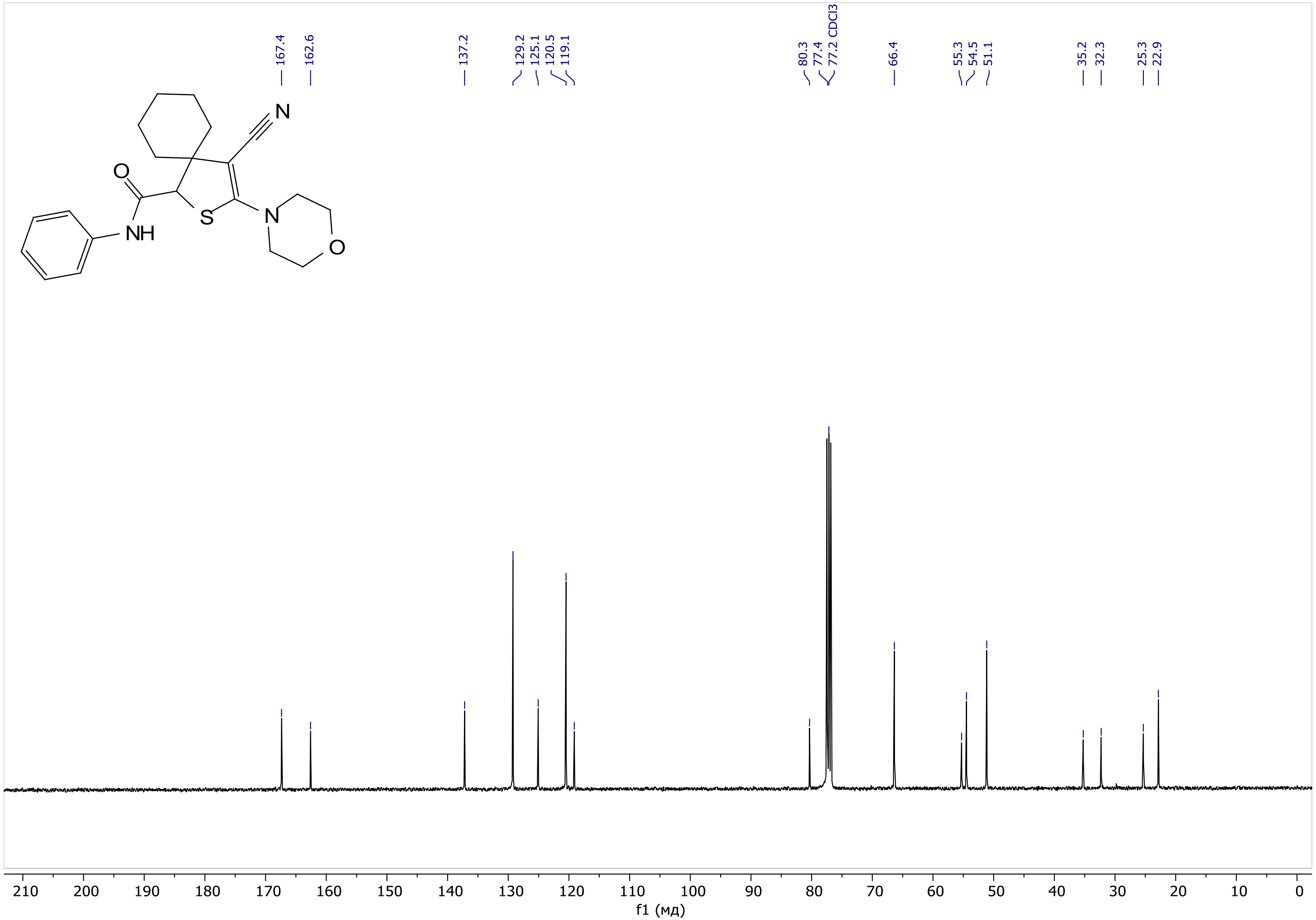

^{13}C NMR (100 MHz, $CDCl_3$-*d*) of **5a**

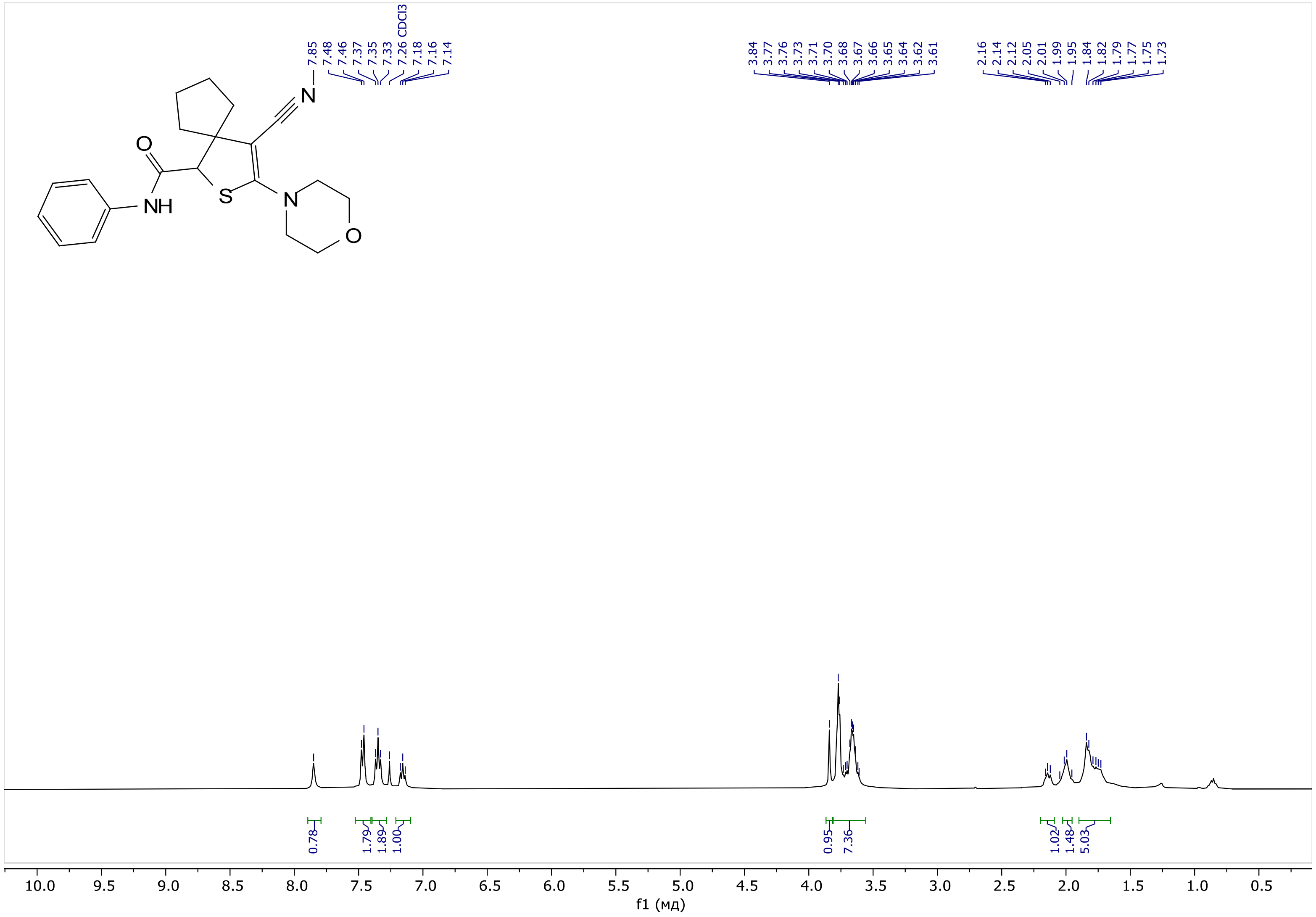

^{1}H NMR (400 MHz, $CDCl_3$–*d*) of **5b**

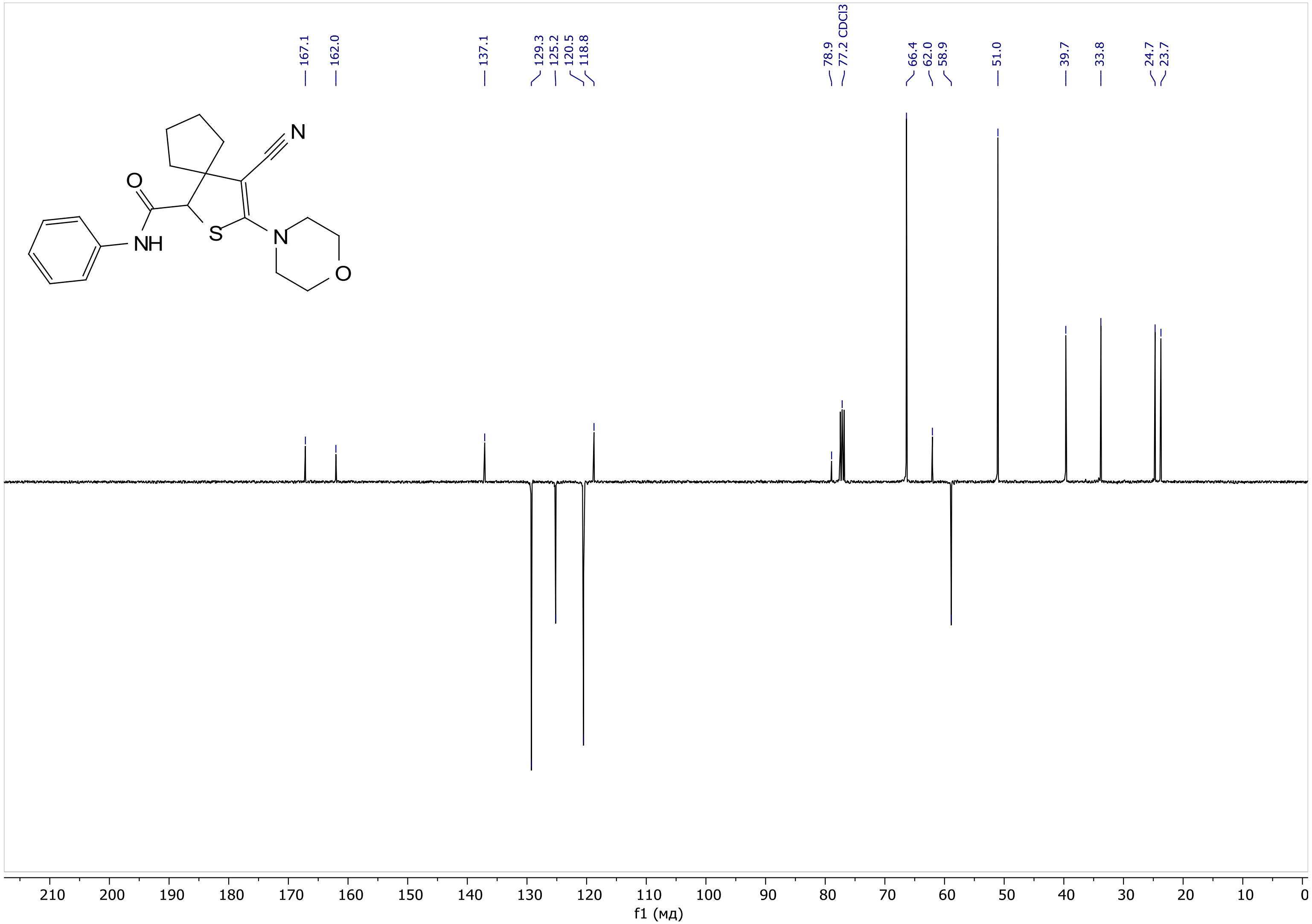

^{13}C NMR (100 MHz, $CDCl_3$-*d*) of **5b**

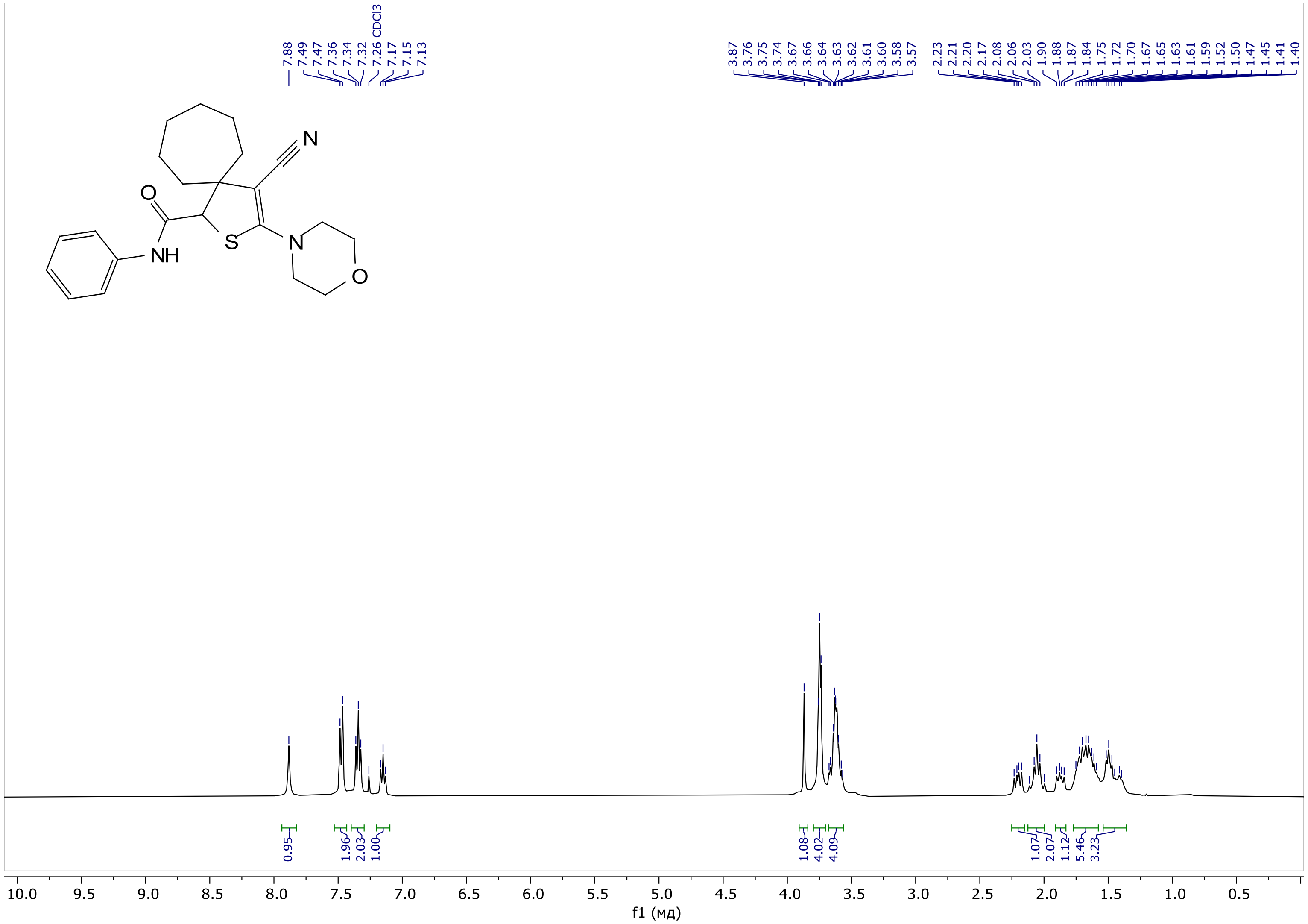

^{1}H NMR (400 MHz, $CDCl_3$–*d*) of **5c**

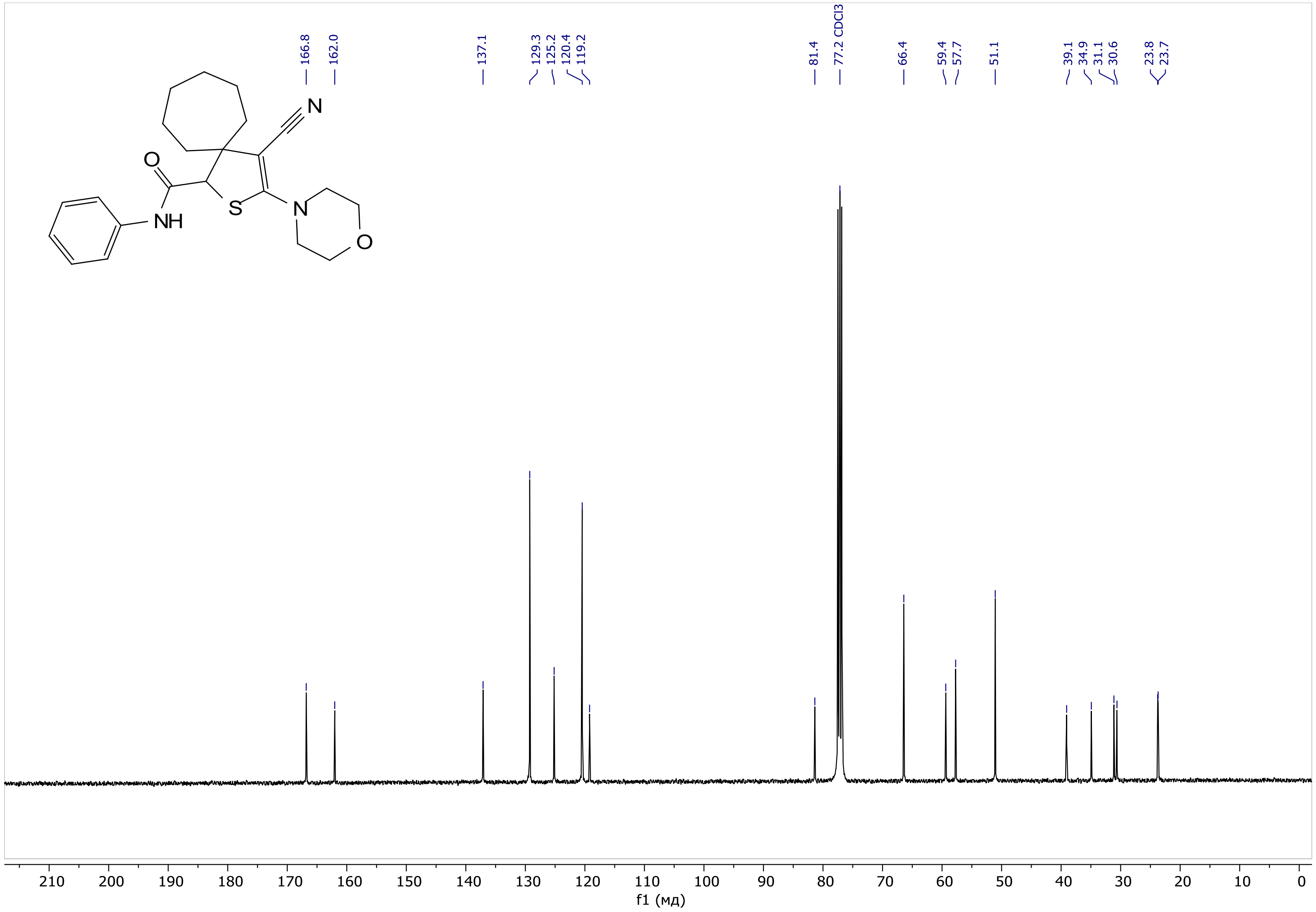

^{13}C NMR (100 MHz, $CDCl_3$-*d*) of **5c**

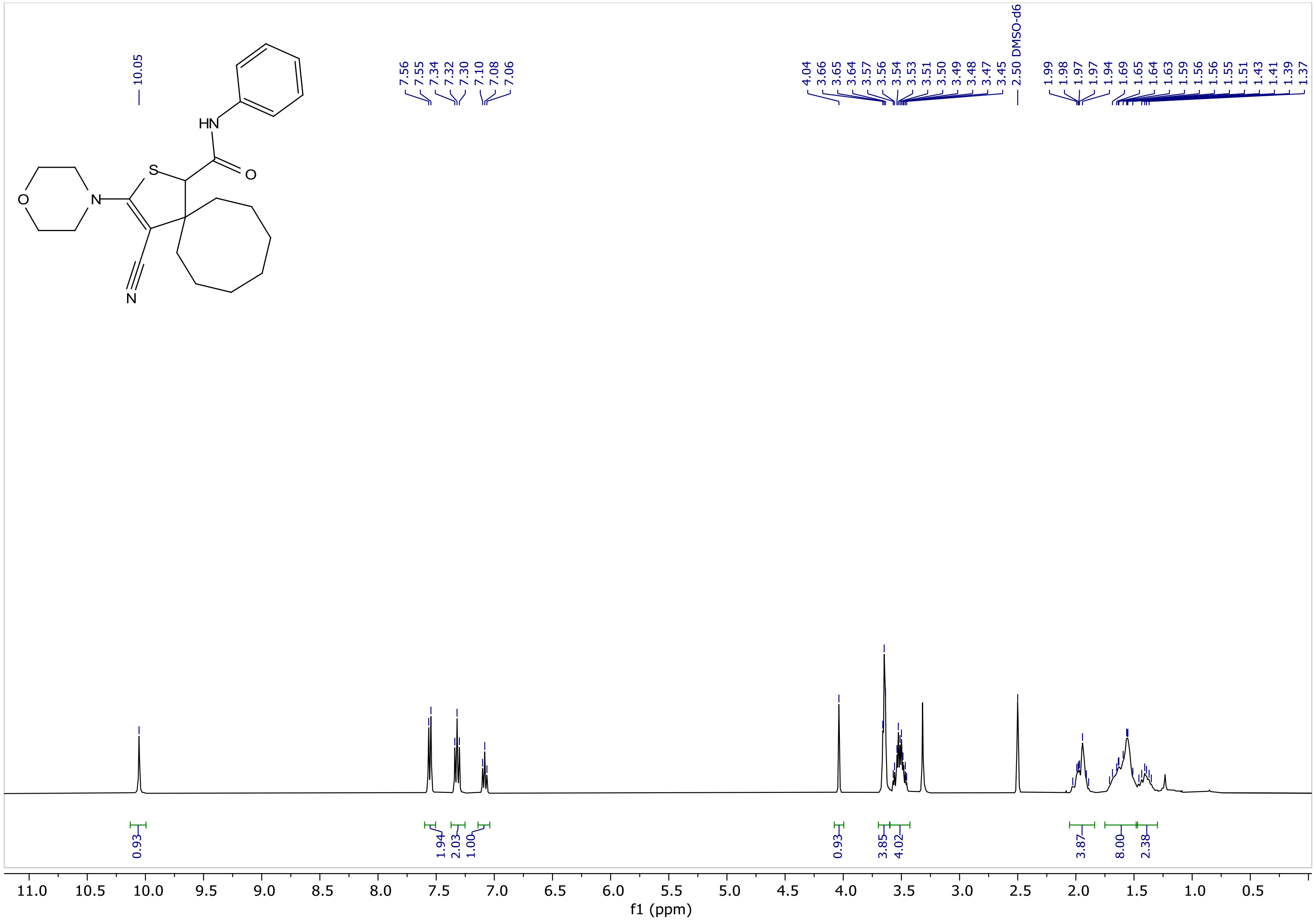

^{1}H NMR (400 MHz, DMSO–d_6) of **5d**

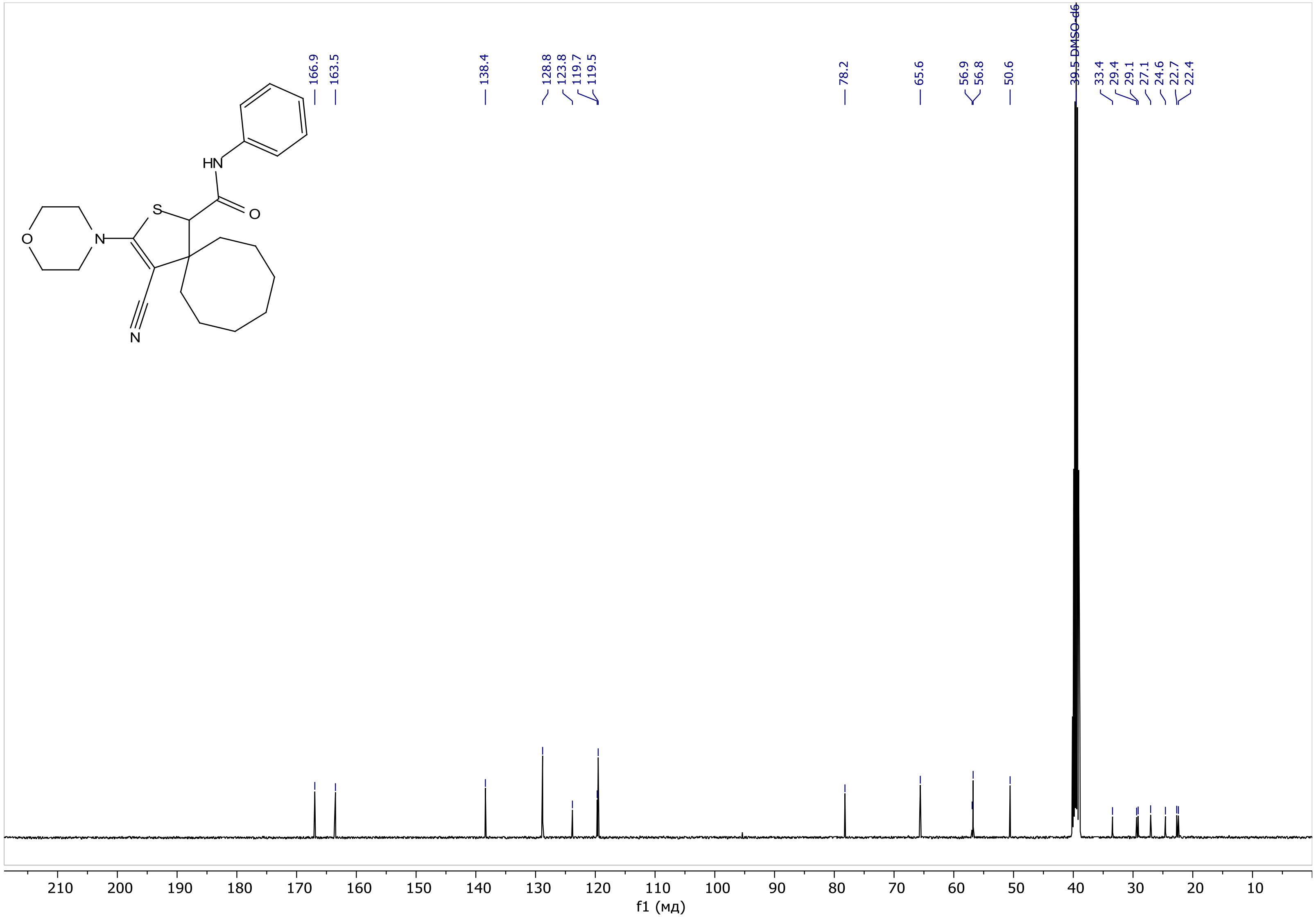

^{13}C NMR (100 MHz, DMSO–d_6) of **5c**

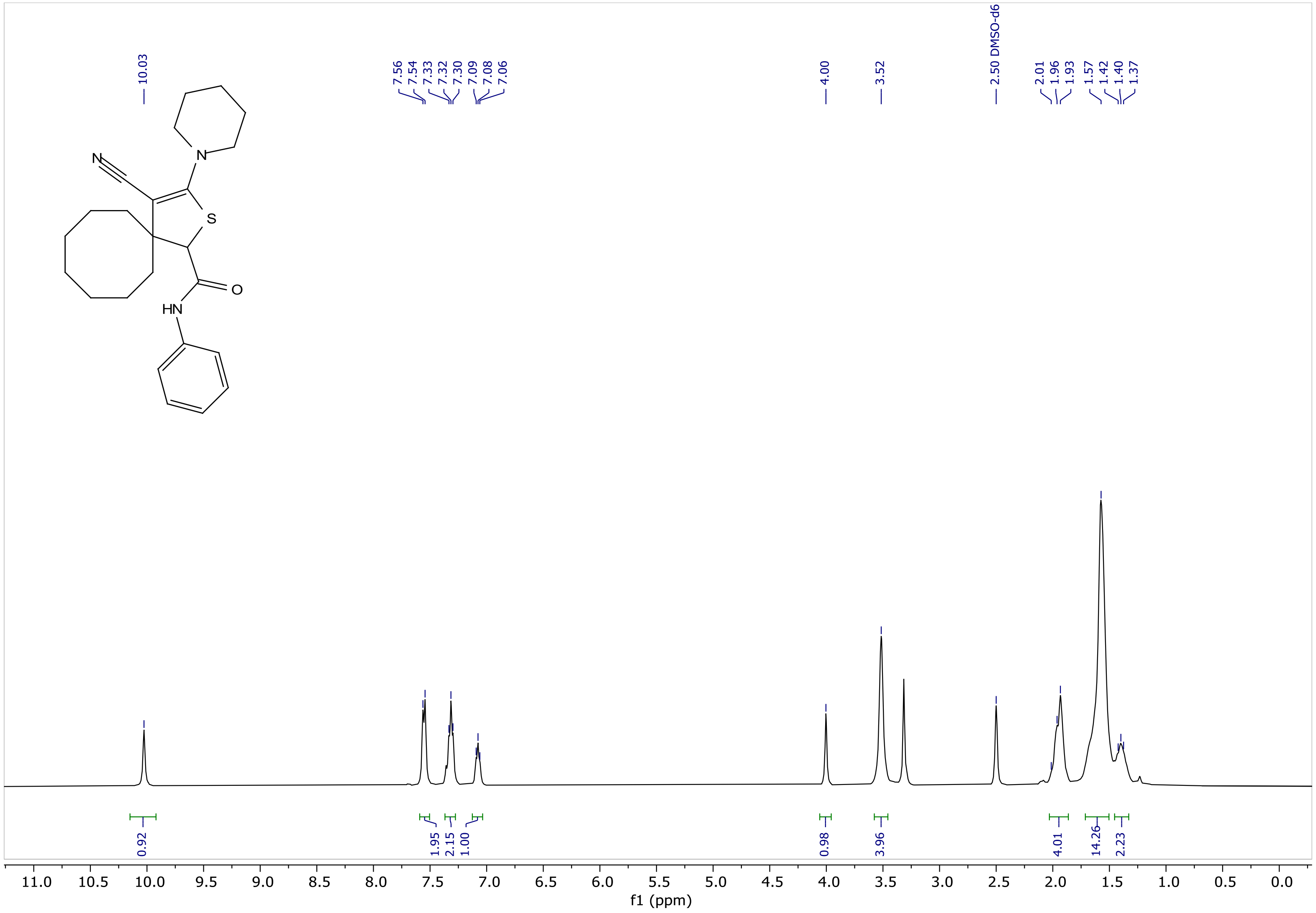

^{1}H NMR (400 MHz, DMSO-d_6) of **5e**

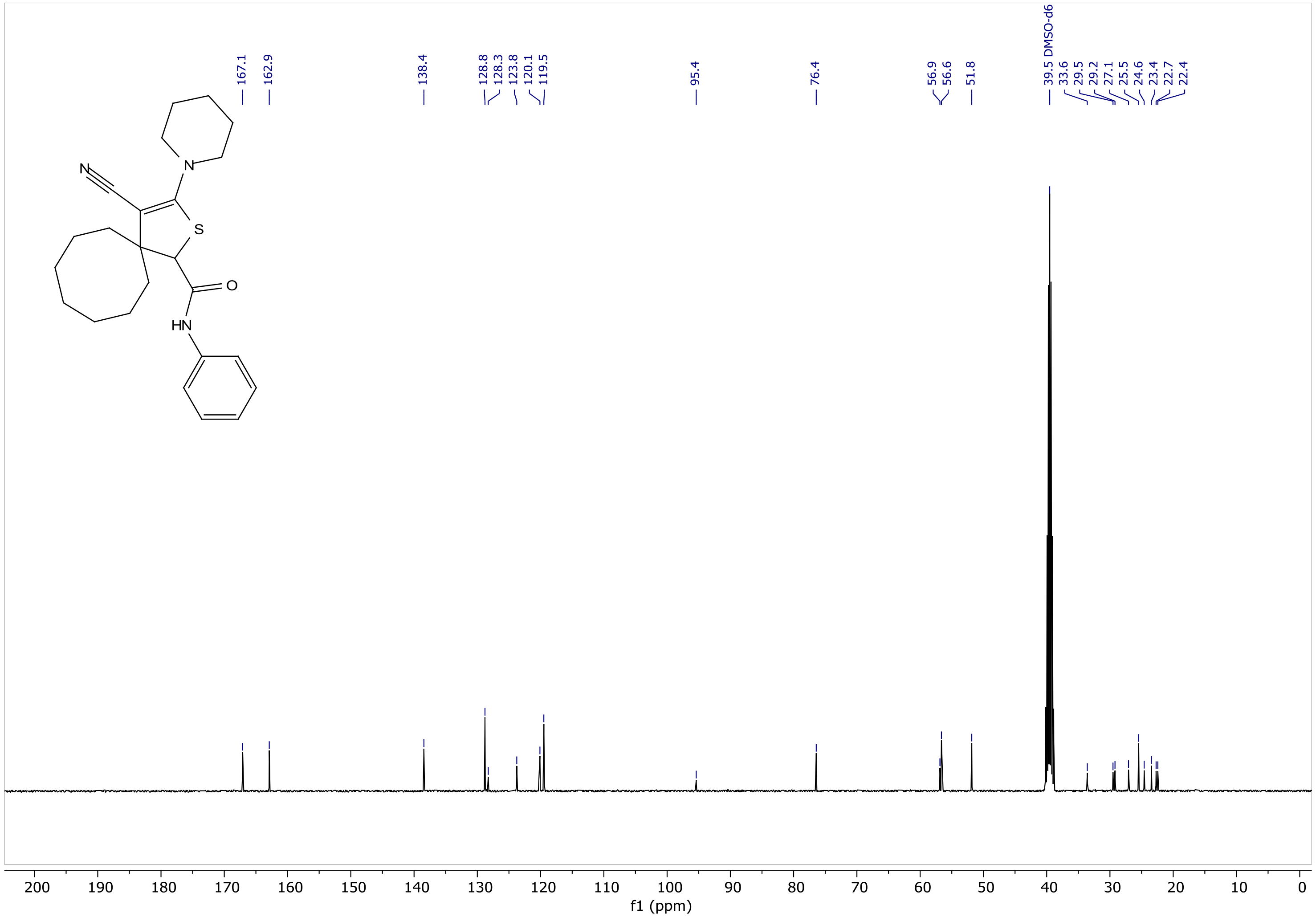

^{13}C NMR (100 MHz, DMSO-d_6) of **5e**

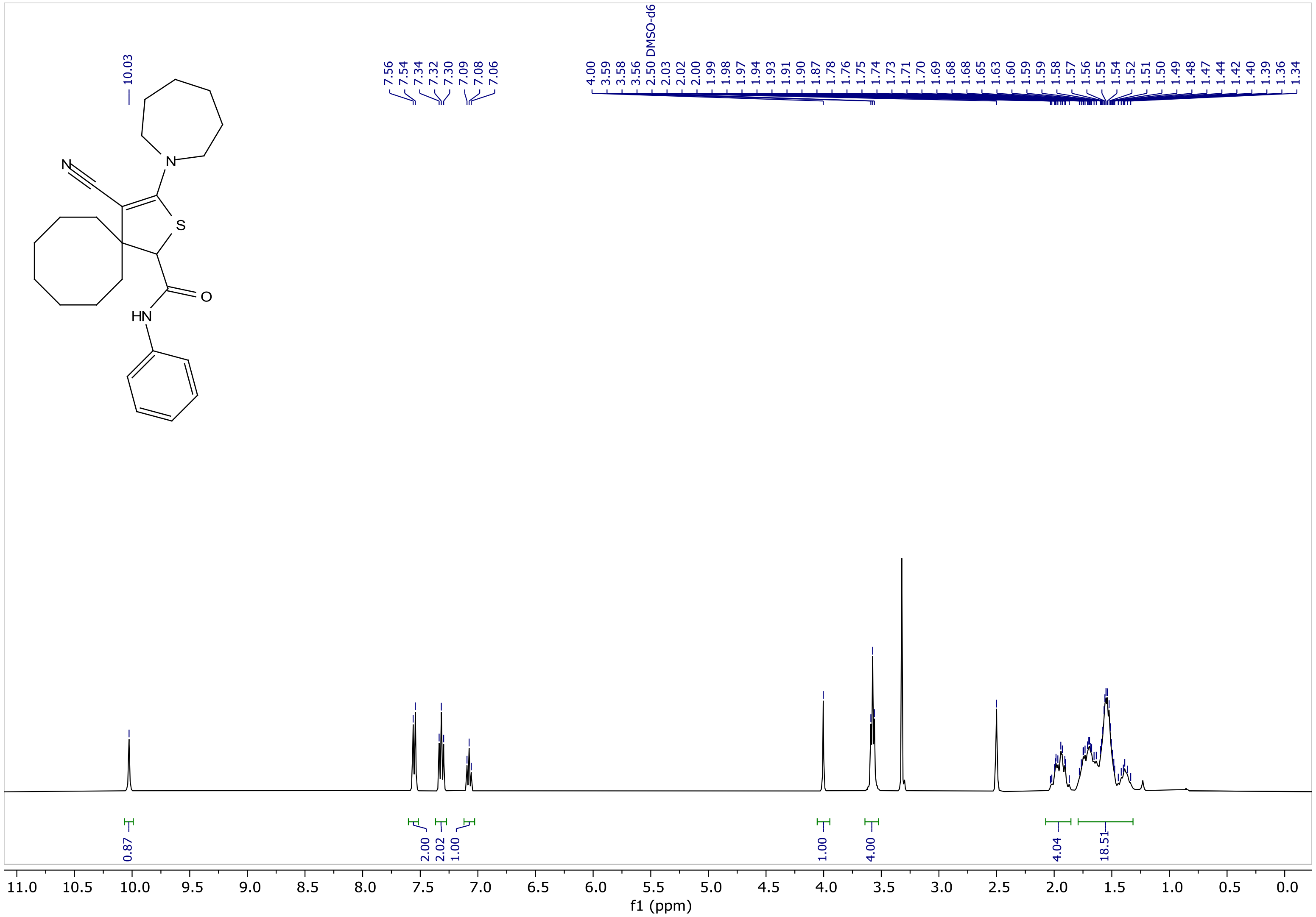

^{1}H NMR (400 MHz, DMSO-d_6) of **5f**

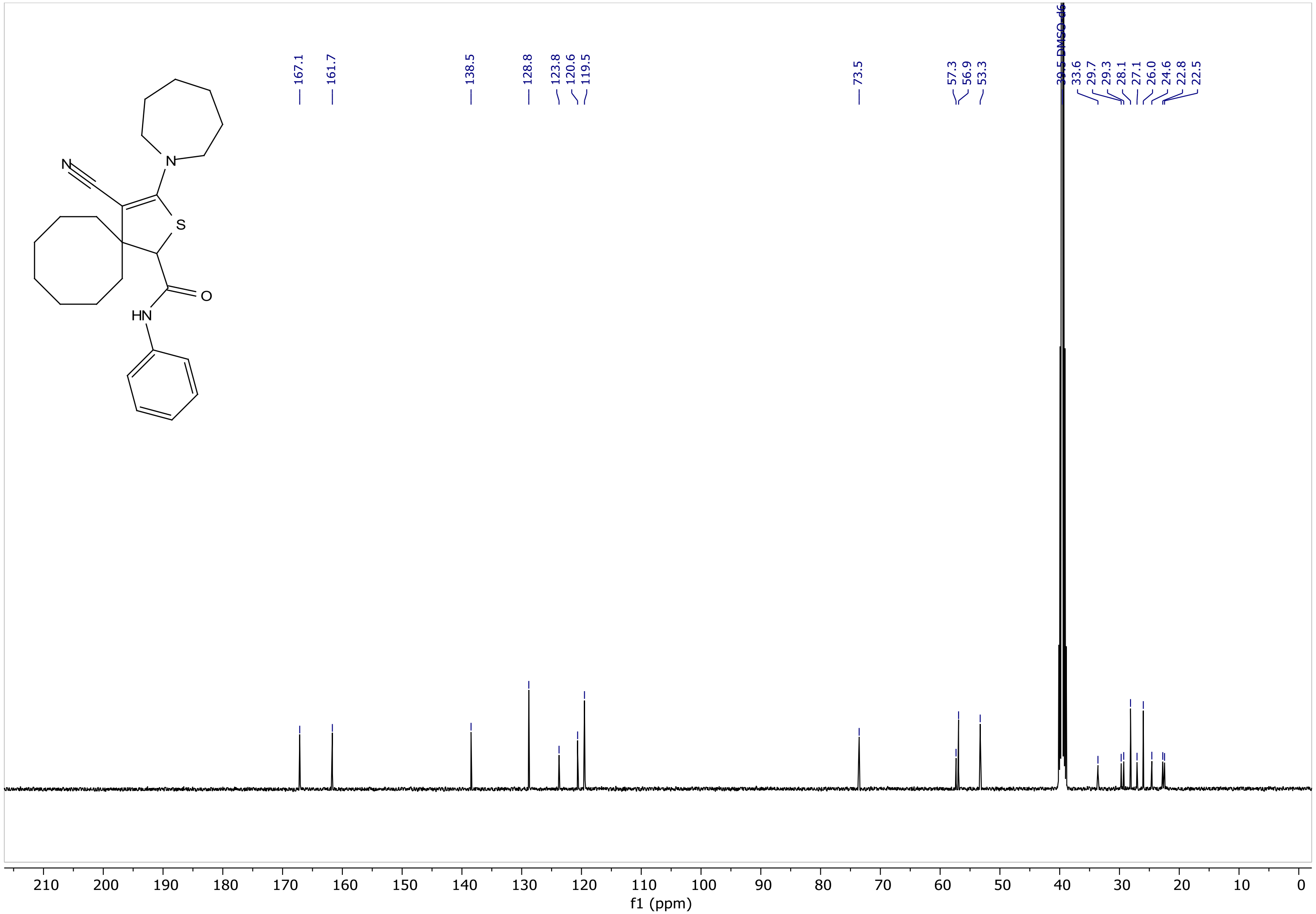

^{13}C NMR (100 MHz, DMSO-d_6) of **5f**

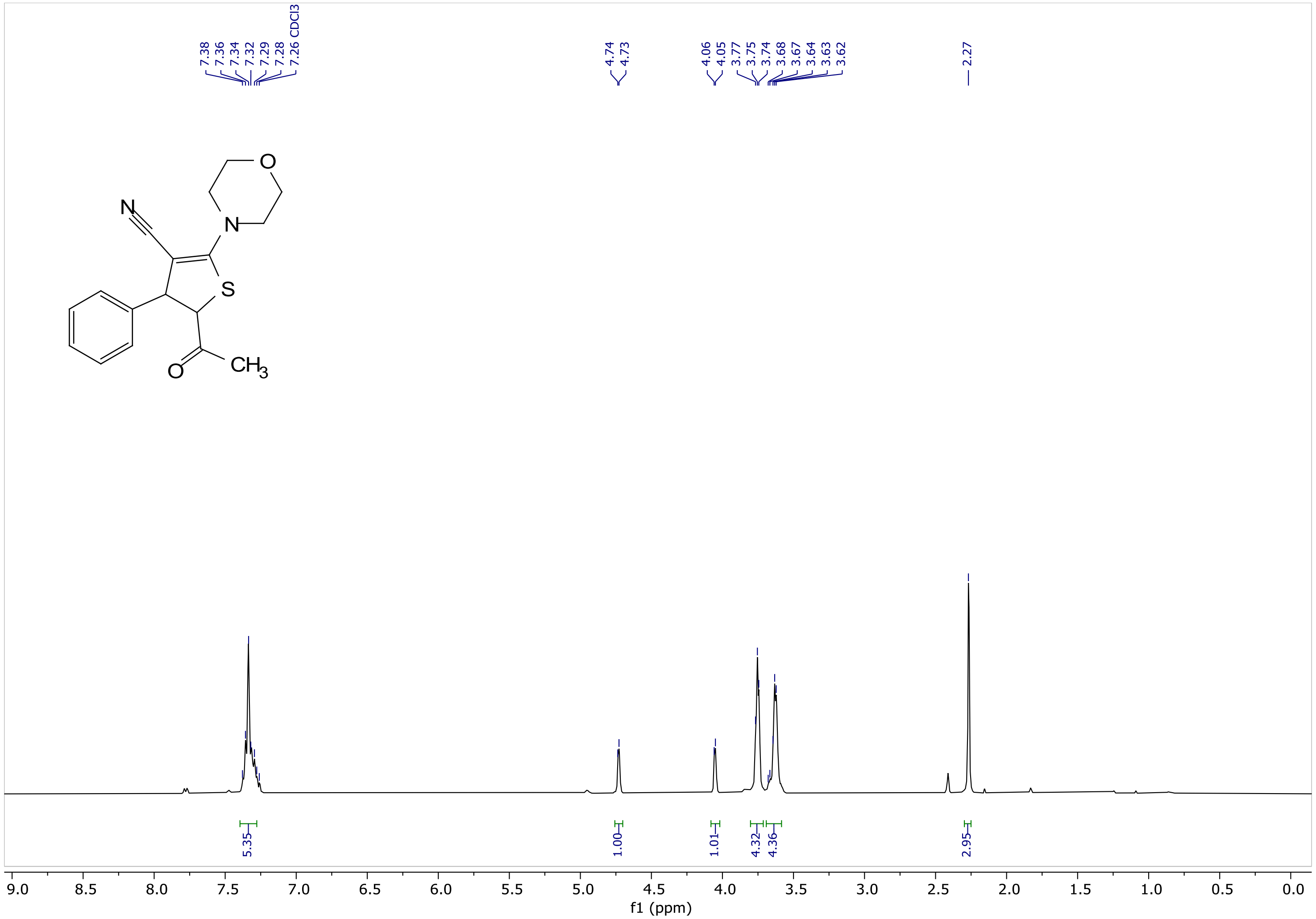

^{1}H NMR (400 MHz, $CDCl_3$–*d*) of **5g**

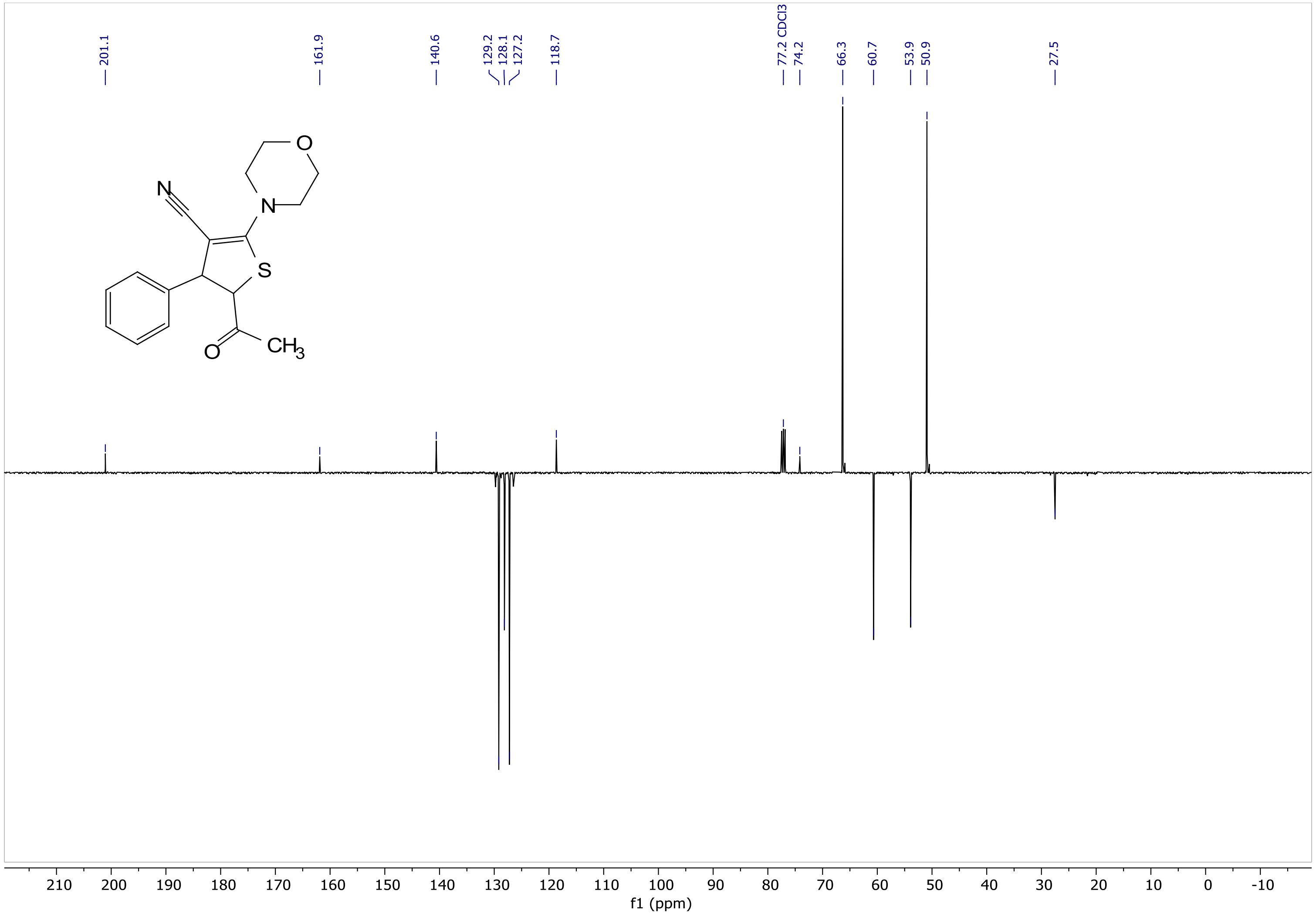

^{13}C NMR (100 MHz, $CDCl_3$–*d*) of **5g**
