# Supplementary material for: Base-promoted deacylation of 2-acetyl-2,5-dihydrothiophenes and their oxygen-mediated hydroxylation
Source: Beilstein J Org Chem. 2026 Jan 28;22:192–204. doi: 10.3762/bjoc.22.13 (PMC12862606; doi:10.3762/bjoc.22.13)
Supplement: File 4 — CheckCIF-file for compound 5g. [file Beilstein_J_Org_Chem-22-192-s004.pdf]

BEILSTEIN JOURNAL OF ORGANIC CHEMISTRY

Supporting Information

for

Base-promoted deacylation of 2-acetyl-2,5-dihydrothiophenes and their oxygen-mediated hydroxylation

Vladimir G. Ilkin, Margarita Likhacheva, Igor V. Trushkov, Tetyana V. Beryozkina, Vera S. Berseneva, Vladimir T. Abaev, Wim Dehaen and Vasiliy A. Bakulev

Beilstein J. Org. Chem. **2026,** *22,* 192–204. doi:10.3762/bjoc.22.13

CheckCIF-file for compound 5g

License and Terms: This is a supporting information file under the terms of the Creative Commons Attribution License (https://creativecommons.org/licenses/by/4.0). Please note that the reuse, redistribution and reproduction in particular requires that the author(s) and source are credited and that individual graphics may be subject to special legal provisions.
The license is subject to the *Beilstein Journal of Organic Chemistry* terms and conditions: (https://www.beilstein-journals.org/bjoc/terms)

No syntax errors found.
Please wait while processing ....

CIF dictionary
Interpreting this report

Datablock: exp_166

Bond precision:	C-C = 0.0035 A		Wavelength=0.71073
Cell:	a=9.3076(5)	b=9.3243(5)	c=10.1072(4)
	alpha=102.086(4)	beta=102.480(4)	gamma=108.253(5)
Temperature:	295 K		

	Calculated	Reported
Volume	776.19(8)	776.19(7)
Space group	P -1	P -1
Hall group	-P 1	-P 1
Moiety formula	C17 H18 N2 O2 S	C17 H18 N2 O2 S
Sum formula	C17 H18 N2 O2 S	C17 H18 N2 O2 S
Mr	314.39	314.39
Dx,g cm-3	1.345	1.345
Z	2	2
Mu (mm-1)	0.217	0.217
F000	332.0	332.0
F000'	332.38	
h,k,lmax	13,13,14	12,13,14
Nref	4745	4252
Tmin,Tmax	0.906,0.953	0.711,1.000
Tmin'	0.899	

Correction method= # Reported T Limits: Tmin=0.711 Tmax=1.000
AbsCorr = MULTI-SCAN

Data completeness= 0.896 Theta(max)= 30.500

R(reflections)= 0.0596(2973) wR2(reflections)= 0.1768(4252)

S = 1.060 Npar= 208

The following ALERTS were generated. Each ALERT has the format
test-name_ALERT_alert-type_alert-level.
Click on the hyperlinks for more details of the test.

Alert level G

```
PLAT012_ALERT_1_G No       _shelx_res_checksum Found in CIF ......     Please Check
PLAT398_ALERT_2_G Deviating  C-O-C   Angle From 120 for O1       .      110.0 Degree
PLAT793_ALERT_4_G Model has Chirality at C4          (Centro SpGr)          R Verify
PLAT793_ALERT_4_G Model has Chirality at C5          (Centro SpGr)          R Verify
PLAT933_ALERT_2_G Number of HKL-OMIT Records in Embedded .res File          4 Note
               -3  5 11,   1 11  0,  -4-10  6,   4 -3  1,
```

```
   0 ALERT level A = Most likely a serious problem - resolve or explain
   0 ALERT level B = A potentially serious problem, consider carefully
   0 ALERT level C = Check. Ensure it is not caused by an omission or oversight
   5 ALERT level G = General information/check it is not something unexpected

   1 ALERT type 1 CIF construction/syntax error, inconsistent or missing data
   2 ALERT type 2 Indicator that the structure model may be wrong or deficient
   0 ALERT type 3 Indicator that the structure quality may be low
   2 ALERT type 4 Improvement, methodology, query or suggestion
   0 ALERT type 5 Informative message, check
```

It is advisable to attempt to resolve as many as possible of the alerts in all categories. Often the minor alerts point to easily fixed oversights, errors and omissions in your CIF or refinement strategy, so attention to these fine details can be worthwhile. It is up to the individual to critically assess their own results and, if necessary, seek expert advice.

PLATON version of 26/09/2025; check.def file version of 20/09/2025

Datablock exp_166 - ellipsoid plot

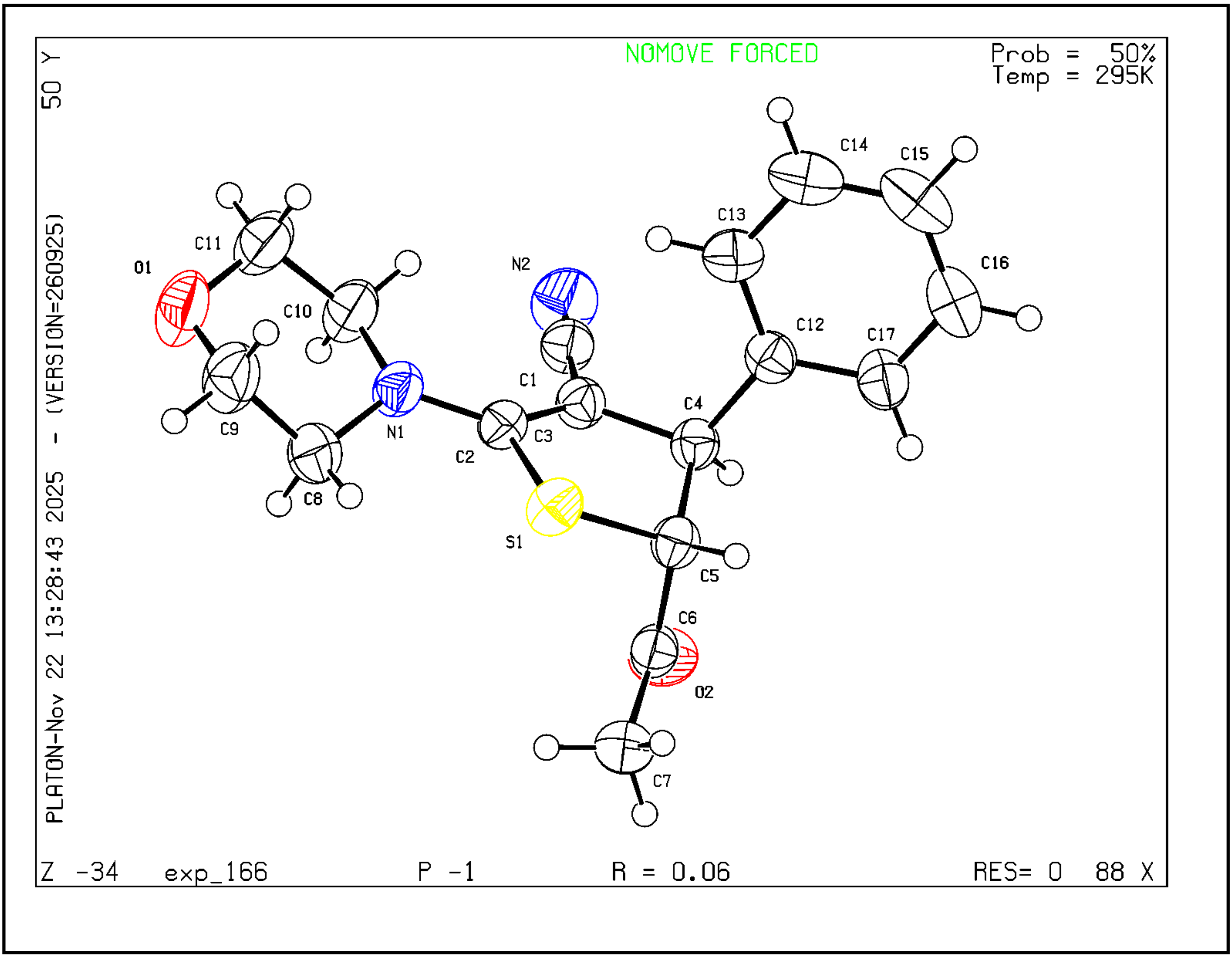

Download CIF editor (publCIF) from the IUCr
Download CIF editor (enCIFer) from the CCDC
Test a new CIF entry
